# The differentially regulated genes *TvQR1* and *TvPirin* of the parasitic plant *Triphysaria* exhibit distinctive natural allelic diversity

**DOI:** 10.1186/1471-2229-13-28

**Published:** 2013-02-18

**Authors:** Quy A Ngo, Huguette Albrecht, Takashi Tsuchimatsu, Ueli Grossniklaus

**Affiliations:** 1 Genetics Graduate Group, University of California – Davis, One Shields Avenue, Davis, CA 95616, USA; 2 Institute of Plant Biology & Zürich-Basel Plant Science Center, University of Zürich, Zollikerstrasse 107, CH-8008, Zürich, Switzerland; 3 Department of Public Health Sciences, School of Medicine, University of California – Davis, One Shields Avenue, Davis, CA 95616, USA; 4 Gregor Mendel Institute, Austrian Academy of Sciences, Dr. Bohr-Gasse 3, 1030, Vienna, Austria

**Keywords:** Parasitic plants, Haustorium, Nucleotide diversity, Allelic polymorphism, Population genetics, Transcriptional responses

## Abstract

**Background:**

Plant parasitism represents an extraordinary interaction among flowering plants: parasitic plants use a specialized organ, the haustorium, to invade the host vascular system to deprive host plants of water and nutrients. Various compounds present in exudates of host plants trigger haustorium development. The two most effective haustorium inducing factors (HIFs) known for the parasitic plant *Triphysaria versicolor* (*T. versicolor*) are peonidin, an antioxidant flavonoid, and 2,6-dimethoxybenzoquinone (DMBQ), an oxidative stress agent. To date, two genes involved in haustorium initiation in *T. versicolor* have been identified: *TvQR1*, a quinone oxidoreductase that generates the active HIF from DMBQ, and *TvPirin*, a transcription co-factor that regulates several other DMBQ- responsive and –non-responsive genes. While the expression of these genes in response to DMBQ is well characterized, their expression in response to peonidin is not. In addition, the pattern of polymorphisms in these genes is unknown, even though nucleotide changes in *TvQR1* and *TvPirin* may have contributed to the ability of *T. versicolor* to develop haustoria. To gain insights into these aspects, we investigated their transcriptional responses to HIFs and non-HIF and their natural nucleotide diversity.

**Results:**

Here we show that *TvQR1* and *TvPirin* are transcriptionally upregulated by both DMBQ and peonidin in *T. versicolor* roots. Yet, while *TvQR1* also responded to juglone, a non-HIF quinone with toxicity comparable to that of DMBQ, *TvPirin* did not. We further demonstrate that *TvPirin* encodes a protein shorter than the one previously reported. In the *T. versicolor* natural population of Northern California, *TvQR1* exhibited remarkably higher molecular diversity and more recombination events than *TvPirin*, with the highest non-synonymous substitution rate in the substrate recognition and catalytic domain of the TvQR1 protein.

**Conclusion:**

Our results suggest that *TvQR1* and *TvPirin* have most likely evolved highly distinct roles for haustorium formation. Unlike *TvPirin*, *TvQR1* might have been under diversifying selection to maintain a diverse collection of polymorphisms, which might be related to the recognition of an assortment of HIF and non-HIF quinones as substrates for successful haustorial establishment in a wide range of host plants.

## Background

The plant kingdom is usually associated with autotrophy as most plants produce their own nutrients via photosynthesis. Plant parasitism presents a divergence from this generalization as parasitic plants derive all or part of their nutrients and water from their host plants. About 4,000 parasitic plant species are widely distributed among various taxa and over diverse environments, ranging from arctic to tropical climates. Some of the best known parasitic plants include the Christmas ornament mistletoe, the world largest blooming flower *Rafflesia*, the fragant oil producing sandalwood, and the debilitating agricultural weeds dodder, witchweed (*Striga*) and broomrape (*Orobanche*). Although diverse in morphology, reproductive aspects, and life habitats, all parasitic plants share a specialized organ - the haustorium - which they evolved to assure a successful life cycle [1]– [4]. Parasitic plants develop haustoria either near their root tips or along their stems in response to selective chemical factors released by their hosts [5,6]. The haustorium initiates via localized cell expansion and division that is coupled with haustorial hair growth in many species. Following attachment and penetration of the host’s roots or stems, the haustorium establishes a vascular continuity between the parasite and its host through which water and nutrients are channeled to the parasite to benefit its own growth and development. By so doing, parasitic plants disturb many developmental and physiological aspects of host plants [1,2,5,6]. Consequently, parasitic plants have a tremendous impact on community ecology among plants, animals, microbes, and even the surrounding physical environment [7]. Therefore, studies on parasitic plants encompass a range of interests, including anatomy and development, cellular physiology, gene regulation, population genetics, phylogeny, ecology and evolution. Here, we focus on a variety of aspects of host perception and recognition by parasitic plants.

The perception and recognition by parasitic plants of haustorium inducing factors (HIFs) released by host plants is best documented in root parasites of the Orobanchaceae family (many previously classified with Scrophulariaceae). The first three HIFs identified, xenognosin (i.e., host recognition factor) A, xenognosin B, and 2,6-dimethoxybenzoquinone (DMBQ), are phenolic compounds [8]– [10]. Additionally, various quinone and flavonoid HIFs were subsequently identified and characterized [11,12]. Further studies on HIF biogenesis, activity, and recognition in the two root hemiparasites *Striga* spp. and *Triphysaria* spp. have shed light onto the chemical signaling by DMBQ, which is involved in the first steps of the haustorium developmental pathway [13]– [16]. The genus *Striga* contains prolific weeds causing substantial damage to cereal crops mostly in Africa [17,18], whereas *Triphysaria* is a wild native genus in the grassland communities of Northern American coastlands [19]. The current model for haustorium initiation proposes that certain unidentified peroxidases or laccases released by parasitic roots catalyze the production of DMBQ at the host contact site, which diffuses towards the parasitic plant [14,15]. Next, a quinone oxidoreductase from the parasite converts DMBQ from its inactive form into the transiently active single-electron free radical form with the suitable redox potential for haustorium induction. This process is hypothesized to be the first step of the haustorium organogenesis pathway [13,16].

To date, only two parasitic plant genes involved in haustorium development have been identified, *TvQR1* and *TvPirin*, both from the root parasite *Triphysaria versicolor* (*T. versicolor*) [13,20]. The TvPirin protein is a general transcription co-factor that up-regulates several DMBQ-responsive and –non-responsive genes [20]. *TvQR1* encodes the aforementioned quinone oxidoreductase, which catalyses the reduction of quinones [13]. In comparison to the HIF DMBQ, this enzyme displays even higher catalytic activities for three other non-HIF quinones, one of which is juglone [13]. Consistent with its biochemical role, *TvQR1* gene expression is rapidly up-regulated in *T. versicolor* root tips exposed to either maize or *Arabidopsis* root exudates containing active HIFs, DMBQ or the non-HIF juglone [11,21,22]. By contrast, up-regulation of *TvPirin* in root tips only occurs in response to DMBQ and *Arabidopsis* root exudates [21].

Most identified HIFs are quinones like DMBQ, which are known to be abundant in plant exudates and have also been associated with allelopathic effects (i.e., inhibition of neighboring plant growth and development) and defense against pathogens [23]. At concentrations below 10^-4^ M, quinone HIFs induce haustorium development in parasitic plants [8,11,24,25]. However, at concentrations between10^-4^ M and 10^-3^ M, they impart the negative effects of oxidative stress to parasitic plants that result in root necrosis and a reduction in the frequency of haustorium formation [8,11,24,25]. Further underscoring this quinone toxicity is the enrichment of transcripts involved in oxidative stress and detoxification pathways in *T. versicolor* root tips following exposure to DMBQ or host root exudates [21]. Like DMBQ, juglone is a well-known allelopathic compound present copiously in root exudates of the black walnut tree [26], but unlike DMBQ, juglone lacks the haustorium-inducing capability [21,24]. The fact that juglone is a better substrate for TvQR1 than DMBQ [13] correlates with the faster and stronger up-regulation of *TvQR1* expression in *T. versicolor* root tips upon juglone exposure [21,22]. The only non-quinone HIF known to date is the flavonoid peonidin [11]. Peonidin belongs to the anthocyanin class of compounds present in red colored flowers and fruits that act as potent antioxidants by scavenging free radicals, and play various roles in plant development and stress protection [27]– [29]. Although peonidin is a HIF as potent as DMBQ for *T. versicolor*[11], its role in the regulation of *TvQR1* and *TvPirin* gene expression during haustorium development has not yet been investigated. Furthermore, the availability of peonidin, a non-toxic HIF, DMBQ, a toxic HIF, and juglone, a toxic non-HIF, offers the opportunity to utilize these three chemicals to de-couple the two seemingly intertwined pathways triggered by the quinone HIFs - namely the oxidative stress response and haustorium organogenesis.

To understand how *TvPirin* and *TvQR1* contribute to the perception of various phytochemicals present in the rhizosphere and, thus, to the initiation of haustorium development, it is essential to survey their patterns of polymorphism in natural populations of *T. versicolor*. Since so far only a single allele of each of these two genes has been reported [13,20]– [22], we examined the pattern of nucleotide polymorphism of *TvQR1* and *TvPirin* by using 20 *T. versicolor* individual plants from natural populations of the Northern Californian grasslands. We found strikingly higher allelic diversity in *TvQR1* compared to *TvPirin*, with the highest non-synonymous substitution rate in the catalytic domain of the TvQR1 protein. In addition, we provide evidence that *TvPirin* encodes a protein shorter than the one previously reported [20], and show that *TvQR1* and *TvPirin* are differentially regulated by HIFs and non-HIF. Our results suggest that *TvQR1*, unlike *TvPirin*, has maintained a high level of nucleotide diversity, which might reflect its important role in the perception of various phytochemicals from host plants.

## Results and discussion

### *TvQR1* and *TvPirin* are differentially regulated by haustorium inducers and non-inducers

**Figure 1:**
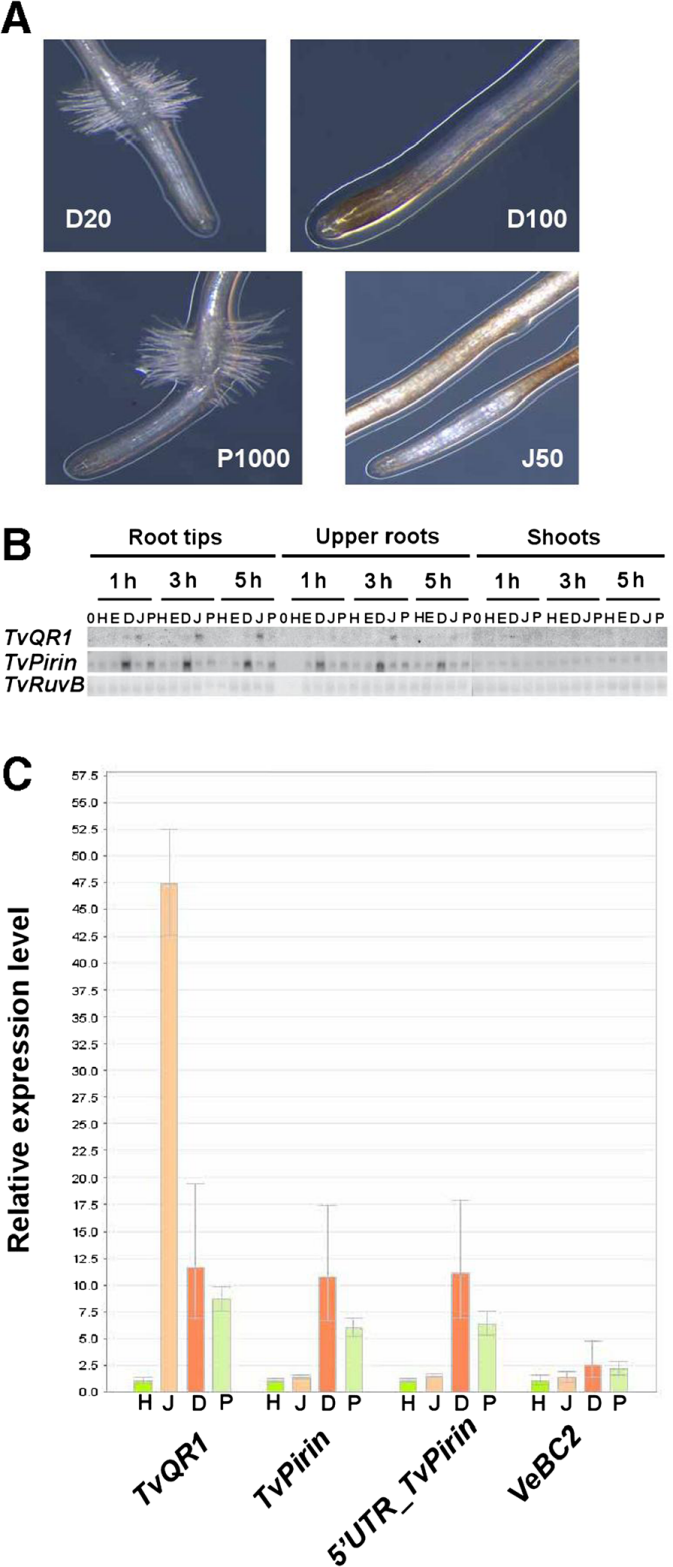
**Expression of *TvQR1 *and *TvPirin *under haustorium-inducing and -non-inducing conditions. (A)** Toxicity effects of HIFs and non-HIFs on *T. versicolor* roots and haustorium formation. D20: 20 μM DMBQ, D100: 100 μM DMBQ, J50: 50 μM juglone, P1000: 1 mM peonidin. **(B)** Northern blot analysis of the spatial and temporal expression of *TvQR1* and *TvPirin* following treatment with HIFs and non-HIFs. 0: no treatment, H: water, E: 0.1% ethanol (solvent of peonidin), D: 20 μM DMBQ, J: 10 μM juglone, P: 30 μM peonidin. *TvRuvB DNA helicase* is a constitutively expressed gene. **(C)** Differential expression of *TvQR1* and *TvPirin* under different HIFs and non-HIFs measured by qRT-PCR. H: water, J: 10 μM juglone, D: 20 μM DMBQ, P: 30 μM peonidin.

To compare the regulation of *TvQR1* and *TvPirin,* we selected two HIFs and a non-HIF displaying overlapping and differential biological properties on parasitic plants: DMBQ with the dual role of an oxidative stress agent and haustorium inducer, peonidin as a non-toxic HIF, and juglone as a non-HIF quinone and strong oxidative stress agent. In *T. versicolor in vitro* haustoria formation assays, both DMBQ and juglone reduced haustorium formation frequency and caused root necrosis at concentrations of ≥ 50 μM (Table 1 and Figure 1A), in agreement with previous observations [11,24]. Although either 10 μM DMBQ or 30 μM juglone alone did not result in root necrosis or a reduced frequency of haustorium formation, the combination of both treatments did (Table 1), further substantiating quinone toxicity to the parasite. On the other hand, peonidin had no toxic effects on *T. versicolor,* even at 1 mM concentration (Table 1 and Figure 1A). Using Northern blot hybridizations, we examined the spatial and temporal pattern of transcript levels of *TvQR1* and *TvPirin* in response to DMBQ, peonidin, and juglone in three areas of *T. versicolor* seedlings - root tips, the remainder of the roots, and shoots - at 1, 3, and 5 hours (h) after treatment. *TvQR1* was more strongly up-regulated by the non-HIF juglone than by the HIF DMBQ (Figure 1B), corroborating its higher substrate affinity for juglone [13]. On the other hand, *TvPirin* was similarly up-regulated by both the HIFs DMBQ and peonidin, but not by the non-HIF juglone (Figure 1B). These up-regulation patterns were observed only in roots but not in shoots (Figure 1B), consistent with a role for *TvQR1* and *TvPirin* in haustorium development only in the root. We further confirmed the differential expression of *TvQR1* and *TvPirin* in response to these HIFs and non-HIFs in *T. versicolor* root tips at 2–3 h post-treatment by reverse transcription quantitative polymerase chain reaction (RT-qPCR) (Figure 1C). Our results suggest that *TvQR1* is associated with both the oxidative stress response and haustorium initiation, while *TvPirin* appears to be mainly involved in haustorium development. Determining whether *TvPirin* is exclusively regulated by HIFs and only involved in haustorium initiation would require the analysis of additional non-toxic HIFs, which so far have not been identified.

**Table 1:** **Effects of different HIFs and non-HIFs on haustorium formation and root necrosis in *T. versicolor***

**Treatment**	**Water**	**DMBQ 10 μM**	**DMBQ 100 μM**	**Juglone 50 μM**	**DMBQ10μM + juglone 30 μM**	**Peonidin 30 μM**	**Peonidin 1 mM**
% responsive plants	0	85	60	0	33	78	90
Presence of root necrosis	no	no	yes	yes	yes	no	no

Haustorium formation and root necrosis were scored on *T. versicolor* seedlings (n = 60 to 66) 24 hours after treatment.

### *TvPirin* encodes a 288 amino acid long protein

The *TvPirin* gene was initially identified as an incomplete EST (Accession BE574904.1) from a suppressive subtractive hybridization (SSH) library enriched for *T. versicolor* root tip transcripts that are up-regulated by DMBQ [22,30]. To determine the sequence of the full length *TvPirin* transcript, we performed 5′ and 3′ rapid amplification of cDNA ends (RACE) reactions and RT-PCR with primers complementary to both ends of the RACE clones using a pooled RNA population isolated from *T. versicolor* root tips with haustoria induced by 20 μm DMBQ. Alignment of the 5, 6, and 4 independently recovered 5′ RACE, 3′ RACE, and full-length cDNA clones, respectively, revealed a *TvPirin* open reading frame (ORF) encoding a 288 amino acid (aa) long protein with a 5′ untranslated region (UTR) of 64–68 base pairs (bp) (Additional file 1). However, the previously published *TvPirin* cDNA sequence (Accession JN606867 in [20]; clone TrVeBC2_244713 in [31] and in http://ppgp.huck.psu.edu/search.php) has a coding capacity for a 322 aa long protein [20]. This cDNA sequence was created by contig assemblies following 454 and Illumina sequencing of the *T. versicolor* transcriptome [31]. In comparison to the sequence of the *TvPirin* full length cDNA reported here (Additional file 2), that of the TrVeBC2_244713 clone contains an additional 80 bp in the 5′UTR and an extra 100 bp in the 3′UTR ( http://ppgp.huck.psu.edu/search.php; [31]). The extended 5′UTR in clone TrVeBC2_244713 contains an ATG codon that was assigned as the translation start codon and explains the longer coding sequence of the previously published *TvPirin* ORF (Accession JN606867 in [20]). In order to resolve these discrepancies regarding the *TvPirin* transcript 5′UTR and ORF, we performed *in silico* experiments and quantitative transcriptional assays.

First, BLAST-searches for the presence of other *TvPirin* contig assemblies from the *T. versicolor* transcriptome ( http://ppgp.huck.psu.edu/search.php, [31]) uncovered seven clones with homology to the *TvPirin* cDNA clone TrVeBC2_244713 (Additional file 2A). Two of these clones (TrVe62GuB1_70666 and TrVe3GB1_60414) had the same orientation as the *TvPirin* cDNA and lacked the extended 5′UTR. The remaining 5 clones had the reverse orientation and 5′ UTRs of variable lengths. One of these clones (TrVeGnuB1_1027) had the same length and shared 100% identity in reverse complementation order to the TrVeBC2_244713 *TvPirin* cDNA. Another reverse-complemented clone (TrVe61FuB1_8246) shared discontinuous identity with the *TvPirin* cDNA sequences in both orientations (Additional files 2A and 3). These observations suggest that these seven clones represent either transcripts from genes other than *TvPirin* or artifacts of the contig assembly. Similar BLAST-searches in the *T. versicolor* SSH root tip contig libraries enriched for transcripts up-regulated by DMBQ ( http://www.plantsciences.ucdavis.edu/yoder/lab/Sequence_index.html) identified two clones, Contig5118 and Contig492, with homologies to the 5′ and 3′ half of our *TvPirin* cDNA sequence, respectively, and without the extended 5′UTR of the TrVeBC2_244713 clone (Additional file 2A).

Second, we performed RT-qPCR on total RNA isolated from *T. versicolor* root tips following exposure to two HIFs (DMBQ and peonidin) and a non-HIF (juglone), using primers chosen for amplification of either the extended 5′UTR or the shorter 5′UTR of the *TvPirin* transcripts. The shorter 5′UTR amplicon displayed the same up-regulated expression pattern as the *TvPirin* ORF amplicon, whereas the prolonged 5′UTR amplicon did not show any significant up-regulation in response to either DMBQ or peonidin (Figure 1C), indicating that the extended 5′UTR does not authentically belong to the *TvPirin* transcript.

Collectively, our *in silico* and RT-qPCR results support that the shorter 5′UTR is the *bona-fide* 5′UTR of the full-length *TvPirin* cDNA. Consequently, the TvPirin protein contains 288 aa (Additional file 1B) and not 322 aa as previously reported [20,31] (Additional file 2B). The formerly published *TvPirin* cDNA sequence likely represents an artifact of 454 and Illumina sequence assembly. The artifactual nature of the *TvPirin* TrVeBC2_244713 clone is further supported by the fact that BLAST searches for *TvQR1* sequences in the *T. versicolor* transcriptome ( http://ppgp.huck.psu.edu/search.php, [31]) did not yield any *TvQR1* complete ORF, suggesting that caution should be applied when using the *T. versicolor* transcriptome assembled by 454 and Illumina sequencing methods to assign gene structures*.* Our findings reinforce that for *T. versicolor*, gene annotation for individual gene studies needs a careful and thorough molecular cloning approach coupled with verification by gene expression analysis until a completely and properly annotated genome is available.

### *TvQR1* and *TvPirin* exhibit distinctive natural allelic diversity

**Figure 2:**
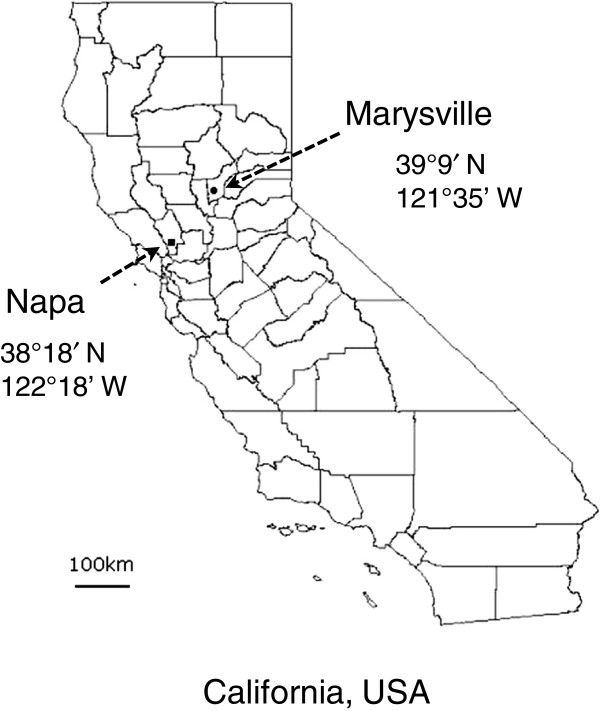
**Map showing the *T. versicolor *seed collection sites.**

The *TvQR1* cDNA (Accession AF304461), originally isolated from the same SSH library used to clone the full-length *TvPirin* cDNA [20,22,30], is a partial cDNA clone containing the complete ORF but lacking the 5′ and 3′UTRs. We cloned the full-length *TvQR1* cDNA using the same procedures carried out for *TvPirin* as described above, and recovered 9 independent full-length *TvQR1* cDNA clones representing 9 different *TvQR1* cDNA alleles (Additional file 4). The aforementioned 4 independently recovered full-length *TvPirin* cDNA clones also corresponded to 4 different *TvPirin* cDNA alleles (Additional file 1). In order to understand the patterns of nucleotide polymorphisms of these genes, we conducted a molecular population genetic analysis by using natural strains collected from a population in Northern Californian grasslands (Figure 2). We randomly selected 20 plants from the *in vitro* haustorium formation assays, of which 6 were responsive to DMBQ (designated as R1-R6), 8 responsive to peonidin (r1-r8), 3 non-responsive to DMBQ (N1-N3), and 3 non-responsive to peonidin (n1-n3) (see Methods). Genomic sequences from the start codon to the stop codon were obtained for each gene from the same plant by the Sanger sequencing method.

**Figure 3:**
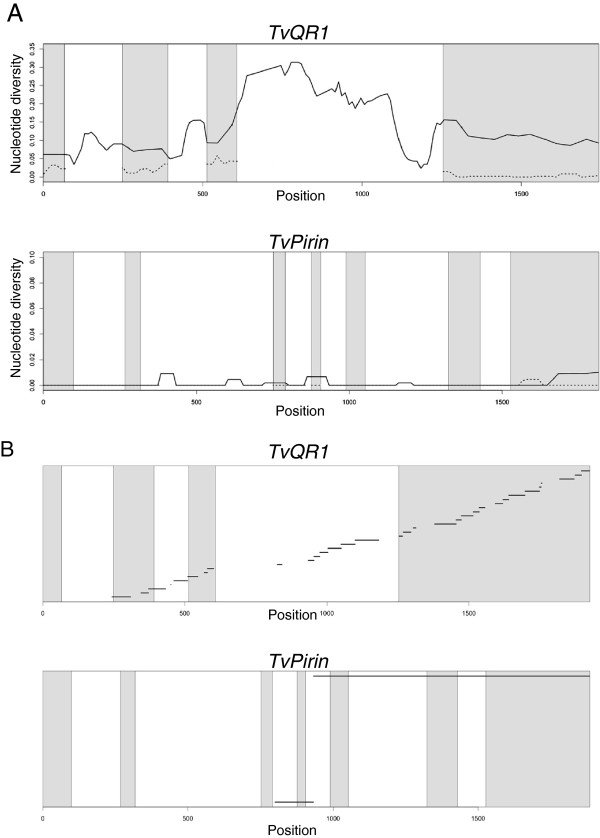
**Distinctive natural allelic polymorphisms of *TvQR1 *and *TvPirin*. (A)** Sliding window analysis of nucleotide diversity of *TvQR1* and *TvPirin*. Solid and dashed lines are silent (synonymous and intron) and non-synonymous nucleotide diversity, respectively. Shaded regions indicate exons. **(B)** Minimum estimates of recombination across *TvQR1* and *TvPirin*. Within each line, at least one recombination event was detected by the four-gamete test. Shaded regions indicate exons.

The complete alignments of all 40 alleles from these 20 plants showed sequences of 1962 and 2011 nucleotides long for *TvQR1* and *TvPirin*, respectively (Additional files 5 and 6). We found striking differences in nucleotide diversity between the two genes. While there were only 9 single nucleotide polymorphisms (SNPs) in *TvPirin* (Additional file 6, Table 2), *TvQR1* contained 350 segregating sites in addition to 31 insertions/deletions (indels) throughout the entire gene body, with indels present only in introns, and with the highest diversity in intron 3 (Additional file 5, Table 2, Figure 3A). This confers *TvQR1* nucleotide diversity two orders of magnitude higher than that of *TvPirin*. Likewise, the numbers of synonymous substitutions and non-synonymous changes in *TvQR1* are 55 and 27 times higher, respectively, than those of *TvPirin* (Table 2). Furthermore, four-gamete tests detected a higher level of recombination in *TvQR1* (Rm = 32) compared to *TvPirin* (Rm = 2) (Table 2, Figure 3B), which might have contributed to the generation of the extremely high number of haplotypes in *TvQR1*. In *TvQR1*, the highest number of recombination events was estimated in the last exon (Figure 3B). As all these individual plants came from one single population of a restricted geographical location (Figure 2), this high level of intra-populational variations of *TvQR1* is even more extraordinary.

**Table 2:** **Population genetic summary statistics of *TvQR1 *and *TvPirin***

	***TvQR1***	***TvPirin***
Number of nucleotide sites	1962	2011
Number of segregating sites (S)	350	9
Average number of pairwise differences *(*π)*	0.09306	0.00096
Watterson’s estimator of population mutation rate (θ_W_)	0.04966	0.00105
Minimum number of recombination (Rm)	32	2
Number of synonymous sites	239.02	198.19
Number of nonsynonymous sites	747.98	665.81
Number of synonymous and non-coding positions	909.02	1345.19
π_s_ (nucleotide diversity of synonymous sites)*	0.12568	0.00228
π_a_ (nucleotide diversity of nonsynonymous sites)*	0.01167	0.00043

*Jukes-Cantor corrected.

Next, we examined the diversity of *TvQR1* and *TvPirin* genes at the protein level by aligning the aa sequences deduced from the ORFs of their genomic and full-length cDNA clones. InterProScan runs from Swissprot for these two proteins identified the alcohol dehydrogenase (ADH) GrosE-like and the ADH_N_2 Rossmann domains in TvQR1 (Additional file 7), and the Pirin N- and C-terminal domains in TvPirin (Additional file 8). Although the crystallized structure of the human Pirin homolog revealed the β-barrel structure of the N- and C-terminal domains [32], no biochemical functions for either domain have been identified. By contrast, in redox enzymes such as ADH or quinone oxidoreductases, the ADH GrosE-like domain is the catalytic site with the oxidoreductase activity and substrate specificity [33,34], while the ADH_N_2 Rossmann domain is the NAD(P)-binding site where the one-electron addition/reduction reaction takes place [35]– [37].

As predicted from the higher number of non-synonymous substitutions described above, the TvQR1 protein exhibited more aa variations than the TvPirin protein (Additional files 7 and 8). A total of 13 aa changes were present in the 288-aa TvPirin protein with no preferential domain of high polymorphic level, and 12 of these variations belonged to rare alleles where the polymorphism occurred only once as singletons in the 34 deduced proteins (Additional file 8). On the other hand, only 13 of the 33 aa variation sites in the 329-aa TvQR1 protein sequence were singletons among the 29 deduced proteins (Additional file 7). The ADH GrosE-like domain of TvQR1 presented the largest cluster of aa polymorphisms. Its 14 changes within the 64-aa stretch (22%) are 3-fold higher than the 17 changes distributed over the remaining 266 aa residues of the protein (6.4%). Notably, the other domain of TvQR1, ADH_N_2 Rossmann, contained only 9 changes in the 130-aa stretch (7%), with 2 residues at positions 289 and 305 having 4 and 3 variants, respectively (Additional file 7). We further looked for aa changes in these two proteins that occurred exclusively in the non-responsive plants in order to identify potential mutations associated with the loss of haustorium formation phenotype. However, such changes were not found (Additional files 7 and 8), suggesting that loss-of-function mutations could reside in the promoter and/or other regulatory elements of the genes.

Collectively, these population genetic analyses revealed a remarkably higher level of molecular diversity, at both nucleotide and aa levels, of *TvQR1* compared to *TvPirin* in the *T. versicolor* natural populations from Northern California. It is noteworthy that the highest aa polymorphism in TvQR1 appears in the ADH GrosE-like domain, which determines substrate specificity. Thus, by analogy to the extremely divergent LRR domain of the plant resistance *R* genes involved in specific pathogen recognition [38]– [43], the ADH GrosE-like domain of TvQR1 may have been under diversifying selection that sustains its molecular labile state in order to enable rapid changes aimed at recognizing a large variety of quinone substrates from host root exudates. At least 19 species belonging to 14 different families in both monocot and dicot clades are natural hosts of *T. versicolor*, and multiple hosts are often parasitized simultaneously by *T. versicolor* in the fields [19]. Therefore, having the ability to recognize a multitude of host factors from such a wide range of hosts would confer this parasitic species an evolutionary advantage. We hypothesize that parasitic plants might have evolved a mechanism to exploit the abundant allelopathic chemicals in their environment and turn the phytotoxicity they initially encountered into a haustorium-inducing cue for their benefit. Maintenance of *TvQR1* molecular diversity could be a mechanism enabling this evolutionary process. High level of nucleotide diversity in synonymous sites as well as in non-synonymous sites of *TvQR1* may suggest that the long-term selection has been acting on this gene, as it is expected to elevate nucleotide diversity of closely linked sites while non-synonymous sites are more likely to be the direct target of selection. Those patterns are indeed observed in the plant R genes that are suggested to be under long-term balancing selection (e.g. [41,42]). TvPirin, on the other hand, as a transcription co-factor, probably interacts with other proteins with specific conformations for DNA binding, and thus would be subject to more evolutionary constraints in order to maintain specific protein-protein interactions, which might have led to the much lower molecular diversity observed here.

## Conclusions

Our molecular analysis has shown that, in the natural *T. versicolor* populations from Northern California, the only two genes known to date to be required for haustorium initiation, *TvQR1* and *TvPirin*, display highly contrasting patterns of molecular polymorphisms. Especially, the nucleotide diversity of *TvQR1* is 97-times higher than that of *TvPirin*, suggesting that these two genes would have been subjected to different selective pressures, possibly reflecting their distinct roles in phytochemical perception during host recognition. This difference is further manifest in their differential regulation by HIFs and non-HIFs. Since *TvQR1* responds to both HIFs and non-HIFs, it might not only function in haustorium development but also in the oxidative stress response. The non-toxic HIF peonidin provides a useful tool to dissect these two pathways in future search for additional parasitic plant genes involved in haustorium development. Differential responses of parasitic genes under peonidin, other toxic HIFs, and toxic non-HIFs bear the potential to separate genes only involved in the detoxification pathway from genes specific to the haustorium signaling pathway. Furthermore, although peonidin is a very effective HIF in *T. versicolor*, its ability to induce haustoria in other parasitic plant species has not yet been investigated. How peonidin functions as a HIF, e.g., with regard to its active form and receptor on the parasite, also awaits further studies. Both *TvQR1* and *TvPirin* genes have orthologs in non-parasitic plants. Thus, further phylogenic investigations of these genes in different plant taxa might elucidate the mechanism(s) by which parasitic plants have, during the evolutionary course from complete autotrophy to heterotrophy, co-opted a quinone oxidoreductase and a transcription co-factor from their existing genetic reservoir and adapted them to fulfill the new function of developing the organ that embodies plant parasitism: the haustorium.

## Methods

### Plant and chemical materials

*T. versicolor* seeds were collected and pooled from wild populations growing in the grasslands of Northern California, USA. For *T. versicolor* cDNA clones and Northern blot analysis, seeds were collected in Napa (Napa county) (Figure 2). For *in vitro* haustorium and root necrosis assays, qRT-PCR experiments, and population genetics study, seeds were collected in Marysville (Yuba county) (Figure 2). DMBQ and juglone were obtained from Pflatz & Bauer (Waterbury, CT, USA) and Alfa Aesar (Karlsruhe, Germany). Peonidin was obtained from Indofine Chemicals (Belle Mead, NJ, USA) and Santa Cruz Biotechnology, Inc. (Heidelberg, Germany).

### Haustorium induction and toxicity assays

*T. versicolor* seeds were surface-sterilized, germinated, grown on agar plates, and seedlings treated with various haustorial inducers (10 or 100 μM DMBQ, 30 μM or 1 mM peonidin) and non-inducers (50 μM juglone), treatment combinations (10 μM DMBQ + 30 μM juglone), or mock-treated with water or 0.1% EtOH (which is the peonidin solvent) as described previously [11,21]. Each plate contained 10–20 seedlings. For each treatment, haustorium formation and root necrosis were scored 24 h after treatments in 60–66 seedlings.

### Nucleic acid extraction

For Northern blot analysis, total RNA was isolated from root tips, remaining roots, and shoots of 100–200 *T. versicolor* plants treated with water, 0.1% ethanol, 20 μM DMBQ, 10 μM juglone, or 30 μM peonidin at 1, 3, and 5 h after treatment. For RT-qPCR, 20 μM DMBQ or 30 μM peonidin was applied twice to the same seedling plates at 2-day intervals and haustorium formation was scored 24 h after each time to identify responsive and non-responsive plants. Then the third treatment was applied, and 2 h later, root tips were collected from three batches of 20 each inducer-responsive plants representing three biological replicates. One application was performed for mock (water and 0.1% ethanol) or 10 μM juglone treatments to collect root tips for three biological replicates. Total RNA was extracted from root tips by the Trizol method (Invitrogen, Carlsbad, CA, USA). From the haustorium formation assays performed for the RT-qPCR experiments, 20 individual plants responsive or non-responsive to DMBQ or peonidin were randomly selected for genomic DNA extraction using CTAB and chloroform, followed by isopropanol precipitation and ethanol washes [44].

### Transcription assays

Northern blot hybridization was performed with 10 μg of total RNA from each sample as described elsewhere [22]. cDNA probes for the RNA blot were synthesized by PCR-amplifying the *TvQR1*, *TvPirin*, and *TvRuvB DNA helicase* partial cDNA clones from the SSH library [21], randomly labeled by nick-translation with CTP ^32^ as per the manufacturer’s protocol (Ambion, now Invitrogen, Carlsbad, CA, USA), and used at 10^7^ cpm/ml of hybridization buffer. The radioactive signals on the filters were captured on the Phosphor screen and images were scanned by the Storm PhosphorImager (GMI, Ramsey, MN, USA). The same filters were sequentially hybridized to different probes following the stripping of each previous probe and exposure to the Phosphor screen to check for residual signals. For RT-qPCR, two technical replicates were carried out with 1 μl of 1:20 diluted cDNAs reverse-transcribed from 2 μg each of three total RNA biological replicates in a 20 μl volume using SYBR Green I dye in an Applied Biosystem 7500 real-time PCR machine (Applied Biosystem). PCR parameters were as followed: 50°C/20 sec, 95°C/10 min, 40× (95°C/15 sec, 60°C/1 min). Melting curve analysis was done at the end of the PCR reactions to confirm single PCR product amplifications at 95°C/1 min, 60°C/30 sec, 95°C/1 min, 60°C/15 sec. Relative quantification of target transcripts were normalized to the reference transcript *TvRuvB DNA helicase*, which is constitutively expressed, and the relative expression levels of each transcript species under inducing and non-inducing conditions were compared to the control (water) treatment by the Δ ΔCt method [45].

### Gene cloning

5′ and 3′ RACE reactions of *TvQR1* and *TvPirin* were performed with total RNA isolated from DMBQ-responsive *T. versicolor* root tips using the Invitrogen’s RLM RACE kit per the company protocol. The amplified cDNAs were cloned into the pCR2.1TOPO vector (Invitrogen) and sequenced; the transcriptional start and stop sites of these sequences were identified and used to design new primers complementary to both ends of each cDNA clone for subsequent PCR amplification of the full-length cDNA clones from the same cDNA source used for RACE and from *T. versicolor* genomic DNA with high-fidelity polymerases *Pfu* or *Phusion Taq* (NEB). PCR parameters were typically as follows: 94°C/2 min, 30× (94°C/15 sec, 60°C/25 sec, 72°C/2.5 min), 72°C/5 min, with the 94°C steps being replaced with 98°C when *Phusion Taq* was used. The full-length cDNA PCR products were cloned into pCRII vector (Invitrogen) and sequenced. The genomic DNA PCR product from each individual plant was directly sequenced by the Sanger sequencing method and the sequence chromatograms visually inspected for sites where two nucleotide peaks of similar height were present which indicated heterozygosity. Sequences of both strands of heterozygotes were visually inspected to confirm the same two nucleotide peaks. The genomic DNA of the identified heterozygotes was subcloned into the pDrive vector, and the corresponding inserts isolated from 2–4 independent clones were subsequently sequenced to identify the SNPs in each allele.

### Sequence analyses

Contig assembly was done manually or by using the CLC Main Workbench 6.6.1 software (CLC bio) coupled with visual inspection for base call error and heterozygosity detection. BLASTN 2.2.13 and BLASTN 2.2.26+ [46] were used to search for *TvPirin* sequences in the *T. versicolor* genome sequence databases at http://ppgp.huck.psu.edu/search.php and http://www.plantsciences.ucdavis.edu/yoder/lab/Sequence_index.html. Multiple sequence alignments were performed with MultAlin ( http://multalin.toulouse.inra.fr/multalin/[47] or ClustalW2 ( http://www.ebi.ac.uk/Tools/msa/clustalw2/, [48,49]), and the multiple alignments presented in the BOXSHADE format ( http://www.ch.embnet.org/software/BOX_form.html, Kay Hofmann and Michael D. Baron). The protein PFAM domains were identified from Swissprot database using InterProScan ( http://www.ebi.ac.uk/Tools/pfa/iprscan/).

### Population genetic analysis

Sequence alignments were performed using ClustalX installed in MEGA5 [50]. Minor adjustments to optimize the alignments were made by eye. Various population genetic analyses were performed using DnaSP v5 [51]. Nucleotide diversity was corrected using the Jukes-Cantor method [52]. In the sliding window analysis, window length was 50 bp and step size was 10 bp.

### Primers (5′ to 3′)

*TvQR1* full-length cDNA: forward CTGGAATTCATTTTCAAGCTTCTCA, reverse CTTATGGCTCGACAACGATTTT.

*TvPirin* full-length cDNA: forward ATTCCTCATAAACATCAAATCCCCA, reverse TAGGTTAACAAAAGATCTCATATCAAAACA.

*TvQR1* RT-qPCR (164 nt amplicon): forward TGATAAAGTTGTTGCTATGCTTGGC, reverse GTTGGGTGAGGGCCATGTG.

*TvPirin* RT-qPCR (90 nt amplicon): forward TCCAAGGACAAAATGATTGAACCT, reverse CACTTTTACTTCAACCCCATCTTTTT.

*Bona-fide TvPirin* 5′UTR RT-qPCR (60 nt amplicon): forward ATTCCTCATAAACATCAAATCCCCAAT, reverse ATGATATAACGTCGAGAAGACGGGTTT.

*TrVeBC2*_*244713* 5′UTR RT-qPCR (102 nt amplicon): forward GAAATGCCACTATTACTCAGCTGGT, reverse TGGGGATTTGATGTTTATGAGGA.

*TvRuvB DNA helicase* RT-qPCR (133 nt amplicon): forward TAAACCGAGCTCTGGAGAACGAC, reverse ATGAGCAGACGGTCGAGAAAATC.

### Accession numbers

Sequence data from this article can be found in the GenBank data libraries under accession numbers XX000000.

## Abbreviations

T. versicolor: *Triphysaria versicolor*; HIF: Haustorium inducing factor; DMBQ: 2,6-dimethoxybenzoquinone; SNP: Single nucleotide polymorphism; Indel: Insertion/deletion; RT-qPCR: Reverse transcription - quantitative polymerase chain reaction.

## Competing interests

The authors declare that they have no competing interests.

## Authors’ contributions

QAN conceptualized and designed the study. QAN, HA, and TT carried out experiments, analyzed the data, and wrote the manuscript. UG provided the infrastructure, research materials and reagents, and analysis tools. All authors critically read and commented on the manuscript and approved the final version for submission.

## Authors’ information

QAN studied *Triphysaria versicolor* and *Triphysaria pussilla* during her PhD program at the Genetics Graduate Group of the University of California - Davis during the years of 2000–2003, focusing on cloning and functional characterization of *Triphysaria* candidate genes involved in haustorium development, and on the development of *Triphysaria* transformation systems for gene functional test by RNAi-mediated gene silencing. Since 2007, QAN is a postdoctoral fellow at the University of Zürich. HA established root exudates as haustorium inducers in *T. versicolor* and identified peonidin as a HIF in *T. versicolor* during her postdoctoral work from 1993 to 1997 at the University of California -Davis. TT studies the genetic basis of adaptation from 2010–2011 as a postdoctoral fellow at the University of Zürich and, since 2012, at the Gregor Mendel Institute in Vienna. In this study, he contributed to the analysis of the nucleotide polymorphism in *T. versicolor*. UG is heading the Department of Plant Developmental Genetics at the Institute of Plant Biology of the University of Zürich and studies developmental, ecological, and evolutionary aspects of plant reproduction.

## Supplementary Material

Additional file 1 **Identification of *TvPirin *full-length cDNA and open reading frame.** Click here for file

Additional file 2 ***TvPirin *open reading frame encodes a 288-amino acid long protein.** Click here for file

Additional file 3 Discontinuous identity between Accession JN606867 and clone TrVe61FuB1_8246. Click here for file

Additional file 4 **Multiple alignment of full-length *TvQR1 *cDNA clones.** Click here for file

Additional file 5 **Multiple sequence alignment of all genomic *TvQR1 *alleles.** Click here for file

Additional file 6 **Multiple sequence alignment of all genomic *TvPirin *alleles.** Click here for file

Additional file 7 TvQR1 protein domains and amino acid diversity. Click here for file

Additional file 8 TvPirin protein domains and amino acid diversity. Click here for file
