# Supplementary material for: The differentially regulated genes TvQR1 and TvPirin of the parasitic plant Triphysaria exhibit distinctive natural allelic diversity
Source: BMC Plant Biol. 2013 Feb 18;13:28. doi: 10.1186/1471-2229-13-28 (PMC3599707; doi:10.1186/1471-2229-13-28)
Supplement: Additional file 1 — Identification of TvPirin full-length cDNA and open reading frame. [file 1471-2229-13-28-S1.pdf]

Additional file 1. Identification of *TvPirin* full-length cDNA and open reading frame

A. Multiple alignment of 5'RACE, 3'RACE, and full-length cDNA clones of *TvPirin*

Five cDNA clones of the 5'RACE reactions (5'Pirin_#), 6 of the 3'RACE reactions (3'Pirin_#), and 4 of the full-length *TvPirin* (cTvPirin_#) are aligned to identify the open reading frame. Overlapping segments between the RACE clones and the full-length cDNA clones are in blue. The start codon is highlighted with **green bold** letters and the stop codon in **red bold** letters.

```
            1                                                          60
 5'Pirin_1  ----AAAATT CCTCATAAAC ATCAAATCCC CAATAATAAA AAACCCGTCT TCTCGACGTT
 5'Pirin_3  ----AAAATT CCTCATAAAC ATCAAATCCC CAATAATAAA AAACCCGTCT TCTCGACGTT
 5'Pirin_2  ----AAAATT CCTCATAAAC ATCAAATCCC CAATAATAAA AAACCCGTCT TCTCGACGTT
 5'Pirin_5  ----AAAATT CATCATAAAC ATCAAATCCC CAATAATAAA AAACCCGTCT TCTCGACGTT
 5'Pirin_4  AAAATTCATT CATCATAAAC ATCAAATCCC CAATAATAAA AAACCCGTCT TCTCGACGTT
 3'Pirin_1  ---------- ---------- ---------- ---------- ---------- ----------
 3'Pirin_4  ---------- ---------- ---------- ---------- ---------- ----------
 3'Pirin_3  ---------- ---------- ---------- ---------- ---------- ----------
 3'Pirin_2  ---------- ---------- ---------- ---------- ---------- ----------
 3'Pirin_5  ---------- ---------- ---------- ---------- ---------- ----------
 3'Pirin_6  ---------- ---------- ---------- ---------- ---------- ----------
cTvPirin_1  -------ATT CCTCATAAAC ATCAAATCCC CAATAATAAA AAACCCGTCT TCTCGACGTT
cTvPirin_2  -------ATT CCTCATAAAC ATCAAATCCC CAATAATAAA AAACCCGTCT TCTCGACGTT
cTvPirin_4  -------ATT CCTCATAAAC ATCAAATCCC CAATAATAAA AAACCCGTCT TCTCGACGTT
cTvPirin_3  -------ATT CCTCATAAAC ATCAAATCCC CAATAATAAA AAACCCGTCT TCTCGACGTT


            61                                                        120
 5'Pirin_1  ATATCATCAT GTCCAGTTTC CAACAGCCCA GACTAGTTGC CAAGAAGATC TTGGCCAAGT
 5'Pirin_3  ATATCATCAT GTCCAGTTTC CAACAGCCCA GGCTAGTTGC CAAGAAGATC TTGGCCAAGT
 5'Pirin_2  ATATCATCAT GTCCAGTTTC CAACAGCCCA GACTAGTTGC CAAGAAGATC TTGGCCAAGT
 5'Pirin_5  ATATCATCAT GTCCAGTTTC CAACAACCCA GACTAGTTGC CAAGAAGATC TTGGCCAAGT
 5'Pirin_4  ATATCATCAT GTCCAGTTTC CAACAGCCCA GACTAGTTGC CAAGAAGATC TTGGCCAAGT
 3'Pirin_1  ---------- ---------- ---------- ---------- ---------- ----------
 3'Pirin_4  ---------- ---------- ---------- ---------- ---------- ----------
 3'Pirin_3  ---------- ---------- ---------- ---------- ---------- ----------
 3'Pirin_2  ---------- ---------- ---------- ---------- ---------- ----------
 3'Pirin_5  ---------- ---------- ---------- ---------- ---------- ----------
 3'Pirin_6  ---------- ---------- ---------- ---------- ---------- ----------
cTvPirin_1  ATATCATCAT GTCCAGTTTC CAACAGCCCA GACTAGTTGC CAAGAAGATC TCGGCCAAGT
cTvPirin_2  ATATCATCAT GTCCAGTTTC CAACAGCCCA GACTAGTTGC CAAGAAGATC TTGGCCAAGT
cTvPirin_4  ATATCATCAT GTCCAGTTTC CAACAGCCCA GACTAGTTGC CAAGAAGATC TTGGCCAAGT
cTvPirin_3  ATATCATCAT GTCCAGTTTC CAACAGCCCA GACTAGTTGC CAAGAAGATC TTGGCCAAGT


            121                                                       180
 5'Pirin_1  CCCAGTCCGA GGGCGATGGT GCACTTGTAA GAAGAAGCAT TGGCAGGCCT GAGTTGAAGT
 5'Pirin_3  CCCAGTCCGA GGGCGATGGT GCACTTGTAA GAAGAAGCAT TGGCAGGCCT GAGTTGAAGT
 5'Pirin_2  CCCAGTCCGA GGGCGATGGT GCACTTGTAA GAAGAAGCAT TGGCAGGCCT GAGTTGAAGT
 5'Pirin_5  CCCAGTCCGA GGGCGATGGT GCACTTGTAA GAAGAAGCAT TGGCAGGCCT GAGTTGAAGT
 5'Pirin_4  CCCAGTCCGA GGGCGATGGT GCACTTGTAA GAAGAAGCAT TGGCAGGCCT GAGTTGAAGT
 3'Pirin_1  ---------- ---------- ---------- ---------- ---------- ----------
 3'Pirin_4  ---------- ---------- ---------- ---------- ---------- ----------
 3'Pirin_3  ---------- ---------- ---------- ---------- ---------- ----------
 3'Pirin_2  ---------- ---------- ---------- ---------- ---------- ----------
 3'Pirin_5  ---------- ---------- ---------- ---------- ---------- ----------
 3'Pirin_6  ---------- ---------- ---------- ---------- ---------- ----------
cTvPirin_1  CCCAGTCCGA GGGCGATGGT GCACTTGTAA GAAGAAGCAT TGGCAGGCCT GAGTTGAAGT
cTvPirin_2  CCCAGTCCGA GGGCGATGGT GCACTTGTAA GAAGAAGCAT TGGCAGGCCT GAGTTGAAGT
```

```
cTvPirin_4  CCCAGTCCGA GGGCGATGGT GCACTTGTAA GAAGAAGCAT TGGCAGGCCT GAGTTGAAGT
cTvPirin_3  CCCAGTCCGA GGGCGATGGT GCACTTGTAA GAAGAAGCAT TGGCAGGCCT GAGTTGAAGT

            181                                                      240
 5'Pirin_1  CCCTAGATCC ATTTCTCTTG CTAGATGAAT CTGCTGTTAC ACCTCCTGCT GGATTTCCTG
 5'Pirin_3  CCCTAGATCC ATTTCTCTTG CTAGATGAAT TTGCTGTTAC ACCTCCTGCT GGATTTCCTG
 5'Pirin_2  CCCTAGATCC ATTTCTCTTG CTAGATGAAT TTGCTGTTAC ACCTCCTGCT GGATTTCCTG
 5'Pirin_5  CCCTAGATCC ATTTCTCTTG CTAGATGAAT TTGCTGTTAC ACCTCCTGCT GGATTTCCTG
 5'Pirin_4  CCCTAGATCC ATTTCTCTTG CTAGATGAAT TTGCTGTTAC ACCTCCTGCT GGATTTCCTG
 3'Pirin_1  ---------- ---------- ---------- ---------- ---------- ----------
 3'Pirin_4  ---------- ---------- ---------- ---------- ---------- ----------
 3'Pirin_3  ---------- ---------- ---------- ---------- ---------- ----------
 3'Pirin_2  ---------- ---------- ---------- ---------- ---------- ----------
 3'Pirin_5  ---------- ---------- ---------- ---------- ---------- ----------
 3'Pirin_6  ---------- ---------- ---------- ---------- ---------- ----------
cTvPirin_1  CCCTAGATCC ATTTCTCTTG CTAGATGAAT TTGCTGTTAC ACCTCCTGCT GGATTTCCTG
cTvPirin_2  CCCTAGATCC ATTTCTCTTG CTAGATGAAT TTGCTGTTAC ACCTCCTGCT GGATTTCCTG
cTvPirin_4  CCCTAGATCC ATTTCTCTTG CTAGATGAAT TTGCTGTTAC ACCTCCTGCT GGATTTCCTG
cTvPirin_3  CCCTAGATCC ATTTCTCTTG CTAGATGAAT TTGCTGTTAC ACCTCCCGCT GGATTTCCTG

            241                                                      300
 5'Pirin_1  ACCATCCTCA CCGAGGCTTT GAGACTGTTA CATATATGTT GCAGGGAGGC GTCACACATC
 5'Pirin_3  ACCATCCTCA CCGAGGCTTT GAGACTGTTA CATATATGTT GCAGGGAGGC GTCACACATC
 5'Pirin_2  ACCATCCTCA CCGAGGCTTT GAGACTGTTA CATATATGTT GCAGGGAGGC GTCACACATC
 5'Pirin_5  ACCATCCTCA CCGAGGCTTT GAGACTGTTA CATATATGTT GCAGGGAGGC GTCACACATC
 5'Pirin_4  ACCATCCTCA CCGAGGCTTT GAGACTGTTA CATATATGTT GCAGGGAGGC GTCACACATC
 3'Pirin_1  ---------- ---------- ---------- ---------- ---------- ----------
 3'Pirin_4  ---------- ---------- ---------- ---------- ---------- ----------
 3'Pirin_3  ---------- ---------- ---------- ---------- ---------- ----------
 3'Pirin_2  ---------- ---------- ---------- ---------- ---------- ----------
 3'Pirin_5  ---------- ---------- ---------- ---------- ---------- ----------
 3'Pirin_6  ---------- ---------- ---------- ---------- ---------- ----------
cTvPirin_1  ACCATCCTCA CCGAGGCTTT GAGACTGTTA CATATATGTT GCAGGGAGGC GTCACACATC
cTvPirin_2  ACCATCCTCA CCGAGGCTTT GAGACTGTTA CATATATGTT GCAGGGAGGC GTCACACATC
cTvPirin_4  ACCATCCTCA CCGAGGCTTT GAGACTGTTA CATATATGTT GCAGGGAGGC GTCACACATC
cTvPirin_3  ACCATCCTCA CCGAGGCTTT GAGACTGTTA CATATATGTT GCAGGGAGGC GTCACACATC

            301                                                      360
 5'Pirin_1  AAGATTTTGC CGGTCATAAG GGCACCATAG GTGCTGGTGA CGTGCAGTGG ATGACTGCCG
 5'Pirin_3  AAGATTTTGC CGGTCATAAG GGCACCATAG GTGCTGGTGA CGTGCAGTGG ATGACTGCCG
 5'Pirin_2  AAGATTTTGC CGGTCATAAG GGCACCATAG GTGCTGGTGA TGTGCAGTGG ATGACTGCCG
 5'Pirin_5  AAGATTTTGC CGGTCATAAG GGCACCATAG GTGCTGGTGA CGTGCAGTGG ATGACTGCCG
 5'Pirin_4  AAGATTTTGC CGGTCATAAG GGCACCATAG GTGCTGGTGA CGTGCAGTGG ATGACTGCCG
 3'Pirin_1  ---------- ---------- ---------- ---------- ---------- ----------
 3'Pirin_4  ---------- ---------- ---------- ---------- ---------- ----------
 3'Pirin_3  ---------- ---------- ---------- ---------- ---------- ----------
 3'Pirin_2  ---------- ---------- ---------- ---------- ---------- ----------
 3'Pirin_5  ---------- ---------- ---------- ---------- ---------- ----------
 3'Pirin_6  ---------- ---------- ---------- ---------- ---------- ----------
cTvPirin_1  AAGATTTTGC CGGTCATAAG GGCACCATAG GTGCTGGTGA CGTGCAGTGG ATGACTGCCG
cTvPirin_2  AAGATTTTGC CGGTCATAAG GGCACCATAG GTGCTGGTGA CGTGCAGTGG ATGGCTGCCG
cTvPirin_4  AAGATTTTGC CGGTCATAAG GGCACCATAG GTGCTGGTGA CGTGCAGTGG ATGACTGCCG
cTvPirin_3  AAGATTTTGC CGGTCATAAG GGCACCATAG GTGCTGGTGA CGTGCAGTGG ATGACTGCCG

            361                                                      420
 5'Pirin_1  GCAGAGGTAT TGTTCACTCA GAAATGCCCG C--------- ---------- ----------
 5'Pirin_3  GCAGAGGTAT TGTTCACTCA GAAATGCCCG C--------- ---------- ----------
 5'Pirin_2  GCAGAGGTAT TGTTCACTCA GAAATGCCCG C--------- ---------- ----------
 5'Pirin_5  GCAGAGGTAT TGCTCACTCA GAAATGCCCG C--------- ---------- ----------
```

```
5'Pirin_4   GCAGAGGTAT TGTTCACTCA GAAATGCCCG C--------- ---------- ----------
3'Pirin_1   ---------- ---------- ---------- ---------- ---------- ----------
3'Pirin_4   ---------- ---------- ---------- ---------- ---------- ----------
3'Pirin_3   ---------- ---------- ---------- ---------- ---------- ----------
3'Pirin_2   ---------- ---------- ---------- ---------- ---------- ----------
3'Pirin_5   ---------- ---------- ---------- ---------- ---------- ----------
3'Pirin_6   ---------- ---------- ---------- ---------- ---------- ----------
cTvPirin_1  GCAGAGGTAT TGTTCACTCA GAAATGCCCG CCGGAGAAGG CACTCACACT GGTCTCCAAT
cTvPirin_2  GCAGAGGTAT TGTTCACTTA GAAATGCCCG CCGGAGAAGG CACTCACACT GGTCTCCAAT
cTvPirin_4  GCAGAGGTAT TGTTCACTCA GAAATGCCCG CCGGAGAAGG CACTCACACT GGTCTCCAAT
cTvPirin_3  GCAGAGGTAT TGTTCACTCA GAAATGCCCG CCGGAGAAGG CACTCACACT GGTCTCCAAT


            421                                                      480
5'Pirin_1   ---------- ---------- ---------- ---------- ---------- ----------
5'Pirin_3   ---------- ---------- ---------- ---------- ---------- ----------
5'Pirin_2   ---------- ---------- ---------- ---------- ---------- ----------
5'Pirin_5   ---------- ---------- ---------- ---------- ---------- ----------
5'Pirin_4   ---------- ---------- ---------- ---------- ---------- ----------
3'Pirin_1   ---------- ---------- ---------- ---------- ---------- ----------
3'Pirin_4   ---------- ---------- ---------- ---------- ---------- ----------
3'Pirin_3   ---------- ---------- ---------- ---------- ---------- ----------
3'Pirin_2   ---------- ---------- ---------- ---------- ---------- ----------
3'Pirin_5   ---------- ---------- ---------- ---------- ---------- ----------
3'Pirin_6   ---------- ---------- ---------- ---------- ---------- ----------
cTvPirin_1  TGTGGATCAA TCTCTCTTCC AAGGACAAAA TGATTGAACC TAGGTATCAA GAATTGCTGA
cTvPirin_2  TGTGGATCAA TCTCTCTTCC AAGGACAAAA TGATTGAACC TAGGTATCAA GAATTGCTGA
cTvPirin_4  TGTGGATCAA TCTCTCTTCC AAGGACAAAA TGATTGAACC TAGGTATCAA GAATTGCTGA
cTvPirin_3  TGTGGATCAA TCTCTCTTCC AAGGACAAAA TGATTGAACC TAGGCATCAA GAATTGCTGA


            481                                                      540
5'Pirin_1   ---------- ---------- ---------- ---------- ---------- ----------
5'Pirin_3   ---------- ---------- ---------- ---------- ---------- ----------
5'Pirin_2   ---------- ---------- ---------- ---------- ---------- ----------
5'Pirin_5   ---------- ---------- ---------- ---------- ---------- ----------
5'Pirin_4   ---------- ---------- ---------- ---------- ---------- ----------
3'Pirin_1   ---------- ---------- ---------- ---------- ---------- ----------
3'Pirin_4   ---------- ---------- ---------- ---------- ---------- ----------
3'Pirin_3   ---------- ---------- ---------- ---------- ---------- ----------
3'Pirin_2   ---------- ---------- ---------- ---------- ---------- ----------
3'Pirin_5   ---------- ---------- ---------- ---------- ---------- ----------
3'Pirin_6   ---------- ---------- ---------- ---------- ---------- ----------
cTvPirin_1  GTAATGACAT CCCCAAATCA CAAAAAGATG GGGTTGAAGT AAAAGTGATC GCAGGAGAAG
cTvPirin_2  GTAATGACAT CCCCAAAGCA CAAAAAGATG GGGTTGAAGT AAAAGTGATC GCAGGAGAAG
cTvPirin_4  GTAATGACAT CCCCAAAGCA CAAAAAGATG GGGTTGAAGT AAAAGTGGTC GCAGGAGAAG
cTvPirin_3  GTAATGACAT CCCCAAAGCA CAAAAAGATG GGGTTGAAGT AAAAGTGATC GCAGGAGAAG


            541                                                      600
5'Pirin_1   ---------- ---------- ---------- ---------- ---------- ----------
5'Pirin_3   ---------- ---------- ---------- ---------- ---------- ----------
5'Pirin_2   ---------- ---------- ---------- ---------- ---------- ----------
5'Pirin_5   ---------- ---------- ---------- ---------- ---------- ----------
5'Pirin_4   ---------- ---------- ---------- ---------- ---------- ----------
3'Pirin_1   ---------- ---------- ---------- ---------- ---------- ----------
3'Pirin_4   ---------- ---------- ---------- ---------- ---------- ----------
3'Pirin_3   ---------- ---------- ---------- ---------- ---------- ----------
3'Pirin_2   ---------- ---------- ---------- ---------- ---------- ----------
3'Pirin_5   ---------- ---------- ---------- ---------- ---------- ----------
3'Pirin_6   ---------- ---------- ---------- ---------- ---------- ----------
cTvPirin_1  CAATGGGTGT TCACTCACAA GTTTTCACAC GAACCCCAAC AATGTACGTC GATTTCACTC
cTvPirin_2  CAATGGGTGT TCACTCACAA GTTTTCACAC GAACCCCAAC AATGTACGTC GATTTCACTC
```

```
cTvPirin_4  CAATGGGTGT TCACTCACAA GTTTTCCCAC GAACCCCAAC AATGTACGTC GATTTCACTC
cTvPirin_3  CAATGGGTGT TCACCCACAA GTTTTCACAC GAACCCCAAC AATGTACGTC GATTTCACTC


            601                                                        660
 5'Pirin_1  ---------- ---------- ---------- ---------- ---------- ----------
 5'Pirin_3  ---------- ---------- ---------- ---------- ---------- ----------
 5'Pirin_2  ---------- ---------- ---------- ---------- ---------- ----------
 5'Pirin_5  ---------- ---------- ---------- ---------- ---------- ----------
 5'Pirin_4  ---------- ---------- ---------- ---------- ---------- ----------
 3'Pirin_1  ---------- ---------- ---------- ---------- ---------G TTTGTGTACA
 3'Pirin_4  ---------- ---------- ---------- ---------- ---------G TTTGTGTACA
 3'Pirin_3  ---------- ---------- ---------- ---------- ---------G TTTGTGTACA
 3'Pirin_2  ---------- ---------- ---------- ---------- ---------G TTTGTGTACA
 3'Pirin_5  ---------- ---------- ---------- ---------- ---------G TTTGTGTACA
 3'Pirin_6  ---------- ---------- ---------- ---------- ---------G TTTGTGTACA
cTvPirin_1  TAAAACCAAA TTCCGAGTAT CATCAAGGCA TTCCGGATTC TTGGAATGCG TTTGTGTACA
cTvPirin_2  TAAAACCAAA TTCCGAGTAT CATCAAGGCA TTCCGGATTC TTGGAATGCG TTTGTGTACA
cTvPirin_4  TAAAACCAAA TTCCGAGTAT CATCAAGGCA TTCCGGATTC TTGGAATGCG TTTGTGTACA
cTvPirin_3  TAAAACCAGA TTCCGAGTAT CATCAAGGCA TTCCGGATTC TTGGAATGCG TTTGTGTACA


            661                                                        720
 5'Pirin_1  ---------- ---------- ---------- ---------- ---------- ----------
 5'Pirin_3  ---------- ---------- ---------- ---------- ---------- ----------
 5'Pirin_2  ---------- ---------- ---------- ---------- ---------- ----------
 5'Pirin_5  ---------- ---------- ---------- ---------- ---------- ----------
 5'Pirin_4  ---------- ---------- ---------- ---------- ---------- ----------
 3'Pirin_1  TTATTGAAGG TGAAGGTGTA TTTGGCATCC CGAATTCGAA GCCTGCAGAA GCTCATCATA
 3'Pirin_4  TTATTGAAGG TGAAGGTGTA TTTGGCATCC CGAATTCGAA GCCTGCAGAA GCTCATCATA
 3'Pirin_3  TTATTGAAGG TGAAGGTGTA TTTGGCATCC CGAATTCGAA GCCTGCAGAA GCTCATCATA
 3'Pirin_2  TTATTGAAGG TGAAGGTGTA TTTGGCATCC CGAATTCGAA GCCTGCAGAA GTTCATCATA
 3'Pirin_5  TTATTGAAGG TGAAGGTGTA TTTGGCATCC CGAATTCGAA GCCTGCAGAA GCTCATCATA
 3'Pirin_6  TTATTGAAGG TGAAGGTGTA TTTGGCATCC CGAATTCGAA GCCTGCAGAA GCTCATCATA
cTvPirin_1  TTATTGAAGG TGAAGGTGTA TTTGGCATCC CGAATTCGAA GCCTGCAGAA GCTCATCATA
cTvPirin_2  TTATTGAAGG CGAGGGTGTA TTTGGCATCC CGAATTCGAA GCCTGCAGAA GCTCATCATA
cTvPirin_4  TTATTGAAGG TGAAGGTGTA TTTGGCATCC CGAATTCGAA GCCTGCAGAA GCTCATCATA
cTvPirin_3  TTATTGAAGG TGAAGGTGTA TTTGGCATCC CGAATTCGAA GCCTGCAGAA GCTCATCATA


            721                                                        780
 5'Pirin_1  ---------- ---------- ---------- ---------- ---------- ----------
 5'Pirin_3  ---------- ---------- ---------- ---------- ---------- ----------
 5'Pirin_2  ---------- ---------- ---------- ---------- ---------- ----------
 5'Pirin_5  ---------- ---------- ---------- ---------- ---------- ----------
 5'Pirin_4  ---------- ---------- ---------- ---------- ---------- ----------
 3'Pirin_1  TCTTGGTTTT GGGACCTGGA GAGGGGCTAA GTGTGTGGAA CAAGTTCACA GAACCATTGA
 3'Pirin_4  TCTTGGTTTT GGGACCTGGA GAGGGGCTAA GTGTGTGGAA CAAGTTCACA GAACCATTGA
 3'Pirin_3  TCTTGGTTTT GGGACCTGGA GAGGGGCTAA GTGTGTGGAA CAAGTTCACA GAACCATTGA
 3'Pirin_2  TCTTGGTTTT GGGACCTGGA GAGGGGCTAA GTGTGTGGAA CAAGTTCACA GAACCATTGA
 3'Pirin_5  TCTTGGTTTT GGGACCTGGA GAGGGGCTAA GTGTGTGGAA CAAGTTCACA GAACCATTGA
 3'Pirin_6  TCTTGGTTTT GGGACCTGGA GAGGGGCTAA GTGTGTGGAA CAAGTTCACA GAACCATTGA
cTvPirin_1  TCTTGGTTTT GGGACCTGGA GAGGGGCTAA GTGTGTGGAA CAAGTTCACA GAACCATTGA
cTvPirin_2  TCTTGGTTTT GGGACCTGGA GAGGGGCTAA GTGTGTGGAA CAAGTTCACA GAACCATTGA
cTvPirin_4  TCTTGGTTTT GGGACCTGGA GAGGGGCTAA GTGTGTGGAA CAAGTTCACA GAACCATTGA
cTvPirin_3  TCTTGGTTTT GGGACCTGGA GAGGGGCTAA GTGTGTGGAA CAAGTTCACA GAACCATTGA


            781                                                        840
 5'Pirin_1  ---------- ---------- ---------- ---------- ---------- ----------
 5'Pirin_3  ---------- ---------- ---------- ---------- ---------- ----------
 5'Pirin_2  ---------- ---------- ---------- ---------- ---------- ----------
 5'Pirin_5  ---------- ---------- ---------- ---------- ---------- ----------
```

```
 5’Pirin_4  ---------- ---------- ---------- ---------- ---------- ----------
 3’Pirin_1  GGTTTATTTT GGTAGGCGGG CAGCCACTGA ATGAGCCTGT GGTTCAGCAC GGTCCGTTTG
 3’Pirin_4  GGTTTATTTT GGTAGGCGGG CAGCCACTGA ATGAGCCTGT GGTTCAGCAC GGTCCGTTTG
 3’Pirin_3  GGTTTATTTT GGTAGGCGGA CAGCCACTGA ATGAGCCTGT GGTTCAGCAC GGTCCGTTTG
 3’Pirin_2  GGTTTATTTT GGTAGGCGGG CAGCCACTGA ATGAGCCTGT GGTTCAGCAC GGTCCGTTTG
 3’Pirin_5  GGTTTATTTT GGTAGGCGGG CAGCCACTGA ATGAGCCTGT GGTTCAGCAC GGTCCGTTTG
 3’Pirin_6  GGTTTATTTT GGTAGGCGGG CAGCCACTGA ATGAGCCTGT GGTTCAGCAC GGTCCGTTTG
cTvPirin_1  GGTTTATTTT GGTAGGCGGG CAGCCACTGA ATGAGCCTGT GGTTCAGCAC GGTCCGTTTG
cTvPirin_2  GGTTTATTTT GGTAGGCGGG CAGCCACTGA ATGAGCCTGT GGTTCAGCAC GGTCCGTTTG
cTvPirin_4  GGTTTATTTT GGTATGCGGG CAGCCACTGA ATGAGCCTGT GGTTCAGCAC GGTCCGTTTG
cTvPirin_3  GGTTTATTTT GGTAGGCGGG CAGCCACTGA ATGAGCCTGT GGTTCAGCAC GGTCCGTTTG


            841                                                      900
 5’Pirin_1  ---------- ---------- ---------- ---------- ---------- ----------
 5’Pirin_3  ---------- ---------- ---------- ---------- ---------- ----------
 5’Pirin_2  ---------- ---------- ---------- ---------- ---------- ----------
 5’Pirin_5  ---------- ---------- ---------- ---------- ---------- ----------
 5’Pirin_4  ---------- ---------- ---------- ---------- ---------- ----------
 3’Pirin_1  TGATGAATAC GCACGCTGAG ATTGACAAAA CAATGGAAGA CTATTACTAT GGCAAGAATG
 3’Pirin_4  TGATGAATAC GCACGCTGAG ATTGACAAAA CAATGGAAGA CTATTACTAT GGCAAGAATG
 3’Pirin_3  TGATGAATAC GCACGCTGAG ATTGGCAAAA CAATGGAAGA CTATTACTAT GGCAAGAATG
 3’Pirin_2  TGATGAATAC GCACGCTGAG ATTGACAAAA CAATGGAAGA CTATTACTAT GGCAAGAATG
 3’Pirin_5  TGATGAATAC GCACGCTGAG ATTGACAAAA CAATGGAAGA CTATTACTAT GGCAAGAATG
 3’Pirin_6  TGATGAATAC GCACGCTGAG ATTGACAAAA CAATGGAAGA CTATTACTAT GGCAAGAATG
cTvPirin_1  TGATGAATAC GCACGCTGAG ATTGACAAAA CAATGGAAGA CTATTACTAT GGCAAGAATG
cTvPirin_2  TGATGAATAC GCACGCTGAG ATTGACAAAA CAATGGAAGA CTATTACTAT GGCAAGAATG
cTvPirin_4  TGATGAATAC GCACGCTGAG ATTGACAAAA CAATGGAAGA CTATTACTAT GGCAAGAATG
cTvPirin_3  TGATGAATAC GCACGCTGAG ATTGACAAAA CAATGGAAGA CTATTACTAT GGCAAGAATG


            901                                                      960
 5’Pirin_1  ---------- ---------- ---------- ---------- ---------- ----------
 5’Pirin_3  ---------- ---------- ---------- ---------- ---------- ----------
 5’Pirin_2  ---------- ---------- ---------- ---------- ---------- ----------
 5’Pirin_5  ---------- ---------- ---------- ---------- ---------- ----------
 5’Pirin_4  ---------- ---------- ---------- ---------- ---------- ----------
 3’Pirin_1  GTTTCGAGCC GGCAAAAAAA TGGAAATCTA AATGAGCTAC ATATAAGCAA CTGCTTAGGG
 3’Pirin_4  GTTTCGAGCT GGCAAAAAAA TGGAAATCTA AATGAGCTAC ATATAAGCAA CTGCTTAGGG
 3’Pirin_3  GTTTCGAGCT GGCAAAAAAA TGGAAATCTA AATGAGCTAC ATATAAGCAA CTGCTTAGGG
 3’Pirin_2  GTTTCGAGCT GGCAAAAAAA TGGAAATCTA AATGAGCTAC ATATAAGCAA CTGCTTAGGG
 3’Pirin_5  GTTTCGAGCT GGCAAAAAAA TGGAAATCTA AATGAGCTAC ATATAAGCAA CTGCTTAGGG
 3’Pirin_6  GTTTCGAGCT GGCAAAAAAA TGGAAATCTA AATGAGCTAC ATATGAGCAA CTGCTTTGGG
cTvPirin_1  GTTTCGAGCT GGCAAAAAAA TGGAAATCTA AATGAGCTAC ATATAAGCAA CTGCTTAGGG
cTvPirin_2  GTTTCGAGCT GGCAAAAAAA TGGAAATCTA AATGAGCTAC ATATAAGCAA CTGCTTAGGG
cTvPirin_4  GTTTCGAGCT GGCAAAAAAA TGGAAATCTA AATGAGCTAC ATATAAGCAA CTGCTTAGGG
cTvPirin_3  GTTTCGAGCT GGCAAAAAAA TGGAAATCTA AATGAGCTAC ATATAAGCAA CTGCTTAGGG


            961                                                     1020
 5’Pirin_1  ---------- ---------- ---------- ---------- ---------- ----------
 5’Pirin_3  ---------- ---------- ---------- ---------- ---------- ----------
 5’Pirin_2  ---------- ---------- ---------- ---------- ---------- ----------
 5’Pirin_5  ---------- ---------- ---------- ---------- ---------- ----------
 5’Pirin_4  ---------- ---------- ---------- ---------- ---------- ----------
 3’Pirin_1  CCCCAAAAGA ATATTTAGAA CGTCCATGTT TTAAAAAGTC CTTATTTCCA TTTCATGTCT
 3’Pirin_4  CCCCAAAAGA ATATTTAGAA CGTCCATGTT TTAAAAAGTC CTTATTTCCA TTTCATGTCT
 3’Pirin_3  CCCCAAAAGA ATATTTAGAA CGTCCATGTT TTAAAAAGTC CTTATTTCCA TTTCATGTCT
 3’Pirin_2  CCCCAAAAGA ATATTTAGAA CGTCCATGTT TTAAAAAGTC CTTATTTCCA TTTCATGTCT
 3’Pirin_5  CCCCAAAAGA ATATTTAGAA CGTCCATGTT TTAAAAAGTC CTTATTTCCA TTTCATGTCT
 3’Pirin_6  CCCCAAAAGA ATATTTAGAA CGTCCATGTT TTAAAAAGTC CTTATTTCCA TTTCATGTCT
cTvPirin_1  CCCCAAAAGA ATATTTAGAA CGTCCATGTT TTAAAAAGTC CTTATTTCCA TTTCATGTCT
cTvPirin_2  CCCCAAAAGA ATATTTAGAA CGTCCATGTT TTAAAAAGTC CTTATTTCCA TTTCATGTCT
```

```
cTvPirin_4  CCCCAAAAGA ATATTTAGAA CGTCCATGTT TTAAAAAGTC CTTATTTCCA TTTCATGTCT
cTvPirin_3  CCCCAAAAGA ATATTTAGAA CGTCCATGTT TTAAAAAGTC CTTATTTCCA TTTCATGTCT

            1021                                                    1080
 5'Pirin_1  ---------- ---------- ---------- ---------- ---------- ----------
 5'Pirin_3  ---------- ---------- ---------- ---------- ---------- ----------
 5'Pirin_2  ---------- ---------- ---------- ---------- ---------- ----------
 5'Pirin_5  ---------- ---------- ---------- ---------- ---------- ----------
 5'Pirin_4  ---------- ---------- ---------- ---------- ---------- ----------
 3'Pirin_1  TTTTTTTTT- -GTTTAAAAA ATAAAGATAT AGGAAAATAA ATATGAAGTG AAATGTAATA
 3'Pirin_4  TTTTTTTTT- -GTTTAAAAA ATAAAGATAT AGGAAAATAA ATATGAAGTG AAATGTAATA
 3'Pirin_3  TTTTTTTTT- -GTTTAAAAA ATAAAGATAT AGGAAAATAA ATATGAAGTG AAATGTAATA
 3'Pirin_2  TTTTTTTT-- -GTTTAAAAA ATAAAGATAT AGGAAAATAA ATATGAAGTG AAATGTAATA
 3'Pirin_5  TTTTTTTT-- -GTTTAAAAA ATAAAGATAT AGGAAAATAA ATATGAAGTG AAATGTAATA
 3'Pirin_6  TTTTTTTT-- -GTTTAAAAA ATAAAGATAT AGGAAAATAA ATATGAAGTG AAATGTAATA
cTvPirin_1  TTTTTTT--- -GTTTAAAAA ATAAAGATAT AGGAAAATAA ATATGAAGTG AAATGTAATA
cTvPirin_2  TTTTTTTT-- -GTTTAAAAA ATAAAGATAT AGGAAAATAA ATATGAAGTG AAATGTAATA
cTvPirin_4  TTTTTTTT-- -GTTTAAAAA ATAAAGATAT AGGAAAATAA ATATGAAGTG AAATGTAATA
cTvPirin_3  TTTTTTTTTT TGTTTAAAAA ATAAAGATAT AGGAAAATAA ATATGAAGTG AAATGTAATA

            1081                                                    1140
 5'Pirin_1  ---------- ---------- ---------- ---------- ---------- ----------
 5'Pirin_3  ---------- ---------- ---------- ---------- ---------- ----------
 5'Pirin_2  ---------- ---------- ---------- ---------- ---------- ----------
 5'Pirin_5  ---------- ---------- ---------- ---------- ---------- ----------
 5'Pirin_4  ---------- ---------- ---------- ---------- ---------- ----------
 3'Pirin_1  CATCTCGTAT TGTGATTTGT GCCATTTATT TTTATTGGAA AGCTTTTGGT TTCGTTAAAA
 3'Pirin_4  CATCTCGTAT TGTGATTTGT GCCATTTATT TTTATTGGAA AGCTTTTGGT TTCGATAAAA
 3'Pirin_3  CATCTCGTAT TGTGATTTGT GCCATTTATT TTTATTGGAA AGCTTTTGGT TTCGTTAAAA
 3'Pirin_2  CATCTCGTAT TGTGATTTGT GCCATTTATT TTTATTGGAA AGCTTTTGGT TTCGTTAAAA
 3'Pirin_5  CATCTCGTAT TGTGATTTGT GCCATTTATT TTTATTGGAA AGCTTTTGGT TTCGTTAAAA
 3'Pirin_6  CATCTCGTAT TGTGATTTGT GCCATTTATT TTTATTGGAA AGCTTTTGGT TTCGGTAAAA
cTvPirin_1  CATCTCGTAT TGTGATTTGT GCCATTTATT TTTATTGGAA AGCTTTTGGT TTCGTT----
cTvPirin_2  CATCTCGTAT TGTGATTTGT GCCATTTATT TTTATTGGAA AGCTTTTGGT TTCGTT----
cTvPirin_4  CATCTCGTAT TGTGATTTGT GCCATTTATT TTTATTGGAA AGCTTTTGGT TTCGTT----
cTvPirin_3  CATCTCGTAT TGTGATTTGT GCCATTTATT TTTATTGGAA AGCTTTTGGT TTCGTT----

            1141
 5'Pirin_1  --------
 5'Pirin_3  --------
 5'Pirin_2  --------
 5'Pirin_5  --------
 5'Pirin_4  --------
 3'Pirin_1  AAAAAAA-
 3'Pirin_4  AAAAAAAA
 3'Pirin_3  AAAAAAA-
 3'Pirin_2  AAAAAAAA
 3'Pirin_5  AAAAAAAA
 3'Pirin_6  AAAAAAA-
cTvPirin_1  --------
cTvPirin_2  --------
cTvPirin_4  --------
cTvPirin_3  --------
```

B. TvPirin deduced protein sequence from the full-length cTvPirin_1 clone

```
attcctcataaacatcaaatccccaataataaaaaacccgtcttctcgacgttatatcatc

atgtccagtttccaacagcccagactagttgccaagaagatctcggccaagtcccagtcc
 M  S  S  F  Q  Q  P  R  L  V  A  K  K  I  S  A  K  S  Q  S
gagggcgatggtgcacttgtaagaagaagcattggcaggcctgagttgaagtccctagat
 E  G  D  G  A  L  V  R  R  S  I  G  R  P  E  L  K  S  L  D
ccatttctcttgctagatgaatttgctgttacacctcctgctggatttcctgaccatcct
 P  F  L  L  L  D  E  F  A  V  T  P  P  A  G  F  P  D  H  P
caccgaggctttgagactgttacatatatgttgcagggaggcgtcacacatcaagatttt
 H  R  G  F  E  T  V  T  Y  M  L  Q  G  G  V  T  H  Q  D  F
gccggtcataagggcaccataggtgctggtgacgtgcagtggatgactgccggcagaggt
 A  G  H  K  G  T  I  G  A  G  D  V  Q  W  M  T  A  G  R  G
attgttcactcagaaatgcccgccggagaaggcactcacactggtctccaattgtggatc
 I  V  H  S  E  M  P  A  G  E  G  T  H  T  G  L  Q  L  W  I
aatctctcttccaaggacaaaatgattgaacctaggtatcaagaattgctgagtaatgac
 N  L  S  S  K  D  K  M  I  E  P  R  Y  Q  E  L  L  S  N  D
atccccaaatcacaaaaagatggggttgaagtaaaagtgatcgcaggagaagcaatgggt
 I  P  K  S  Q  K  D  G  V  E  V  K  V  I  A  G  E  A  M  G
gttcactcacaagttttcacacgaaccccaacaatgtacgtcgatttcactctaaaacca
 V  H  S  Q  V  F  T  R  T  P  T  M  Y  V  D  F  T  L  K  P
aattccgagtatcatcaaggcattccggattcttggaatgcgtttgtgtacattattgaa
 N  S  E  Y  H  Q  G  I  P  D  S  W  N  A  F  V  Y  I  I  E
ggtgaaggtgtatttggcatcccgaattcgaagcctgcagaagctcatcatatcttggtt
 G  E  G  V  F  G  I  P  N  S  K  P  A  E  A  H  H  I  L  V
ttgggacctggagagggctaagtgtgtggaacaagttcacagaaccattgaggtttatt
 L  G  P  G  E  G  L  S  V  W  N  K  F  T  E  P  L  R  F  I
ttggtaggcgggcagccactgaatgagcctgtggttcagcacggtccgtttgtgatgaat
 L  V  G  G  Q  P  L  N  E  P  V  V  Q  H  G  P  F  V  M  N
acgcacgctgagattgacaaaacaatggaagactattactatggcaagaatggtttcgag
 T  H  A  E  I  D  K  T  M  E  D  Y  Y  Y  G  K  N  G  F  E
ctggcaaaaaaatggaaatctaaatgagctacatataagcaactgcttagggccccaaaa
 L  A  K  K  W  K  S  K  -
gaatatttagaacgtccatgttttaaaaagtccttatttccatttcatgtctttttttg

tttaaaaaataaagatataggaaaataaatatgaagtgaaatgtaatacatctcgtattg

tgatttgtgccatttatttttattggaaagcttttggtttcgtt
```
