# Supplementary material for: The differentially regulated genes TvQR1 and TvPirin of the parasitic plant Triphysaria exhibit distinctive natural allelic diversity
Source: BMC Plant Biol. 2013 Feb 18;13:28. doi: 10.1186/1471-2229-13-28 (PMC3599707; doi:10.1186/1471-2229-13-28)

Additional file 2. *TvPirin* gene structure

A. Multiple alignments of *TvPirin* cDNA clones and EST assemblies

The sequences are 5'RACE products (5'Pirin_#), full-length *TvPirin* cDNAs (cTvPirin_#), *TvPirin* ESTs from SSH libraries at http://www.plantsciences.ucdavis.edu/yoder/lab/Sequence_index.html (Contig492, Contig5118), and *Triphysaria versicolor* contig assemblies from Wickett *et al* (2011) at http://ppgp.huck.psu.edu/search.php (TrVeBC1_14677, TrVeBC2_244713, TrVe61FuB1_8246, TrVe61GuB1_71237, TrVe62GuB1_70666, TrVe63GuB1_32652, TrVe3GB1_60414, TrVeGnuB1_1027). Assemblies in reverse complementation orientation are *italicized*. The accession JN606867 is highlighted in gray as part of the TrVeBC2_244713 clone. The part of the *TrVe61FuB1_8246* clone identical to the 3' end of TrVeBC2_244713 clone if aligned in the same orientation (see Additional file 3) is highlighted in yellow. The full-length *TvPirin* cDNA sequences are colored blue with SNPs in red (common alleles) or black (rare alleles). The bona-fide start codon is depicted with **green bold** letters and the stop codon in **purple bold** letters. The artificial start codon is depicted in **black bold** letters. Dashes represent missing nucleotides.

```
              1                                                              60
 5'Pirin_1    ---------- ---------- ---------- ---------- ---------- ----------
 5'Pirin_3    ---------- ---------- ---------- ---------- ---------- ----------
 5'Pirin_2    ---------- ---------- ---------- ---------- ---------- ----------
 5'Pirin_4    ---------- ---------- ---------- ---------- ---------- ----------
 5'Pirin_5    ---------- ---------- ---------- ---------- ---------- ----------
cTvPirin_1    ---------- ---------- ---------- ---------- ---------- ----------
cTvPirin_2    ---------- ---------- ---------- ---------- ---------- ----------
cTvPirin_4    ---------- ---------- ---------- ---------- ---------- ----------
TrVeBC2_24    ---------- ---------- ---------- ---------- ---------- ----------
TrVeGnuB1_    ---------- ---------- ---------- ---------- ---------- ----------
cTvPirin_3    ---------- ---------- ---------- ---------- ---------- ----------
TrVe63GuB1    ---------- ---------- ---------- ---------- ---------- ----------
TrVe62GuB1    ---------- ---------- ---------- ---------- ---------- ----------
TrVe61GuB1    ---------- ---------- ---------- ---------- ---------- ----------
TrVe3GB1_6    ---------- ---------- ---------- ---------- ---------- ----------
Contig5118    ---------- ---------- ---------- ---------- ---------- ----------
TrVeBC1_14    ---------- ---------- ---------- ---------- ---------- ----------
TrVe61FuB1    TCATGATCAT GATCACAAGG ATGAGACTAC TTCTCATTGA TTATCATCGA TCCATTCTTC
Contig492     ---------- ---------- ---------- ---------- ---------- ----------


              61                                                            120
 5'Pirin_1    ---------- ---------- ---------- ---------- ---------- ----------
 5'Pirin_3    ---------- ---------- ---------- ---------- ---------- ----------
 5'Pirin_2    ---------- ---------- ---------- ---------- ---------- ----------
 5'Pirin_4    ---------- ---------- ---------- ---------- ---------- ----------
 5'Pirin_5    ---------- ---------- ---------- ---------- ---------- ----------
cTvPirin_1    ---------- ---------- ---------- ---------- ---------- ----------
cTvPirin_2    ---------- ---------- ---------- ---------- ---------- ----------
cTvPirin_4    ---------- ---------- ---------- ---------- ---------- ----------
TrVeBC2_24    ---------- ---------- ---------- ---------- ---------- ----------
TrVeGnuB1_    ---------- ---------- ---------- ---------- ---------- ----------
cTvPirin_3    ---------- ---------- ---------- ---------- ---------- ----------
TrVe63GuB1    ---------- ---------- ---------- ---------- ---------- ----------
TrVe62GuB1    ---------- ---------- ---------- ---------- ---------- ----------
TrVe61GuB1    ---------- ---------- ---------- ---------- ---------- ----------
TrVe3GB1_6    ---------- ---------- ---------- ---------- ---------- ----------
Contig5118    ---------- ---------- ---------- ---------- ---------- ----------
TrVeBC1_14    ---------- ---------- ---------- ---------- ---------- ----------
TrVe61FuB1    TCTTTTGGGG CCCTAAGCAG TTGCTTATAT GTAGCTCATT TAGATTTCCA TTTTTTTGCC
Contig492     ---------- ---------- ---------- ---------- ---------- ----------
```

```
              121                                                          180
  5'Pirin_1   ---------- ---------- ---------- ---------- ---------- ----------
  5'Pirin_3   ---------- ---------- ---------- ---------- ---------- ----------
  5'Pirin_2   ---------- ---------- ---------- ---------- ---------- ----------
  5'Pirin_4   ---------- ---------- ---------- ---------- ---------- ----------
  5'Pirin_5   ---------- ---------- ---------- ---------- ---------- ----------
cTvPirin_1    ---------- ---------- ---------- ---------- ---------- ----------
cTvPirin_2    ---------- ---------- ---------- ---------- ---------- ----------
cTvPirin_4    ---------- ---------- ---------- ---------- ---------- ----------
TrVeBC2_24    ---------- ---------- -------AAA TATTTCAATT TATTAGAAAT GCCAC-----
TrVeGnuB1_    ---------- ---------- -------AAA TATTTCAATT TATTAGAAAT GCCAC-----
cTvPirin_3    ---------- ---------- ---------- ---------- ---------- ----------
TrVe63GuB1    ---------- ---------- ---------- ---------- ---------- ----C-----
TrVe62GuB1    ---------- ---------- ---------- ---------- ---------- ----------
TrVe61GuB1    ---------- ---------- ---------- ---------- ---------- ----------
TrVe3GB1_6    ---------- ---------- ---------- ---------- ---------- ----------
Contig5118    ---------- ---------- ---------- ---------- ---------- ----------
TrVeBC1_14    ---------- ---------- ---------- ---------- ---------- ----------
TrVe61FuB1    AGCTCGAAAC CATTCTTGCC ATAGTAATAG TCTTCCATTG TTTTGTCAAT CTCAGCGTGC
Contig492     ---------- ---------- ---------- ---------- ---------- ----------

              181                                                          240
  5'Pirin_1   ---------- ---------- ---------- ---------- ---------- ----------
  5'Pirin_3   ---------- ---------- ---------- ---------- ---------- ----------
  5'Pirin_2   ---------- ---------- ---------- ---------- ---------- ----------
  5'Pirin_4   ---------- ---------- ---------- ---------- ---------- ----------
  5'Pirin_5   ---------- ---------- ---------- ---------- ---------- ----------
cTvPirin_1    ---------- ---------- ---------- ---------- ---------- ----------
cTvPirin_2    ---------- ---------- ---------- ---------- ---------- ----------
cTvPirin_4    ---------- ---------- ---------- ---------- ---------- ----------
TrVeBC2_24    -TATTACTC- -AGCTGGTTT GTAGACGGGC GGGCGCCTAT ATAAATGATC TCTGTATCTA
TrVeGnuB1_    -TATTACTC- -AGCTGGTTT GTAGACGGGC GGGCGCCTAT ATAAATGATC TCTGTATCTA
cTvPirin_3    ---------- ---------- ---------- ---------- ---------- ----------
TrVe63GuB1    -TATTACTCT AAGCTGGTTT GTAGACGGGC GGGCGCCTAT ATAAATGATC TCTGTATCTA
TrVe62GuB1    ---------- ---------- ---------- ---------- ---------- ----------
TrVe61GuB1    ---------- ---------- -----CGGGC GGGCGCCTAT ATAAATGATC TCTGTATCTA
TrVe3GB1_6    ---------- ---------- ---------- ---------- ---------- ----------
Contig5118    ---------- ---------- ---------- ---------- ---------- ----------
TrVeBC1_14    ---------- ---------- ----ACGGGC GGGCGCCTAT ATAAATGATC TCTGTATCTA
TrVe61FuB1    GTATTCATCA CAAACGGACC GT-GACGGGC GGGCGCCTAT ATAAATGATC TCTGTATCTA
Contig492     ---------- ---------- ---------- ---------- ---------- ----------

              241                                                          300
  5'Pirin_1   -------AAA ATTCCTCATA AACATCAAAT CCCCAATAAT AAAAAACCCG TCTTCTCGAC
  5'Pirin_3   -------AAA ATTCCTCATA AACATCAAAT CCCCAATAAT AAAAAACCCG TCTTCTCGAC
  5'Pirin_2   -------AAA ATTCCTCATA AACATCAAAT CCCCAATAAT AAAAAACCCG TCTTCTCGAC
  5'Pirin_4   ---AAAATTC ATTCATCATA AACATCAAAT CCCCAATAAT AAAAAACCCG TCTTCTCGAC
  5'Pirin_5   -------AAA ATTCATCATA AACATCAAAT CCCCAATAAT AAAAAACCCG TCTTCTCGAC
cTvPirin_1    ---------- ATTCCTCATA AACATCAAAT CCCCAATAAT AAAAAACCCG TCTTCTCGAC
cTvPirin_2    ---------- ATTCCTCATA AACATCAAAT CCCCAATAAT AAAAAACCCG TCTTCTCGAC
cTvPirin_4    ---------- ATTCCTCATA AACATCAAAT CCCCAATAAT AAAAAACCCG TCTTCTCGAC
TrVeBC2_24    CTAAACTTTC ATTCCTCATA AACATCAAAT CCCCAATAAT AAAAAACCCG TCTTCTCGAC
TrVeGnuB1_    CTAAACTTTC ATTCCTCATA AACATCAAAT CCCCAATAAT AAAAAACCCG TCTTCTCGAC
cTvPirin_3    ---------- ATTCCTCATA AACATCAAAT CCCCAATAAT AAAAAACCCG TCTTCTCGAC
TrVe63GuB1    CTAAACTTTC ATTCATCATA AACATCAAAT CCCCAATAAT AAAAAACCCG TCTTCTCGAC
TrVe62GuB1    ---------- ---------- -----CAAAT CCCCAATAAT AAAAAACCCG TCTTCTCGAC
TrVe61GuB1    CTAAACTTTC ATTCATCATA AACATCAAAT CCCCAATAAT AAAAAACCCG TCTTCTCGAC
TrVe3GB1_6    ---------- ---------- ---------- CCCCAATAAT AAAAAACCCG TCTTCTCGAC
Contig5118    ---------- ---------- -----CAAAT CCCCAATAAT AAAAAACCCG TCTTCTCGAC
TrVeBC1_14    CTAAACTTTC ATTCCTCATA AACATCAAAT CCCCAATAAT AAAAAACCCG TCTTCTCGAC
TrVe61FuB1    CTAAACTTTC ATTCCTCATA AACATCAAAT CCCMAATAAT AAAAAACCCG TCTTCTCGAC
Contig492     ---------- ---------- ---------- ---------- ---------- ----------
```

```
             301                                                           360
 5'Pirin_1   GTTATATCAT CATGTCCAGT TTCCAACAGC CCAGACTAGT TGCCAAGAAG ATCTTGGCCA
 5'Pirin_3   GTTATATCAT CATGTCCAGT TTCCAACAGC CCAGGCTAGT TGCCAAGAAG ATCTTGGCCA
 5'Pirin_2   GTTATATCAT CATGTCCAGT TTCCAACAGC CCAGACTAGT TGCCAAGAAG ATCTTGGCCA
 5'Pirin_4   GTTATATCAT CATGTCCAGT TTCCAACAGC CCAGACTAGT TGCCAAGAAG ATCTTGGCCA
 5'Pirin_5   GTTATATCAT CATGTCCAGT TTCCAACAAC CCAGACTAGT TGCCAAGAAG ATCTTGGCCA
cTvPirin_1   GTTATATCAT CATGTCCAGT TTCCAACAGC CCAGACTAGT TGCCAAGAAG ATCTCGGCCA
cTvPirin_2   GTTATATCAT CATGTCCAGT TTCCAACAGC CCAGACTAGT TGCCAAGAAG ATCTTGGCCA
cTvPirin_4   GTTATATCAT CATGTCCAGT TTCCAACAGC CCAGACTAGT TGCCAAGAAG ATCTTGGCCA
TrVeBC2_24   GTTATATCAT CATGTCCAGT TTCCAACAGC CCAGACTAGT TGCCAAGAAG ATCTTGGCCA
TrVeGnuB1_   GTTATATCAT CATGTCCAGT TTCCAACAGC CCAGACTAGT TGCCAAGAAG ATCTTGGCCA
cTvPirin_3   GTTATATCAT CATGTCCAGT TTCCAACAGC CCAGACTAGT TGCCAAGAAG ATCTTGGCCA
TrVe63GuB1   GTTATATCAT CATGTCCAGT TTCCAACAGC CCAGACTAGT TGCCAAGAAG ATCTTGGCCA
TrVe62GuB1   GTTATATCAT CATGTCCAGT TTCCAACAGC CCAGACTAGT TGCCAAGAAG ATCTTGGCCA
TrVe61GuB1   GTTATATCAT CATGTCCAGT TTCCAACAGC CCAGACTAGT TGCCAAGAAG ATCTTGGCCA
TrVe3GB1_6   GTTATATCAT CATGTCCAGT TTCCAACAGC CCAGACTAGT TGCCAAGAAG ATCTTGGCCA
Contig5118   GTTATATCAT CATGTCCAGT TTCCAACAGC CCAGACTAGT TGCCAAGAAG ATCTTGGCCA
TrVeBC1_14   GTTATATCAT CATGTCCAGT TTCCAACAGC CCAGACTAGT TGCCAAGAAG ATCTTGGCCA
TrVe61FuB1   GTTATATCAT CATGTCCAGT TTCCAACAGC CCAGACTAGT TGCCAAGAAG ATCTTGGCCA
Contig492    ---------- ---------- ---------- ---------- ---------- ----------

             361                                                           420
 5'Pirin_1   AGTCCCAGTC CGAGGGCGAT GGTGCACTTG TAAGAAGAAG CATTGGCAGG CCTGAGTTGA
 5'Pirin_3   AGTCCCAGTC CGAGGGCGAT GGTGCACTTG TAAGAAGAAG CATTGGCAGG CCTGAGTTGA
 5'Pirin_2   AGTCCCAGTC CGAGGGCGAT GGTGCACTTG TAAGAAGAAG CATTGGCAGG CCTGAGTTGA
 5'Pirin_4   AGTCCCAGTC CGAGGGCGAT GGTGCACTTG TAAGAAGAAG CATTGGCAGG CCTGAGTTGA
 5'Pirin_5   AGTCCCAGTC CGAGGGCGAT GGTGCACTTG TAAGAAGAAG CATTGGCAGG CCTGAGTTGA
cTvPirin_1   AGTCCCAGTC CGAGGGCGAT GGTGCACTTG TAAGAAGAAG CATTGGCAGG CCTGAGTTGA
cTvPirin_2   AGTCCCAGTC CGAGGGCGAT GGTGCACTTG TAAGAAGAAG CATTGGCAGG CCTGAGTTGA
cTvPirin_4   AGTCCCAGTC CGAGGGCGAT GGTGCACTTG TAAGAAGAAG CATTGGCAGG CCTGAGTTGA
TrVeBC2_24   AGTCCCAGTC CGAGGGCGAT GGTGCACTTG TAAGAAGAAG CATTGGCAGG CCTGAGTTGA
TrVeGnuB1_   AGTCCCAGTC CGAGGGCGAT GGTGCACTTG TAAGAAGAAG CATTGGCAGG CCTGAGTTGA
cTvPirin_3   AGTCCCAGTC CGAGGGCGAT GGTGCACTTG TAAGAAGAAG CATTGGCAGG CCTGAGTTGA
TrVe63GuB1   AGTCCCAGTC CGAGGGCGAT GGTGCACTTG TAAGAAGAAG CATTGGCAGG CCTGAGTTGA
TrVe62GuB1   AGTCCCAGTC CGAGGGCGAT GGTGCACTTG TAAGAAGAAG CATTGGCAGG CCTGAGTTGA
TrVe61GuB1   AGTCCCAGTC CGAGGGCGAT GGTGCACTTG TAAGAAGAAG CATTGGCAGG CCTGAGTTGA
TrVe3GB1_6   AGTCCCAGTC CGAGGGCGAT GGTGCACTTG TAAGAAGAAG CATTGGCAGG CCTGAGTTGA
Contig5118   AGTCCCAGTC CGAGGGCGAT GGTGCACTTG TAAGAAGAAG CATTGGCAGG CCTGAGTTGA
TrVeBC1_14   AGTCCCAGTC CGAGGGCGAT GGTGCACTTG TAAGAAGAAG CATTGGCAGG CCTGAGTTGA
TrVe61FuB1   AGTCCCAGTC CGAGGGCGAT GGTGCACTTG TAAGAAGAAG CATTGGCAGG CCTGAGTTGA
Contig492    ---------- ---------- ---------- ---------- ---------- ----------

             421                                                           480
 5'Pirin_1   AGTCCCTAGA TCCATTTCTC TTGCTAGATG AATCTGCTGT TACACCTCCT GCTGGATTTC
 5'Pirin_3   AGTCCCTAGA TCCATTTCTC TTGCTAGATG AATTTGCTGT TACACCTCCT GCTGGATTTC
 5'Pirin_2   AGTCCCTAGA TCCATTTCTC TTGCTAGATG AATTTGCTGT TACACCTCCT GCTGGATTTC
 5'Pirin_4   AGTCCCTAGA TCCATTTCTC TTGCTAGATG AATTTGCTGT TACACCTCCT GCTGGATTTC
 5'Pirin_5   AGTCCCTAGA TCCATTTCTC TTGCTAGATG AATTTGCTGT TACACCTCCT GCTGGATTTC
cTvPirin_1   AGTCCCTAGA TCCATTTCTC TTGCTAGATG AATTTGCTGT TACACCTCCT GCTGGATTTC
cTvPirin_2   AGTCCCTAGA TCCATTTCTC TTGCTAGATG AATTTGCTGT TACACCTCCT GCTGGATTTC
cTvPirin_4   AGTCCCTAGA TCCATTTCTC TTGCTAGATG AATTTGCTGT TACACCTCCT GCTGGATTTC
TrVeBC2_24   AGTCCCTAGA TCCATTTCTC TTGCTAGATG AATTTGCTGT TACACCTCCT GCTGGATTTC
TrVeGnuB1_   AGTCCCTAGA TCCATTTCTC TTGCTAGATG AATTTGCTGT TACACCTCCT GCTGGATTTC
cTvPirin_3   AGTCCCTAGA TCCATTTCTC TTGCTAGATG AATTTGCTGT TACACCTCCC GCTGGATTTC
TrVe63GuB1   AGTCCCTAGA TCCATTTCTC TTGCTAGATG AATTTGCTGT TACACCTCCT GCTGGATTTC
TrVe62GuB1   AGTCCCTAGA TCCATTTCTC TTGCTAGATG AATTTGCTGT TACACCTCCT GCTGGATTTC
TrVe61GuB1   AGTCCCTAGA TCCATTTCTC TTGCTAGATG AATTTGCTGT TACACCTCCT GCTGGATTTC
TrVe3GB1_6   AGTCCCTAGA TCCATTTCTC TTGCTAGATG AATTTGCTGT TACACCTCCT GCTGGATTTC
Contig5118   AGTCCCTAGA TCCATTTCTC TTGCTAGATG AATTTGCTGT TACACCTCCT GCTGGATTTC
TrVeBC1_14   AGTCCCTAGA TCCATTTCTC TTGCTAGATG AATTTGCTGT TACACCTCCT GCTGGATTTC
TrVe61FuB1   AGTCCCTAGA TCCATTTCTC TTGCTAGATG AATTTGCTGT TACACCTCCT GCTGGATTTC
Contig492    ---------- ---------- ---------- ---------- ---------- ----------
```

```
              481                                                         540
 5'Pirin_1    CTGACCATCC TCACCGAGGC TTTGAGACTG TTACATATAT GTTGCAGGGA GGCGTCACAC
 5'Pirin_3    CTGACCATCC TCACCGAGGC TTTGAGACTG TTACATATAT GTTGCAGGGA GGCGTCACAC
 5'Pirin_2    CTGACCATCC TCACCGAGGC TTTGAGACTG TTACATATAT GTTGCAGGGA GGCGTCACAC
 5'Pirin_4    CTGACCATCC TCACCGAGGC TTTGAGACTG TTACATATAT GTTGCAGGGA GGCGTCACAC
 5'Pirin_5    CTGACCATCC TCACCGAGGC TTTGAGACTG TTACATATAT GTTGCAGGGA GGCGTCACAC
cTvPirin_1    CTGACCATCC TCACCGAGGC TTTGAGACTG TTACATATAT GTTGCAGGGA GGCGTCACAC
cTvPirin_2    CTGACCATCC TCACCGAGGC TTTGAGACTG TTACATATAT GTTGCAGGGA GGCGTCACAC
cTvPirin_4    CTGACCATCC TCACCGAGGC TTTGAGACTG TTACATATAT GTTGCAGGGA GGCGTCACAC
TrVeBC2_24    CTGACCATCC TCACCGAGGC TTTGAGACTG TTACATATAT GTTGCAGGGA GGCGTCACAC
TrVeGnuB1_    CTGACCATCC TCACCGAGGC TTTGAGACTG TTACATATAT GTTGCAGGGA GGCGTCACAC
cTvPirin_3    CTGACCATCC TCACCGAGGC TTTGAGACTG TTACATATAT GTTGCAGGGA GGCGTCACAC
TrVe63GuB1    CTGACCATCC TCACCGAGGC TTTGAGACTG TTACATATAT GTTGCAGGGA GGCGTCACAC
TrVe62GuB1    CTGACCATCC TCACCGAGGC TTTGAGACTG TTACATATAT GTTGCAGGGA GGCGTCACAC
TrVe61GuB1    CTGACCATCC TCACCGAGGC TTTGAGACTG TTACATATAT GTTGCAGGGA GGCGTCACAC
TrVe3GB1_6    CTGACCATCC TCACCGAGGC TTTGAGACTG TTACATATAT GTTGCAGGGA GGCGTCACAC
Contig5118    CTGACCATCC TCACCGAGGC TTTGAGACTG TTACATATAT GTTGCAGGGA GGCGTCACAC
TrVeBC1_14    CTGACCATCC TCACCGAGGC TTTGAGACTG TTACATATAT GTTGCAGGGA GGCGTCACAC
TrVe61FuB1    CTGACCATCC TCACCGAGGC TTTGAGACTG TTACATATAT GTTGCAGGGA GGCGTCACAC
Contig492     ---------- ---------- ---------- ---------- ---------- ----------

              541                                                         600
 5'Pirin_1    ATCAAGATTT TGCCGGTCAT AAGGGCACCA TAGGTGCTGG TGACGTGCAG TGGATGACTG
 5'Pirin_3    ATCAAGATTT TGCCGGTCAT AAGGGCACCA TAGGTGCTGG TGACGTGCAG TGGATGACTG
 5'Pirin_2    ATCAAGATTT TGCCGGTCAT AAGGGCACCA TAGGTGCTGG TGATGTGCAG TGGATGACTG
 5'Pirin_4    ATCAAGATTT TGCCGGTCAT AAGGGCACCA TAGGTGCTGG TGACGTGCAG TGGATGACTG
 5'Pirin_5    ATCAAGATTT TGCCGGTCAT AAGGGCACCA TAGGTGCTGG TGACGTGCAG TGGATGACTG
cTvPirin_1    ATCAAGATTT TGCCGGTCAT AAGGGCACCA TAGGTGCTGG TGACGTGCAG TGGATGACTG
cTvPirin_2    ATCAAGATTT TGCCGGTCAT AAGGGCACCA TAGGTGCTGG TGACGTGCAG TGGATGGCTG
cTvPirin_4    ATCAAGATTT TGCCGGTCAT AAGGGCACCA TAGGTGCTGG TGACGTGCAG TGGATGACTG
TrVeBC2_24    ATCAAGATTT TGCCGGTCAT AAGGGCACCA TAGGTGCTGG TGACGTGCAG TGGATGACTG
TrVeGnuB1_    ATCAAGATTT TGCCGGTCAT AAGGGCACCA TAGGTGCTGG TGACGTGCAG TGGATGACTG
cTvPirin_3    ATCAAGATTT TGCCGGTCAT AAGGGCACCA TAGGTGCTGG TGACGTGCAG TGGATGACTG
TrVe63GuB1    ATCAAGATTT TGCCGGTCAT AAGGGCACCA TAGGTGCTGG TGACGTGCAG TGGATGACTG
TrVe62GuB1    ATCAAGATTT TGCCGGTCAT AAGGGCACCA TAGGTGCTGG TGACGTGCAG TGGATGACTG
TrVe61GuB1    ATCAAGATTT TGCCGGTCAT AAGGGCACCA TAGGTGCTGG TGACGTGCAG TGGATGACTG
TrVe3GB1_6    ATCAAGATTT TGCCGGTCAT AAGGGCACCA TAGGTGCTGG TGACGTGCAG TGGATGACTG
Contig5118    ATCAAGATTT TGCCGGTCAT AAGGGCACCA TAGGTGCTGG TGACGTGCAG TGGATGACTG
TrVeBC1_14    ATCAAGATTT TGCCGGTCAT AAGGGCACCA TAGGTGCTGG TGACGTGCAG TGGATGACTG
TrVe61FuB1    ATCAAGATTT TGCCGGTCAT AAGGGCACCA TAGGTGCTGG TGACGTGCAG TGGATGACTG
Contig492     ---------- ---------- ---------- ---------- ---------- ----------

              601                                                         660
 5'Pirin_1    CCGGCAGAGG TATTGTTCAC TCAGAAATGC CCGC------ ---------- ----------
 5'Pirin_3    CCGGCAGAGG TATTGTTCAC TCAGAAATGC CCGC------ ---------- ----------
 5'Pirin_2    CCGGCAGAGG TATTGTTCAC TCAGAAATGC CCGC------ ---------- ----------
 5'Pirin_4    CCGGCAGAGG TATTGTTCAC TCAGAAATGC CCGC------ ---------- ----------
 5'Pirin_5    CCGGCAGAGG TATTGCTCAC TCAGAAATGC CCGC------ ---------- ----------
cTvPirin_1    CCGGCAGAGG TATTGTTCAC TCAGAAATGC CCGCCGGAGA AGGCACTCAC ACTGGTCTCC
cTvPirin_2    CCGGCAGAGG TATTGTTCAC TTAGAAATGC CCGCCGGAGA AGGCACTCAC ACTGGTCTCC
cTvPirin_4    CCGGCAGAGG TATTGTTCAC TCAGAAATGC CCGCCGGAGA AGGCACTCAC ACTGGTCTCC
TrVeBC2_24    CCGGCAGAGG TATTGTTCAC TCAGAAATGC CCGCCGGAGA AGGCACTCAC ACTGGTCTCC
TrVeGnuB1_    CCGGCAGAGG TATTGTTCAC TCAGAAATGC CCGCCGGAGA AGGCACTCAC ACTGGTCTCC
cTvPirin_3    CCGGCAGAGG TATTGTTCAC TCAGAAATGC CCGCCGGAGA AGGCACTCAC ACTGGTCTCC
TrVe63GuB1    CCGGCAGAGG TATTGTTCAC TCAGAAATGC CCGCCGGAGA AGGCACTCAC ACTGGTCTCC
TrVe62GuB1    CCGGCAGAGG TATTGTTCAC TCAGAAATGC CCGCCGGAGA AGGCACTCAC ACTGGTCTCC
TrVe61GuB1    CCGGCAGAGG TATTGTTCAC TCAGAAATGC CCGCCGGAGA AGGCACTCAC ACTGGTCTCC
TrVe3GB1_6    CCGGCAGAGG TATTGTTCAC TCAGAAATGC CCGCCGGAGA AGGCACTCAC ACTGGTCTCC
Contig5118    CCGGCAGAGG TATTGTTCAC TCAGAAATGC CCGCCGGAGA AGGCACTCAC ACTGGTCTCC
TrVeBC1_14    CCGGCAGAGG TATTGTTCAC TCAGAAATGC CCGCCGGAGA AGGCACTCAC ACTGGTCTCC
TrVe61FuB1    CCGGCAGAGG TATTGTTCAC TCAGAAATGC CCGCCGGAGA AGGCACTCAC ACTGGTCTCC
Contig492     ---------- ---------- ---------- ---------- ---------- ----------
```

```
             661                                                                720
 5'Pirin_1   ---------- ---------- ---------- ---------- ---------- ----------
 5'Pirin_3   ---------- ---------- ---------- ---------- ---------- ----------
 5'Pirin_2   ---------- ---------- ---------- ---------- ---------- ----------
 5'Pirin_4   ---------- ---------- ---------- ---------- ---------- ----------
 5'Pirin_5   ---------- ---------- ---------- ---------- ---------- ----------
cTvPirin_1   AATTGTGGAT CAATCTCTCT TCCAAGGACA AAA-TGATTG AACCTAGGTA TCAAGAATTG
cTvPirin_2   AATTGTGGAT CAATCTCTCT TCCAAGGACA AAA-TGATTG AACCTAGGTA TCAAGAATTG
cTvPirin_4   AATTGTGGAT CAATCTCTCT TCCAAGGACA AAA-TGATTG AACCTAGGTA TCAAGAATTG
TrVeBC2_24   AATTGTGGAT CAATCTCTCT TCCAAGGACA AAA-TGATTG AACCTAGGTA TCAAGAATTG
TrVeGnuB1_   AATTGTGGAT CAATCTCTCT TCCAAGGACA AAA-TGATTG AACCTAGGTA TCAAGAATTG
cTvPirin_3   AATTGTGGAT CAATCTCTCT TCCAAGGACA AAA-TGATTG AACCTAGGCA TCAAGAATTG
TrVe63GuB1   AATTGTGGAT CAATCTCTCT TCCAAGGACA AAA-TGATTG AACCTAGGTA TCAAGAATTG
TrVe62GuB1   AATTGTGGAT CAATCTCTCT TCCAAGGACA AAA-TGATTG AACCTAGGTA TCAAGAATTG
TrVe61GuB1   AATTGTGGAT CAATCTCTCT TCCAAGGACA AAA-TGATTG AACCTAGGTA TCAAGAATTG
TrVe3GB1_6   AATTGTGGAT CAATCTCTCT TCCAAGGACA AAA-TGATTG AACCTAGGTA TCAAGAATTG
Contig5118   AATTGTGGAT CAATCTCTCT TCCAAGGACA AAA-TGATTG AACCTAGGTA TCAAGAATTG
TrVeBC1_14   AATTGTGGAT CAATCTCTCT TCCAAGGACA AAA-TGATTG AACCTAGGTA TCAAGAATTG
TrVe61FuB1   AATTGTGGAT CAATCTCTCT TCCAAGGACA AAAATGATTG AACCTAGGTA TCAAGAA---
Contig492    ---------- ---------- ---------- ---------- ---------- ----------

             721                                                                780
 5'Pirin_1   ---------- ---------- ---------- ---------- ---------- ----------
 5'Pirin_3   ---------- ---------- ---------- ---------- ---------- ----------
 5'Pirin_2   ---------- ---------- ---------- ---------- ---------- ----------
 5'Pirin_4   ---------- ---------- ---------- ---------- ---------- ----------
 5'Pirin_5   ---------- ---------- ---------- ---------- ---------- ----------
cTvPirin_1   CTGAGTAATG ACATCCCCAA ATCACAAAAA GATGGGGTTG AAGTAAAAGT GATCGCAGGA
cTvPirin_2   CTGAGTAATG ACATCCCCAA AGCACAAAAA GATGGGGTTG AAGTAAAAGT GATCGCAGGA
cTvPirin_4   CTGAGTAATG ACATCCCCAA AGCACAAAAA GATGGGGTTG AAGTAAAAGT GGTCGCAGGA
TrVeBC2_24   CTGAGTAATG ACATCCCCAA AGCACAAAAA GATGGGGTTG AAGTAAAAGT GATCGCAGGA
TrVeGnuB1_   CTGAGTAATG ACATCCCCAA AGCACAAAAA GATGGGGTTG AAGTAAAAGT GATCGCAGGA
cTvPirin_3   CTGAGTAATG ACATCCCCAA AGCACAAAAA GATGGGGTTG AAGTAAAAGT GATCGCAGGA
TrVe63GuB1   CTGAGTAATG ACATCCCCAA AGCACAAAAA GATGGGGTTG AAGTAAAAGT GATCGCAGGA
TrVe62GuB1   CTGAGTAATG ACATCCCCAA AGCACAAAAA GATGGGGTTG AAGTAAAAGT GATCGCAGGA
TrVe61GuB1   CTGAGTAATG ACATCCCCAA AGCACAAAAA GATGGGGTTG AAGTAAAAGT GATCGCAGGA
TrVe3GB1_6   CTGAGTAATG ACATCCCCAA AGCACAAAAA GATGGGGTTG AAGTAAAAGT GATCGCAGGA
Contig5118   CTGAGTAATG ACATCCCCAA AGCACAAAAA GATGGGGTTG AAGTAAAAGT GATCGCAGGA
TrVeBC1_14   CTGAGTAATG ACATCCCCAA AKCACAAAAA GATGGGGTTG AAGTAAAAGT GATCGCAGGA
TrVe61FuB1   ---------- ---------- ---------- ---------- ---------- ----------
Contig492    ---------- ---------- ---------- ---------- ---------- ----------

             781                                                                840
 5'Pirin_1   ---------- ---------- ---------- ---------- ---------- ----------
 5'Pirin_3   ---------- ---------- ---------- ---------- ---------- ----------
 5'Pirin_2   ---------- ---------- ---------- ---------- ---------- ----------
 5'Pirin_4   ---------- ---------- ---------- ---------- ---------- ----------
 5'Pirin_5   ---------- ---------- ---------- ---------- ---------- ----------
cTvPirin_1   GAAGCAATGG GTGTTCACTC ACAAGTTTTC ACACGAACCC CAACAATGTA CGTCGATTTC
cTvPirin_2   GAAGCAATGG GTGTTCACTC ACAAGTTTTC ACACGAACCC CAACAATGTA CGTCGATTTC
cTvPirin_4   GAAGCAATGG GTGTTCACTC ACAAGTTTTC CCACGAACCC CAACAATGTA CGTCGATTTC
TrVeBC2_24   GAAGCAATGG GTGTTCACTC ACAAGTTTTC ACACGAACCC CAACAATGTA CGTCGATTTC
TrVeGnuB1_   GAAGCAATGG GTGTTCACTC ACAAGTTTTC ACACGAACCC CAACAATGTA CGTCGATTTC
cTvPirin_3   GAAGCAATGG GTGTTCACCC ACAAGTTTTC ACACGAACCC CAACAATGTA CGTCGATTTC
TrVe63GuB1   GAAGCAATGG GTGTTCACTC ACAAGTTTTC ACACGAACCC CAACAATGTA CGTCGATTTC
TrVe62GuB1   GAAGCAATGG GTGTTCACTC ACAAGTTTTC ACACGAACCC CAACAATGTA CGTCGATTTC
TrVe61GuB1   GAAGCAATGG GTGTTCACTC ACAAGTTTTC ACACGAACCC CAACAATGTA CGTCGATTTC
TrVe3GB1_6   GAAGCAATGG GTGTTCACTC ACAAGTTTTC ACACGAACCC CAACAATGTA CGTCGATTTC
Contig5118   GAAGCAATGG GTGTTCACTC ACAAGTTTTC ACACGAACCC CAACAATGTA CGTCGATTTC
TrVeBC1_14   GAAG------ ---------- ---------- ---------- ---------- ----------
TrVe61FuB1   ---------- ---------- ---------- ---------- ---------- ----------
Contig492    ---------- ---------- ---------- ---------- ---------- ----------
```

```
               841                                                            900
  5'Pirin_1    ---------- ---------- ---------- ---------- ---------- ----------
  5'Pirin_3    ---------- ---------- ---------- ---------- ---------- ----------
  5'Pirin_2    ---------- ---------- ---------- ---------- ---------- ----------
  5'Pirin_4    ---------- ---------- ---------- ---------- ---------- ----------
  5'Pirin_5    ---------- ---------- ---------- ---------- ---------- ----------
cTvPirin_1     ACTCTAAAAC CAAATTCCGA GTATCATCAA GGCATTCCGG ATTCTTGGAA TGCGTTTGTG
cTvPirin_2     ACTCTAAAAC CAAATTCCGA GTATCATCAA GGCATTCCGG ATTCTTGGAA TGCGTTTGTG
cTvPirin_4     ACTCTAAAAC CAAATTCCGA GTATCATCAA GGCATTCCGG ATTCTTGGAA TGCGTTTGTG
TrVeBC2_24     ACTCTAAAAC CAAATTCCGA GTATCATCAA GGCATTCCGG ATTCTTGGAA TGCGTTTGTG
TrVeGnuB1_     ACTCTAAAAC CAAATTCCGA GTATCATCAA GGCATTCCGG ATTCTTGGAA TGCGTTTGTG
cTvPirin_3     ACTCTAAAAC CAGATTCCGA GTATCATCAA GGCATTCCGG ATTCTTGGAA TGCGTTTGTG
TrVe63GuB1     ACTCTAAAAC CAAATTCCGA GTATCATCAA GGCATTCCGG ATTCTTGGAA TGCGTTTGTG
TrVe62GuB1     ACTCTAAAAC CAAATTCCGA GTATCATCAA GGCATTCCGG ATTCTTGGAA TGCGTTTGTG
TrVe61GuB1     ACTCTAAAAC CAAATTCCGA GTATCATCAA GGCATTCCGG ATTCTTGGAA TGCGTTTGTG
TrVe3GB1_6     ACTCTAAAAC CAAATTCCGA GTATCATCAA GGCATTCCGG ATTCTTGGAA TGCGTTTGTG
Contig5118     ACTCTAAAAC CAAATTCCGA GTATCATCAA GGCATTCCGG ATTCTTGGAA TGCGTTTGTG
TrVeBC1_14     ---------- ---------- ---------- ---------- ---------- ----------
TrVe61FuB1     ---------- ---------- ---------- ---------- ---------- ----------
Contig492      ---------- ---------- ---------- ---------- ---------- ----------

               901                                                            960
  5'Pirin_1    ---------- ---------- ---------- ---------- ---------- ----------
  5'Pirin_3    ---------- ---------- ---------- ---------- ---------- ----------
  5'Pirin_2    ---------- ---------- ---------- ---------- ---------- ----------
  5'Pirin_4    ---------- ---------- ---------- ---------- ---------- ----------
  5'Pirin_5    ---------- ---------- ---------- ---------- ---------- ----------
cTvPirin_1     TACATTATTG AAGGTGAAGG TGTATTTGGC ATCCCGAATT CGAAGCCTGC AGAAGCTCAT
cTvPirin_2     TACATTATTG AAGGCGAGGG TGTATTTGGC ATCCCGAATT CGAAGCCTGC AGAAGCTCAT
cTvPirin_4     TACATTATTG AAGGTGAAGG TGTATTTGGC ATCCCGAATT CGAAGCCTGC AGAAGCTCAT
TrVeBC2_24     TACATTATTG AAGGTGAAGG TGTATTTGGC ATCCCGAATT CGAAGCCTGC AGAAGCTCAT
TrVeGnuB1_     TACATTATTG AAGGTGAAGG TGTATTTGGC ATCCCGAATT CGAAGCCTGC AGAAGCTCAT
cTvPirin_3     TACATTATTG AAGGTGAAGG TGTATTTGGC ATCCCGAATT CGAAGCCTGC AGAAGCTCAT
TrVe63GuB1     TACATTATTG AAGGTGAAGG TGTATTTGGC ATCCCGAATT CGAAGCCTGC AGAAGCTCAT
TrVe62GuB1     TACATTATTG AAGGTGAAGG TGTATTTGGC ATCCCGAATT CGAAGCCTGC AGAAGCTCAT
TrVe61GuB1     TACATTATTG AAGGTGAAGG TGTATTTGGC ATCCCGAATT CGAAGCCTGC AGAAGCTCAT
TrVe3GB1_6     TACATTATTG AAGGTGAAGG TGTATTTGGC ATCCCGAATT CGAAGCCTGC AGAAGCTCAT
Contig5118     TA-------- ---------- ---------- ---------- ---------- ----------
TrVeBC1_14     ---------- ---------- ---------- ---------- ---------- ----------
TrVe61FuB1     ---------- ---------- ---------- ---------- ---------- ----------
Contig492      TACATTATTG AAGGTGAAGG TGTATTTGGC ATCCCGAATT CGAAGCCTGC AGAAGCTCAT

               961                                                           1020
  5'Pirin_1    ---------- ---------- ---------- ---------- ---------- ----------
  5'Pirin_3    ---------- ---------- ---------- ---------- ---------- ----------
  5'Pirin_2    ---------- ---------- ---------- ---------- ---------- ----------
  5'Pirin_4    ---------- ---------- ---------- ---------- ---------- ----------
  5'Pirin_5    ---------- ---------- ---------- ---------- ---------- ----------
cTvPirin_1     CATATCTTGG TTTTGGGACC TGGAGAGGGG CTAAGTGTGT GGAACAAGTT CACAGAACCA
cTvPirin_2     CATATCTTGG TTTTGGGACC TGGAGAGGGG CTAAGTGTGT GGAACAAGTT CACAGAACCA
cTvPirin_4     CATATCTTGG TTTTGGGACC TGGAGAGGGG CTAAGTGTGT GGAACAAGTT CACAGAACCA
TrVeBC2_24     CATATCTTGG TTTTGGGACC TGGAGAGGGG CTAAGTGTGT GGAACAAGTT CACAGAACCA
TrVeGnuB1_     CATATCTTGG TTTTGGGACC TGGAGAGGGG CTAAGTGTGT GGAACAAGTT CACAGAACCA
cTvPirin_3     CATATCTTGG TTTTGGGACC TGGAGAGGGG CTAAGTGTGT GGAACAAGTT CACAGAACCA
TrVe63GuB1     CATATCTTGG TTTTGGGACC TGGAGAGGGG CTAAGTGTGT GGAACAAGTT CACAGAACCA
TrVe62GuB1     CATATCTTGG TTTTGGGACC TGGAGAGGGG CTAAGTGTGT GGAACAAGTT CACAGAACCA
TrVe61GuB1     CATATCTTGG TTTTGGGACC TGGAGAGGGG CTAAGTGTGT GGAACAAGTT CACAGAACCA
TrVe3GB1_6     CATATCTTGG TTTTGGGACC TGGAGAGGGG CTAAGTGTGT GGAACAAGTT CACAGAACCA
Contig5118     ---------- ---------- ---------- ---------- ---------- ----------
TrVeBC1_14     ---------- ---------- ---------- ---------- ---------- ----------
TrVe61FuB1     ---------- ---------- ---------- ---------- ---------- ----------
Contig492      CATATCTTGG TTTTGGGACC TGGAGAGGGG CTAAGTGTGT GGAACAAGTT CACAGAACCA
```

```
               1021                                                            1080
 5'Pirin_1     ---------- ---------- ---------- ---------- ---------- ----------
 5'Pirin_3     ---------- ---------- ---------- ---------- ---------- ----------
 5'Pirin_2     ---------- ---------- ---------- ---------- ---------- ----------
 5'Pirin_4     ---------- ---------- ---------- ---------- ---------- ----------
 5'Pirin_5     ---------- ---------- ---------- ---------- ---------- ----------
cTvPirin_1     TTGAGGTTTA TTTTGGTAGG CGGGCAGCCA CTGAATGAGC CTGTGGTTCA GCACGGTCCG
cTvPirin_2     TTGAGGTTTA TTTTGGTAGG CGGGCAGCCA CTGAATGAGC CTGTGGTTCA GCACGGTCCG
cTvPirin_4     TTGAGGTTTA TTTTGGTATG CGGGCAGCCA CTGAATGAGC CTGTGGTTCA GCACGGTCCG
TrVeBC2_24     TTGAGGTTTA TTTTGGTAGG CGGGCAGCCA CTGAATGAGC CTGTGGTTCA GCACGGTCCG
TrVeGnuB1_     TTGAGGTTTA TTTTGGTAGG CGGGCAGCCA CTGAATGAGC CTGTGGTTCA GCACGGTCCG
cTvPirin_3     TTGAGGTTTA TTTTGGTAGG CGGGCAGCCA CTGAATGAGC CTGTGGTTCA GCACGGTCCG
TrVe63GuB1     TTGAGGTTTA TTTTGGTAGG CGGGCAGCCA CTGAATGAGC CTGTGGTTCA GCACGGTCCG
TrVe62GuB1     TTGAGGTTTA TTTTGGTAGG CGGGCAGCCA CTGAATGAGC CTGTGGTTCA GCACGGTCCG
TrVe61GuB1     TTGAGGTTTA TTTTGGTAGG CGGGCAGCCA CTGAATGAGC CTGTGGTTCA GCACGGTCCG
TrVe3GB1_6     TTGAGGTTTA TTTTGGTAGG CGGGCAGCCA CTGAATGAGC CTGTGGTTCA G---------
Contig5118     ---------- ---------- ---------- ---------- ---------- ----------
TrVeBC1_14     ---------- ---------- ---------- ---------- ---------- ----------
TrVe61FuB1     ---------- ---------- ---------- ---------- ---------- ----------
Contig492      TTGAGGTTTA TTTTGGTAGG CGGGCAGCCA CTGAATGAGC CTGTGGTTCA GCACGGTCCG

               1081                                                            1140
 5'Pirin_1     ---------- ---------- ---------- ---------- ---------- ----------
 5'Pirin_3     ---------- ---------- ---------- ---------- ---------- ----------
 5'Pirin_2     ---------- ---------- ---------- ---------- ---------- ----------
 5'Pirin_4     ---------- ---------- ---------- ---------- ---------- ----------
 5'Pirin_5     ---------- ---------- ---------- ---------- ---------- ----------
cTvPirin_1     TTTGTGATGA ATACGCACGC TGAGATTGAC AAAACAATGG AAGACTATTA CTATGGCAAG
cTvPirin_2     TTTGTGATGA ATACGCACGC TGAGATTGAC AAAACAATGG AAGACTATTA CTATGGCAAG
cTvPirin_4     TTTGTGATGA ATACGCACGC TGAGATTGAC AAAACAATGG AAGACTATTA CTATGGCAAG
TrVeBC2_24     TTTGTGATGA ATACGCACGC TGAGATTGAC AAAACAATGG AAGACTATTA CTATGGCAAG
TrVeGnuB1_     TTTGTGATGA ATACGCACGC TGAGATTGAC AAAACAATGG AAGACTATTA CTATGGCAAG
cTvPirin_3     TTTGTGATGA ATACGCACGC TGAGATTGAC AAAACAATGG AAGACTATTA CTATGGCAAG
TrVe63GuB1     TTTGTGATGA ATACGCACGC TGAGATTGAC AAAACAATGG AAGACTATTA CTATGGCAAG
TrVe62GuB1     TTTGTGATGA ATACGCACGC TGAGATTGAC AAAACAATGG AAGACTATTA CTATGGCAAG
TrVe61GuB1     TTTGTGATGA ATACGCACGC TGAGATTGAC AAAACAATGG AAGACTATTA CTATGGCAAG
TrVe3GB1_6     ---------- ---------- ---------- ---------- ---------- ----------
Contig5118     ---------- ---------- ---------- ---------- ---------- ----------
TrVeBC1_14     ---------- ---------- ---------- ---------- ---------- ----------
TrVe61FuB1     ---------- ---------- ---------- ---------- ---------- ----------
Contig492      TTTGTGATGA ATACGCACGC TGAGATTGAC AAAACAATGG AAGACTATTA CTATGGCAAG

               1141                                                            1200
 5'Pirin_1     ---------- ---------- ---------- ---------- ---------- ----------
 5'Pirin_3     ---------- ---------- ---------- ---------- ---------- ----------
 5'Pirin_2     ---------- ---------- ---------- ---------- ---------- ----------
 5'Pirin_4     ---------- ---------- ---------- ---------- ---------- ----------
 5'Pirin_5     ---------- ---------- ---------- ---------- ---------- ----------
cTvPirin_1     AATGGTTTCG AGCTGGCAAA AAAATGGAAA TCTAAATGAG CTACATATAA GCAACTGCTT
cTvPirin_2     AATGGTTTCG AGCTGGCAAA AAAATGGAAA TCTAAATGAG CTACATATAA GCAACTGCTT
cTvPirin_4     AATGGTTTCG AGCTGGCAAA AAAATGGAAA TCTAAATGAG CTACATATAA GCAACTGCTT
TrVeBC2_24     AATGGTTTCG AGCTGGCAAA AAAATGGAAA TCTAAATGAG CTACATATAA GCAACTGCTT
TrVeGnuB1_     AATGGTTTCG AGCTGGCAAA AAAATGGAAA TCTAAATGAG CTACATATAA GCAACTGCTT
cTvPirin_3     AATGGTTTCG AGCTGGCAAA AAAATGGAAA TCTAAATGAG CTACATATAA GCAACTGCTT
TrVe63GuB1     AATGGTTTCG AGCTGGCAAA AAAATGGAAA TCTAAATGAG CTACATATAA GCAACTGCTT
TrVe62GuB1     AATGGTTTCG AGCTGGCAAA AAAATGGAAA TCTAAATGAG CTACATATAA GCAACTGCTT
TrVe61GuB1     AATGGTTTCG AGCTGGCAAA AAAATGGAAA TCTAAATGAG CTACATATAA GCAACTGCTT
TrVe3GB1_6     ---------- ---------- ---------- ---------- ---------- ----------
Contig5118     ---------- ---------- ---------- ---------- ---------- ----------
TrVeBC1_14     ---------- ---------- ---------- ---------- ---------- ----------
TrVe61FuB1     ---------- ---------- ---------- ---------- ---------- ----------
Contig492      AATGGTTTCG AGCTGGCAAA AAAATGGAAA TCTAAATGAG CTACATATAA GCAACTGCTT
```

```
                1201                                                         1260
  5'Pirin_1     ---------- ---------- ---------- ---------- ---------- ----------
  5'Pirin_3     ---------- ---------- ---------- ---------- ---------- ----------
  5'Pirin_2     ---------- ---------- ---------- ---------- ---------- ----------
  5'Pirin_4     ---------- ---------- ---------- ---------- ---------- ----------
  5'Pirin_5     ---------- ---------- ---------- ---------- ---------- ----------
cTvPirin_1      AGGGCCCCAA AAGAATATTT AGAACGTCCA TGTTTTAAAA AGTCCTTATT TCCATTTCAT
cTvPirin_2      AGGGCCCCAA AAGAATATTT AGAACGTCCA TGTTTTAAAA AGTCCTTATT TCCATTTCAT
cTvPirin_4      AGGGCCCCAA AAGAATATTT AGAACGTCCA TGTTTTAAAA AGTCCTTATT TCCATTTCAT
TrVeBC2_24      AGGGCCCCAA AAGAATATTT AGAACGTCCA TGTTTTAAAA AGTCCTTATT TCCATTTCAT
TrVeGnuB1_      AGGGCCCCAA AAGAATATTT AGAACGTCCA TGTTTTAAAA AGTCCTTATT TCCATTTCAT
cTvPirin_3      AGGGCCCCAA AAGAATATTT AGAACGTCCA TGTTTTAAAA AGTCCTTATT TCCATTTCAT
TrVe63GuB1      AGGGCCCCAA AAGAATATTT AGAACGTCTA TCTT---CGA TGTTCAACGG GC--TTGCAA
TrVe62GuB1      AGGGCCCCAA AAGAATATTT AGAACGTCCA TGTTTTAAAA AGTCCTTATT TCCATTTCAT
TrVe61GuB1      AGGGCCCCAA AAGAATATTT AGAACGTCCA ---------- ---------- ----------
TrVe3GB1_6      ---------- ---------- ---------- ---------- ---------- ----------
Contig5118      ---------- ---------- ---------- ---------- ---------- ----------
TrVeBC1_14      ---------- ---------- ---------- ---------- ---------- ----------
TrVe61FuB1      ---------- ---------- ---------- ---------- ---------- ----------
Contig492       AGGGCCCCAA AAGAATATTT AGAACGTCCA TGTTTTAAAA AGTCCTTATT TCCATTTCAT

                1261                                                         1320
  5'Pirin_1     ---------- ---------- ---------- ---------- ---------- ----------
  5'Pirin_3     ---------- ---------- ---------- ---------- ---------- ----------
  5'Pirin_2     ---------- ---------- ---------- ---------- ---------- ----------
  5'Pirin_4     ---------- ---------- ---------- ---------- ---------- ----------
  5'Pirin_5     ---------- ---------- ---------- ---------- ---------- ----------
cTvPirin_1      GTCTTTTTTT T----GTTTA AAAAATAAAG ATATAGGAAA ATAAATATGA AGTGAAATGT
cTvPirin_2      GTCTTTTTTT TT---GTTTA AAAAATAAAG ATATAGGAAA ATAAATATGA AGTGAAATGT
cTvPirin_4      GTCTTTTTTT TT---GTTTA AAAAATAAAG ATATAGGAAA ATAAATATGA AGTGAAATGT
TrVeBC2_24      GTCTTTTTTT TT---GTTTA AAAAATAAAG ATATAGGAAA ATAAATATGA AGTGAAATGT
TrVeGnuB1_      GTCTTTTTTT TT---GTTTA AAAAATAAAG ATATAGGAAA ATAAATATGA AGTGAAATGT
cTvPirin_3      GTCTTTTTTT TTTTTGTTTA AAAAATAAAG ATATAGGAAA ATAAATATGA AGTGAAATGT
TrVe63GuB1      GATCGTTCTC ----AATAAA GAAAACTAAA AGCCCGAATA ACAATTCCGA CG---GACGG
TrVe62GuB1      GTCTTTTTTT TT-------- ---------- ---------- ---------- ----------
TrVe61GuB1      ---------- ---------- ---------- ---------- ---------- ----------
TrVe3GB1_6      ---------- ---------- ---------- ---------- ---------- ----------
Contig5118      ---------- ---------- ---------- ---------- ---------- ----------
TrVeBC1_14      ---------- ---------- ---------- ---------- ---------- ----------
TrVe61FuB1      ---------- ---------- ---------- ---------- ---------- ----------
Contig492       GTCTTTTTTT TTT--GTTTA AAAAATAAAG ATATAGGAAA ATAAATATGA AGTGAAATGT

                1321                                                         1380
  5'Pirin_1     ---------- ---------- ---------- ---------- ---------- ----------
  5'Pirin_3     ---------- ---------- ---------- ---------- ---------- ----------
  5'Pirin_2     ---------- ---------- ---------- ---------- ---------- ----------
  5'Pirin_4     ---------- ---------- ---------- ---------- ---------- ----------
  5'Pirin_5     ---------- ---------- ---------- ---------- ---------- ----------
cTvPirin_1      AATACATCTC GTATTGTGAT TTGTGCCATT TATTTTTATT GGAAAGCTTT TGGTTTCGTT
cTvPirin_2      AATACATCTC GTATTGTGAT TTGTGCCATT TATTTTTATT GGAAAGCTTT TGGTTTCGTT
cTvPirin_4      AATACATCTC GTATTGTGAT TTGTGCCATT TATTTTTATT GGAAAGCTTT TGGTTTCGTT
TrVeBC2_24      AATACATCTC GTATTGTGAT TTGTGCCATT TATTTTTATT GGAAAGCTTT TGGTTTCGTT
TrVeGnuB1_      AATACATCTC GTATTGTGAT TTGTGCCATT TATTTTTATT GGAAAGCTTT TGGTTTCGTT
cTvPirin_3      AATACATCTC GTATTGTGAT TTGTGCCATT TATTTTTATT GGAAAGCTTT TGGTTTCGTT
TrVe63GuB1      GAGGC-TGTC GAAG------ ---------- ---------- ---------- ----------
TrVe62GuB1      ---------- ---------- ---------- ---------- ---------- ----------
TrVe61GuB1      ---------- ---------- ---------- ---------- ---------- ----------
TrVe3GB1_6      ---------- ---------- ---------- ---------- ---------- ----------
Contig5118      ---------- ---------- ---------- ---------- ---------- ----------
TrVeBC1_14      ---------- ---------- ---------- ---------- ---------- ----------
TrVe61FuB1      ---------- ---------- ---------- ---------- ---------- ----------
Contig492       AATACATCTC GTATTGTGAT TTGTGCCATT TATTTTTATT GGAAAGCTTT TGGTTTCGTT
```

```
               1381                                                          1440
 5’Pirin_1     ---------- ---------- ---------- ---------- ---------- ----------
 5’Pirin_3     ---------- ---------- ---------- ---------- ---------- ----------
 5’Pirin_2     ---------- ---------- ---------- ---------- ---------- ----------
 5’Pirin_4     ---------- ---------- ---------- ---------- ---------- ----------
 5’Pirin_5     ---------- ---------- ---------- ---------- ---------- ----------
cTvPirin_1     ---------- ---------- ---------- ---------- ---------- ----------
cTvPirin_2     ---------- ---------- ---------- ---------- ---------- ----------
cTvPirin_4     ---------- ---------- ---------- ---------- ---------- ----------
TrVeBC2_24     ACATAGGTTT GATTTGATTT TGAAGATCCA AAATGTCATT GGCTTGCCCT GTCTATCACT
TrVeGnuB1_     ACATAGGTTT GATTTGATTT TGAAGATCCA AAATGTCATT GGCTTGCCCT GTCTATCACT
cTvPirin_3     ---------- ---------- ---------- ---------- ---------- ----------
TrVe63GuB1     ---------- ---------- ---------- ---------- ---------- ----------
TrVe62GuB1     ---------- ---------- ---------- ---------- ---------- ----------
TrVe61GuB1     ---------- ---------- ---------- ---------- ---------- ----------
TrVe3GB1_6     ---------- ---------- ---------- ---------- ---------- ----------
Contig5118     ---------- ---------- ---------- ---------- ---------- ----------
TrVeBC1_14     ---------- ---------- ---------- ---------- ---------- ----------
TrVe61FuB1     ---------- ---------- ---------- ---------- ---------- ----------
Contig492      AAAAAAAAAA AAAAAAAAAA AAAAGCTTGT ---------- ---------- ----------

               1441                                       1483
 5’Pirin_1     ---------- ---------- ---------- ---------- ---
 5’Pirin_3     ---------- ---------- ---------- ---------- ---
 5’Pirin_2     ---------- ---------- ---------- ---------- ---
 5’Pirin_4     ---------- ---------- ---------- ---------- ---
 5’Pirin_5     ---------- ---------- ---------- ---------- ---
cTvPirin_1     ---------- ---------- ---------- ---------- ---
cTvPirin_2     ---------- ---------- ---------- ---------- ---
cTvPirin_4     ---------- ---------- ---------- ---------- ---
TrVeBC2_24     GTCAATCCAA TCTTTCTTCC TGAGAATGCT GCATACAGAT TTG
TrVeGnuB1_     GTCAATCCAA TCTTTCTTCC TGAGAATGCT GCATACAGAT TTG
cTvPirin_3     ---------- ---------- ---------- ---------- ---
TrVe63GuB1     ---------- ---------- ---------- ---------- ---
TrVe62GuB1     ---------- ---------- ---------- ---------- ---
TrVe61GuB1     ---------- ---------- ---------- ---------- ---
TrVe3GB1_6     ---------- ---------- ---------- ---------- ---
Contig5118     ---------- ---------- ---------- ---------- ---
TrVeBC1_14     ---------- ---------- ---------- ---------- ---
TrVe61FuB1     ---------- ---------- ---------- ---------- ---
Contig492      ---------- ---------- ---------- ---------- ---
```

B. Schematic *TvPirin* gene structure

The genomic sequence of the *TvPirin* gene is presented in Additional file 6.

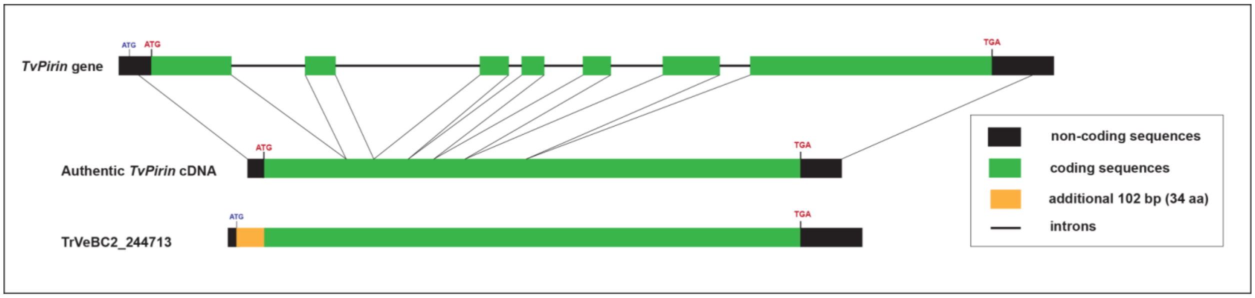

Supplement: Additional file 2 — TvPirin open reading frame encodes a 288-amino acid long protein. [file 1471-2229-13-28-S2.pdf]
