# Supplementary material for: The differentially regulated genes TvQR1 and TvPirin of the parasitic plant Triphysaria exhibit distinctive natural allelic diversity
Source: BMC Plant Biol. 2013 Feb 18;13:28. doi: 10.1186/1471-2229-13-28 (PMC3599707; doi:10.1186/1471-2229-13-28)
Supplement: Additional file 3 — Discontinuous identity between Accession JN606867 and clone TrVe61FuB1_8246. [file 1471-2229-13-28-S3.pdf]

Additional File 3. Discontinuous sequence identity between Accession JN606867 and clone TrVe61FuB1_8246

“Align Sequences Nucleotide BLAST” was performed with the default setting of blastn.

Blast 2 sequences

TrVe61FuB1_8246: Length -- 513 bp | protein...

**Query ID**	lcl|56675	**Subject ID**	56677
**Description**	TrVe61FuB1_8246: Length -- 513 bp | protein binding | other cellular components | other biological processes	**Description**	JN606867
**Molecule type**	nucleic acid	**Molecule type**	nucleic acid
**Query Length**	716	**Subject Length**	2295
		**Program**	BLASTN 2.2.26+

Graphic Summary

Distribution of 7 Blast Hits on the Query Sequence

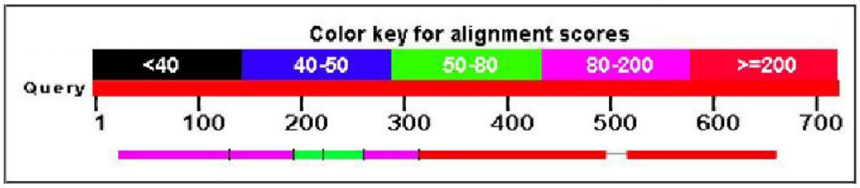

Accession	Description	Max score	Total score	Query coverage	E value	Max ident	Links
56677	JN606867	335	1171	85%	2e-95	100%	

Alignments

```
>lcl|56677 JN606867
Length=2295

                                                  Sort alignments for this subject seq
                                                    E value  Score  Percent identity
                                                    Query start position  Subject start

 Score =  335 bits (181),  Expect = 2e-95
 Identities = 184/186 (99%), Gaps = 0/186 (0%)
 Strand=Plus/Minus

Query  308   CCTGCCAATGCTTCTTCTTACAAGTGCACCATCGCCCTCGGACTGGGACTTGGCCAAGAT  367
             ||||||||||||||||||||||||||||||||||||||||||||||||||||||||||||
Sbjct  186   CCTGCCAATGCTTCTTCTTACAAGTGCACCATCGCCCTCGGACTGGGACTTGGCCAAGAT  127

Query  368   CTTCTTGGCAACTAGTCTGGGCTGTTGGAAACTGGACATGATGATATAACGTCGAGAAGA  427
             |||||||||||||| |||||||||||||||||||||||||||||||||||||||||||||
Sbjct  126   CTTCTTGGCAACTATTCTGGGCTGTTGGAAACTGGACATGATGATATAACGTCGAGAAGA  67

Query  428   CGGGTTTTTTATTATTKGGGATTTGATGTTTATGAGGAATGAAAGTTTAGTAGATACAGA  487
             |||||||||||||||| |||||||||||||||||||||||||||||||||||||||||||
Sbjct  66    CGGGTTTTTTATTATTGGGGATTTGATGTTTATGAGGAATGAAAGTTTAGTAGATACAGA  7

Query  488   GATCAT  493
             ||||||
Sbjct  6     GATCAT  1

 Score =  265 bits (143),  Expect = 2e-74
 Identities = 143/143 (100%), Gaps = 0/143 (0%)
 Strand=Plus/Plus

Query  514   CACGGTCCGTTTGTGATGAATACGCACGCTGAGATTGACAAAACAATGGAAGACTATTAC  573
             ||||||||||||||||||||||||||||||||||||||||||||||||||||||||||||
Sbjct  1988  CACGGTCCGTTTGTGATGAATACGCACGCTGAGATTGACAAAACAATGGAAGACTATTAC  2047

Query  574   TATGGCAAGAATGGTTTCGAGCTGGCaaaaaaaTGGAAATCTAAATGAGCTACATATAAG  633
             ||||||||||||||||||||||||||||||||||||||||||||||||||||||||||||
Sbjct  2048  TATGGCAAGAATGGTTTCGAGCTGGCAAAAAAATGGAAATCTAAATGAGCTACATATAAG  2107

Query  634   CAACTGCTTAGGGCCCCAAAAGA  656
             |||||||||||||||||||||||
Sbjct  2108  CAACTGCTTAGGGCCCCAAAAGA  2130

 Score =  196 bits (106),  Expect = 9e-54
 Identities = 106/106 (100%), Gaps = 0/106 (0%)
 Strand=Plus/Minus

Query  25    TTTTGTCCTTGGAAGAGAGATTGATCCACAATTGGAGACCAGTGTGAGTGCCTTCTCCGG  84
             ||||||||||||||||||||||||||||||||||||||||||||||||||||||||||||
Sbjct  1511  TTTTGTCCTTGGAAGAGAGATTGATCCACAATTGGAGACCAGTGTGAGTGCCTTCTCCGG  1452

Query  85    CGGGCATTTCTGAGTGAACAATACCTCTGCCGGCAGTCATCCACTG  130
             ||||||||||||||||||||||||||||||||||||||||||||||


Sbjct  1451  CGGGCATTTCTGAGTGAACAATACCTCTGCCGGCAGTCATCCACTG  1406

 Score =  121 bits (65),  Expect = 5e-31
 Identities = 65/65 (100%), Gaps = 0/65 (0%)
 Strand=Plus/Minus

Query  128   CTGCACGTCACCAGCACCTATGGTGCCCTTATGACCGGCAAAATCTTGATGTGTGACGCC  187
             ||||||||||||||||||||||||||||||||||||||||||||||||||||||||||||
Sbjct  1136  CTGCACGTCACCAGCACCTATGGTGCCCTTATGACCGGCAAAATCTTGATGTGTGACGCC  1077

Query  188   TCCCT  192
             |||||
Sbjct  1076  TCCCT  1072

 Score =  115 bits (62),  Expect = 3e-29
 Identities = 62/62 (100%), Gaps = 0/62 (0%)
 Strand=Plus/Minus

Query  251   AGGTGTAACAGCAAATTCATCTAGCAAGAGAAATGGATCTAGGGACTTCAACTCAGGCCT  310
             ||||||||||||||||||||||||||||||||||||||||||||||||||||||||||||
Sbjct  412   AGGTGTAACAGCAAATTCATCTAGCAAGAGAAATGGATCTAGGGACTTCAACTCAGGCCT  353

Query  311   GC  312
             ||
Sbjct  352   GC  351

 Score = 76.8 bits (41),  Expect = 1e-17
 Identities = 41/41 (100%), Gaps = 0/41 (0%)
 Strand=Plus/Minus

Query  219   CCTCGGTGAGGATGGTCAGGAAATCCAGCAGGAGGTGTAAC  259
             |||||||||||||||||||||||||||||||||||||||||
Sbjct  878   CCTCGGTGAGGATGGTCAGGAAATCCAGCAGGAGGTGTAAC  838

 Score = 60.2 bits (32),  Expect = 1e-12
 Identities = 32/32 (100%), Gaps = 0/32 (0%)
 Strand=Plus/Minus

Query  190   CCTGCAACATATATGTAACAGTCTCAAAGCCT  221
             ||||||||||||||||||||||||||||||||
Sbjct  993   CCTGCAACATATATGTAACAGTCTCAAAGCCT  962
```
