# Supplementary material for: The differentially regulated genes TvQR1 and TvPirin of the parasitic plant Triphysaria exhibit distinctive natural allelic diversity
Source: BMC Plant Biol. 2013 Feb 18;13:28. doi: 10.1186/1471-2229-13-28 (PMC3599707; doi:10.1186/1471-2229-13-28)
Supplement: Additional file 4 — Multiple alignment of full-length TvQR1 cDNA clones. [file 1471-2229-13-28-S4.pdf]

Additional file 4. Multiple alignment of full-length *TvQR1* cDNA clones

The start codon is depicted with **green bold** letters and the stop codon in **red bold** letters. Dots represent identical nucleotides. Dashes represent missing nucleotides.

```
cTvQR1_1      1 CTGGAATTCATTTTCAAGCTTCTCAAGCCTTTTACATAATAAAAAAAAATTAATTTAATT
cTvQR1_9      1 ............................................................
cTvQR1_8      1 ............................................................
cTvQR1_3      1 ............................................................
cTvQR1_4      1 ............................................................
cTvQR1_5      1 ............................................................
cTvQR1_7      1 ............................................................
cTvQR1_2      1 ..........................T......TA.AT.AT...............G.A.
cTvQR1_6      1 ..........................T......TACA..AT.......-.......G.A.
Consensus     1 ............................................................


cTvQR1_1     61 ATGGCCGGAAAGCTTATGCGTGCGGTTCAGTACGACGGTTAGCTGCTGGTTTGAAGCATG
cTvQR1_9     61 ............................................................
cTvQR1_8     61 ............................................................
cTvQR1_3     61 ............................................................
cTvQR1_4     61 ............................................................
cTvQR1_5     61 ............................................................
cTvQR1_7     61 ............................................................
cTvQR1_2     61 ..........................................................C.
cTvQR1_6     60 ..........................................................C.
Consensus    61 ............................................................


cTvQR1_1    121 TTGAAGTTCCAATACCTAGTCCTGGCAAGGTATAAAGCTTGAAGCCATAAGCTTAAATCA
cTvQR1_9    121 ................................................C...........
cTvQR1_8    121 ............................................................
cTvQR1_3    121 ............................................................
cTvQR1_4    121 ............................................................
cTvQR1_5    121 ............................................................
cTvQR1_7    121 ............................................................
cTvQR1_2    121 ...........G.T........A......................A..............
cTvQR1_6    120 ...........G.T...............................A..............
Consensus   121 ............................................................


cTvQR1_1    181 ACTTGATTGGAAGCTTCAGATCGTCCTTTTCTTCCTCGGAAATTCCCTTTTATACCTGCT
cTvQR1_9    181 ............................................................
cTvQR1_8    181 ............................................................
cTvQR1_3    181 ............................................................
cTvQR1_4    181 ............................................................
cTvQR1_5    181 ............................................................
cTvQR1_7    181 ..................................T.........................
cTvQR1_2    181 ............AA.............A................T...............
cTvQR1_6    180 ............AA.........C...A..........C.....T...............
Consensus   181 ............................................................


cTvQR1_1    241 ACCGACGTGGGGTCCGGATCGGACCGGATGTCAAAAACTTTAAACCCGGTGATAAAGTTG
cTvQR1_9    241 ............................................................
cTvQR1_8    241 ............................................................
```

```
cTvQR1_3   241 ....................................................C.......
cTvQR1_4   241 ....................................................C.......
cTvQR1_5   241 ....................................................C.......
cTvQR1_7   241 ....................................................C.......
cTvQR1_2   241 .....T.......T..A..........G....G.......C...........C.......
cTvQR1_6   240 .....T.......T..A..........G....G.......C...........C.......
Consensus  241 ....................................................C.......

cTvQR1_1   301 TGGCAGTTTTGGAGGAGGTGGCTTAGCCGAATACGGCGTAGCAAGTGCAATCACCGGCCA
cTvQR1_9   301 ............................................................
cTvQR1_8   301 ...............................................A......A....G
cTvQR1_3   301 ...............................................A..C..TA....G
cTvQR1_4   301 ...............................................A..C..TA....G
cTvQR1_5   301 ...............................................A..C..TA....G
cTvQR1_7   301 ...............................................A..C..TA....G
cTvQR1_2   301 ....CA....A....................................A......A....G
cTvQR1_6   300 ....CA....A....................................A..C...A....G
Consensus  301 ...............................................A......A....G

cTvQR1_1   361 CCCGAGGTCTCAGCTGCCGAGAGTTCCGGCCTTCCCATTGGGCCCACATGGCCCTCACCC
cTvQR1_9   361 ............................................................
cTvQR1_8   361 ........................................A...................
cTvQR1_3   361 ........A..............C..A.............A...................
cTvQR1_4   361 ........A..............C..A.............A...................
cTvQR1_5   361 ........A..............C..A.............A...................
cTvQR1_7   361 ........A..............C..A.............A...................
cTvQR1_2   361 .................A.....C..G........T...................A....
cTvQR1_6   360 ........................................A..............A....
Consensus  361 ........................................A...................

cTvQR1_1   421 AACACATCGGCCTAAACCTCGACAAAAGTGAAACATCCTCATCACAGCCGCCTCCGGTGG
cTvQR1_9   421 ............................................................
cTvQR1_8   421 ............................................................
cTvQR1_3   421 ...................................................T........
cTvQR1_4   421 ...................................................T........
cTvQR1_5   421 ...................................................T........
cTvQR1_7   421 ...........................................C.......T........
cTvQR1_2   421 ............................................................
cTvQR1_6   420 ............................................................
Consensus  421 ............................................................

cTvQR1_1   481 TGTTGGCCAATACGCCGTTCGCTAGGAAACACACATGTAACCGCCACATGTGGGTCCCGA
cTvQR1_9   481 ............................................................
cTvQR1_8   481 C..C............A..................C..............C.........
cTvQR1_3   481 C.................A................C.................A......
cTvQR1_4   481 C.................A................C.................A......
cTvQR1_5   481 C.................A................C.................A......
cTvQR1_7   481 C.................A................C.................A......
cTvQR1_2   481 C..................................C........................
cTvQR1_6   480 C..............T...................C.................A......
Consensus  481 C..................................C........................
```

```
cTvQR1_1   541 AACTTTGACTCCTCGGAGCCGACGAGGTTATTGACTATAAAACTCCCGAAGGGGCAGCCC
cTvQR1_9   541 ............................................................
cTvQR1_8   541 ......................T....................C................
cTvQR1_3   541 ...........................................C................
cTvQR1_4   541 ....................A......................C................
cTvQR1_5   541 ...........................................C................
cTvQR1_7   541 ...........................................C................
cTvQR1_2   541 ............................A..............C...........T....
cTvQR1_6   540 ...........................................C................
Consensus  541 ...........................................C................

cTvQR1_1   601 GTCGGGCAAGAAGTATGATGCGGTTATTCATTGTGCGTCGCCTTTGCCATTAAACCGAAC
cTvQR1_9   601 ............................................................
cTvQR1_8   601 ...............C....................A.......................
cTvQR1_3   601 .........A..........................A.......................
cTvQR1_4   601 .........A..........................A.......................
cTvQR1_5   601 .........A..........................A.......................
cTvQR1_7   601 .........A..........................A..A....................
cTvQR1_2   601 .........A..................................................
cTvQR1_6   600 T........A.....C............................................
Consensus  601 .........A..................................................

cTvQR1_1   661 TTGAGCAAACATGGGAAAGTGATTGATATAACTCCCGGTCGTTGACTTCGGCTATGACAA
cTvQR1_9   661 ............................................................
cTvQR1_8   661 .......................C..............A..........T..........
cTvQR1_3   661 .......................C....................................
cTvQR1_4   661 .......................C.........................T..........
cTvQR1_5   661 .......................C.........................T..........
cTvQR1_7   661 .......................C.........................T..........
cTvQR1_2   661 .......................C....................................
cTvQR1_6   660 ............................................................
Consensus  661 .......................C....................................

cTvQR1_1   721 AACTTACGTGCTCGAAGAAACGATTGGTGATGTGATCAAGGGCGAGCATTTGAGTTATCT
cTvQR1_9   721 ............................................................
cTvQR1_8   721 ....C........T..............................................
cTvQR1_3   721 ............................................................
cTvQR1_4   721 ....C........T............................T.................
cTvQR1_5   721 ....G...............A.......T...............................
cTvQR1_7   721 ....G...............A.......T...............................
cTvQR1_2   721 ............................................................
cTvQR1_6   720 ....C.......................................................
Consensus  721 ............................................................

cTvQR1_1   781 TGTTGAGTTAATGAGAGAAGGACCGTTATCGACTCTAAGTTTTCGTTGAGCAAGGCTGAG
cTvQR1_9   781 ............................................................
cTvQR1_8   781 ...C.G.................A....................T...............
cTvQR1_3   781 .......................G..................C....A..T.........
cTvQR1_4   781 ..........................................C.......T.........
cTvQR1_5   781 .......................G.................CC.T.....T.........
cTvQR1_7   781 .......................G.................CC.T.....T.........
cTvQR1_2   781 .......................G..................C....A..T.........
cTvQR1_6   780 ...C......................................C....A..T.........
```

```
Consensus   780 ..........................................C.......T.........

cTvQR1_1    841 GAGGCTTGGGCGACGGCCATGCTACCGGGAAAATCGTTGTCGAGCCATAAGTTAGTAAGA
cTvQR1_9    841 ..........................................................A.
cTvQR1_8    841 ...............................G............................
cTvQR1_3    841 ............................................................
cTvQR1_4    841 ...............................G............................
cTvQR1_5    841 ...............................G............................
cTvQR1_7    841 ...............................G............................
cTvQR1_2    841 ...............................G............................
cTvQR1_6    840 ...............................G............................
Consensus   839 ...............................G............................

cTvQR1_1    901 TTTTATGATATTGTAATGTGGAATTTGGCTTATGACTTGTTTTGGTAATCGATATGAGAT
cTvQR1_9    901 .................................................G..........
cTvQR1_8    901 ..............................................G.............
cTvQR1_3    901 ..............................................G.............
cTvQR1_4    901 ..............................................G.............
cTvQR1_5    901 ...............................G.A...............G..........
cTvQR1_7    901 ...............................G.A...............G..........
cTvQR1_2    901 ..............................................G.............
cTvQR1_6    900 ...............................G.A...............G..........
Consensus   899 ..............................................G.............

cTvQR1_1    961 CTTTTGTTAACCTA
cTvQR1_9    961 ..............
cTvQR1_8    961 ..............
cTvQR1_3    961 ..............
cTvQR1_4    961 ..............
cTvQR1_5    961 ..............
cTvQR1_7    961 ..............
cTvQR1_2    961 ..............
cTvQR1_6    960 ..............
Consensus   959 ..............
```
