# Supplementary material for: The differentially regulated genes TvQR1 and TvPirin of the parasitic plant Triphysaria exhibit distinctive natural allelic diversity
Source: BMC Plant Biol. 2013 Feb 18;13:28. doi: 10.1186/1471-2229-13-28 (PMC3599707; doi:10.1186/1471-2229-13-28)
Supplement: Additional file 5 — Multiple sequence alignment of all genomic TvQR1 alleles. [file 1471-2229-13-28-S5.pdf]

Additional file 5. Multiple sequence alignment of all genomic *TvQR1* alleles

Two alleles of each plant are designated as “A” and “B”. Exon: upper case letters, intron: lower case letters. R: responsive to DMBQ, r: responsive to peonidin, N: non-responsive to DMBQ, n: non-responsive to peonidine.

```
             1                                                          60
gTvQR1_r1A  ATGGCCGGAA AGCTTATGCG TGCGGTTCAG TACGACGGTT ATAGCGGTGG AGCTGCTGGT
gTvQR1_r1B  ATGGCCGGAA AGCTTATGCG TGCGGTTCAG TACGACGGTT ATAGCGGTGG AGCTGCTGGT
gTvQR1_R3A  ATGGCCGGAA AGCTTATGCG TGCGGTTCAG TACGACGGTT ATAGCGGTGG AGCTGCTGGT
gTvQR1_R3B  ATGGCCGGAA AGCTTATGCG TGCGGTTCAG TACGACGGTT ATAGCGGTGG AGCTGCTGGT
gTvQR1_N1A  ATGGCCGGAA AGCTTATGCG TGCGGTTCAG TACGACGGTT ATAGCGGTGG AGCTGCTGGT
gTvQR1_N1B  ATGGCCGGAA AGCTTATGCG TGCGGTTCAG TACGACGGTT ATAGCGGTGG AGCTGCTGGT
gTvQR1_r4A  ATGGCCGGAA AGCTTATGCG TGCGGTTCAG TACGACGGTT ATAGCGGTGG AGCTGCTGGT
gTvQR1_r4B  ATGGCCGGAA AGCTTATGCG TGCGGTTCAG TACGACGGTT ATAGCGGTGG AGCTGCTGGT
gTvQR1_r7A  ATGGCCGGAA AGCTTATGCG TGCGGTTCAG TACGACGGTT ATAGCGGTGG AGCTGCTGGT
gTvQR1_r7B  ATGGCCGGAA AGCTTATGCG TGCGGTTCAG TACGACGGTT ATAGCGGTGG AGCTGCTGGT
gTvQR1_r3A  ATGGCCGGAA AGCTTATGCG TGCGGTTCAG TACGACGGTT ATAGCGGTGG AGCTGCTGGT
gTvQR1_r3B  ATGGCCGGAA AGCTTATGCG TGCGGTTCAG TACGACGGTT ATAGCGGTGG AGCTGCTGGT
gTvQR1_r6A  ATGGCCGGAA AGCTTATGCG TGCGGTTCAG TACGACGGTT ATAGCGGTGG AGCTGCTGGT
gTvQR1_r6B  ATGGCCGGAA AGCTTATGCG TGCGGTTCAG TACGACGGTT ATAGCGGTGG AGCTGCTGGT
gTvQR1_r2A  ATGGCCGGAA AGCTTATGCG TGCGGTTCAG TACGACGGTT ATGGCGGTGG AGCTGCTGGT
gTvQR1_r2B  ATGGCCGGAA AGCTTATGCG TGCGGTTCAG TACGACGGTT ATGGCGGTGG AGCTGCTGGT
gTvQR1_R1A  ATGGCCGGAA AGCTTATGCG TGCGGTTCAG TACGACGGTT ATGGCGGTGG AGCTGCTGGT
gTvQR1_R1B  ATGGCCGGAA AGCTTATGCG TGCGGTTCAG TACGACGGTT ATGGCGGTGG AGCTGCTGGT
gTvQR1_R4B  ATGGCCGGAA AGCTTATGCG TGCGGTTCAG TACGACGGTT ATGGCGGTGG AGCTGCTGGT
gTvQR1_R4A  ATGGCCGGAA AGCTTATGCG TGCGGTTCAG TACGACGGTT ATGGCGGTGG AGCTGCTGGT
gTvQR1_R6B  ATGGCCGGAA AGCTTATGCG TGCGGTTCAG TACGACGGTT ATGGCGGTGG AGCTGCTGGT
gTvQR1_R6A  ATGGCCGGAA AGCTTATGCG TGCGGTTCAG TACGACGGTT ATGGCGGTGG AGCTGCTGGT
gTvQR1_R5A  ATGGCCGGAA AGCTTATGCG TGCGGTTCAG TACGACGGTT ATGGCGGTGG AGCTGCTGGT
gTvQR1_R5B  ATGGCCGGAA AGCTTATGCG TGCGGTTCAG TACGACGGTT ATGGCGGTGG AGCTGCTGGT
gTvQR1_r8A  ATGGCCGGAA AGCTTATGCG TGCGGTTCAG TACGACGGTT ATGGCGGTGG AGCTGCTGGT
gTvQR1_r8B  ATGGCCGGAA AGCTTATGCG TGCGGTTCAG TACGACGGTT ATGGCGGTGG AGCTGCTGGT
gTvQR1_r5A  ATGGCCGGAA AGCTTATGCG TGCGGTTCAG TACGACGGTT ATGGCGGTGG AGCTGCTGGT
gTvQR1_r5B  ATGGCCGGAA AGCTTATGCG TGCGGTTCAG TACGACGGTT ATGGCGGTGG AGCTGCTGGT
gTvQR1_R2A  ATGGCCGGAA AGCTTATGCG TGCGGTTCAG TACGACGGTT ATGGCGGTGG AGCTGCTGGT
gTvQR1_R2B  ATGGCCGGAA AGCTTATGCG TGCGGTTCAG TACGACGGTT ATGGCGGTGG AGCTGCTGGT
gTvQR1_n1A  ATGGCCGGAA AGCTTATGCG TGCGGTTCAG TACGACGGTT ATGGCGGTGG AGCTGCTGGT
gTvQR1_n1B  ATGGCCGGAA AGCTTATGCG TGCGGTTCAG TACGACGGTT ATGGCGGTGG AGCTGCTGGT
gTvQR1_N2B  ATGGCCGGAA AGCTTATGCG TGCGGTTCAG TACGACGGTT ATGGCGGTGG AGCTGCTGGT
gTvQR1_N2A  ATGGCCGGAA AGCTTATGCG TGCGGTTCAG TACGACGGTT ATGGCGGTGG AGCTGCTGGT
gTvQR1_n2A  ATGGCCGGAA AGCTTATGCG TGCGGTTCAG TACGACGGTT ATGGCGGTGG AGCTGCTGGT
gTvQR1_n2B  ATGGCCGGAA AGCTTATGCG TGCGGTTCAG TACGACGGTT ATGGCGGTGG AGCTGCTGGT
gTvQR1_N3A  ATGGCCGGAA AGCTTATGCG TGCGGTTCAG TACGACGGTT ATGGCGGTGG AGCTGCTGGT
gTvQR1_N3B  ATGGCCGGAA AGCTTATGCG TGCGGTTCAG TACGACGGTT ATGGCGGTGG AGCTGCTGGT
gTvQR1_n3A  ATGGCCGGAA AGCTTATGCG TGCGGTTCAG TACGACGGTT ATGGCGGTGG AGCTGCTGGT
gTvQR1_n3B  ATGGCCGGAA AGCTTATGCG TGCGGTTCAG TACGACGGTT ATGGCGGTGG AGCTGCTGGT
```

```
              61                                                      120
gTvQR1_r1A    TTGAAGgtgc ttg---ttcc ttgtttatct ----tactaa taatttctta ----cttaat
gTvQR1_r1B    TTGAAGgtgc ttg---ttcc ttgtttatct ----tactaa taatttctta ----cttaat
gTvQR1_R3A    TTGAAGgtgc ttg---ttcc ttgtttatct ----tactaa taatttctta ----cttaat
gTvQR1_R3B    TTGAAGgtgc ttg---ttcc ttgtttatct ----tactaa taatttctta ----cttaat
gTvQR1_N1A    TTGAAGgtgc ttg---ttcc ttgtttatct ----tactaa taatttctta ----cttaat
gTvQR1_N1B    TTGAAGgtgc ttg---ttcc ttgtttatct ----tactaa taatttctta ----cttaat
gTvQR1_r4A    TTGAAGgtgc tcg---ttct ttgttcatct ----tactaa taatttctta ----cttaat
gTvQR1_r4B    TTGAAGgtgc tcg---ttct ttgttcatct ----tactaa taatttctta ----cttaat
gTvQR1_r7A    TTGAAGgtgc ttg---ttcc ttgtttatct ----tactaa taatttctta ----cttaat
gTvQR1_r7B    TTGAAGgtgc ttg---ttcc ttgtttatct ----tactaa taatttctta ----cttaat
gTvQR1_r3A    TTGAAGgtgc ttg---ttcc ttgtttatct ----tactaa taatttctta ----cttaat
gTvQR1_r3B    TTGAAGgtgc ttg---ttcc ttgtttatct ----tactaa taatttctta ----cttaat
gTvQR1_r6A    TTGAAGgtgc ttg---ttcc ttgtttatct ----tactaa taatttctta ----cttaat
gTvQR1_r6B    TTGAAGgtgc ttg---ttcc ttgtttatct ----tactaa taatttctta ----cttaat
gTvQR1_r2A    TTGAAGgtgc tttaatttcc ttgtttttt- ---ctattaa tattttctta gcaacttaat
gTvQR1_r2B    TTGAAGgtgc tttaatttcc ttgtttttt- ---ctattaa tattttctta gcaacttaat
gTvQR1_R1A    TTGAAGgtgc tttaatttcc ttgtttttt- ---ctattaa tattttctta gcaacttaat
gTvQR1_R1B    TTGAAGgtgc tttaatttcc ttgtttttt- ---ctattaa tattttctta gcaacttaat
gTvQR1_R4B    TTGAAGgtgc tttaatttcc ttgtttttt- ---ctattaa tattttctta gcaacttaat
gTvQR1_R4A    TTGAAGgtgc tttaatttcc ttgtttttt- ---ctattaa tattttctta gcaacttaat
gTvQR1_R6B    TTGAAGgtgc tttaatttcc ttgtttttt- ---ctattaa tattttctta gcaacttaat
gTvQR1_R6A    TTGAAGgtgc tttaatttcc ttgtttttt- ---ctattaa tattttctta gcaacttaat
gTvQR1_R5A    TTGAAGgtgc tttaatttcc ttgtttttt- ---ctattaa tattttctta gcaacttaat
gTvQR1_R5B    TTGAAGgtgc tttaatttcc ttgtttttt- ---ctattaa tattttctta gcaacttaat
gTvQR1_r8A    TTGAAGgtgc tttaatttcc ttgttttttt tttctattaa tattttctta gcaacttaat
gTvQR1_r8B    TTGAAGgtgc tttaatttcc ttgttttttt tttctattaa tattttctta gcaacttaat
gTvQR1_r5A    TTGAAGgtgc tttaatttcc ttgttttttt tttctattaa tattttctta gcaacttaat
gTvQR1_r5B    TTGAAGgtgc tttaatttcc ttgttttttt tttctattaa tattttctta gcaacttaat
gTvQR1_R2A    TTGAAGgtgc tttaatttcc ttgttttttt tttctattaa tattttctta gcaacttaat
gTvQR1_R2B    TTGAAGgtgc tttaatttcc ttgttttttt tttctattaa tattttctta gcaacttaat
gTvQR1_n1A    TTGAAGgtgc tttaatttcc ttgttttttt tttctattaa tattttctta gcaacttaat
gTvQR1_n1B    TTGAAGgtgc tttaatttcc ttgttttttt tttctattaa tattttctta gcaacttaat
gTvQR1_N2B    TTGAAGgtgc tttaatttcc ttgttttttt tttctattaa tattttctta gcaacttaat
gTvQR1_N2A    TTGAAGgtgc tttaatttcc ttgttttttt tttctattaa tattttctta gcaacttaat
gTvQR1_n2A    TTGAAGgtgc tttaatttcc ttgttttttt tttctattaa tattttctta gcaacttaat
gTvQR1_n2B    TTGAAGgtgc tttaatttcc ttgttttttt tttctattaa tattttctta gcaacttaat
gTvQR1_N3A    TTGAAGgtgc tttaatttcc ttgttttttt tttctattaa tattttctta gcaacttaat
gTvQR1_N3B    TTGAAGgtgc tttaatttcc ttgttttttt tttctattaa tattttctta gcaacttaat
gTvQR1_n3A    TTGAAGgtgc tttaatttcc ttgttttttt tttctattaa tattttctta gcaacttaat
gTvQR1_n3B    TTGAAGgtgc tttaatttcc ttgttttttt tttctattaa tattttctta gcaacttaat
```

```
            121                                                        180
gTvQR1_r1A  caatataatc ttgaattaat tactgactat ttttaaaatc cagtagtaat tagacgtact
gTvQR1_r1B  caatataatc ttgaattaat tactgactat ttttaaaatc cagtagtaat tagacgtact
gTvQR1_R3A  caatataatc ttgaattaat tactgactat ttttaaaatc cagtagtaat tagacgtact
gTvQR1_R3B  caatataatc ttgaattaat tactgactat ttttaaaatc cagtagtaat tagacgtact
gTvQR1_N1A  caatataatc ttgaattaat tactgattat ttttaaaatc cagtagtaat tagccgtact
gTvQR1_N1B  caatataatc ttgaattaat tactgattat ttttaaaatc cagtagtaat tagccgtact
gTvQR1_r4A  caatataatc ttgaattaat tactgattat ttttaaaatc caatagtaat tagccgtact
gTvQR1_r4B  caatataatc ttgaattaat tactgattat ttttaaaatc caatagtaat tagccgtact
gTvQR1_r7A  caatataatc ttgagttaat tactgattat ttttaaaatc cagtagtaat taggcgtact
gTvQR1_r7B  caatataatc ttgagttaat tactgattat ttttaaaatc cagtagtaat taggcgtact
gTvQR1_r3A  caatataatc ttgaattaat tactgattat ttttaaaatc cagtagtaat tagccgtact
gTvQR1_r3B  caatataatc ttgaattaat tactgattat ttttaaaatc cagtagtaat tagccgtact
gTvQR1_r6A  caatataatc ttgaattaat tactgattat ttttaaaatc cagtagtaat tagccgtact
gTvQR1_r6B  caatataatc ttgaattaat tactgattat ttttaaaatc cagtagtaat tagccgtact
gTvQR1_r2A  caatataatc ttaaattaat tactgattat t--------- ---------- ----------
gTvQR1_r2B  caatataatc ttaaattaat tactgattat t--------- ---------- ----------
gTvQR1_R1A  caatataatc ttaaattaat tactgattat t--------- ---------- ----------
gTvQR1_R1B  caatataatc ttaaattaat tactgattat t--------- ---------- ----------
gTvQR1_R4B  caatataatc ttaaattaat tactgattat t--------- ---------- ----------
gTvQR1_R4A  caatataatc ttaaattaat tactgattat t--------- ---------- ----------
gTvQR1_R6B  caatataatc ttaaattaat tactgattat t--------- ---------- ----------
gTvQR1_R6A  caatataatc ttaaattaat tactgattat t--------- ---------- ----------
gTvQR1_R5A  caatataatc ttaaattaat tactgattat t--------- ---------- ----------
gTvQR1_R5B  caatataatc ttaaattaat tactgattat t--------- ---------- ----------
gTvQR1_r8A  caatataatc ttaaattaat tactgattat t--------- ---------- ----------
gTvQR1_r8B  caatataatc ttaaattaat tactgattat t--------- ---------- ----------
gTvQR1_r5A  caatataatc ttaaattaat tactgattat t--------- ---------- ----------
gTvQR1_r5B  caatataatc ttaaattaat tactgattat t--------- ---------- ----------
gTvQR1_R2A  caatataatc ttaaattaat tactgattat t--------- ---------- ----------
gTvQR1_R2B  caatataatc ttaaattaat tactgattat t--------- ---------- ----------
gTvQR1_n1A  caatataatc ttaaattaat tactgattat t--------- ---------- ----------
gTvQR1_n1B  caatataatc ttaaattaat tactgattat t--------- ---------- ----------
gTvQR1_N2B  caatataatc ttaaattaat tactgattat t--------- ---------- ----------
gTvQR1_N2A  caatataatc ttaaattaat tactgattat t--------- ---------- ----------
gTvQR1_n2A  caatataatc ttaaattaat tactgattat t--------- ---------- ----------
gTvQR1_n2B  caatataatc ttaaattaat tactgattat t--------- ---------- ----------
gTvQR1_N3A  caatataatc ttaaattaat tactgattat t--------- ---------- ----------
gTvQR1_N3B  caatataatc ttaaattaat tactgattat t--------- ---------- ----------
gTvQR1_n3A  caatataatc ttaaattaat tactgattat t--------- ---------- ----------
gTvQR1_n3B  caatataatc ttaaattaat tactgattat t--------- ---------- ----------
```

```
             181                                                        240
gTvQR1_r1A  ttagtgtcca attgtgtaat tttgtgtttc ctatatgtca ttttatacga aagctaaaat
gTvQR1_r1B  ttagtgtcca attgtgtaat tttgtgtttc ctatatgtca ttttatacga aagctaaaat
gTvQR1_R3A  ttagtgtcca attgtgtaat tttgtgtttc ctatatgtca ttttatacga aagctaaaat
gTvQR1_R3B  ttagtgtcca attgtgtaat tttgtgtttc ctatatgtca ttttatacga aagctaaaat
gTvQR1_N1A  ttactgtcca attgtgtaat tttgtgtttc ctatatgtta ttttatacta aagctaaaat
gTvQR1_N1B  ttactgtcca attgtgtaat tttgtgtttc ctatatgtta ttttatacta aagctaaaat
gTvQR1_r4A  ttactgtcca attgtgtaat tttgtgtttc ctatatgtta ttttatacta aagctaaaat
gTvQR1_r4B  ttactgtcca attgtgtaat tttgtgtttc ctatatgtta ttttatacta aagctaaaat
gTvQR1_r7A  ttactgtcca attgtgtaat tttgtgtttc ctatatgtca ttttatacta aagctaaaat
gTvQR1_r7B  ttactgtcca attgtgtaat tttgtgtttc ctatatgtca ttttatacta aagctaaaat
gTvQR1_r3A  ttactgtcca attgtgtaat tttgtgtttc ctatatgtta ttttatacga aagctaaaat
gTvQR1_r3B  ttactgtcca attgtgtaat tttgtgtttc ctatatgtta ttttatacga aagctaaaat
gTvQR1_r6A  ttactgtcca attgtgtaat tttgtgtttc ctatatgtta ttttatacga aagctaaaat
gTvQR1_r6B  ttactgtcca attgtgtaat tttgtgtttc ctatatgtta ttttatacga aagctaaaat
gTvQR1_r2A  -----gtcca att-tgcaaa ttagtagatt aagtaatata tgtaatacgg ag-----tat
gTvQR1_r2B  -----gtcca att-tgcaaa ttagtagatt aagtaatata tgtaatacgg ag-----tat
gTvQR1_R1A  -----gtcca att-tgcaaa ttagtagatt aagtaatata tgtaatacgg ag-----tat
gTvQR1_R1B  -----gtcca att-tgcaaa ttagtagatt aagtaatata tgtaatacgg ag-----tat
gTvQR1_R4B  -----gtcca att-tgcaaa ttagtagatt aagtaatata tgtaatacgg ag-----tat
gTvQR1_R4A  -----gtcca att-tgcaaa ttagtagatt aagtaatata tgtaatacgg ag-----tat
gTvQR1_R6B  -----gtcca att-tgcaaa ttagtagatt aagtaatata tgtaatacgg ag-----tat
gTvQR1_R6A  -----gtcca att-tgcaaa ttagtagatt aagtaatata tgtaatacgg ag-----tat
gTvQR1_R5A  -----gtcca att-tgcaaa ttagtagatt aagtaatata tgtaatacgg ag-----tat
gTvQR1_R5B  -----gtcca att-tgcaaa ttagtagatt aagtaatata tgtaatacgg ag-----tat
gTvQR1_r8A  -----gtcca att-tgcaaa ttagtagatt aagtaatata tgtaatacgg ag-----tat
gTvQR1_r8B  -----gtcca att-tgcaaa ttagtagatt aagtaatata tgtaatacgg ag-----tat
gTvQR1_r5A  -----gtcca att-tgcaaa ttagtagatt aagtaatata tgtaata--- ---------t
gTvQR1_r5B  -----gtcca att-tgcaaa ttagtagatt aagtaatata tgtaata--- ---------t
gTvQR1_R2A  -----gtcca att-tgcaaa ttagtagatt aagtaatata tgtaata--- ---------t
gTvQR1_R2B  -----gtcca att-tgcaaa ttagtagatt aagtaatata tgtaata--- ---------t
gTvQR1_n1A  -----gtcca att-tgcaaa ttagtagatt aagtaatata tgtaata--- ---------t
gTvQR1_n1B  -----gtcca att-tgcaaa ttagtagatt aagtaatata tgtaata--- ---------t
gTvQR1_N2B  -----gtcca att-tgcaaa ttagtagatt aagtaatata tgtaata--- ---------t
gTvQR1_N2A  -----gtcca att-tgcaaa ttagtagatt aagtaatata tgtaata--- ---------t
gTvQR1_n2A  -----gtcca att-tgcaaa ttagtagatt aagtaatata tgtaata--- ---------t
gTvQR1_n2B  -----gtcca att-tgcaaa ttagtagatt aagtaatata tgtaata--- ---------t
gTvQR1_N3A  -----gtcca att-tgcaaa ttagtagatt aagtaatata tgtaata--- ---------t
gTvQR1_N3B  -----gtcca att-tgcaaa ttagtagatt aagtaatata tgtaata--- ---------t
gTvQR1_n3A  -----gtcca att-tgcaaa ttagtagatt aagtaatata tgtaata--- ---------t
gTvQR1_n3B  -----gtcca att-tgcaaa ttagtagatt aagtaatata tgtaata--- ---------t
```

```
                241                                                          300
gTvQR1_r1A   atgttacagC ACGTTGAAGT TCCAGTTCCT AGTCCTGGCA AGGGCGAGGT CCTTATAAAG
gTvQR1_r1B   atgttacagC ACGTTGAAGT TCCAGTTCCT AGTCCTGGCA AGGGCGAGGT CCTTATAAAG
gTvQR1_R3A   atgttacagC ACGTTGAAGT TCCAGTTCCT AGTCCTGGCA AGGGCGAGGT CCTTATAAAG
gTvQR1_R3B   atgttacagC ACGTTGAAGT TCCAGTTCCT AGTCCTGGCA AGGGCGAGGT CCTTATAAAG
gTvQR1_N1A   atgttacagC ACGTTGAAGT TCCAGTTCCT AGTCCAGGCA AGGGCGAGGT CCTTATAAAG
gTvQR1_N1B   atgttacagC ACGTTGAAGT TCCAGTTCCT AGTCCAGGCA AGGGCGAGGT CCTTATAAAG
gTvQR1_r4A   atgttacagC ACGTTGAAGT TCCAGTTCCT AGTCCTGGCA AGGGAGAGGT CCTTATAAAG
gTvQR1_r4B   atgttacagC ACGTTGAAGT TCCAGTTCCT AGTCCTGGCA AGGGAGAGGT CCTTATAAAG
gTvQR1_r7A   atgttacagC ACGTTGAAGT TCCAGTTCCT AGTCCTGGCA AGGGCGAGGT CCTTATAAAG
gTvQR1_r7B   atgttacagC ACGTTGAAGT TCCAGTTCCT AGTCCTGGCA AGGGCGAGGT CCTTATAAAG
gTvQR1_r3A   atgttacagC ACGTTGAAAT TCCAGTTCCT AGTCCAGGCA AGGGCGAGGT CCTTATAAAG
gTvQR1_r3B   atgttacagC ACGTTGAAAT TCCAGTTCCT AGTCCAGGCA AGGGCGAGGT CCTTATAAAG
gTvQR1_r6A   atgttacagC ACGTTGAAAT TCCAGTTCCT AGTCCAGGCA AGGGCGAGGT CCTTATAAAG
gTvQR1_r6B   atgttacagC ACGTTGAAAT TCCAGTTCCT AGTCCAGGCA AGGGCGAGGT CCTTATAAAG
gTvQR1_r2A   attttacagC ATGTTGAAGT TCCAATACCT AGTCCTGGCA AGGGCGAGGT CCTTATAAAG
gTvQR1_r2B   attttacagC ATGTTGAAGT TCCAATACCT AGTCCTGGCA AGGGCGAGGT CCTTATAAAG
gTvQR1_R1A   attttacagC ATGTTGAAGT TCCAATACCT AGTCCTGGCA AGGGCGAGGT CCTTATAAAG
gTvQR1_R1B   attttacagC ATGTTGAAGT TCCAATACCT AGTCCTGGCA AGGGCGAGGT CCTTATAAAG
gTvQR1_R4B   attttacagC ATGTTGAAGT TCCAATACCT AGTCCTGGCA AGGGCGAGGT CCTTATAAAG
gTvQR1_R4A   attttacagC ATGTTGAAGT TCCAATACCT AGTCCTGGCA AGGGCGAGGT CCTTATAAAG
gTvQR1_R6B   attttacagC ATGTTGAAGT TCCAATACCT AGTCCTGGCA AGGGCGAGGT CCTTATAAAG
gTvQR1_R6A   attttacagC ATGTTGAAGT TCCAATACCT AGTCCTGGCA AGGGCGAGGT CCTTATAAAG
gTvQR1_R5A   attttacagC ATGTTGAAGT TCCAATACCT AGTCCTGGCA AGGGCGAGGT CCTTATAAAG
gTvQR1_R5B   attttacagC ATGTTGAAGT TCCAATACCT AGTCCTGGCA AGGGCGAGGT CCTTATAAAG
gTvQR1_r8A   attttacagC ATGTTGAAGT TCCAATACCT AGTCCTGGCA AGGGCGAGGT CCTTATAAAG
gTvQR1_r8B   attttacagC ATGTTGAAGT TCCAATACCT AGTCCTGGCA AGGGCGAGGT CCTTATAAAG
gTvQR1_r5A   atgttacagC ATGATGAAGT TCCAATACCT AGTCCTGGCA AGGGCGAGGT CCTTATAAAG
gTvQR1_r5B   atgttacagc ATGATGAAGT TCCAATACCT AGTCCTGGCA AGGGCGAGGT CCTTATAAAG
gTvQR1_R2A   atgttacagC ATGATGAAGT TCCAATACCT AGTCCTGGCA AGGGCGAGGT CCTTATAAAG
gTvQR1_R2B   atgttacagC ATGATGAAGT TCCAATACCT AGTCCTGGCA AGGGCGAGGT CCTTATAAAG
gTvQR1_n1A   atgttacagC ATGATGAAGT TCCAATACCT AGTCCTGGCA AGGGCGAGGT CCTTATAAAG
gTvQR1_n1B   atgttacagC ATGATGAAGT TCCAATACCT AGTCCTGGCA AGGGCGAGGT CCTTATAAAG
gTvQR1_N2B   atgttacagC ATGATGAAGT TCCAATACCT AGTCCTGGCA AGGGCGAGGT CCTTATAAAG
gTvQR1_N2A   atgttacagC ATGATGAAGT TCCAATACCT AGTCCTGGCA AGGGCGAGGT CCTTATAAAG
gTvQR1_n2A   atgttacagC ATGATGAAGT TCCAATACCT AGTCCTGGCA AGGGCGAGGT CCTTATAAAG
gTvQR1_n2B   atgttacagC ATGATGAAGT TCCAATACCT AGTCCTGGCA AGGGCGAGGT CCTTATAAAG
gTvQR1_N3A   atgttacagC ATGATGAAGT TCCAATACCT AGTCCTGGCA AGGGCGAGGT CCTTATAAAG
gTvQR1_N3B   atgttacagC ATGATGAAGT TCCAATACCT AGTCCTGGCA AGGGCGAGGT CCTTATAAAG
gTvQR1_n3A   atgttacagC ATGATGAAGT TCCAATACCT AGTCCTGGCA AGGGCGAGGT CCTTATAAAG
gTvQR1_n3B   atgttacagC ATGATGAAGT TCCAATACCT AGTCCTGGCA AGGGCGAGGT CCTTATAAAG
```

	301					360
gTvQR1_r1A	CTTGAAGCAA	TAAGCTTAAA	TCAACTTGAT	TGGAAAATTC	AGAACGGCAT	GGTTCGTCCT
gTvQR1_r1B	CTTGAAGCAA	TAAGCTTAAA	TCAACTTGAT	TGGAAAATTC	AGAACGGCAT	GGTTCGTCCT
gTvQR1_R3A	CTTGAAGCAA	TAAGCTTAAA	TCAACTTGAT	TGGAAAATTC	AGAACGGCAT	GGTTCGTCCT
gTvQR1_R3B	CTTGAAGCAA	TAAGCTTAAA	TCAACTTGAT	TGGAAAATTC	AGAACGGCAT	GGTTCGTCCT
gTvQR1_N1A	CTTGAAGCAA	TAAGCTTAAA	TCAACTTGAT	TGGAAAATTC	AGAACGGCAT	GGTTCGTCCT
gTvQR1_N1B	CTTGAAGCAA	TAAGCTTAAA	TCAACTTGAT	TGGAAAATTC	AGAACGGCAT	GGTTCGTCCT
gTvQR1_r4A	CTTGAAGCAG	TAAGCTTAAA	TCAACTTGAT	TGGAAAATTC	AGAACGGCAT	GGTTCGTCCT
gTvQR1_r4B	CTTGAAGCAG	TAAGCTTAAA	TCAACTTGAT	TGGAAAATTC	AGAACGGCAT	GGTTCGTCCT
gTvQR1_r7A	CTTGAAGCAA	TAAGCTTAAA	TCAACTCGAT	TGGAAAATTC	AGAACGGCAT	GGTTCGTCCT
gTvQR1_r7B	CTTGAAGCAA	TAAGCTTAAA	TCAACTCGAT	TGGAAAATTC	AGAACGGCAT	GGTTCGTCCT
gTvQR1_r3A	CTTGAAGCAA	TAAGCTTAAA	TCAACTTGAT	TGGAAAATTC	AGAATGGCAT	GGTTCGTCCT
gTvQR1_r3B	CTTGAAGCAA	TAAGCTTAAA	TCAACTTGAT	TGGAAAATTC	AGAATGGCAT	GGTTCGTCCT
gTvQR1_r6A	CTTGAAGCAA	TAAGCTTAAA	TCAACTTGAT	TGGAAAATTC	AGAATGGCAT	GGTTCGTCCT
gTvQR1_r6B	CTTGAAGCAA	TAAGCTTAAA	TCAACTTGAT	TGGAAAATTC	AGAATGGCAT	GGTTCGTCCT
gTvQR1_r2A	CTTGAAGCAA	TAAGCTTAAA	TCAACTTGAT	TGGAAGCTTC	AGAATGGCAT	GGTTCGTCCT
gTvQR1_r2B	CTTGAAGCAA	TAAGCTTAAA	TCAACTTGAT	TGGAAGCTTC	AGAATGGCAT	GGTTCGTCCT
gTvQR1_R1A	CTTGAAGCAA	TAAGCTTAAA	TCAACTTGAT	TGGAAGCTTC	AGAATGGCAT	GGTTCGTCCT
gTvQR1_R1B	CTTGAAGCAA	TAAGCTTAAA	TCAACTTGAT	TGGAAGCTTC	AGAATGGCAT	GGTTCGTCCT
gTvQR1_R4B	CTTGAAGCAA	TAAGCTTAAA	TCAACTTGAT	TGGAAGCTTC	AGAATGGCAT	GGTTCGTCCT
gTvQR1_R4A	CTTGAAGCAA	TAAGCTTAAA	TCAACTTGAT	TGGAAGCTTC	AGAATGGCAT	GGTTCGTCCT
gTvQR1_R6B	CTTGAAGCAA	TAAGCTTAAA	TCAACTTGAT	TGGAAGCTTC	AGAATGGCAT	GGTTCGTCCT
gTvQR1_R6A	CTTGAAGCAA	TAAGCTTAAA	TCAACTTGAT	TGGAAGCTTC	AGAATGGCAT	GGTTCGTCCT
gTvQR1_R5A	CTTGAAGCAA	TAAGCTTAAA	TCAACTTGAT	TGGAAGCTTC	AGAATGGCAT	GGTTCGTCCT
gTvQR1_R5B	CTTGAAGCAA	TAAGCTTAAA	TCAACTTGAT	TGGAAGCTTC	AGAATGGCAT	GGTTCGTCCT
gTvQR1_r8A	CTTGAAGCCA	TAAGCTTAAA	TCAACTTGAT	TGGAAGCTTC	AGAATGGCAT	GGTTCGTCCT
gTvQR1_r8B	CTTGAAGCCA	TAAGCTTAAA	TCAACTTGAT	TGGAAGCTTC	AGAATGGCAT	GGTTCGTCCT
gTvQR1_r5A	CTTGAAGCCA	TAAGCTTAAA	TCAACTTGAT	TGGAAGCTTC	AGAATGGCAT	GGTTCGTCCT
gTvQR1_r5B	CTTGAAGCCA	TAAGCTTAAA	TCAACTTGAT	TGGAAGCTTC	AGAATGGCAT	GGTTCGTCCT
gTvQR1_R2A	CTTGAAGCCA	TAAGCTTAAA	TCAACTTGAT	TGGAAGCTTC	AGAATGGCAT	GGTTCGTCCT
gTvQR1_R2B	CTTGAAGCCA	TAAGCTTAAA	TCAACTTGAT	TGGAAGCTTC	AGAATGGCAT	GGTTCGTCCT
gTvQR1_n1A	CTTGAAGCCA	TAAGCTTAAA	TCAACTTGAT	TGGAAGCTTC	AGAATGGCAT	GGTTCGTCCT
gTvQR1_n1B	CTTGAAGCCA	TAAGCTTAAA	TCAACTTGAT	TGGAAGCTTC	AGAATGGCAT	GGTTCGTCCT
gTvQR1_N2B	CTTGAAGCCA	TAAGCTTAAA	TCAACTTGAT	TGGAAGCTTC	AGAATGGCAT	GGTTCGTCCT
gTvQR1_N2A	CTTGAAGCCA	TAAGCTTAAA	TCAACTTGAT	TGGAAGCTTC	AGAATGGCAT	GGTTCGTCCT
gTvQR1_n2A	CTTGAAGCCA	TAAGCTTAAA	TCAACTTGAT	TGGAAGCTTC	AGAATGGCAT	GGTTCGTCCT
gTvQR1_n2B	CTTGAAGCCA	TAAGCTTAAA	TCAACTTGAT	TGGAAGCTTC	AGAATGGCAT	GGTTCGTCCT
gTvQR1_N3A	CTTGAAGCCA	TAAGCTTAAA	TCAACTTGAT	TGGAAGCTTC	AGAATGGCAT	GGTTCGTCCT
gTvQR1_N3B	CTTGAAGCCA	TAAGCTTAAA	TCAACTTGAT	TGGAAGCTTC	AGAATGGCAT	GGTTCGTCCT
gTvQR1_n3A	CTTGAAGCCA	TAAGCTTAAA	TCAACTTGAT	TGGAAGCTTC	AGAATGGCAT	GGTTCGTCCT
gTvQR1_n3B	CTTGAAGCCA	TAAGCTTAAA	TCAACTTGAT	TGGAAGCTTC	AGAATGGCAT	GGTTCGTCCT

```
             361                                                        420
gTvQR1_r1A   ATTCTTCCTC AGAAGTTTCC TTTTATACCT Ggtaatttg cttattaca- -------cca
gTvQR1_r1B   ATTCTTCCTC AGAAGTTTCC TTTTATACCT Ggtaatttg cttattaca- -------cca
gTvQR1_R3A   ATTCTTCCTC AGAAGTTTCC TTTTATACCT Ggtaatttg cttattaca- -------cca
gTvQR1_R3B   ATTCTTCCTC AGAAGTTTCC TTTTATACCT Ggtaatttg cttattaca- -------cca
gTvQR1_N1A   ATTCTTCCTC GGAAATTTCC TTTTATACCT Ggtaatttg cttattaca- -------c-a
gTvQR1_N1B   ATTCTTCCTC GGAAATTTCC TTTTATACCT Ggtaatttg cttattaca- -------c-a
gTvQR1_r4A   ATTCTTCCTC GGAAATTTCC TTTTATACCT Ggtaatttg cttattaca- -------c-a
gTvQR1_r4B   ATTCTTCCTC GGAAATTTCC TTTTATACCT Ggtaatttg cttattaca- -------c-a
gTvQR1_r7A   ATTCTTCCTC GCAAATTTCC TTTTATACCT Ggtaatttg cttattaca- -------aca
gTvQR1_r7B   ATTCTTCCTC GCAAATTTCC TTTTATACCT Ggtaatttg cttattaca- -------aca
gTvQR1_r3A   ATTCTTCCTC GCAAATTTCC TTTTATACCT Ggtaatttg ctta------ ----------
gTvQR1_r3B   ATTCTTCCTC GCAAATTTCC TTTTATACCT Ggtaatttg ctta------ ----------
gTvQR1_r6A   ATTCTTCCTC GCAAATTTCC TTTTATACCT Ggtaatttg ctta------ ----------
gTvQR1_r6B   ATTCTTCCTC GCAAATTTCC TTTTATACCT Ggtaatttg ctta------ ----------
gTvQR1_r2A   TTTCTTCCTC GGAAATTCCC TTTTATACCT Ggtaagtttg cttattacaa tttttaacct
gTvQR1_r2B   TTTCTTCCTC GGAAATTCCC TTTTATACCT Ggtaagtttg cttattacaa tttttaacct
gTvQR1_R1A   TTTCTTCCTC GGAAATTCCC TTTTATACCT Ggtaagtttg cttattacaa tttttaacct
gTvQR1_R1B   TTTCTTCCTC GGAAATTCCC TTTTATACCT Ggtaagtttg cttattacaa tttttaacct
gTvQR1_R4B   TTTCTTCCTC GGAAATTCCC TTTTATACCT Ggtaagtttg cttattacaa tttttaacct
gTvQR1_R4A   TTTCTTCCTC GGAAATTCCC TTTTATACCT Ggtaagtttg cttattacaa tttttaacct
gTvQR1_R6B   TTTCTTCCTC GGAAATTCCC TTTTATACCT Ggtaagtttg cttattacaa tttttaacct
gTvQR1_R6A   TTTCTTCCTC GGAAATTCCC TTTTATACCT Ggtaagtttg cttattacaa tttttaacct
gTvQR1_R5A   TTTCTTCCTC GGAAATTCCC TTTTATACCT Ggtaagtttg cttattacaa tttttaacct
gTvQR1_R5B   TTTCTTCCTC GGAAATTCCC TTTTATACCT Ggtaagtttg cttattacaa tttttaacct
gTvQR1_r8A   TTTCTTCCTC GGAAATTCCC TTTTATACCT Ggtaagtttg cttattacaa tttttaacct
gTvQR1_r8B   TTTCTTCCTC GGAAATTCCC TTTTATACCT Ggtaagtttg cttattacaa tttttaacct
gTvQR1_r5A   TTTCTTCCTC GGAAATTCCC TTTTATACCT Ggtaagtttg cttattacaa tttttaacct
gTvQR1_r5B   TTTCTTCCTC GGAAATTCCC TTTTATACCT Ggtaagtttg cttattacaa tttttaacct
gTvQR1_R2A   TTTCTTCCTC GGAAATTCCC TTTTATACCT Ggtaagtttg cttattacaa tttttaacct
gTvQR1_R2B   TTTCTTCCTC GGAAATTCCC TTTTATACCT Ggtaagtttg cttattacaa tttttaacct
gTvQR1_n1A   TTTCTTCCTC GGAAATTCCC TTTTATACCT Ggtaagtttg cttattacaa tttttaacct
gTvQR1_n1B   TTTCTTCCTC GGAAATTCCC TTTTATACCT Ggtaagtttg cttattacaa tttttaacct
gTvQR1_N2B   TTTCTTCCTC GGAAATTCCC TTTTATACCT Ggtaagtttg cttattacaa tttttaacct
gTvQR1_N2A   TTTCTTCCTC GGAAATTCCC TTTTATACCT Ggtaagtttg cttattacaa tttttaacct
gTvQR1_n2A   TTTCTTCCTC GGAAATTCCC TTTTATACCT Ggtaagtttg cttattacaa tttttaacct
gTvQR1_n2B   TTTCTTCCTC GGAAATTCCC TTTTATACCT Ggtaagtttg cttattacaa tttttaacct
gTvQR1_N3A   TTTCTTCCTC GGAAATTCCC TTTTATACCT Ggtaagtttg cttattacaa tttttaacct
gTvQR1_N3B   TTTCTTCCTC GGAAATTCCC TTTTATACCT Ggtaagtttg cttattacaa tttttaacct
gTvQR1_n3A   TTTCTTCCTC GGAAATTCCC TTTTATACCT Ggtaagtttg cttattacaa tttttaacct
gTvQR1_n3B   TTTCTTCCTC GGAAATTCCC TTTTATACCT Ggtaagtttg cttattacaa tttttaacct
```

```
             421                                                           480
gTvQR1_r1A   ctggctgctt accaaaatct taggattaca tcttttgtaa caattctcat tttctttcat
gTvQR1_r1B   ctggctgctt accaaaatct taggattaca tcttttgtaa caattctcat tttctttcat
gTvQR1_R3A   ctggctgctt accaaaatct taggattaca tcttttgtaa caattctcat tttctttcat
gTvQR1_R3B   ctggctgctt accaaaatct taggattaca tcttttgtaa caattctcat tttctttcat
gTvQR1_N1A   ctagctgctt accaaaatct taggattaca actttgtaa caattctcat tttctttcat
gTvQR1_N1B   ctagctgctt accaaaatct taggattaca actttgtaa caattctcat tttctttcat
gTvQR1_r4A   ctagctgctt accaaaatct taggattaca actttgtaa caattctcat tttctttcaa
gTvQR1_r4B   ctagctgctt accaaaatct taggattaca actttgtaa caattctcat tttctttcaa
gTvQR1_r7A   cca-ctactt gcttatcaaa tta-attaca acctttgtaa caattctcat ttcctttcat
gTvQR1_r7B   cca-ctactt gcttatcaaa tta-attaca acctttgtaa caattctcat ttcctttcat
gTvQR1_r3A   ---------- -ccaaaatct taggattaca tattttgtaa tgattctcat tttctttcat
gTvQR1_r3B   ---------- -ccaaaatct taggattaca tattttgtaa tgattctcat tttctttcat
gTvQR1_r6A   ---------- -ccaaaatct taggattaca tattttgtaa tgattctcat tttctttcat
gTvQR1_r6B   ---------- -ccaaaatct taggattaca tattttgtaa tgattctcat tttctttcat
gTvQR1_r2A   atagtaactt atcaaaatct caggattaca aattttgtaa taattcacat tt--------
gTvQR1_r2B   atagtaactt atcaaaatct caggattaca aattttgtaa taattcacat tt--------
gTvQR1_R1A   atagtaactt atcaaaatct caggattaca aattttgtaa taattcacat tt--------
gTvQR1_R1B   atagtaactt atcaaaatct caggattaca aattttgtaa taattcacat tt--------
gTvQR1_R4B   atagtaactt atcaaaatct caggattaca aattttgtaa taattcacat tt--------
gTvQR1_R4A   atagtaactt atcaaaatct caggattaca aattttgtaa taattcacat tt--------
gTvQR1_R6B   atagtaactt atcaaaatct caggattaca aattttgtaa taattcacat tt--------
gTvQR1_R6A   atagtaactt atcaaaatct caggattaca aattttgtaa taattcacat tt--------
gTvQR1_R5A   atagtaactt atcaaaatct caggattaca aattttgtaa taattcacat tt--------
gTvQR1_R5B   atagtaactt atcaaaatct caggattaca aattttgtaa taattcacat tt--------
gTvQR1_r8A   atagtaactt atcaaaatct caggattaca aattttgtaa taattcacat tt--------
gTvQR1_r8B   atagtaactt atcaaaatct caggattaca aattttgtaa taattcacat tt--------
gTvQR1_r5A   atagtaactt atcaaaatct caggattaca aattttgtaa taattcacat tt--------
gTvQR1_r5B   atagtaactt atcaaaatct caggattaca aattttgtaa taattcacat tt--------
gTvQR1_R2A   atagtaactt atcaaaatct caggattaca aattttgtaa taattcacat tt--------
gTvQR1_R2B   atagtaactt atcaaaatct caggattaca aattttgtaa taattcacat tt--------
gTvQR1_n1A   atagtaactt atcaaaatct caggattaca aattttgtaa taattcacat tt--------
gTvQR1_n1B   atagtaactt atcaaaatct caggattaca aattttgtaa taattcacat tt--------
gTvQR1_N2B   atagtaactt atcaaaatct caggattaca aattttgtaa taattcacat tt--------
gTvQR1_N2A   atagtaactt atcaaaatct caggattaca aattttgtaa taattcacat tt--------
gTvQR1_n2A   atagtaactt atcaaaatct caggattaca aattttgtaa taattcacat tt--------
gTvQR1_n2B   atagtaactt atcaaaatct caggattaca aattttgtaa taattcacat tt--------
gTvQR1_N3A   atagtaactt atcaaaatct caggattaca aattttgtaa taattcacat tt--------
gTvQR1_N3B   atagtaactt atcaaaatct caggattaca aattttgtaa taattcacat tt--------
gTvQR1_n3A   atagtaactt atcaaaatct caggattaca aattttgtaa taattcacat tt--------
gTvQR1_n3B   atagtaactt atcaaaatct caggattaca aattttgtaa taattcacat tt--------
```

```
             481                                                       540
gTvQR1_r1A  attttttctc gttttttcgc cgatcaacac gcagCTACCG ATGTGGCTGG AGAGGTGGTT
gTvQR1_r1B  attttttctc gttttttcgc cgatcaacac gcagCTACCG ATGTGGCTGG AGAGGTGGTT
gTvQR1_R3A  attttttctc gttttttcgc cgatcaacac gcagCTACCG ATGTGGCTGG AGAGGTGGTT
gTvQR1_R3B  attttttctc gttttttcgc cgatcaacac gcagCTACCG ATGTGGCTGG AGAGGTGGTT
gTvQR1_N1A  attttttctc gttttttcgc ggatcaacac acagCTACCG ATGTGGCTGG AGAGGTGGTT
gTvQR1_N1B  attttttctc gttttttcgc ggatcaacac acagCTACCG ATGTGGCTGG AGAGGTGGTT
gTvQR1_r4A  aaattttctc gttttttcgc ggatcaacac acagCTACCG ATGTGGCTGG AGAGGTGGTT
gTvQR1_r4B  aaattttctc gttttttcgc ggatcaacac acagCTACCG ATGTGGCTGG AGAGGTGGTT
gTvQR1_r7A  attttttctc gttttttcgc cgatcgacac gcagCTACAG ACGTGGCTGG AGAGGTGGTC
gTvQR1_r7B  attttttctc gttttttcgc cgatcgacac gcagCTACAG ACGTGGCTGG AGAGGTGGTC
gTvQR1_r3A  attttttctc gttttttcgc ggatcaacac acagCTACCG ATGTGGCTGG AGAGGTGGTT
gTvQR1_r3B  attttttctc gttttttcgc ggatcaacac acagCTACCG ATGTGGCTGG AGAGGTGGTT
gTvQR1_r6A  attttttctc gttttttcgc ggatcaacac acagCTACCG ATGTGGCTGG AGAGGTGGTT
gTvQR1_r6B  attttttctc gttttttcgc ggatcaacac acagCTACCG ATGTGGCTGG AGAGGTGGTT
gTvQR1_r2A  -----ttctc gtttttttg- -gccttttgg gcagCTACCG ACGTGGCTGG GGAGGTGGTC
gTvQR1_r2B  -----ttctc gtttttttg- -gccttttgg gcagCTACCG ACGTGGCTGG GGAGGTGGTC
gTvQR1_R1A  -----ttctc gtttttttg- -gccttttgg gcagCTACCG ACGTGGCTGG GGAGGTGGTC
gTvQR1_R1B  -----ttctc gtttttttg- -gccttttgg gcagCTACCG ACGTGGCTGG GGAGGTGGTC
gTvQR1_R4B  -----ttctc gtttttttg- -gccttttgg gcagCTACCG ACGTGGCTGG GGAGGTGGTC
gTvQR1_R4A  -----ttctc gtttttttg- -gccttttgg gcagCTACCG ACGTGGCTGG GGAGGTGGTC
gTvQR1_R6B  -----ttctc gtttttttg- -gccttttgg gcagCTACCG ACGTGGCTGG GGAGGTGGTC
gTvQR1_R6A  -----ttctc gtttttttg- -gccttttgg gcagCTACCG ACGTGGCTGG GGAGGTGGTC
gTvQR1_R5A  -----ttctc gtttttttg- -gccttttgg gcagCTACCG ACGTGGCTGG GGAGGTGGTC
gTvQR1_R5B  -----ttctc gtttttttg- -gccttttgg gcagCTACCG ACGTGGCTGG GGAGGTGGTC
gTvQR1_r8A  -----ttctc gtttttttg- -gcctattgg gcagCTACCG ACGTGGCTGG GGAGGTGGTC
gTvQR1_r8B  -----ttctc gtttttttg- -gcctattgg gcagCTACCG ACGTGGCTGG GGAGGTGGTC
gTvQR1_r5A  -----ttctc gtttttttg- -gcctattgg gcagCTACCG ACGTGGCTGG GGAGGTGGTC
gTvQR1_r5B  -----ttctc gtttttttg- -gcctattgg gcagCTACCG ACGTGGCTGG GGAGGTGGTC
gTvQR1_R2A  -----ttctc gtttttttg- -gcctattgg gcagCTACCG ACGTGGCTGG GGAGGTGGTC
gTvQR1_R2B  -----ttctc gtttttttg- -gcctattgg gcagCTACCG ACGTGGCTGG GGAGGTGGTC
gTvQR1_n1A  -----ttctc gtttttttg- -gcctattgg gcagCTACCG ACGTGGCTGG GGAGGTGGTC
gTvQR1_n1B  -----ttctc gtttttttg- -gcctattgg gcagCTACCG ACGTGGCTGG GGAGGTGGTC
gTvQR1_N2B  -----ttctc gtttttttg- -gcctattgg gcagCTACCG ACGTGGCTGG GGAGGTGGTC
gTvQR1_N2A  -----ttctc gtttttttg- -gcctattgg gcagCTACCG ACGTGGCTGG GGAGGTGGTC
gTvQR1_n2A  -----ttctc gtttttttg- -gcctattgg gcagCTACCG ACGTGGCTGG GGAGGTGGTC
gTvQR1_n2B  -----ttctc gtttttttg- -gcctattgg gcagCTACCG ACGTGGCTGG GGAGGTGGTC
gTvQR1_N3A  -----ttctc gtttttttg- -gcctattgg gcagCTACCG ACGTGGCTGG GGAGGTGGTC
gTvQR1_N3B  -----ttctc gtttttttg- -gcctattgg gcagCTACCG ACGTGGCTGG GGAGGTGGTC
gTvQR1_n3A  -----ttctc gtttttttg- -gcctattgg gcagCTACCG ACGTGGCTGG GGAGGTGGTC
gTvQR1_n3B  -----ttctc gtttttttg- -gcctattgg gcagCTACCG ACGTGGCTGG GGAGGTGGTC
```

```
            541                                                       600
gTvQR1_r1A  CGAATTGGAC CGGGTGTCAA AAACTTCAAA CCCGGTGATA AAGTTGTTGC TATGCTTGGC
gTvQR1_r1B  CGAATTGGAC CGGGTGTCAA AAACTTCAAA CCCGGTGATA AAGTTGTTGC TATGCTTGGC
gTvQR1_R3A  CGAATTGGAC CGGGTGTCAA AAACTTCAAA CCCGGTGATA AAGTTGTTGC TATGCTTGGC
gTvQR1_R3B  CGAATTGGAC CGGGTGTCAA AAACTTCAAA CCCGGTGATA AAGTTGTTGC TATGCTTGGC
gTvQR1_N1A  CGAATCGGAC CGGGTGTCGA AAACTTCAAA CCCGGTGACA AAGTTGTTGC TATGCTTGGC
gTvQR1_N1B  CGAATCGGAC CGGGTGTCGA AAACTTCAAA CCCGGTGACA AAGTTGTTGC TATGCTTGGC
gTvQR1_r4A  CGAATTGGAC CGGGTGTCAA AAACTTCAAA CCCGGTGATA AAGTTGTTGC TATGCTTGGC
gTvQR1_r4B  CGAATTGGAC CGGGTGTCAA AAACTTCAAA CCCGGTGATA AAGTTGTTGC TATGCTTGGC
gTvQR1_r7A  CAAGTCGGAC CGGATGTCAA AAACTTTAAA CCCGGTGATA AAGTTGTTGC TATGCTTGGC
gTvQR1_r7B  CAAGTCGGAC CGGATGTCAA AAACTTTAAA CCCGGTGATA AAGTTGTTGC TATGCTTGGC
gTvQR1_r3A  CGAATCGGAC CGGGTGTCGA AAACTTCAAA CCTGGTGATA AAGTTGTTGC TATGCTTGGC
gTvQR1_r3B  CGAATCGGAC CGGGTGTCGA AAACTTCAAA CCTGGTGATA AAGTTGTTGC TATGCTTGGC
gTvQR1_r6A  CGAATCGGAC CGGGTGTCGA AAACTTCAAA CCTGGTGATA AAGTTGTTGC TATGCTTGGC
gTvQR1_r6B  CGAATCGGAC CGGGTGTCGA AAACTTCAAA CCTGGTGATA AAGTTGTTGC TATGCTTGGC
gTvQR1_r2A  CGGATCGGAC CGGATGTCAA AAACTTTGAA CCCGGTGACA AAGTTGTTGC TATGCTTGGC
gTvQR1_r2B  CGGATCGGAC CGGATGTCAA AAACTTTGAA CCCGGTGACA AAGTTGTTGC TATGCTTGGC
gTvQR1_R1A  CGGATCGGAC CGGATGTCAA AAACTTTGAA CCCGGTGACA AAGTTGTTGC TATGCTTGGC
gTvQR1_R1B  CGGATCGGAC CGGATGTCAA AAACTTTGAA CCCGGTGACA AAGTTGTTGC TATGCTTGGC
gTvQR1_R4B  CGGATCGGAC CGGATGTCAA AAACTTTGAA CCCGGTGACA AAGTTGTTGC TATGCTTGGC
gTvQR1_R4A  CGGATCGGAC CGGATGTCAA AAACTTTGAA CCCGGTGACA AAGTTGTTGC TATGCTTGGC
gTvQR1_R6B  CGGATCGGAC CGGATGTCAA AAACTTTGAA CCCGGTGACA AAGTTGTTGC TATGCTTGGC
gTvQR1_R6A  CGGATCGGAC CGGATGTCAA AAACTTTGAA CCCGGTGACA AAGTTGTTGC TATGCTTGGC
gTvQR1_R5A  CGGATCGGAC CGGATGTCAA AAACTTTGAA CCCGGTGACA AAGTTGTTGC TATGCTTGGC
gTvQR1_R5B  CGGATCGGAC CGGATGTCAA AAACTTTGAA CCCGGTGACA AAGTTGTTGC TATGCTTGGC
gTvQR1_r8A  CGGATCGGAC CGGATGTCAA AAACTTTAAA CCCGGTGATA AAGTTGTTGC TATGCTTGGC
gTvQR1_r8B  CGGATCGGAC CGGATGTCAA AAACTTTAAA CCCGGTGATA AAGTTGTTGC TATGCTTGGC
gTvQR1_r5A  CGGATCGGAC AGGATGTCAA AAACTTTAAA CCCGGTGATA AAGTTGTTGC TATGCTTGGC
gTvQR1_r5B  CGGATCGGAC AGGATGTCAA AAACTTTAAA CCCGGTGATA AAGTTGTTGC TATGCTTGGC
gTvQR1_R2A  CGGATCGGAC AGGATGTCAA AAACTTTAAA CCCGGTGATA AAGTTGTTGC TATGCTTGGC
gTvQR1_R2B  CGGATCGGAC AGGATGTCAA AAACTTTAAA CCCGGTGATA AAGTTGTTGC TATGCTTGGC
gTvQR1_n1A  CGGATCGGAC AGGATGTCAA AAACTTTAAA CCCGGTGATA AAGTTGTTGC TATGCTTGGC
gTvQR1_n1B  CGGATCGGAC AGGATGTCAA AAACTTTAAA CCCGGTGATA AAGTTGTTGC TATGCTTGGC
gTvQR1_N2B  CGGATCGGAC AGGATGTCAA AAACTTTAAA CCCGGTGATA AAGTTGTTGC TATGCTTGGC
gTvQR1_N2A  CGGATCGGAC AGGATGTCAA AAACTTTAAA CCCGGTGATA AAGTTGTTGC TATGCTTGGC
gTvQR1_n2A  CGGATCGGAC AGGATGTCAA AAACTTTAAA CCCGGTGATA AAGTTGTTGC TATGCTTGGC
gTvQR1_n2B  CGGATCGGAC AGGATGTCAA AAACTTTAAA CCCGGTGATA AAGTTGTTGC TATGCTTGGC
gTvQR1_N3A  CGGATCGGAC AGGATGTCAA AAACTTTAAA CCCGGTGATA AAGTTGTTGC TATGCTTGGC
gTvQR1_N3B  CGGATCGGAC AGGATGTCAA AAACTTTAAA CCCGGTGATA AAGTTGTTGC TATGCTTGGC
gTvQR1_n3A  CGGATCGGAC AGGATGTCAA AAACTTTAAA CCCGGTGATA AAGTTGTTGC TATGCTTGGC
gTvQR1_n3B  CGGATCGGAC AGGATGTCAA AAACTTTAAA CCCGGTGATA AAGTTGTTGC TATGCTTGGC
```

```
             601                                                     660
gTvQR1_r1A   CATTTTgtaa gtgtgattaa tttggccttg ttgctgtaaa aactt----- ----------
gTvQR1_r1B   CATTTTgtaa gtgtgattaa tttggccttg ttgctgtaaa aactt----- ----------
gTvQR1_R3A   CATTTTgtaa gtgtgattaa tttggccttg ttgctgtaaa a-ctt----- ----------
gTvQR1_R3B   CATTTTgtaa gtgtgattaa tttggccttg ttgctgtaaa a-ctt----- ----------
gTvQR1_N1A   CATTTTgtaa gtgtgattaa tttggccttg ttgctgtaaa a-ctt----- ----------
gTvQR1_N1B   CATTTTgtaa gtgtgattaa tttggccttg ttgctgtaaa a-ctt----- ----------
gTvQR1_r4A   CATTTTgtaa gtgtgattaa tttggccttg ttgctgtaaa a-ctt----- ----------
gTvQR1_r4B   CATTTTgtaa gtgtgattaa tttggccttg ttgctgtaaa a-ctt----- ----------
gTvQR1_r7A   CATTTTgtaa gtgttattga ttttgccttg ttgctgtaaa aacttgagta aattacaaat
gTvQR1_r7B   CATTTTgtaa gtgttattga ttttgccttg ttgctgtaaa aacttgagta aattacaaat
gTvQR1_r3A   CATTTTgtaa gtgtgattaa tttggcc--- ---ctgtaaa aactt----- ----------
gTvQR1_r3B   CATTTTgtaa gtgtgattaa tttggcc--- ---ctgtaaa aactt----- ----------
gTvQR1_r6A   CATTTTgtaa gtgtgattaa tttggcc--- ---ctgtaaa aactt----- ----------
gTvQR1_r6B   CATTTTgtaa gtgtgattaa tttggcc--- ---ctgtaaa aactt----- ----------
gTvQR1_r2A   AGTTTTgtaa gtgtgattga ttttgccttg gtgcagtaaa aacttggtta tgctaattac
gTvQR1_r2B   AGTTTTgtaa gtgtgattga ttttgccttg gtgcagtaaa aacttggtta tgctaattac
gTvQR1_R1A   AGTTTTgtaa gtgtgattga ttttgccttg gtgcagtaaa aacttggtta tgctaattac
gTvQR1_R1B   AGTTTTgtaa gtgtgattga ttttgccttg gtgcagtaaa aacttggtta tgctaattac
gTvQR1_R4B   AGTTTTgtaa gtgtgattga ttttgccttg gtgcagtaaa aacttggtta tgctaattac
gTvQR1_R4A   AGTTTTgtaa gtgtgattga ttttgccttg gtgcagtaaa aacttggtta tgctaattac
gTvQR1_R6B   AGTTTTgtaa gtgtgattga ttttgccttg gtgcagtaaa aacttggtta tgctaattac
gTvQR1_R6A   AGTTTTgtaa gtgtgattga ttttgccttg gtgcagtaaa aacttggtta tgctaattac
gTvQR1_R5A   AGTTTTgtaa gtgtgattga ttttgccttg gtgcagtaaa a-cttggtta tgctaattac
gTvQR1_R5B   AGTTTTgtaa gtgtgattga ttttgccttg gtgcagtaaa a-cttggtta tgctaattac
gTvQR1_r8A   AGTTTTgtaa gtgtgattga ttttgccttg gtgcagtaaa aacttggtta tgctaattac
gTvQR1_r8B   AGTTTTgtaa gtgtgattga ttttgccttg gtgcagtaaa aacttggtta tgctaattac
gTvQR1_r5A   AGTTTTgtaa gtgtgattga ttttgccttg gtgcagtaaa aacttggtta tgctaattac
gTvQR1_r5B   AGTTTTgtaa gtgtgattga ttttgccttg gtgcagtaaa aacttggtta tgctaattac
gTvQR1_R2A   AGTTTTgtaa gtgtgattga ttttgccttg gtgcagtaaa aacttggtta tgctaattac
gTvQR1_R2B   AGTTTTgtaa gtgtgattga ttttgccttg gtgcagtaaa aacttggtta tgctaattac
gTvQR1_n1A   AGTTTTgtaa gtgtgattga ttttgccttg gtgcagtaaa aacttggtta tgctaattac
gTvQR1_n1B   AGTTTTgtaa gtgtgattga ttttgccttg gtgcagtaaa aacttggtta tgctaattac
gTvQR1_N2B   AGTTTTgtaa gtgtgattga ttttgccttg gtgcagtaaa aacttggtta tgctaattac
gTvQR1_N2A   AGTTTTgtaa gtgtgattga ttttgccttg gtgcagtaaa aacttggtta tgctaattac
gTvQR1_n2A   AGTTTTgtaa gtgtgattga ttttgccttg gtgcagtaaa aacttggtta tgctaattac
gTvQR1_n2B   AGTTTTgtaa gtgtgattga ttttgccttg gtgcagtaaa aacttggtta tgctaattac
gTvQR1_N3A   AGTTTTgtaa gtgtgattga ttttgccttg gtgcagtaaa aacttggtta tgctaattac
gTvQR1_N3B   AGTTTTgtaa gtgtgattga ttttgccttg gtgcagtaaa aacttggtta tgctaattac
gTvQR1_n3A   AGTTTTgtaa gtgtgattga ttttgccttg gtgcagtaaa aacttggtta tgctaattac
gTvQR1_n3B   AGTTTTgtaa gtgtgattga ttttgccttg gtgcagtaaa aacttggtta tgctaattac
```

```
             661                                                         720
gTvQR1_r1A   ---------- ---------- ---------- ---------- ---------- ----------
gTvQR1_r1B   ---------- ---------- ---------- ---------- ---------- ----------
gTvQR1_R3A   ---------- ---------- ---------- ---------- ---------- ----------
gTvQR1_R3B   ---------- ---------- ---------- ---------- ---------- ----------
gTvQR1_N1A   ---------- ---------- ---------- ---------- ---------- ----------
gTvQR1_N1B   ---------- ---------- ---------- ---------- ---------- ----------
gTvQR1_r4A   ---------- ---------- ---------- ---------- ---------- ----------
gTvQR1_r4B   ---------- ---------- ---------- ---------- ---------- ----------
gTvQR1_r7A   attacacttg tctagtgtca aactaataca aatcccaccg taaaatgtaa cctgtaccaa
gTvQR1_r7B   attacacttg tctagtgtca aactaataca aatcccaccg taaaatgtaa cctgtaccaa
gTvQR1_r3A   ---------- ---------- ---------- ---------- ---------- ----------
gTvQR1_r3B   ---------- ---------- ---------- ---------- ---------- ----------
gTvQR1_r6A   ---------- ---------- ---------- ---------- ---------- ----------
gTvQR1_r6B   ---------- ---------- ---------- ---------- ---------- ----------
gTvQR1_r2A   aaataagccc ttgacatttt gtttattttt agttaatacc ttaataataa caactaac--
gTvQR1_r2B   aaataagccc ttgacatttt gtttattttt agttaatacc ttaataataa caactaac--
gTvQR1_R1A   aaataagccc ttgacatttt gtttattttt agttaatacc ttaataataa caactaac--
gTvQR1_R1B   aaataagccc ttgacatttt gtttattttt agttaatacc ttaataataa caactaac--
gTvQR1_R4B   aaataagccc ttgacatttt gtttattttt agttaatacc ttaataataa caactaac--
gTvQR1_R4A   aaataagccc ttgacatttt gtttattttt agttaatacc ttaataataa caactaac--
gTvQR1_R6B   aaataagccc ttgacatttt gtttattttt agttaatacc ttaataataa caactaac--
gTvQR1_R6A   aaataagccc ttgacatttt gtttattttt agttaatacc ttaataataa caactaac--
gTvQR1_R5A   aaataagccc ttgacatttt gtttattttt agttaatacc ttaataataa caactaac--
gTvQR1_R5B   aaataagccc ttgacatttt gtttattttt agttaatacc ttaataataa caactaac--
gTvQR1_r8A   aaataagccc ttgacatttt gtttattttt agttaatacc ttaataataa caactaac--
gTvQR1_r8B   aaataagccc ttgacatttt gtttattttt agttaatacc ttaataataa caactaac--
gTvQR1_r5A   aaataagccc ttgacatttt gtttattttt agttaatacc ttaataataa caactaac--
gTvQR1_r5B   aaataagccc ttgacatttt gtttattttt agttaatacc ttaataataa caactaac--
gTvQR1_R2A   aaataagccc ttgacatttt gtttattttt agttaatacc ttaataataa caactaac--
gTvQR1_R2B   aaataagccc ttgacatttt gtttattttt agttaatacc ttaataataa caactaac--
gTvQR1_n1A   aaataagccc ttgacatttt gtttattttt agttaatacc ttaataataa caactaac--
gTvQR1_n1B   aaataagccc ttgacatttt gtttattttt agttaatacc ttaataataa caactaac--
gTvQR1_N2B   aaataagccc ttgacatttt gtttattttt agttaatacc ttaataataa caactaac--
gTvQR1_N2A   aaataagccc ttgacatttt gtttattttt agttaatacc ttaataataa caactaac--
gTvQR1_n2A   aa-taagccc ttgacatttt gtttattttt agttaatacc ttaataataa caactaac--
gTvQR1_n2B   aa-taagccc ttgacatttt gtttattttt agttaatacc ttaataataa caactaac--
gTvQR1_N3A   aaataagccc ttgacatttt gtttattttt agttaatacc ttaataataa caactaac--
gTvQR1_N3B   aaataagccc ttgacatttt gtttattttt agttaatacc ttaataataa caactaac--
gTvQR1_n3A   aaataagccc ttgacatttt gtttattttt agttaatacc ttaataataa caactaac--
gTvQR1_n3B   aaataagccc ttgacatttt gtttattttt agttaatacc ttaataataa caactaac--
```

```
             721                                                    780
gTvQR1_r1A   ---------- ---------t agacct---c tagggg-tt tataccacta actaaatttt
gTvQR1_r1B   ---------- ---------t agacct---c tagggg-tt tataccacta actaaatttt
gTvQR1_R3A   ---------- ---------t agacct---c tagggg-tt tataccacta actaaatttt
gTvQR1_R3B   ---------- ---------t agacct---c tagggg-tt tataccacta actaaatttt
gTvQR1_N1A   ---------- ---------t agacct---c tagggg-tt tataccacta actaaatttt
gTvQR1_N1B   ---------- ---------t agacct---c tagggg-tt tataccacta actaaatttt
gTvQR1_r4A   ---------- ---------t agacct---c tagggg-tt tataccacta actaaatttt
gTvQR1_r4B   ---------- ---------t agacct---c tagggg-tt tataccacta actaaatttt
gTvQR1_r7A   agtaactatt tatttagttt agacct---c tagagggatt tataccacta actaaatttt
gTvQR1_r7B   agtaactatt tatttagttt agacct---c tagagggatt tataccacta actaaatttt
gTvQR1_r3A   ---------- ---------t agacct---c tagggg-tt tataccacta actaaatttt
gTvQR1_r3B   ---------- ---------t agacct---c tagggg-tt tataccacta actaaatttt
gTvQR1_r6A   ---------- ---------t agacct---c tagggg-tt tataccacta actaaatttt
gTvQR1_r6B   ---------- ---------t agacct---c tagggg-tt tataccacta actaaatttt
gTvQR1_r2A   ---------- ---------a agcactt--- --gccggatt ggtatcttgg attataagtt
gTvQR1_r2B   ---------- ---------a agcactt--- --gccggatt ggtatcttgg attataagtt
gTvQR1_R1A   ---------- ---------a agcactt--- --gccggatt ggtatcttgg attataagtt
gTvQR1_R1B   ---------- ---------a agcactt--- --gccggatt ggtatcttgg attataagtt
gTvQR1_R4B   ---------- ---------a agcactt--- --gccggatt ggtatcttgg attataagtt
gTvQR1_R4A   ---------- ---------a agcactt--- --gccggatt ggtatcttgg attataagtt
gTvQR1_R6B   ---------- ---------a agcactt--- --gccggatt ggtatcttgg attataagtt
gTvQR1_R6A   ---------- ---------a agcactt--- --gccggatt ggtatcttgg attataagtt
gTvQR1_R5A   ---------- ---------a agcactt--- --gccggatt ggtatcttgg attataagtt
gTvQR1_R5B   ---------- ---------a agcactt--- --gccggatt ggtatcttgg attataagtt
gTvQR1_r8A   ---------- ---------a agcacttagc tagccggatt ggtatcttgg attataagtt
gTvQR1_r8B   ---------- ---------a agcacttagc tagccggatt ggtatcttgg attataagtt
gTvQR1_r5A   ---------- ---------a agcacttagc tagccggatt ggtatcttgg attataagtt
gTvQR1_r5B   ---------- ---------a agcacttagc tagccggatt ggtatcttgg attataagtt
gTvQR1_R2A   ---------- ---------a agcacttagc tagccggatt ggtatcttgg attataagtt
gTvQR1_R2B   ---------- ---------a agcacttagc tagccggatt ggtatcttgg attataagtt
gTvQR1_n1A   ---------- ---------a agcacttagc tagccggatt ggtatcttgg attataagtt
gTvQR1_n1B   ---------- ---------a agcacttagc tagccggatt ggtatcttgg attataagtt
gTvQR1_N2B   ---------- ---------a agcacttagc tagccggatt ggtatcttgg attataagtt
gTvQR1_N2A   ---------- ---------a agcacttagc tagccggatt ggtatcttgg attataagtt
gTvQR1_n2A   ---------- ---------a agcacttagc tagccggatt ggtatcttgg attataagtt
gTvQR1_n2B   ---------- ---------a agcacttagc tagccggatt ggtatcttgg attataagtt
gTvQR1_N3A   ---------- ---------a agcacttagc tagccggatt ggtatcttgg attataagtt
gTvQR1_N3B   ---------- ---------a agcacttagc tagccggatt ggtatcttgg attataagtt
gTvQR1_n3A   ---------- ---------a agcacttagc tagccggatt ggtatcttgg attataagtt
gTvQR1_n3B   ---------- ---------a agcacttagc tagccggatt ggtatcttgg attataagtt
```

```
             781                                                        840
gTvQR1_r1A   gtacggggtt aaacgatatt aactaaatct tat----gtt atatcctaat tt--attttc
gTvQR1_r1B   gtacggggtt aaacgatatt aactaaatct tat----gtt atatcctaat tt--attttc
gTvQR1_R3A   gtacggggtt aaacgatatt aactaaatct tat----gtt atatcctaat tt--attttc
gTvQR1_R3B   gtacggggtt aaacgatatt aactaaatct tat----gtt atatcctaat tt--attttc
gTvQR1_N1A   gtacgaggtt aaacgatatt aactaaatct tat----gtt atatcctaat tt--gttttc
gTvQR1_N1B   gtacgaggtt aaacgatatt aactaaatct tat----gtt atatcctaat tt--gttttc
gTvQR1_r4A   gtatgaggtt aaacgatatt aactaaatct tat----gtt atatcctaat tt--attttc
gTvQR1_r4B   gtatgaggtt aaacgatatt aactaaatct tat----gtt atatcctaat tt--attttc
gTvQR1_r7A   gta-gggatt aaacgatatt aactaaatcg tgtaggggtt atatcataat tt--attttc
gTvQR1_r7B   gta-gggatt aaacgatatt aactaaatcg tgtaggggtt atatcataat tt--attttc
gTvQR1_r3A   gtacggggtt aaacgatatt aactaaatct tat----gtt atatcctaat tt--atttcc
gTvQR1_r3B   gtacggggtt aaacgatatt aactaaatct tat----gtt atatcctaat tt--atttcc
gTvQR1_r6A   gtacggggtt aaacgatatt aactaaatct tat----gtt atatcctaat tt--atttcc
gTvQR1_r6B   gtacggggtt aaacgatatt aactaaatct tat----gtt atatcctaat tt--atttcc
gTvQR1_r2A   cgagaatgga gtaaattgca aatatagcct cca----gta atttcgtaac tagcagaaac
gTvQR1_r2B   cgagaatgga gtaaattgca aatatagcct cca----gta atttcgtaac tagcagaaac
gTvQR1_R1A   cgagaatgga gtaaattgca aatatagcct cca----gta atttcgtaac tagcagaaac
gTvQR1_R1B   cgagaatgga gtaaattgca aatatagcct cca----gta atttcgtaac tagcagaaac
gTvQR1_R4B   cgagaatgga gtaaattgca aatatagcct cca----gta atttcgtaac tagcagaaac
gTvQR1_R4A   cgagaatgga gtaaattgca aatatagcct cca----gta atttcgtaac tagcagaaac
gTvQR1_R6B   cgagaatgga gtaaattgca aatatagcct cca----gta atttcgtaac tagcagaaac
gTvQR1_R6A   cgagaatgga gtaaattgca aatatagcct cca----gta atttcgtaac tagcagaaac
gTvQR1_R5A   cgagaatgga gtaaattgca aatatagcct cca----gta atttcgtaac tagcagaaac
gTvQR1_R5B   cgagaatgga gtaaattgca aatatagcct cca----gta atttcgtaac tagcagaaac
gTvQR1_r8A   cgagaatgga gtaaattgca aatatagcct cca----gta atttcgtaac tagcagaaac
gTvQR1_r8B   cgagaatgga gtaaattgca aatatagcct cca----gta atttcgtaac tagcagaaac
gTvQR1_r5A   ggagaatgga gtaaattgca aatatagcct cca----gta atttcgtaac tagcagaaac
gTvQR1_r5B   ggagaatgga gtaaattgca aatatagcct cca----gta atttcgtaac tagcagaaac
gTvQR1_R2A   ggagaatgga gtaaattgca aatatagcct cca----gta atttcgtaac tagcagaaac
gTvQR1_R2B   ggagaatgga gtaaattgca aatatagcct cca----gta atttcgtaac tagcagaaac
gTvQR1_n1A   ggagaatgga gtaaattgca aatatagcct cca----gta atttcgtaac tagcagaaac
gTvQR1_n1B   ggagaatgga gtaaattgca aatatagcct cca----gta atttcgtaac tagcagaaac
gTvQR1_N2B   ggagaatgga gtaaattgca aatatagcct cca----gta atttcgtaac tagcagaaac
gTvQR1_N2A   ggagaatgga gtaaattgca aatatagcct cca----gta atttcgtaac tagcagaaac
gTvQR1_n2A   ggagaatgga gtaaattgca aatatagcct cca----gta atttcgtaac tagcagaaac
gTvQR1_n2B   ggagaatgga gtaaattgca aatatagcct cca----gta atttcgtaac tagcagaaac
gTvQR1_N3A   cgagaatgga gtaaattgca aatatagcct cca----gta atttcgtaac tagcagaaac
gTvQR1_N3B   cgagaatgga gtaaattgca aatatagcct cca----gta atttcgtaac tagcagaaac
gTvQR1_n3A   ggagaatgga gtaaattgca aatatagcct cca----gta atttcgtaac tagcagaaac
gTvQR1_n3B   ggagaatgga gtaaattgca aatatagcct cca----gta atttcgtaac tagcagaaac
```

```
             841                                                900
gTvQR1_r1A   atggggg--t ttgtgcaagt taagagatta aagaggagat atttgctagt tt--------
gTvQR1_r1B   atggggg--t ttgtgcaagt taagagatta aagaggagat atttgctagt tt--------
gTvQR1_R3A   atggggg--t ttgtgcaagt taagagatta aagaggagat atttgctagt tt--------
gTvQR1_R3B   atggggg--t ttgtgcaagt taagagatta aagaggagat atttgctagt tt--------
gTvQR1_N1A   atgtggggtt ttgtgcaagt taagagatta aacagg-gat atttgctagt tt--------
gTvQR1_N1B   atgtggggtt ttgtgcaagt taagagatta aacagg-gat atttgctagt tt--------
gTvQR1_r4A   atgggggatt ttgtgcaagt t--------- ---------- ---------- -t--------
gTvQR1_r4B   atgggggatt ttgtgcaagt t--------- ---------- ---------- -t--------
gTvQR1_r7A   atggggggtt ttgtgcaagt taagagatta aacaggagac atttgctagt ttgtttttaa
gTvQR1_r7B   atggggggtt ttgtgcaagt taagagatta aacaggagac atttgctagt ttgtttttaa
gTvQR1_r3A   atgggggatt ttgtgcaagt tt-------- ---------- ---------- ----------
gTvQR1_r3B   atgggggatt ttgtgcaagt tt-------- ---------- ---------- ----------
gTvQR1_r6A   atgggggatt ttgtgcaagt tt-------- ---------- ---------- ----------
gTvQR1_r6B   atgggggatt ttgtgcaagt tt-------- ---------- ---------- ----------
gTvQR1_r2A   ttccacgtga aaaaataagt taggataggc ccctagattt agctaatgat gtttagtccc
gTvQR1_r2B   ttccacgtga aaaaataagt taggataggc ccctagattt agctaatgat gtttagtccc
gTvQR1_R1A   ttccacgtga aaaaataagt taggataggc ccctagattt agctaatgat gtttagtccc
gTvQR1_R1B   ttccacgtga aaaaataagt taggataggc ccctagattt agctaatgat gtttagtccc
gTvQR1_R4B   ttccacgtga aaaaataagt taggataggc ccctagattt agctaatgat gtttagtccc
gTvQR1_R4A   ttccacgtga aaaaataagt taggataggc ccctagattt agctaatgat gtttagtccc
gTvQR1_R6B   ttccacgtga aaaaataagt taggataggc ccctagattt agctaatgat gtttagtccc
gTvQR1_R6A   ttccacgtga aaaaataagt taggataggc ccctagattt agctaatgat gtttagtccc
gTvQR1_R5A   ttccacgtga aaaaataagt taggataggc ccctagattt agctaatgat gtttagtccc
gTvQR1_R5B   ttccacgtga aaaaataagt taggataggc ccctagattt agctaatgat gtttagtccc
gTvQR1_r8A   ttccacgtga aaaaataagt taggataggc ccctagattt agctaatgat gtttagtccc
gTvQR1_r8B   ttccacgtga aaaaataagt taggataggc ccctagattt agctaatgat gtttagtccc
gTvQR1_r5A   ttccacgtga aaaaataagt taggataggc ccctagattt agctaatgat gtttagtccc
gTvQR1_r5B   ttccacgtga aaaaataagt taggataggc ccctagattt agctaatgat gtttagtccc
gTvQR1_R2A   ttccacgtga aaaaataagt taggataggc ccctagattt agctaatgat gtttagtccc
gTvQR1_R2B   ttccacgtga aaaaataagt taggataggc ccctagattt agctaatgat gtttagtccc
gTvQR1_n1A   ttccacgtga aaaaataagt taggataggc ccctagattt agctaatgat gtttagtccc
gTvQR1_n1B   ttccacgtga aaaaataagt taggataggc ccctagattt agctaatgat gtttagtccc
gTvQR1_N2B   ttccacgtga aaaaataagt taggataggc ccctagattt agctaatgat gtttagtccc
gTvQR1_N2A   ttccacgtga aaaaataagt taggataggc ccctagattt agctaatgat gtttagtccc
gTvQR1_n2A   ttccacgtga aaaaataagt taggataggc ccctagattt agctaatgat gtttagtccc
gTvQR1_n2B   ttccacgtga aaaaataagt taggataggc ccctagattt agctaatgat gtttagtccc
gTvQR1_N3A   ttccacgtga aaaaataagt taggataggc ccctagattt agctaatgat gtttagtccc
gTvQR1_N3B   ttccacgtga aaaaataagt taggataggc ccctagattt agctaatgat gtttagtccc
gTvQR1_n3A   ttccacgtga aaaaataagt taggataggc ccctagattt agctaatgat gtttagtccc
gTvQR1_n3B   ttccacgtga aaaaataagt taggataggc ccctagattt agctaatgat gtttagtccc
```

```
                901                                                        960
gTvQR1_r1A  cggagttttc tattagttct gaaattacac ------gaga gtgt-atttg caatttactc
gTvQR1_r1B  cggagttttc tattagttct gaaattacac ------gaga gtgt-atttg caatttactc
gTvQR1_R3A  cggagttttc tattagttct gaaattacac ------gaga gtgt-atttg caatttactc
gTvQR1_R3B  cggagttttc tattagttct gaaattacac ------gaga gtgt-atttg caatttactc
gTvQR1_N1A  cggagttttc tattagttct gaaattacac ------gaga ttgt-atttg caatttactc
gTvQR1_N1B  cggagttttc tattagttct gaaattacac ------gaga ttgt-atttg caatttactc
gTvQR1_r4A  cggagttttc tattagtttt gaaattacac ------gaga gtgt-atttg caatttactc
gTvQR1_r4B  cggagttttc tattagtttt gaaattacac ------gaga gtgt-atttg caatttactc
gTvQR1_r7A  cggagttttc tattagttct gaaattacac ------gaga gtgt-atttg caatttactc
gTvQR1_r7B  cggagttttc tattagttct gaaattacac ------gaga gtgt-atttg caatttactc
gTvQR1_r3A  cggagttttc tattagtttt gaaattacag ------gaga gtgt-atttg caatttactc
gTvQR1_r3B  cggagttttc tattagtttt gaaattacag ------gaga gtgt-atttg caatttactc
gTvQR1_r6A  cggagttttc tattagtttt gaaattacag ------gaga gtgt-atttg caatttactc
gTvQR1_r6B  cggagttttc tattagtttt gaaattacag ------gaga gtgt-atttg caatttactc
gTvQR1_r2A  cgtaaatttt agttaccagc acaaggcctc tatatagagg gagttaaatg tcattaaata
gTvQR1_r2B  cgtaaatttt agttaccagc acaaggcctc tatatagagg gagttaaatg tcattaaata
gTvQR1_R1A  cgtaaatttt agttaccagc acaaggcctc tatatagagg gagttaaatg tcattaaata
gTvQR1_R1B  cgtaaatttt agttaccagc acaaggcctc tatatagagg gagttaaatg tcattaaata
gTvQR1_R4B  cgtaaatttt agttaccagc acaaggcctc tatatagagg gagttaaatg tcattaaata
gTvQR1_R4A  cgtaaatttt agttaccagc acaaggcctc tatatagagg gagttaaatg tcattaaata
gTvQR1_R6B  cgtaaatttt agttaccagc acaaggcctc tatatagagg gagttaaatg tcattaaata
gTvQR1_R6A  cgtaaatttt agttaccagc acaaggcctc tatatagagg gagttaaatg tcattaaata
gTvQR1_R5A  cgtaaatttt agttaccagc acaaggcctc tatatagagg gagttaaatg tcattaaata
gTvQR1_R5B  cgtaaatttt agttaccagc acaaggcctc tatatagagg gagttaaatg tcattaaata
gTvQR1_r8A  cgtaaatttt agttaccagc acaaggcctc tatatagagg gagttaaatg tcattaaata
gTvQR1_r8B  cgtaaatttt agttaccagc acaaggcctc tatatagagg gagttaaatg tcattaaata
gTvQR1_r5A  cgtaaatctt agttaccagc acaaggcctc tatatagagg gagttaaatg tcattaaata
gTvQR1_r5B  cgtaaatctt agttaccagc acaaggcctc tatatagagg gagttaaatg tcattaaata
gTvQR1_R2A  cgtaaatctt agttaccagc acaaggcctc tatatagagg gagttaaatg tcattaaata
gTvQR1_R2B  cgtaaatctt agttaccagc acaaggcctc tatatagagg gagttaaatg tcattaaata
gTvQR1_n1A  cgtaaatctt agttaccagc acaaggcctc tatatagagg gagttaaatg tcattaaata
gTvQR1_n1B  cgtaaatctt agttaccagc acaaggcctc tatatagagg gagttaaatg tcattaaata
gTvQR1_N2B  cgtaaatctt agttaccagc acaaggcctc tatatagagg gagttaaatg tcattaaata
gTvQR1_N2A  cgtaaatctt agttaccagc acaaggcctc tatatagagg gagttaaatg tcattaaata
gTvQR1_n2A  cgtaaatctt agttaccagc acaaggcctc tatatagagg gagttaaatg tcattaaata
gTvQR1_n2B  cgtaaatctt agttaccagc acaaggcctc tatatagagg gagttaaatg tcattaaata
gTvQR1_N3A  cgtaaatttt agttaccagc acaaggcctc tatatagagg gagttaaatg tcattaaata
gTvQR1_N3B  cgtaaatttt agttaccagc acaaggcctc tatatagagg gagttaaatg tcattaaata
gTvQR1_n3A  cgtaaatctt agttaccagc acaaggcctc tatatagagg gagttaaatg tcattaaata
gTvQR1_n3B  cgtaaatctt agttaccagc acaaggcctc tatatagagg gagttaaatg tcattaaata
```

```
             961                                                        1020
gTvQR1_r1A   gtaaaaaatt ggttatgctt caaaattaat ta-caaatac gccctagata ttttgtttat
gTvQR1_r1B   gtaaaaaatt ggttatgctt caaaattaat ta-caaatac gccctagata ttttgtttat
gTvQR1_R3A   gtaaaaaatt ggttatgctt caaaattaat ta-caaatac gccctagata ttttgtttat
gTvQR1_R3B   gtaaaaaatt ggttatgctt caaaattaat ta-caaatac gccctagata ttttgtttat
gTvQR1_N1A   gtaaaaagtt ggttatgctt aaaaattaat ta-caaatac gccctagaca ttttgtttat
gTvQR1_N1B   gtaaaaagtt ggttatgctt aaaaattaat ta-caaatac gccctagaca ttttgtttat
gTvQR1_r4A   ataaaaagtt ggttatgctt caaaattaat ta-caaatac gtcctagaca ttttgtttat
gTvQR1_r4B   ataaaaagtt ggttatgctt caaaattaat ta-caaatac gtcctagaca ttttgtttat
gTvQR1_r7A   gtaaaaagtt ggttatgctt cgaaattaat ta-caaatac gccctagaca ttttgtttat
gTvQR1_r7B   gtaaaaagtt ggttatgctt cgaaattaat ta-caaatac gccctagaca ttttgtttat
gTvQR1_r3A   ataaaaagtt ggttatgctt caaaattaat ta-caaatac gccctagaca ttttgtttat
gTvQR1_r3B   ataaaaagtt ggttatgctt caaaattaat ta-caaatac gccctagaca ttttgtttat
gTvQR1_r6A   ataaaaagtt ggttatgctt caaaattaat ta-caaatac gccctagaca ttttgtttat
gTvQR1_r6B   ataaaaagtt ggttatgctt caaaattaat ta-caaatac gccctagaca ttttgtttat
gTvQR1_r2A   attttgtagg ggatattatg ctaatttatt tttcatatga gattttgtgc taattttaaa
gTvQR1_r2B   attttgtagg ggatattatg ctaatttatt tttcatatga gattttgtgc taattttaaa
gTvQR1_R1A   attttgtagg ggatattatg ctaatttatt tttcatatga gattttgtgc taattttaaa
gTvQR1_R1B   attttgtagg ggatattatg ctaatttatt tttcatatga gattttgtgc taattttaaa
gTvQR1_R4B   attttgtagg ggatattatg ctaatttatt tttcatatga gattttgtgc taattttaaa
gTvQR1_R4A   attttgtagg ggatattatg ctaatttatt tttcatatga gattttgtgc taattttaaa
gTvQR1_R6B   attttgtagg ggatattatg ctaatttatt tttcatatga gattttgtgc taattttaaa
gTvQR1_R6A   attttgtagg ggatattatg ctaatttatt tttcatatga gattttgtgc taattttaaa
gTvQR1_R5A   attttgtagg ggatattatg ctaatttatt tttcatatga gattttgtgc taattttaaa
gTvQR1_R5B   attttgtagg ggatattatg ctaatttatt tttcatatga gattttgtgc taattttaaa
gTvQR1_r8A   attttgtagg g-atattatg ctaatttatt tttcatatga gattttgtgc taattttaaa
gTvQR1_r8B   attttgtagg g-atattatg ctaatttatt tttcatatga gattttgtgc taattttaaa
gTvQR1_r5A   attttgtagg ggatattatg ctaatttatt tttcatatga gattttgtgc taattttaaa
gTvQR1_r5B   attttgtagg ggatattatg ctaatttatt tttcatatga gattttgtgc taattttaaa
gTvQR1_R2A   attttgtagg ggatattatg ctaatttatt tttcatatga gattttgtgc taattttaaa
gTvQR1_R2B   attttgtagg ggatattatg ctaatttatt tttcatatga gattttgtgc taattttaaa
gTvQR1_n1A   attttgtagg ggatattatg ctaatttatt tttcatatga gattttgtgc taattttaaa
gTvQR1_n1B   attttgtagg ggatattatg ctaatttatt tttcatatga gattttgtgc taattttaaa
gTvQR1_N2B   attttgtagg ggatattatg ctaatttatt tttcatatga gattttgtgc taattttaaa
gTvQR1_N2A   attttgtagg ggatattatg ctaatttatt tttcatatga gattttgtgc taattttaaa
gTvQR1_n2A   attttgtagg ggatattatg ctaatttatt tttcatatga gattttgtgc taattttaaa
gTvQR1_n2B   attttgtagg ggatattatg ctaatttatt tttcatatga gattttgtgc taattttaaa
gTvQR1_N3A   attttgtagg g-atattatg ctaatttatt tttcatatga gattttgtgc taattttaaa
gTvQR1_N3B   attttgtagg g-atattatg ctaatttatt tttcatatga gattttgtgc taattttaaa
gTvQR1_n3A   attttgtagg ggatattatg ctaatttatt tttcatatga gattttgtgc taattttaaa
gTvQR1_n3B   attttgtagg ggatattatg ctaatttatt tttcatatga gattttgtgc taattttaaa
```

```
             1021                                                           1080
gTvQR1_r1A   tttca----- ----attagt acctaaa--- ---ttacaa- --gcacttgg ctagcc-gga
gTvQR1_r1B   tttca----- ----attagt acctaaa--- ---ttacaa- --gcacttgg ctagcc-gga
gTvQR1_R3A   tttca----- ----attagt acctaaa--- ---ttacaa- --gcacttgg ctagcc-gga
gTvQR1_R3B   tttca----- ----attagt acctaaa--- ---ttacaa- --gcacttgg ctagcc-gga
gTvQR1_N1A   tttca----- ----attagt acctaaa--- ---ttacaa- --gcacttgg ctagcc-gga
gTvQR1_N1B   tttca----- ----attagt acctaaa--- ---ttacaa- --gcacttgg ctagcc-gga
gTvQR1_r4A   tttca----- ----attaat acctaaacct aaattacaa- --gcacttgg ctagcc-gga
gTvQR1_r4B   tttca----- ----attaat acctaaacct aaattacaa- --gcacttgg ctagcc-gga
gTvQR1_r7A   tttca----- ----attagt acctaaa--- ---ttgcaa- --gcacttgg ctagcc-gga
gTvQR1_r7B   tttca----- ----attagt acctaaa--- ---ttgcaa- --gcacttgg ctagcc-gga
gTvQR1_r3A   tttca----- ----attagt acctatacct aaattacaa- --gcacttgg ctagcc-gga
gTvQR1_r3B   tttca----- ----attagt acctatacct aaattacaa- --gcacttgg ctagcc-gga
gTvQR1_r6A   tttca----- ----attagt acctatacct aaattacaa- --gcacttgg ctagcc-gga
gTvQR1_r6B   tttca----- ----attagt acctatacct aaattacaa- --gcacttgg ctagcc-gga
gTvQR1_r2A   ttacatggaa gaatatttgt caaatagctt acttttcact tgggatttg ctagtttggc
gTvQR1_r2B   ttacatggaa gaatatttgt caaatagctt acttttcact tgggatttg ctagtttggc
gTvQR1_R1A   ttacatggaa gaatatttgt caaatagctt acttttcact tgggatttg ctagtttggc
gTvQR1_R1B   ttacatggaa gaatatttgt caaatagctt acttttcact tgggatttg ctagtttggc
gTvQR1_R4B   ttacatggaa gaatatttgt caaatagctt acttttcact tgggatttg ctagtttggc
gTvQR1_R4A   ttacatggaa gaatatttgt caaatagctt acttttcact tgggatttg ctagtttggc
gTvQR1_R6B   ttacatggaa gaatatttgt caaatagctt acttttcact tgggatttg ctagtttggc
gTvQR1_R6A   ttacatggaa gaatatttgt caaatagctt acttttcact tgggatttg ctagtttggc
gTvQR1_R5A   ttacatggaa gaatatttgt caaatagctt acttttcact tgggatttg ctagtttggc
gTvQR1_R5B   ttacatggaa gaatatttgt caaatagctt acttttcact tgggatttg ctagtttggc
gTvQR1_r8A   ttacatggaa gaatatttgt caaatagctt acttttcact tgggatttg ctagtttggc
gTvQR1_r8B   ttacatggaa gaatatttgt caaatagctt acttttcact tgggatttg ctagtttggc
gTvQR1_r5A   ttacatggaa gaatatttgt caaatagctt acttttcact tgggatttg ctagtttggc
gTvQR1_r5B   ttacatggaa gaatatttgt caaatagctt acttttcact tgggatttg ctagtttggc
gTvQR1_R2A   ttacatggaa gaatatttgt caaatagctt acttttcact tgggatttg ctagtttggc
gTvQR1_R2B   ttacatggaa gaatatttgt caaatagctt acttttcact tgggatttg ctagtttggc
gTvQR1_n1A   ttacatggaa gaatatttgt caaatagctt acttttcact tgggatttg ctagtttggc
gTvQR1_n1B   ttacatggaa gaatatttgt caaatagctt acttttcact tgggatttg ctagtttggc
gTvQR1_N2B   ttacatggaa gaatatttgt caaatagctt acttttcact tgggatttg ctagtttggc
gTvQR1_N2A   ttacatggaa gaatatttgt caaatagctt acttttcact tgggatttg ctagtttggc
gTvQR1_n2A   ttacatggaa gaatatttgt caaatagctt acttttcact tgggatttg ctagtttggc
gTvQR1_n2B   ttacatggaa gaatatttgt caaatagctt acttttcact tgggatttg ctagtttggc
gTvQR1_N3A   ttacatggaa gaatatttgt caaatagctt acttttcact tgggatttg ctagtttggc
gTvQR1_N3B   ttacatggaa gaatatttgt caaatagctt acttttcact tgggatttg ctagtttggc
gTvQR1_n3A   ttacatggaa gaatatttgt caaatagctt acttttcact tgggatttg ctagtttggc
gTvQR1_n3B   ttacatggaa gaatatttgt caaatagctt acttttcact tgggatttg ctagtttggc
```

```
             1081                                                       1140
gTvQR1_r1A  ttaatacctc agattat--g agtttgagaa tgac------ ------taat tttgattctg
gTvQR1_r1B  ttaatacctc agattat--g agtttgagaa tgac------ ------taat tttgattctg
gTvQR1_R3A  ttaatacctc agattat--g agtttgagaa tgac------ ------taat tttgattctg
gTvQR1_R3B  ttaatacctc agattat--g agtttgagaa tgac------ ------taat tttgattctg
gTvQR1_N1A  ttaatacctt agattat--g agtttgagaa tgac------ ------taat tttgattctg
gTvQR1_N1B  ttaatacctt agattat--g agtttgagaa tgac------ ------taat tttgattctg
gTvQR1_r4A  ttaatacctt agattat--g agtttaagta tgac------ ------taat tttgattctg
gTvQR1_r4B  ttaatacctt agattat--g agtttaagta tgac------ ------taat tttgattctg
gTvQR1_r7A  ttaatacctt agattat--g agtttgagaa tgac------ ------taat tttgattctg
gTvQR1_r7B  ttaatacctt agattat--g agtttgagaa tgac------ ------taat tttgattctg
gTvQR1_r3A  ttaatacctt agattat--g agtttaagaa tgac------ ------taat tttgattctg
gTvQR1_r3B  ttaatacctt agattat--g agtttaagaa tgac------ ------taat tttgattctg
gTvQR1_r6A  ttaatacctt agattat--g agtttaagaa tgac------ ------taat tttgattctg
gTvQR1_r6B  ttaatacctt agattat--g agtttaagaa tgac------ ------taat tttgattctg
gTvQR1_r2A  tatttacaat ttattatttg agaatgagta attcagtaca cagttgttat tgtaattcta
gTvQR1_r2B  tatttacaat ttattatttg agaatgagta attcagtaca cagttgttat tgtaattcta
gTvQR1_R1A  tatttacaat ttattatttg agaatgagta attcagtaca cagttgttat tgtaattcta
gTvQR1_R1B  tatttacaat ttattatttg agaatgagta attcagtaca cagttgttat tgtaattcta
gTvQR1_R4B  tatttacaat ttattatttg agaatgagta attcagtaca cagttgttat tgtaattcta
gTvQR1_R4A  tatttacaat ttattatttg agaatgagta attcagtaca cagttgttat tgtaattcta
gTvQR1_R6B  tatttacaat ttattatttg agaatgagta attcagtaca cagttgttat tgtaattcta
gTvQR1_R6A  tatttacaat ttattatttg agaatgagta attcagtaca cagttgttat tgtaattcta
gTvQR1_R5A  tatttacaat ttattatttg agaatgagta attcagtaca cagttgttat tgtaattcta
gTvQR1_R5B  tatttacaat ttattatttg agaatgagta attcagtaca cagttgttat tgtaattcta
gTvQR1_r8A  tatttacaat ttattatttg agaatgagta attcagtaca cagttgttat tgtaattcaa
gTvQR1_r8B  tatttacaat ttattatttg agaatgagta attcagtaca cagttgttat tgtaattcaa
gTvQR1_r5A  tatttacaat ttattatttg agaatgagtc attcagtaca cagttgttat tgtaattcta
gTvQR1_r5B  tatttacaat ttattatttg agaatgagtc attcagtaca cagttgttat tgtaattcta
gTvQR1_R2A  tatttacaat ttattatttg agaatgagtc attcagtaca cagttgttat tgtaattcta
gTvQR1_R2B  tatttacaat ttattatttg agaatgagtc attcagtaca cagttgttat tgtaattcta
gTvQR1_n1A  tatttacaat ttattatttg agaatgagtc attcagtaca cagttgttat tgtaattcta
gTvQR1_n1B  tatttacaat ttattatttg agaatgagtc attcagtaca cagttgttat tgtaattcta
gTvQR1_N2B  tatttacaat ttattatttg agaatgagtc attcagtaca cagttgttat tgtaattcta
gTvQR1_N2A  tatttacaat ttattattcg agaatgagtc attcagtaca cagttgttat tgtaattcta
gTvQR1_n2A  tatttacaat ttattatttg agaatgagtc attcagtaca cagttgttat tgtaattcta
gTvQR1_n2B  tatttacaat ttattatttg agaatgagtc attcagtaca cagttgttat tgtaattcta
gTvQR1_N3A  tatttacaat ttattatttg agaatgagta attcagtaca cagttgttat tgtaattcaa
gTvQR1_N3B  tatttacaat ttattatttg agaatgagta attcagtaca cagttgttat tgtaattcaa
gTvQR1_n3A  tatttacaat ttattatttg agaatgagtc attcagtaca cagttgttat tgtaattcta
gTvQR1_n3B  tatttacaat ttattatttg agaatgagtc attcagtaca cagttgttat tgtaattcta
```

```
             1141                                                    1200
gTvQR1_r1A   aacgataacg ggagtttctt gtaattaggc taaaccttag gtgaactttg tgtaattaat
gTvQR1_r1B   aacgataacg ggagtttctt gtaattaggc taaaccttag gtgaactttg tgtaattaat
gTvQR1_R3A   aacgataacg ggagtttctt gtaattaggc taaaccttag gtgaactttg tgtaattaat
gTvQR1_R3B   aacgataacg ggagtttctt gtaattaggc taaaccttag gtgaactttg tgtaattaat
gTvQR1_N1A   aacgataacg ggagtttctt gtaattaggc taaaccttag gtgaactttg tgtaatta-t
gTvQR1_N1B   aacgataacg ggagtttctt gtaattaggc taaaccttag gtgaactttg tgtaatta-t
gTvQR1_r4A   aacgataacg ggagtttctt gtaattaggc taaaccttag gtgaactttg tgtaatta-t
gTvQR1_r4B   aacgataacg ggagtttctt gtaattaggc taaaccttag gtgaactttg tgtaatta-t
gTvQR1_r7A   aacgatgacg ggagtttctt gtaattaggc taaaccttag gtgaactttg tgtaatta-g
gTvQR1_r7B   aacgatgacg ggagtttctt gtaattaggc taaaccttag gtgaactttg tgtaatta-g
gTvQR1_r3A   aacgataacg ggagtttctt gtaattaggc taaaccttag gtgaactttg tgtaatta-t
gTvQR1_r3B   aacgataacg ggagtttctt gtaattaggc taaaccttag gtgaactttg tgtaatta-t
gTvQR1_r6A   aacgataacg ggagtttctt gtaattaggc taaaccttag gtgaactttg tgtaatta-t
gTvQR1_r6B   aacgataacg ggagtttctt gtaattaggc taaaccttag gtgaactttg tgtaatta-t
gTvQR1_r2A   aaagacaaat gacatttctt gtaattaggc taaaccttag gtgaactttg tgttattaat
gTvQR1_r2B   aaagacaaat gacatttctt gtaattaggc taaaccttag gtgaactttg tgttattaat
gTvQR1_R1A   aaagacaaat gacatttctt gtaattaggc taaaccttag gtgaactttg tgttattaat
gTvQR1_R1B   aaagacaaat gacatttctt gtaattaggc taaaccttag gtgaactttg tgttattaat
gTvQR1_R4B   aaagacaaat gacatttctt gtaattaggc taaaccttag gtgaactttg tgttattaat
gTvQR1_R4A   aaagacaaat gacatttctt gtaattaggc taaaccttag gtgaactttg tgttattaat
gTvQR1_R6B   aaagacaaat gacatttctt gtaattaggc taaaccttag gtgaactttg tgttattaat
gTvQR1_R6A   aaagacaaat gacatttctt gtaattaggc taaaccttag gtgaactttg tgttattaat
gTvQR1_R5A   aaagacaaat gacatttctt gtaattaggc taaaccttag gtgaactttg tgttattaat
gTvQR1_R5B   aaagacaaat gacatttctt gtaattaggc taaaccttag gtgaactttg tgttattaat
gTvQR1_r8A   aacgacaaat gaagtttctt gtaattaagc taaacattag gtgaagtttg tgtaattaat
gTvQR1_r8B   aacgacaaat gaagtttctt gtaattaagc taaacattag gtgaagtttg tgtaattaat
gTvQR1_r5A   aaagacaaat gacatttctt gtaattaggc taaaccttag gtgaactttg tgttattaat
gTvQR1_r5B   aaagacaaat gacatttctt gtaattaggc taaaccttag gtgaactttg tgttattaat
gTvQR1_R2A   aaagacaaat gacatttctt gtaattaggc taaaccttag gtgaactttg tgttattaat
gTvQR1_R2B   aaagacaaat gacatttctt gtaattaggc taaaccttag gtgaactttg tgttattaat
gTvQR1_n1A   aaagacaaat gacatttctt gtaattaggc taaaccttag gtgaactttg tgttattaat
gTvQR1_n1B   aaagacaaat gacatttctt gtaattaggc taaaccttag gtgaactttg tgttattaat
gTvQR1_N2B   aaagacaaat gacatttctt gtaattaggc taaaccttag gtgaactttg tgttattaat
gTvQR1_N2A   aaagacaaat gacatttctt gtaattaggc taaaccttag gtgaactttg tgttattaat
gTvQR1_n2A   aaagacaaat gacatttctt gtaattaggc taaaccttag gtgaactttg tgttattaat
gTvQR1_n2B   aaagacaaat gacatttctt gtaattaggc taaaccttag gtgaactttg tgttattaat
gTvQR1_N3A   aacgacaaat gaagtttctt gtaattaagc taaacattag gtgaagtttg tgtaattaat
gTvQR1_N3B   aacgacaaat gaagtttctt gtaattaagc taaacattag gtgaagtttg tgtaattaat
gTvQR1_n3A   aaagacaaat gacatttctt gtaattaggc taaaccttag gtgaactttg tgttattaat
gTvQR1_n3B   aaagacaaat gacatttctt gtaattaggc taaaccttag gtgaactttg tgttattaat
```

```
               1201                                                       1260
gTvQR1_r1A  ccatatttca tatagcctta cacgaagttt aattaattcc aaaa-tggtc caactacttt
gTvQR1_r1B  ccatatttca tatagcctta cacgaagttt aattaattcc aaaa-tggtc caactacttt
gTvQR1_R3A  ccatatttca tatagcctta cacgaagttt aattaattcc aaaa-tggtc caactacttt
gTvQR1_R3B  ccatatttca tatagcctta cacgaagttt aattaattcc aaaa-tggtc caactacttt
gTvQR1_N1A  acatatttta tatagcctta cacgaacttt aattaattcc aaaaatggtc caactacttt
gTvQR1_N1B  acatatttta tatagcctta cacgaacttt aattaattcc aaaaatggtc caactacttt
gTvQR1_r4A  acatatttta tatagcctta cacgaagttt aattaattcc aaaa-tggtc caactacttt
gTvQR1_r4B  acatatttta tatagcctta cacgaagttt aattaattcc aaaa-tggtc caactacttt
gTvQR1_r7A  acatatttta tatagcctta cacgaagttt aattaattcc aaaa-tggtc caactacttt
gTvQR1_r7B  acatatttta tatagcctta cacgaagttt aattaattcc aaaa-tggtc caactacttt
gTvQR1_r3A  acatatttta tatagcctta cacgaagttt aattaattcc aaaa-tggtc caactacttt
gTvQR1_r3B  acatatttta tatagcctta cacgaagttt aattaattcc aaaa-tggtc caactacttt
gTvQR1_r6A  acatatttta tatagcctta cacgaagttt aattaattcc aaaa-tggtc caactacttt
gTvQR1_r6B  acatatttta tatagcctta cacgaagttt aattaattcc aaaa-tggtc caactacttt
gTvQR1_r2A  cgatatttca tatagcctta cacgaagttt aattaattcc aaaa-tggtc caactaaatc
gTvQR1_r2B  cgatatttca tatagcctta cacgaagttt aattaattcc aaaa-tggtc caactaaatc
gTvQR1_R1A  cgatatttca tatagcctta cacgaagttt aattaattcc aaaa-tggtc caactaaatc
gTvQR1_R1B  cgatatttca tatagcctta cacgaagttt aattaattcc aaaa-tggtc caactaaatc
gTvQR1_R4B  cgatatttca tatagcctta cacgaagttt aattaattcc aaaa-tggtc caactaaatc
gTvQR1_R4A  cgatatttca tatagcctta cacgaagttt aattaattcc aaaa-tggtc caactaaatc
gTvQR1_R6B  cgatatttca tatagcctta cacgaagttt aattaattcc aaaa-tggtc caactaaatc
gTvQR1_R6A  cgatatttca tatagcctta cacgaagttt aattaattcc aaaa-tggtc caactaaatc
gTvQR1_R5A  cgatatttca tatagcctta cacgaagttt aattaattcc aaaa-tggtc caactaaatc
gTvQR1_R5B  cgatatttca tatagcctta cacgaagttt aattaattcc aaaa-tggtc caactaaatc
gTvQR1_r8A  ccatatttca tatagcctta cacgaagttt aattaatttc aaaa-tggtc caactaattc
gTvQR1_r8B  ccatatttca tatagcctta cacgaagttt aattaatttc aaaa-tggtc caactaattc
gTvQR1_r5A  cgatatttca tatagcctta cacgaagttt aattaattcc aaaa-tggtc caactaaatc
gTvQR1_r5B  cgatatttca tatagcctta cacgaagttt aattaattcc aaaa-tggtc caactaaatc
gTvQR1_R2A  cgatatttca tatagcctta cacgaagttt aattaattcc aaaa-tggtc caactaaatc
gTvQR1_R2B  cgatatttca tatagcctta cacgaagttt aattaattcc aaaa-tggtc caactaaatc
gTvQR1_n1A  cgatatttca tatagcctta cacgaagttt aattaattcc aaaa-tggtc caactaaatc
gTvQR1_n1B  cgatatttca tatagcctta cacgaagttt aattaattcc aaaa-tggtc caactaaatc
gTvQR1_N2B  cgatatttca tatagcctta cacgaagttt aattaattcc aaaa-tggtc caactaaatc
gTvQR1_N2A  cgatatttca tatagcctta cacgaagttt aattaattcc aaaa-tggtc caactaaatc
gTvQR1_n2A  cgatatttca tatagcctta cacgaagttt aattaattcc aaaa-tggtc caactaaatc
gTvQR1_n2B  cgatatttca tatagcctta cacgaagttt aattaattcc aaaa-tggtc caactaaatc
gTvQR1_N3A  ccatatttca tatagcctta cacgaagttt aattaatttc aaaa-tggtc caactaattc
gTvQR1_N3B  ccatatttca tatagcctta cacgaagttt aattaatttc aaaa-tggtc caactaattc
gTvQR1_n3A  cgatatttca tatagcctta cacgaagttt aattaattcc aaaa-tggtc caactaaatc
gTvQR1_n3B  cgatatttca tatagcctta cacgaagttt aattaattcc aaaa-tggtc caactaaatc
```

```
              1261                                                        1320
gTvQR1_r1A    ttaccaaaac agAGAGGAGG TGGTTTGGCC GAATACGGAG TAGCAAGTGA AAAGCTAACA
gTvQR1_r1B    ttaccaaaac agAGAGGAGG TGGTTTGGCC GAATACGGAG TAGCAAGTGA AAAGCTAACA
gTvQR1_R3A    ttaccaaaac agAGAGGAGG TGGTTTGGCC GAATACGGAG TAGCAAGTGA AAAGCTAACA
gTvQR1_R3B    ttaccaaaac agAGAGGAGG TGGTTTGGCC GAATACGGAG TAGCAAGTGA AAAGCTAACA
gTvQR1_N1A    ttaccaaaac agAGAGGAGG TGGCTTAGCC GAATACGGCG TAGCAAGTGA AAAATTAACA
gTvQR1_N1B    ttaccaaaac agAGAGGAGG TGGCTTAGCC GAATACGGCG TAGCAAGTGA AAAATTAACA
gTvQR1_r4A    ttaccaaaac agAGAGGAGG TGGTTTGGCC GAATACGGCG TAGCAAGTGA AAAGCTAACA
gTvQR1_r4B    ttaccaaaac agAGAGGAGG TGGTTTGGCC GAATACGGCG TAGCAAGTGA AAAGCTAACA
gTvQR1_r7A    ttaccaaaac agAGAGGAGG TGGTTTGGCC GAATACGGAG TAGCAAGTGA AAAGCTAACA
gTvQR1_r7B    ttaccaaaac agAGAGGAGG TGGTTTGGCC GAATACGGAG TAGCAAGTGA AAAGCTAACA
gTvQR1_r3A    ttaccaaaac agAGAGGAGG TGGTTTGGCC GAATACGGAG TAGCAAGTGA AAAGCTAACA
gTvQR1_r3B    ttaccaaaac agAGAGGAGG TGGTTTGGCC GAATACGGAG TAGCAAGTGA AAAGCTAACA
gTvQR1_r6A    ttaccaaaac agAGAGGAGG TGGTTTGGCC GAATACGGAG TAGCAAGTGA AAAGCTAACA
gTvQR1_r6B    ttaccaaaac agAGAGGAGG TGGTTTGGCC GAATACGGAG TAGCAAGTGA AAAGCTAACA
gTvQR1_r2A    ttaccaaaac agGGAGGAGG TGGTTTGGCC GAATACGGCA TAGCAAGTGC AAAATTAACA
gTvQR1_r2B    ttaccaaaac agGGAGGAGG TGGTTTGGCC GAATACGGCA TAGCAAGTGC AAAATTAACA
gTvQR1_R1A    ttaccaaaac agGGAGGAGG TGGTTTGGCC GAATACGGCA TAGCAAGTGC AAAATTAACA
gTvQR1_R1B    ttaccaaaac agGGAGGAGG TGGTTTGGCC GAATACGGCA TAGCAAGTGC AAAATTAACA
gTvQR1_R4B    ttaccaaaac agGGAGGAGG TGGTTTGGCC GAATACGGCA TAGCAAGTGC AAAATTAACA
gTvQR1_R4A    ttaccaaaac agGGAGGAGG TGGTTTGGCC GAATACGGCA TAGCAAGTGC AAAATTAACA
gTvQR1_R6B    ttaccaaaac agGGAGGAGG TGGTTTGGCC GAATACGGCA TAGCAAGTGC AAAATTAACA
gTvQR1_R6A    ttaccaaaac agGGAGGAGG TGGTTTGGCC GAATACGGCA TAGCAAGTGC AAAATTAACA
gTvQR1_R5A    ttaccaaaac agGGAGGAGG TGGTTTGGCC GAATACGGCA TAGCAAGTGC AAAATTAACA
gTvQR1_R5B    ttaccaaaac agGGAGGAGG TGGTTTGGCC GAATACGGCA TAGCAAGTGC AAAATTAACA
gTvQR1_r8A    ttaccaaaac agGGAGGAGG TGGCTTAGCC GAATACGGCG TAGCAAGTGA AAAATTAACA
gTvQR1_r8B    ttaccaaaac agGGAGGAGG TGGCTTAGCC GAATACGGCG TAGCAAGTGA AAAATTAACA
gTvQR1_r5A    ttaccaaaac agGGAGGAGG TGGCTTAGCC GAATACGGCG TAGCAAGTGA AAAGCTAACA
gTvQR1_r5B    ttaccaaaac agGGAGGAGG TGGCTTAGCC GAATACGGCG TAGCAAGTGA AAAGCTAACA
gTvQR1_R2A    ttaccaaaac agGGAGGAGG TGGCTTAGCC GAATACGGCG TAGCAAGTGA AAAGCTAACA
gTvQR1_R2B    ttaccaaaac agGGAGGAGG TGGCTTAGCC GAATACGGCG TAGCAAGTGA AAAGCTAACA
gTvQR1_n1A    ttaccaaaac agGGAGGAGG TGGCTTAGCC GAATACGGCG TAGCAAGTGA AAAGCTAACA
gTvQR1_n1B    ttaccaaaac agGGAGGAGG TGGCTTAGCC GAATACGGCG TAGCAAGTGA AAAGCTAACA
gTvQR1_N2B    ttaccaaaac agGGAGGAGG TGGCTTAGCC GAATACGGCG TAGCAAGTGA AAAGCTAACA
gTvQR1_N2A    ttaccaaaac agGGAGGAGG TGGCTTAGCC GAATACGGCG TAGCAAGTGA AAAGCTAACA
gTvQR1_n2A    ttaccaaaac agGGAGGAGG TGGCTTAGCC GAATACGGCG TAGCAAGTGA AAAGCTAACA
gTvQR1_n2B    ttaccaaaac agGGAGGAGG TGGCTTAGCC GAATACGGCG TAGCAAGTGA AAAGCTAACA
gTvQR1_N3A    ttaccaaaac agGGAGGAGG TGGCTTAGCC GAATACGGCG TAGCAAGTGA AAAGCTAACA
gTvQR1_N3B    ttaccaaaac agGGAGGAGG TGGCTTAGCC GAATACGGCG TAGCAAGTGA AAAGCTAACA
gTvQR1_n3A    ttaccaaaac agGGAGGAGG TGGCTTAGCC GAATACGGCG TAGCAAGTGA AAAGCTAACA
gTvQR1_n3B    ttaccaaaac agGGAGGAGG TGGCTTAGCC GAATACGGCG TAGCAAGTGA AAAGCTAACA
```

```
             1321                                                       1380
gTvQR1_r1A   GTCCACAGGC CACCCGAGGT CTCAGCTGCC GAGAGTTCCG GCCTTCCCAT TGCCGGCCTT
gTvQR1_r1B   GTCCACAGGC CACCCGAGGT CTCAGCTGCC GAGAGTTCCG GCCTTCCCAT TGCCGGCCTT
gTvQR1_R3A   GTCCACAGGC CACCCGAGGT CTCAGCTGCC GAGAGTTCCG GCCTTCCCAT TGCCGGCCTT
gTvQR1_R3B   GTCCACAGGC CACCCGAGGT CTCAGCTGCC GAGAGTTCCG GCCTTCCCAT TGCCGGCCTT
gTvQR1_N1A   GTTCACAGGC CGCCCGAGGT CTCAGCTGCA GAGAGCTCGG GCCTTCCTAT TGCCGGCCTT
gTvQR1_N1B   GTTCACAGGC CGCCCGAGGT CTCAGCTGCA GAGAGCTCGG GCCTTCCTAT TGCCGGCCTT
gTvQR1_r4A   GTCCACAGGC CGCCCGAGGT CTCAGCTGCC GAGAGTTCCG GCCTTCCCAT CGCCGGCCTT
gTvQR1_r4B   GTCCACAGGC CGCCCGAGGT CTCAGCTGCC GAGAGTTCCG GCCTTCCCAT CGCCGGCCTT
gTvQR1_r7A   GTCCATAGGC CGCCCGAGGT CTCAGCTGCC GAGAGTTCCG GCCTTCCCAT TGCCGGCCTT
gTvQR1_r7B   GTCCATAGGC CGCCCGAGGT CTCAGCTGCC GAGAGTTCCG GCCTTCCCAT TGCCGGCCTT
gTvQR1_r3A   GTCCACAGAC CACCCGAGGT CTCAGCTGCC GAGAGTTCCG GCCTTCCCAT TGCCGGCCTT
gTvQR1_r3B   GTCCACAGAC CACCCGAGGT CTCAGCTGCC GAGAGTTCCG GCCTTCCCAT TGCCGGCCTT
gTvQR1_r6A   GTCCACAGAC CACCCGAGGT CTCAGCTGCC GAGAGTTCCG GCCTTCCCAT TGCCGGCCTT
gTvQR1_r6B   GTCCACAGAC CACCCGAGGT CTCAGCTGCC GAGAGTTCCG GCCTTCCCAT TGCCGGCCTT
gTvQR1_r2A   GTTCACAGGC CACCCGAGGT CTCAGCTGCC GAGAGTTCCG GCCTTCCCAT TGCCGGCCTT
gTvQR1_r2B   GTTCACAGGC CACCCGAGGT CTCAGCTGCC GAGAGTTCCG GCCTTCCCAT TGCCGGCCTT
gTvQR1_R1A   GTTCACAGGC CACCCGAGGT CTCAGCTGCC GAGAGTTCCG GCCTTCCCAT TGCCGGCCTT
gTvQR1_R1B   GTTCACAGGC CACCCGAGGT CTCAGCTGCC GAGAGTTCCG GCCTTCCCAT TGCCGGCCTT
gTvQR1_R4B   GTTCACAGGC CACCCGAGGT CTCAGCTGCC GAGAGTTCCG GCCTTCCCAT TGCCGGCCTT
gTvQR1_R4A   GTTCACAGGC CACCCGAGGT CTCAGCTGCC GAGAGTTCCG GCCTTCCCAT TGCCGGCCTT
gTvQR1_R6B   GTTCACAGGC CACCCGAGGT CTCAGCTGCC GAGAGTTCCG GCCTTCCCAT TGCCGGCCTT
gTvQR1_R6A   GTTCACAGGC CACCCGAGGT CTCAGCTGCC GAGAGTTCCG GCCTTCCCAT TGCCGGCCTT
gTvQR1_R5A   GTTCACAGGC CACCCGAGGT CTCAGCTGCC GAGAGTTCCG GCCTTCCCAT TGCCGGCCTT
gTvQR1_R5B   GTTCACAGGC CACCCGAGGT CTCAGCTGCC GAGAGTTCCG GCCTTCCCAT TGCCGGCCTT
gTvQR1_r8A   GTTCACAGGC CGCCCGAGGT CTCAGCTGCC GAGAGTTCCG GCCTTCCCAT TGCTGGCCTC
gTvQR1_r8B   GTTCACAGGC CGCCCGAGGT CTCAGCTGCC GAGAGTTCCG GCCTTCCCAT TGCTGGCCTC
gTvQR1_r5A   GTCCATAGGC CGCCCGAGGT ATCAGCTGCC GAGAGCTCAG GCCTTCCCAT TGCCGGCCTT
gTvQR1_r5B   GTCCATAGGC CGCCCGAGGT ATCAGCTGCC GAGAGCTCAG GCCTTCCCAT TGCCGGCCTT
gTvQR1_R2A   GTCCATAGGC CGCCCGAGGT ATCAGCTGCC GAGAGCTCAG GCCTTCCCAT TGCCGGCCTT
gTvQR1_R2B   GTCCATAGGC CGCCCGAGGT ATCAGCTGCC GAGAGCTCAG GCCTTCCCAT TGCCGGCCTT
gTvQR1_n1A   GTCCATAGGC CGCCCGAGGT ATCAGCTGCC GAGAGCTCAG GCCTTCCCAT TGCCGGCCTT
gTvQR1_n1B   GTCCATAGGC CGCCCGAGGT ATCAGCTGCC GAGAGCTCAG GCCTTCCCAT TGCCGGCCTT
gTvQR1_N2B   GTCCATAGGC CGCCCGAGGT ATCAGCTGCC GAGAGCTCAG GCCTTCCCAT TGCCGGCCTT
gTvQR1_N2A   GTCCATAGGC CGCCCGAGGT ATCAGCTGCC GAGAGCTCAG GCCTTCCCAT TGCCGGCCTT
gTvQR1_n2A   GTCCATAGGC CGCCCGAGGT ATCAGCTGCC GAGAGCTCAG GCCTTCCCAT TGCCGGCCTT
gTvQR1_n2B   GTCCATAGGC CGCCCGAGGT ATCAGCTGCC GAGAGCTCAG GCCTTCCCAT TGCCGGCCTT
gTvQR1_N3A   GTCCATAGGC CGCCCGAGGT ATCAGCTGCC GAGAGCTCAG GCCTTCCCAT TGCCGGCCTT
gTvQR1_N3B   GTCCATAGGC CGCCCGAGGT ATCAGCTGCC GAGAGCTCAG GCCTTCCCAT TGCCGGCCTT
gTvQR1_n3A   GTCCATAGGC CGCCCGAGGT CTCAGCTGCC GAGAGTTCCG GCCTTCCCAT TGCCGGCCTT
gTvQR1_n3B   GTCCATAGGC CGCCCGAGGT CTCAGCTGCC GAGAGTTCCG GCCTTCCCAT TGCCGGCCTT
```

```
             1381                                                       1440
gTvQR1_r1A   ACTGCCCACA TGGCCCTCAC CCAACACATC GGCCTAAACC TCGACAAAAG TGGGCCCCAC
gTvQR1_r1B   ACTGCCCACA TGGCCCTCAC CCAACACATC GGCCTAAACC TCGACAAAAG TGGGCCCCAC
gTvQR1_R3A   ACTGCCCACA TGGCCCTCAC CCAACACATC GGCCTAAACC TCGACAAAAG TGGGCCCCAC
gTvQR1_R3B   ACTGCCCACA TGGCCCTCAC CCAACACATC GGCCTAAACC TCGACAAAAG TGGGCCCCAC
gTvQR1_N1A   ACGGCCCACA TGGCCCTAAC CCAACACATC GGCCTAAACC TCGACAAAAG TGGTCCCCAC
gTvQR1_N1B   ACGGCCCACA TGGCCCTAAC CCAACACATC GGCCTAAACC TCGACAAAAG TGGTCCCCAC
gTvQR1_r4A   ACGGCCCACA TGGCCCTCAC CCAACACATC GGCCTAAACC TCGACAAAAG TGGACCCCAC
gTvQR1_r4B   ACGGCCCACA TGGCCCTCAC CCAACACATC GGCCTAAACC TCGACAAAAG TGGACCCCAC
gTvQR1_r7A   ACAGCCCACA TGGCCCTCAC CCAACACATC GGCCTAAGCC TCGACAAAAG TGGGCCCCAC
gTvQR1_r7B   ACAGCCCACA TGGCCCTCAC CCAACACATC GGCCTAAGCC TCGACAAAAG TGGGCCCCAC
gTvQR1_r3A   ACAGCCCACA TGGCCCTCAC CCAACACATC GGCCTAAACC TCGACAAAAG CGGGCCCCAC
gTvQR1_r3B   ACAGCCCACA TGGCCCTCAC CCAACACATC GGCCTAAACC TCGACAAAAG CGGGCCCCAC
gTvQR1_r6A   ACAGCCCACA TGGCCCTCAC CCAACACATC GGCCTAAACC TCGACAAAAG TGGCCCCCAC
gTvQR1_r6B   ACAGCCCACA TGGCCCTCAC CCAACACATC GGCCTAAACC TCGACAAAAG TGGCCCCCAC
gTvQR1_r2A   ACAGCCCACA TGGCCCTCAC CCAACACATC GGCCTAAACC TCGACAAAAG TGGGCCCCAC
gTvQR1_r2B   ACAGCCCACA TGGCCCTCAC CCAACACATC GGCCTAAACC TCGACAAAAG TGGGCCCCAC
gTvQR1_R1A   ACAGCCCACA TGGCCCTCAC CCAACACATC GGCCTAAACC TCGACAAAAG TGGGCCCCAC
gTvQR1_R1B   ACAGCCCACA TGGCCCTCAC CCAACACATC GGCCTAAACC TCGACAAAAG TGGGCCCCAC
gTvQR1_R4B   ACAGCCCACA TGGCCCTCAC CCAACACATC GGCCTAAACC TCGACAAAAG TGGGCCCCAC
gTvQR1_R4A   ACAGCCCACA TGGCCCTCAC CCAACACATC GGCCTAAACC TCGACAAAAG TGGGCCCCAC
gTvQR1_R6B   ACAGCCCACA TGGCCCTCAC CCAACACATC GGCCTAAACC TCGACAAAAG TGGGCCCCAC
gTvQR1_R6A   ACAGCCCACA TGGCCCTCAC CCAACACATC GGCCTAAACC TCGACAAAAG TGGGCCCCAC
gTvQR1_R5A   ACAGCCCACA TGGCCCTCAC CCAACACATC GGCCTAAACC TCGACAAAAG TGGGCCCCAC
gTvQR1_R5B   ACAGCCCACA TGGCCCTCAC CCAACACATC GGCCTAAACC TCGACAAAAG TGGGCCCCAC
gTvQR1_r8A   ACAGCCCACA TGGCCCTCAC CCAACACATC GGCCTAAACC TCGACAAAAG TGGGCCCCAC
gTvQR1_r8B   ACAGCCCACA TGGCCCTCAC CCAACACATC GGCCTAAACC TCGACAAAAG TGGGCCCCAC
gTvQR1_r5A   ACAGCCCACA TGGCCCTAAC CCAACACATT GGCCTAAACC TCGACAAAAG TGGTCCCCAC
gTvQR1_r5B   ACAGCCCACA TGGCCCTAAC CCAACACATT GGCCTAAACC TCGACAAAAG TGGTCCCCAC
gTvQR1_R2A   ACAGCCCACA TGGCCCTAAC CCAACACATT GGCCTAAACC TCGACAAAAG TGGTCCCCAC
gTvQR1_R2B   ACAGCCCACA TGGCCCTAAC CCAACACATT GGCCTAAACC TCGACAAAAG TGGTCCCCAC
gTvQR1_n1A   ACAGCCCACA TGGCCCTAAC CCAACACATT GGCCTAAACC TCGACAAAAG TGGTCCCCAC
gTvQR1_n1B   ACAGCCCACA TGGCCCTAAC CCAACACATT GGCCTAAACC TCGACAAAAG TGGTCCCCAC
gTvQR1_N2B   ACAGCCCACA TGGCCCTAAC CCAACACATT GGCCTAAACC TCGACAAAAG TGGTCCCCAC
gTvQR1_N2A   ACAGCCCACA TGGCCCTAAC CCAACACATT GGCCTAAACC TCGACAAAAG TGGTCCCCAC
gTvQR1_n2A   ACAGCCCACA TGGCCCTAAC CCAACACATT GGCCTAAACC TCGACAAAAG TGGTCCCCAC
gTvQR1_n2B   ACAGCCCACA TGGCCCTAAC CCAACACATT GGCCTAAACC TCGACAAAAG TGGTCCCCAC
gTvQR1_N3A   ACAGCCCACA TGGCCCTAAC CCAACACATT GGCCTAAACC TCGACAAAAG TGGTCCCCAC
gTvQR1_N3B   ACAGCCCACA TGGCCCTAAC CCAACACATT GGCCTAAACC TCGACAAAAG TGGTCCCCAC
gTvQR1_n3A   ACGGCCCACA TGGCCCTAAC CCAACACATC GGCCTAAACC TCGACAAAAG TGGGCCCCAC
gTvQR1_n3B   ACGGCCCACA TGGCCCTAAC CCAACACATC GGCCTAAACC TCGACAAAAG TGGGCCCCAC
```

```
            1441                                                        1500
gTvQR1_r1A  AAAAACATCC TCATCACAGC CGCCTCCGGT GGCGTTGGCC AATACGCCGT ACAGCTCGCA
gTvQR1_r1B  AAAAACATCC TCATCACAGC CGCCTCCGGT GGCGTTGGCC AATACGCCGT ACAGCTCGCA
gTvQR1_R3A  AAAAACATCC TCATCACAGC CGCCTCCGGT GGCGTTGGCC AATACGCCGT ACAGCTCGCA
gTvQR1_R3B  AAAAACATCC TCATCACAGC CGCCTCCGGT GGCGTTGGCC AATACGCCGT ACAGCTCGCA
gTvQR1_N1A  AAAAACATCC TCATCACAGC CGCCTCCGGT GGCGTTGGCC AATACGCCGT TCAGCTCGCA
gTvQR1_N1B  AAAAACATCC TCATCACAGC CGCCTCCGGT GGCGTTGGCC AATACGCCGT TCAGCTCGCA
gTvQR1_r4A  AAAAACATCC TCATCACAGC CGCCTCCGGC GGCGTTGGCC AATACGCCGT TCAGCTCGCA
gTvQR1_r4B  AAAAACATCC TCATCACAGC CGCCTCCGGC GGCGTTGGCC AATACGCCGT TCAGCTCGCA
gTvQR1_r7A  AAAAACATCC TCATCACAGC CGCCTCCGGT GGCGTTGGCC AATACGCCGT TCAGCTCGCA
gTvQR1_r7B  AAAAACATCC TCATCACAGC CGCCTCCGGT GGCGTTGGCC AATACGCCGT TCAGCTCGCA
gTvQR1_r3A  AAAAACATCC TCATCACAGC CGCCTCCGGT GGCGTTGGCC AATACGCCGT ACAGCTCGCA
gTvQR1_r3B  AAAAACATCC TCATCACAGC CGCCTCCGGT GGCGTTGGCC AATACGCCGT ACAGCTCGCA
gTvQR1_r6A  AAAAACATCC TCATCACAGC CGCCTCCGGT GGCGTTGGCC AATACGCCGT ACAGCTCGCA
gTvQR1_r6B  AAAAACATCC TCATCACAGC CGCCTCCGGT GGCGTTGGCC AATACGCCGT ACAGCTCGCA
gTvQR1_r2A  AAAAACATCC TCATCACAGC CGCCTCCGGT GGTGTTGGCC AATACGCCGT ACAGCTCGCA
gTvQR1_r2B  AAAAACATCC TCATCACAGC CGCCTCCGGT GGTGTTGGCC AATACGCCGT ACAGCTCGCA
gTvQR1_R1A  AAAAACATCC TCATCACAGC CGCCTCCGGT GGTGTTGGCC AATACGCCGT ACAGCTCGCA
gTvQR1_R1B  AAAAACATCC TCATCACAGC CGCCTCCGGT GGTGTTGGCC AATACGCCGT ACAGCTCGCA
gTvQR1_R4B  AAAAACATCC TCATCACAGC CGCCTCCGGT GGTGTTGGCC AATACGCCGT ACAGCTCGCA
gTvQR1_R4A  AAAAACATCC TCATCACAGC CGCCTCCGGT GGTGTTGGCC AATACGCCGT ACAGCTCGCA
gTvQR1_R6B  AAAAACATCC TCATCACAGC CGCCTCCGGT GGTGTTGGCC AATACGCCGT ACAGCTCGCA
gTvQR1_R6A  AAAAACATCC TCATCACAGC CGCCTCCGGT GGTGTTGGCC AATACGCCGT ACAGCTCGCA
gTvQR1_R5A  AAAAACATCC TCATCACAGC CGCCTCCGGT GGTGTTGGCC AATACGCCGT ACAGCTCGCA
gTvQR1_R5B  AAAAACATCC TCATCACAGC CGCCTCCGGT GGTGTTGGCC AATACGCCGT ACAGCTCGCA
gTvQR1_r8A  AAAAACATCC TCATCACAGC CGCCTCCGGT GGCGTCGGCC AATACGCCAT TCAGCTCGCA
gTvQR1_r8B  AAAAACATCC TCATCACAGC CGCCTCCGGT GGCGTCGGCC AATACGCCAT TCAGCTCGCA
gTvQR1_r5A  AAAAACATCC TCATCACAGC CGCCTCCGGT GGTGTTGGCC AATACGCCGT ACAGCTCGCA
gTvQR1_r5B  AAAAACATCC TCATCACAGC CGCCTCCGGT GGTGTTGGCC AATACGCCGT ACAGCTCGCA
gTvQR1_R2A  AAAAACATCC TCATCACAGC CGCCTCCGGT GGTGTTGGCC AATACGCCGT TCAGCTCGCA
gTvQR1_R2B  AAAAACATCC TCATCACAGC CGCCTCCGGT GGTGTTGGCC AATACGCCGT TCAGCTCGCA
gTvQR1_n1A  AAAAACATCC TCATCACAGC CGCCTCCGGT GGTGTTGGCC AATACGCCGT TCAGCTCGCA
gTvQR1_n1B  AAAAACATCC TCATCACAGC CGCCTCCGGT GGTGTTGGCC AATACGCCGT TCAGCTCGCA
gTvQR1_N2B  AAAAACATCC TCATCACAGC CGCCTCCGGT GGTGTTGGCC AATACGCCGT TCAGCTCGCA
gTvQR1_N2A  AAAAACATCC TCATCACAGC CGCCTCCGGT GGTGTTGGCC AATACGCCGT TCAGCTCGCA
gTvQR1_n2A  AAAAACATCC TCATCACAGC CGCCTCCGGT GGTGTTGGCC AATACGCCGT TCAGCTCGCA
gTvQR1_n2B  AAAAACATCC TCATCACAGC CGCCTCCGGT GGTGTTGGCC AATACGCCGT TCAGCTCGCA
gTvQR1_N3A  AAAAACATCC TCATCACAGC CGCCTCCGGT GGTGTTGGCC AATACGCCGT TCAGCTCGCA
gTvQR1_N3B  AAAAACATCC TCATCACAGC CGCCTCCGGT GGTGTTGGCC AATACGCCGT TCAGCTCGCA
gTvQR1_n3A  AAAAACATCC TCATCACAGC CGCCTCCGGT GGCGTTGGCC AATACGCCGT TCAGCTCGCA
gTvQR1_n3B  AAAAACATCC TCATCACAGC CGCCTCCGGT GGCGTTGGCC AATACGCCGT TCAGCTCGCA
```

	1501					1560
gTvQR1_r1A	AAGCTAGGAA	ACACACACGT	AACCGCCACA	TGTGGGTCCC	GAAACTTTGA	CTTGGTCAAA
gTvQR1_r1B	AAGCTAGGAA	ACACACACGT	AACCGCCACA	TGTGGGTCCC	GAAACTTTGA	CTTGGTCAAA
gTvQR1_R3A	AAGCTAGGAA	ACACACACGT	AACCGCCACA	TGTGGGTCCC	GAAACTTTGA	CTTGGTCAAA
gTvQR1_R3B	AAGCTAGGAA	ACACACACGT	AACCGCCACA	TGTGGGTCCC	GAAACTTTGA	CTTGGTCAAA
gTvQR1_N1A	AAGCTAGGAA	ACACACACGT	AACCGCCACA	TGTGGGTCCC	GAAACTTTGA	CTTAGTCAAA
gTvQR1_N1B	AAGCTAGGAA	ACACACACGT	AACCGCCACA	TGTGGGTCCC	GAAACTTTGA	CTTAGTCAAA
gTvQR1_r4A	AAGCTAGGAA	ACACACATGT	AACCGCCACA	TGTGGGTCCC	GAAACTTTGA	CTTGGTCAAA
gTvQR1_r4B	AAGCTAGGAA	ACACACATGT	AACCGCCACA	TGTGGGTCCC	GAAACTTTGA	CTTGGTCAAA
gTvQR1_r7A	AAGCTAGGAA	ACACACATGT	AACCGCCACA	TGTGGGTCCC	GAAACTTTGA	CTTAGTCAAA
gTvQR1_r7B	AAGCTAGGAA	ACACACATGT	AACCGCCACA	TGTGGGTCCC	GAAACTTTGA	CTTAGTCAAA
gTvQR1_r3A	AAGCTAGGAA	ACACACACGT	AACCGCCACA	TGCGGGTCCC	GAAACTTTGA	CTTAGTCAAA
gTvQR1_r3B	AAGCTAGGAA	ACACACACGT	AACCGCCACA	TGCGGGTCCC	GAAACTTTGA	CTTAGTCAAA
gTvQR1_r6A	AAGCTAGGAA	ACACACACGT	AACCGCCACA	TGCGGGTCCC	GAAACTTTGA	CTTAGTCAAA
gTvQR1_r6B	AAGCTAGGAA	ACACACACGT	AACCGCCACA	TGCGGGTCCC	GAAACTTTGA	CTTAGTCAAA
gTvQR1_r2A	AAGCTAGGAA	ACACACACGT	AACCGCCACA	TGTGGGTCCC	GAAACTTTGA	CTTGGTCAAA
gTvQR1_r2B	AAGCTAGGAA	ACACACACGT	AACCGCCACA	TGTGGGTCCC	GAAACTTTGA	CTTGGTCAAA
gTvQR1_R1A	AAGCTAGGAA	ACACACACGT	AACCGCCACA	TGTGGGTCCC	GAAACTTTGA	CTTGGTCAAA
gTvQR1_R1B	AAGCTAGGAA	ACACACACGT	AACCGCCACA	TGTGGGTCCC	GAAACTTTGA	CTTGGTCAAA
gTvQR1_R4B	AAGCTAGGAA	ACACACACGT	AACCGCCACA	TGTGGGTCCC	GAAACTTTGA	CTTGGTCAAA
gTvQR1_R4A	AAGCTAGGAA	ACACACACGT	AACCGCCACA	TGTGGGTCCC	GAAACTTTGA	CTTGGTCAAA
gTvQR1_R6B	AAGCTAGGAA	ACACACACGT	AACCGCCACA	TGTGGGTCCC	GAAACTTTGA	CTTGGTCAAA
gTvQR1_R6A	AAGCTAGGAA	ACACACACGT	AACCGCCACA	TGTGGGTCCC	GAAACTTTGA	CTTGGTCAAA
gTvQR1_R5A	AAGCTAGGAA	ACACACACGT	AACCGCCACA	TGTGGGTCCC	GAAACTTTGA	CTTGGTCAAA
gTvQR1_R5B	AAGCTAGGAA	ACACACACGT	AACCGCCACA	TGTGGGTCCC	GAAACTTTGA	CTTGGTCAAA
gTvQR1_r8A	AAGCTAGGAA	ACACACACGT	AACCGCCACA	TGCGGGTCCC	GAAACTTTGA	CTTGGTCAAA
gTvQR1_r8B	AAGCTAGGAA	ACACACACGT	AACCGCCACA	TGCGGGTCCC	GAAACTTTGA	CTTGGTCAAA
gTvQR1_r5A	AAGCTAGGAA	ACACACACGT	AACCGCCACA	TGTGGGTCCC	GAAACTTTGA	CTTGGTCAAA
gTvQR1_r5B	AAGCTAGGAA	ACACACACGT	AACCGCCACA	TGTGGGTCCC	GAAACTTTGA	CTTGGTCAAA
gTvQR1_R2A	AAGCTAGGAA	ACACACACGT	GACCGCCACA	TGTGGGTCCC	GAAACTTTGA	CTTAGTCAAA
gTvQR1_R2B	AAGCTAGGAA	ACACACACGT	GACCGCCACA	TGTGGGTCCC	GAAACTTTGA	CTTAGTCAAA
gTvQR1_n1A	AAGCTAGGAA	ACACACACGT	GACCGCCACA	TGTGGGTCCC	GAAACTTTGA	CTTAGTCAAA
gTvQR1_n1B	AAGCTAGGAA	ACACACACGT	GACCGCCACA	TGTGGGTCCC	GAAACTTTGA	CTTAGTCAAA
gTvQR1_N2B	AAGCTAGGAA	ACACACACGT	GACCGCCACA	TGTGGGTCCC	GAAACTTTGA	CTTAGTCAAA
gTvQR1_N2A	AAGCTAGGAA	ACACACACGT	GACCGCCACA	TGTGGGTCCC	GAAACTTTGA	CTTAGTCAAA
gTvQR1_n2A	AAGCTAGGAA	ACACACACGT	GACCGCCACA	TGTGGGTCCC	GAAACTTTGA	CTTAGTCAAA
gTvQR1_n2B	AAGCTAGGAA	ACACACACGT	GACCGCCACA	TGTGGGTCCC	GAAACTTTGA	CTTAGTCAAA
gTvQR1_N3A	AAGCTAGGAA	ACACACACGT	GACCGCCACA	TGTGGGTCCC	GAAACTTTGA	CTTAGTCAAA
gTvQR1_N3B	AAGCTAGGAA	ACACACACGT	GACCGCCACA	TGTGGGTCCC	GAAACTTTGA	CTTAGTCAAA
gTvQR1_n3A	AAGCTAGGAA	ACACACACGT	AACCGCCACA	TGCGGGTCCC	GAAACTTTGA	CTTGGTCAAA
gTvQR1_n3B	AAGCTAGGAA	ACACACACGT	AACCGCCACA	TGCGGGTCCC	GAAACTTTGA	CTTGGTCAAA

```
              1561                                                            1620
gTvQR1_r1A    AGCCTCGGAG CCGACGAGGT TATTGACTAT AAAACCCCCG AAGGGGCAGC CCTTAAGAGC
gTvQR1_r1B    AGCCTCGGAG CCGACGAGGT TATTGACTAT AAAACCCCCG AAGGGGCAGC CCTTAAGAGC
gTvQR1_R3A    AGCCTCGGAG CCGACGAGGT TATTGACTAT AAAACCCCCG AAGGGGCAGC CCTTAAGAGC
gTvQR1_R3B    AGCCTCGGAG CCGACGAGGT TATTGACTAT AAAACCCCCG AAGGGGCAGC CCTTAAGAGC
gTvQR1_N1A    AGCCTCGGAG CCGACGAGGT TATTGACTAT AAAACCCCCG AAGGGGCAGC CCTTAAGAGC
gTvQR1_N1B    AGCCTCGGAG CCGACGAGGT TATTGACTAT AAAACCCCCG AAGGGGCAGC CCTTAAGAGC
gTvQR1_r4A    AGCCTCGGAG CCGACGAGGT TATTGACTAT AAAACCCCCG AAGGGGCAGC CCTTAAGAGC
gTvQR1_r4B    AGCCTCGGAG CCGACGAGGT TATTGACTAT AAAACCCCCG AAGGGGCAGC CCTTAAGAGC
gTvQR1_r7A    AGCCTCGGAG CCGACGAGGT TATTGACTAT AAAACCCCTG AAGGGGCAGC ACTTAAGAGC
gTvQR1_r7B    AGCCTCGGAG CCGACGAGGT TATTGACTAT AAAACCCCTG AAGGGGCAGC ACTTAAGAGC
gTvQR1_r3A    AGCCTCGGAG CCGATGAGGT TATTGACTAT AAAACCCCCG AAGGGGCAGC CCTTAAGAGC
gTvQR1_r3B    AGCCTCGGAG CCGATGAGGT TATTGACTAT AAAACCCCCG AAGGGGCAGC CCTTAAGAGC
gTvQR1_r6A    AGCCTCGGAG CCGATGAGGT TATTGACTAT AAAACCCCCG AAGGGGCAGC CCTTAAGAGC
gTvQR1_r6B    AGCCTCGGAG CCGATGAGGT TATTGACTAT AAAACCCCCG AAGGGGCAGC CCTTAAGAGC
gTvQR1_r2A    AGCCTTGGGG CCGACGAGGT TATTGACTAT AAAACCCCCG AAGGGGCAGC CCTTAAGAGC
gTvQR1_r2B    AGCCTTGGGG CCGACGAGGT TATTGACTAT AAAACCCCCG AAGGGGCAGC CCTTAAGAGC
gTvQR1_R1A    AGCCTTGGGG CCGACGAGGT TATTGACTAT AAAACCCCCG AAGGGGCAGC CCTTAAGAGC
gTvQR1_R1B    AGCCTTGGGG CCGACGAGGT TATTGACTAT AAAACCCCCG AAGGGGCAGC CCTTAAGAGC
gTvQR1_R4B    AGCCTTGGGG CCGACGAGGT TATTGACTAT AAAACCCCCG AAGGGGCAGC CCTTAAGAGC
gTvQR1_R4A    AGCCTTGGGG CCGACGAGGT TATTGACTAT AAAACCCCCG AAGGGGCAGC CCTTAAGAGC
gTvQR1_R6B    AGCCTTGGGG CCGACGAGGT TATTGACTAT AAAACCCCCG AAGGGGCAGC CCTTAAGAGC
gTvQR1_R6A    AGCCTTGGGG CCGACGAGGT TATTGACTAT AAAACCCCCG AAGGGGCAGC CCTTAAGAGC
gTvQR1_R5A    AGCCTTGGGG CCGACGAGGT TATTGACTAT AAAACCCCCG AAGGGGCAGC CCTTAAGAGC
gTvQR1_R5B    AGCCTTGGGG CCGACGAGGT TATTGACTAT AAAACCCCCG AAGGGGCAGC CCTTAAGAGC
gTvQR1_r8A    AGCCTCGGAG CCGATGAGGT TATTGACTAT AAAACCCCCG AAGGGGCAGC CCTTACGAGC
gTvQR1_r8B    AGCCTCGGAG CCGATGAGGT TATTGACTAT AAAACCCCCG AAGGGGCAGC CCTTACGAGC
gTvQR1_r5A    AGCCTTGGGG CCGACGAGGT TATTGACTAT AAAACCCCCG AAGGGGCAGC CCTTAAGAGC
gTvQR1_r5B    AGCCTTGGGG CCGACGAGGT TATTGACTAT AAAACCCCCG AAGGGGCAGC CCTTAAGAGC
gTvQR1_R2A    AGCCTCGGAG CCGACGAGGT TATTGACTAT AAAACCCCCG AAGGGGCAGC CCTTAAGAGC
gTvQR1_R2B    AGCCTCGGAG CCGACGAGGT TATTGACTAT AAAACCCCCG AAGGGGCAGC CCTTAAGAGC
gTvQR1_n1A    AGCCTCGGAG CCGACGAGGT TATTGACTAT AAAACCCCCG AAGGGGCAGC CCTTAAGAGC
gTvQR1_n1B    AGCCTCGGAG CCGACGAGGT TATTGACTAT AAAACCCCCG AAGGGGCAGC CCTTAAGAGC
gTvQR1_N2B    AGCCTCGGAG CCGACGAGGT TATTGACTAT AAAACCCCCG AAGGGGCAGC CCTTAAGAGC
gTvQR1_N2A    AGCCTCGGAG CCGACGAGGT TATTGACTAT AAAACCCCCG AAGGGGCAGC CCTTAAGAGC
gTvQR1_n2A    AGCCTCGGAG CCGACGAGGT TATTGACTAT AAAACCCCCG AAGGGGCAGC CCTTAAGAGC
gTvQR1_n2B    AGCCTCGGAG CCGACGAGGT TATTGACTAT AAAACCCCCG AAGGGGCAGC CCTTAAGAGC
gTvQR1_N3A    AGCCTCGGAG CCGACGAGGT TATTGACTAT AAAACCCCCG AAGGGGCAGC CCTTAAGAGC
gTvQR1_N3B    AGCCTCGGAG CCGACGAGGT TATTGACTAT AAAACCCCCG AAGGGGCAGC CCTTAAGAGC
gTvQR1_n3A    AGCCTCGGAG CCGACGAGGT TATTGACTAT AAAACCCCCG AAGGGGCAGC ACTTAAGAGC
gTvQR1_n3B    AGCCTCGGAG CCGACGAGGT TATTGACTAT AAAACCCCCG AAGGGGCAGC ACTTAAGAGC
```

```
             1621                                                        1680
gTvQR1_r1A   CCTTCGGGCA AAAAGTATGA TGCGGTTATT CATTGTGCGT CGCCTTTACC ATGGTCCGTT
gTvQR1_r1B   CCTTCGGGCA AAAAGTATGA TGCGGTTATT CATTGTGCGT CGCCTTTACC ATGGTCCGTT
gTvQR1_R3A   CCTTCGGGCA AAAAGTATGA TGCGGTTATT CATTGTGCGT CGCCTTTACC ATGGTCCGTT
gTvQR1_R3B   CCTTCGGGCA AAAAGTATGA TGCGGTTATT CATTGTGCGT CGCCTTTACC ATGGTCCGTT
gTvQR1_N1A   CCGTCGGGCA AAAAGTACGA TGCGGTTATT CATTGTGCAT CACCTTTGCC ATGGTCCGTT
gTvQR1_N1B   CCGTCGGGCA AAAAGTACGA TGCGGTTATT CATTGTGCAT CACCTTTGCC ATGGTCCGTT
gTvQR1_r4A   CCTTCGGGCA AAAAGTATGA TGCGGTTATT CATTGTGCAT CGCCTTTGCC ATGGTCCGTT
gTvQR1_r4B   CCTTCGGGCA AAAAGTATGA TGCGGTTATT CATTGTGCAT CGCCTTTGCC ATGGTCCGTT
gTvQR1_r7A   CCGTCGGGCA AAAAGTATGA TGCGGTTATT CATTGTGCAT CGCCTTTGCC ATGGTCCGTT
gTvQR1_r7B   CCGTCGGGCA AAAAGTATGA TGCGGTTATT CATTGTGCAT CGCCTTTGCC ATGGTCCGTT
gTvQR1_r3A   CCGTCGGGCA AAAAGTACGA TGCGGTTATT CATTGTGCAT CACCTTTGCC ATGGTCCGTT
gTvQR1_r3B   CCGTCGGGCA AAAAGTACGA TGCGGTTATT CATTGTGCAT CACCTTTGCC ATGGTCCGTT
gTvQR1_r6A   CCGTCGGGCA AAAAGTACGA TGCGGTTATT CATTGTGCAT CACCTTTGCC ATGGTCCGTT
gTvQR1_r6B   CCGTCGGGCA AAAAGTACGA TGCGGTTATT CATTGTGCAT CACCTTTGCC ATGGTCCGTT
gTvQR1_r2A   CCGTCGGGCA AAAAGTACGA TGCGGTTATT CATTGTGCAT CGCCTTTGCC ATGGTCCGTT
gTvQR1_r2B   CCGTCGGGCA AAAAGTACGA TGCGGTTATT CATTGTGCAT CGCCTTTGCC ATGGTCCGTT
gTvQR1_R1A   CCGTCGGGCA AAAAGTACGA TGCGGTTATT CATTGTGCAT CGCCTTTGCC ATGGTCCGTT
gTvQR1_R1B   CCGTCGGGCA AAAAGTACGA TGCGGTTATT CATTGTGCAT CGCCTTTGCC ATGGTCCGTT
gTvQR1_R4B   CCGTCGGGCA AAAAGTACGA TGCGGTTATT CATTGTGCAT CGCCTTTGCC ATGGTCCGTT
gTvQR1_R4A   CCGTCGGGCA AAAAGTACGA TGCGGTTATT CATTGTGCAT CGCCTTTGCC ATGGTCCGTT
gTvQR1_R6B   CCGTCGGGCA AAAAGTACGA TGCGGTTATT CATTGTGCAT CGCCTTTGCC ATGGTCCGTT
gTvQR1_R6A   CCGTCGGGCA AAAAGTACGA TGCGGTTATT CATTGTGCAT CGCCTTTGCC ATGGTCCGTT
gTvQR1_R5A   CCGTCGGGCA AAAAGTACGA TGCGGTTATT CATTGTGCAT CGCCTTTGCC ATGGTCCGTT
gTvQR1_R5B   CCGTCGGGCA AAAAGTACGA TGCGGTTATT CATTGTGCAT CGCCTTTGCC ATGGTCCGTT
gTvQR1_r8A   CCGTCGGGCA AGAAGTACGA TGCGGTTATT CATTGTGCAT CGCCTTTGCC ATGGTCCGTT
gTvQR1_r8B   CCGTCGGGCA AGAAGTACGA TGCGGTTATT CATTGTGCAT CGCCTTTGCC ATGGTCCGTT
gTvQR1_r5A   CCGTCGGGCA AAAAGTACGA TGCGGTTATT CATTGTGCAT CGCCTTTGCC ATGGTCCGTT
gTvQR1_r5B   CCGTCGGGCA AAAAGTACGA TGCGGTTATT CATTGTGCAT CGCCTTTGCC ATGGTCCGTT
gTvQR1_R2A   CCTTCGGGCA AAAAGTATGA TGCGGTTATT CATTGTGCGT CGCCTTTACC ATGGTCCGTT
gTvQR1_R2B   CCTTCGGGCA AAAAGTATGA TGCGGTTATT CATTGTGCGT CGCCTTTACC ATGGTCCGTT
gTvQR1_n1A   CCTTCGGGCA AAAAGTATGA TGCGGTTATT CATTGTGCGT CGCCTTTACC ATGGTCCGTT
gTvQR1_n1B   CCTTCGGGCA AAAAGTATGA TGCGGTTATT CATTGTGCGT CGCCTTTACC ATGGTCCGTT
gTvQR1_N2B   CCTTCGGGCA AAAAGTATGA TGCGGTTATT CATTGTGCGT CGCCTTTACC ATGGTCCGTT
gTvQR1_N2A   CCTTCGGGCA AAAAGTATGA TGCGGTTATT CATTGTGCGT CGCCTTTACC ATGGTCCGTT
gTvQR1_n2A   CCTTCGGGCA AAAAGTATGA TGCGGTTATT CATTGTGCGT CGCCTTTACC ATGGTCCGTT
gTvQR1_n2B   CCTTCGGGCA AAAAGTATGA TGCGGTTATT CATTGTGCGT CGCCTTTACC ATGGTCCGTT
gTvQR1_N3A   CCTTCGGGCA AAAAGTATGA TGCGGTTATT CATTGTGCGT CGCCTTTACC ATGGTCCGTT
gTvQR1_N3B   CCTTCGGGCA AAAAGTATGA TGCGGTTATT CATTGTGCGT CGCCTTTACC ATGGTCCGTT
gTvQR1_n3A   CCGTCGGGCA AAAAGTACGA TGCGGTTATT CATTGTGCGT CGCCTTTGCC ATGGTCCGTT
gTvQR1_n3B   CCGTCGGGCA AAAAGTACGA TGCGGTTATT CATTGTGCGT CGCCTTTGCC ATGGTCCGTT
```

```
              1681                                                    1740
gTvQR1_r1A   TTTAAACCGA ACTTGAGCAA ACATGGGAAA GTGATCGATA TAACTCCCGG ACCGAGGGTT
gTvQR1_r1B   TTTAAACCGA ACTTGAGCAA ACATGGGAAA GTGATCGATA TAACTCCCGG ACCGAGGGTT
gTvQR1_R3A   TTTAAACCGA ACTTGAGCAA ACATGGGAAA GTGATCGATA TAACTCCCGG ACCGAGGGTT
gTvQR1_R3B   TTTAAACCGA ACTTGAGCAA ACATGGGAAA GTGATCGATA TAACTCCCGG ACCGAGGGTT
gTvQR1_N1A   TTTAAACCGA ACTTGAGCAA ACATGGGAAA GTGATCGATA TAACTCCTGG TCCGAGGGTT
gTvQR1_N1B   TTTAAACCGA ACTTGAGCAA ACATGGGAAA GTGATCGATA TAACTCCTGG TCCGAGGGTT
gTvQR1_r4A   TTTAAACCGA ACTTGAGCAA ACATGGGAAA GTGATCGATA TAACTCCCGG TCCGAGGGTT
gTvQR1_r4B   TTTAAACCGA ACTTGAGCAA ACATGGGAAA GTGATCGATA TAACTCCCGG TCCGAGGGTT
gTvQR1_r7A   TTTAAACCGA ACTTGAGCAA ACATGGGAAA GTGATTGATA TAACTCCCGG TCCGAGGGTT
gTvQR1_r7B   TTTAAACCGA ACTTGAGCAA ACATGGGAAA GTGATTGATA TAACTCCCGG TCCGAGGGTT
gTvQR1_r3A   TTTAAACCGA ACTTGAGCAA ACATGGGAAA GTGATCGATA TAACTCCCGG ACCGAGGGTT
gTvQR1_r3B   TTTAAACCGA ACTTGAGCAA ACATGGGAAA GTGATCGATA TAACTCCCGG ACCGAGGGTT
gTvQR1_r6A   TTTAAACCGA ACTTGAGCAA ACATGGGAAA GTGATCGATA TAACTCCCGG GCCGAGGGTT
gTvQR1_r6B   TTTAAACCGA ACTTGAGCAA ACATGGGAAA GTGATCGATA TAACTCCCGG GCCGAGGGTT
gTvQR1_r2A   TTTAAACCGA ACTTGAGCAA ACGTGGGAAA GTGATTGATA TAACTCCCGG ACCGAGGGTT
gTvQR1_r2B   TTTAAACCGA ACTTGAGCAA ACGTGGGAAA GTGATTGATA TAACTCCCGG ACCGAGGGTT
gTvQR1_R1A   TTTAAACCGA ACTTGAGCAA ACGTGGGAAA GTGATTGATA TAACTCCCGG ACCGAGGGTT
gTvQR1_R1B   TTTAAACCGA ACTTGAGCAA ACGTGGGAAA GTGATTGATA TAACTCCCGG ACCGAGGGTT
gTvQR1_R4B   TTTAAACCGA ACTTGAGCAA ACGTGGGAAA GTGATTGATA TAACTCCCGG ACCGAGGGTT
gTvQR1_R4A   TTTAAACCGA ACTTGAGCAA ACGTGGGAAA GTGATTGATA TAACTCCCGG ACCGAGGGTT
gTvQR1_R6B   TTTAAACCGA ACTTGAGCAA ACGTGGGAAA GTGATTGATA TAACTCCCGG ACCGAGGGTT
gTvQR1_R6A   TTTAAACCGA ACTTGAGCAA ACGTGGGAAA GTGATTGATA TAACTCCCGG ACCGAGGGTT
gTvQR1_R5A   TTTAAACCGA ACTTGAGCAA ACGTGGGAAA GTGATTGATA TAACTCCCGG ACCGAGGGTT
gTvQR1_R5B   TTTAAACCGA ACTTGAGCAA ACGTGGGAAA GTGATTGATA TAACTCCCGG ACCGAGGGTT
gTvQR1_r8A   TTTAAACCGA ACTTGAGCAA ACATGGGAAA GTGATCGATA TAACTCCCGG ACCGAGGGTT
gTvQR1_r8B   TTTAAACCGA ACTTGAGCAA ACATGGGAAA GTGATCGATA TAACTCCCGG ACCGAGGGTT
gTvQR1_r5A   TTTAAACCGA ACTTGAGCAA ACGTGGGAAA GTGATTGATA TAACTCCCGG ACCGAGGGTT
gTvQR1_r5B   TTTAAACCGA ACTTGAGCAA ACGTGGGAAA GTGATTGATA TAACTCCCGG ACCGAGGGTT
gTvQR1_R2A   TTTAAACCGA ACTTGAGCAA ACATGGGAAA GTGATCGATA TAACTCCCGG ACCGAGGGTT
gTvQR1_R2B   TTTAAACCGA ACTTGAGCAA ACATGGGAAA GTGATCGATA TAACTCCCGG ACCGAGGGTT
gTvQR1_n1A   TTTAAACCGA ACTTGAGCAA ACATGGGAAA GTGATCGATA TAACTCCCGG ACCGAGGGTT
gTvQR1_n1B   TTTAAACCGA ACTTGAGCAA ACATGGGAAA GTGATCGATA TAACTCCCGG ACCGAGGGTT
gTvQR1_N2B   TTTAAACCGA ACTTGAGCAA ACATGGGAAA GTGATCGATA TAACTCCCGG ACCGAGGGTT
gTvQR1_N2A   TTTAAACCGA ACTTGAGCAA ACATGGGAAA GTGATCGATA TAACTCCCGG ACCGAGGGTT
gTvQR1_n2A   TTTAAACCGA ACTTGAGCAA ACATGGGAAA GTGATCGATA TAACTCCCGG ACCGAGGGTT
gTvQR1_n2B   TTTAAACCGA ACTTGAGCAA ACATGGGAAA GTGATCGATA TAACTCCCGG ACCGAGGGTT
gTvQR1_N3A   TTTAAACCGA ACTTGAGCAA ACATGGGAAA GTGATCGATA TAACTCCCGG ACCGAGGGTT
gTvQR1_N3B   TTTAAACCGA ACTTGAGCAA ACATGGGAAA GTGATCGATA TAACTCCCGG ACCGAGGGTT
gTvQR1_n3A   TTTAAACCGA ACTTGAGCAA ACATGGGAAA GTGATTGATA TAACTCCCGG TCCGAGGGTT
gTvQR1_n3B   TTTAAACCGA ACTTGAGCAA ACATGGGAAA GTGATTGATA TAACTCCCGG TCCGAGGGTT
```

	1741					1800
gTvQR1_r1A	ATGTTGACTT	CCGCTATGAC	AAAACTTACG	TGCTCGAAGA	AACGATTGGT	GACGTTACTT
gTvQR1_r1B	ATGTTGACTT	CCGCTATGAC	AAAACTTACG	TGCTCGAAGA	AACGATTGGT	GACGTTACTT
gTvQR1_R3A	ATGTTGACTT	CCGCTATGAC	AAAACTTACG	TGCTCGAAGA	AACGATTGGT	GACGTTACTT
gTvQR1_R3B	ATGTTGACTT	CCGCTATGAC	AAAACTTACG	TGCTCGAAGA	AACGATTGGT	GACGTTACTT
gTvQR1_N1A	ATGTTGACTT	CTGCTATGAC	AAAACTCACG	TGTTCTAAGA	AACGATTGGT	GACGTTACTT
gTvQR1_N1B	ATGTTGACTT	CTGCTATGAC	AAAACTCACG	TGTTCTAAGA	AACGATTGGT	GACGTTACTT
gTvQR1_r4A	ATGTTGACTT	CGGCTATGAC	AAAACTCACG	TGCTCGAAGA	AACGATTGGT	GACGTTACTT
gTvQR1_r4B	ATGTTGACTT	CGGCTATGAC	AAAACTCACG	TGCTCGAAGA	AACGATTGGT	GACGTTACTT
gTvQR1_r7A	ATGTTGACTT	CGGCTATGAC	AAAACTCACG	TGCTCTAAGA	AACGATTGGT	GACGTTACTT
gTvQR1_r7B	ATGTTGACTT	CGGCTATGAC	AAAACTCACG	TGCTCTAAGA	AACGATTGGT	GACGTTACTT
gTvQR1_r3A	ATGTTGACTT	CCGCTATGAC	AAAACTTACG	TGCTCGAAGA	AAAGATTGGT	GACGTTACTT
gTvQR1_r3B	ATGTTGACTT	CTGCTATGAC	AAAACTCACG	TGCTCGAAGA	AAAGATTGGT	GACGTTACTT
gTvQR1_r6A	ATGTTGACTT	CTGCTATGAC	AAAACTCACG	TGCTCGAAGA	AACGATTGGT	GACGTTACTT
gTvQR1_r6B	ATGTTGACTT	CTGCTATGAC	AAAACTCACG	TGCTCGAAGA	AACGATTGGT	GACGTTACTT
gTvQR1_r2A	ATGTTGACTT	CTGCTATGAC	AAAACTTACG	TGCTCGAAGA	AACGATTGGT	GACGTTACTT
gTvQR1_r2B	ATGTTGACTT	CTGCTATGAC	AAAACTTACG	TGCTCGAAGA	AACGATTGGT	GACGTTACTT
gTvQR1_R1A	ATGTTGACTT	CTGCTATGAC	AAAACTTACG	TGCTCGAAGA	AACGATTGGT	GACGTTACTT
gTvQR1_R1B	ATGTTGACTT	CTGCTATGAC	AAAACTTACG	TGCTCGAAGA	AACGATTGGT	GACGTTACTT
gTvQR1_R4B	ATGTTGACTT	CTGCTATGAC	AAAACTTACG	TGCTCGAAGA	AACGATTGGT	GACGTTACTT
gTvQR1_R4A	ATGTTGACTT	CTGCTATGAC	AAAACTTACG	TGCTCGAAGA	AACGATTGGT	GACGTTACTT
gTvQR1_R6B	ATGTTGACTT	CTGCTATGAC	AAAACTTACG	TGCTCGAAGA	AACGATTGGT	GACGTTACTT
gTvQR1_R6A	ATGTTGACTT	CTGCTATGAC	AAAACTTACG	TGCTCGAAGA	AACGATTGGT	GACGTTACTT
gTvQR1_R5A	ATGTTGACTT	CTGCTATGAC	AAAACTTACG	TGCTCGAAGA	AACGATTGGT	GACGTTACTT
gTvQR1_R5B	ATGTTGACTT	CTGCTATGAC	AAAACTTACG	TGCTCGAAGA	AACGATTGGT	GACGTTACTT
gTvQR1_r8A	ATGTTGACTT	CTGCTATGAC	AAAACTCACG	TGCTCTAAGA	AACGATTGGT	GACGTTACTT
gTvQR1_r8B	ATGTTGACTT	CTGCTATGAC	AAAACTCACG	TGCTCTAAGA	AACGATTGGT	GACGTTACTT
gTvQR1_r5A	ATGTTGACTT	CTGCTATGAC	AAAACTTACG	TGCTCGAAGA	AACGATTGGT	GACGTTACTT
gTvQR1_r5B	ATGTTGACTT	CTGCTATGAC	AAAACTTACG	TGCTCGAAGA	AACGATTGGT	GACGTTACTT
gTvQR1_R2A	ATGTTGACTT	CCGCTATGAC	AAAACTTACG	TGCTCGAAGA	AACGATTGGT	GACGTTACTT
gTvQR1_R2B	ATGTTGACTT	CCGCTATGAC	AAAACTTACG	TGCTCGAAGA	AACGATTGGT	GACGTTACTT
gTvQR1_n1A	ATGTTGACTT	CCGCTATGAC	AAAACTTACG	TGCTCGAAGA	AACGATTGGT	GACGTTACTT
gTvQR1_n1B	ATGTTGACTT	CCGCTATGAC	AAAACTTACG	TGCTCGAAGA	AACGATTGGT	GACGTTACTT
gTvQR1_N2B	ATGTTGACTT	CCGCTATGAC	AAAACTTACG	TGCTCGAAGA	AACGATTGGT	GACGTTACTT
gTvQR1_N2A	ATGTTGACTT	CCGCTATGAC	AAAACTTACG	TGCTCGAAGA	AACGATTGGT	GACGTTACTT
gTvQR1_n2A	ATGTTGACTT	CCGCTATGAC	AAAACTTACG	TGCTCGAAGA	AACGATTGGT	GACGTTACTT
gTvQR1_n2B	ATGTTGACTT	CCGCTATGAC	AAAACTTACG	TGCTCGAAGA	AACGATTGGT	GACGTTACTT
gTvQR1_N3A	ATGTTGACTT	CCGCTATGAC	AAAACTTACG	TGCTCGAAGA	AACGATTGGT	GACGTTACTT
gTvQR1_N3B	ATGTTGACTT	CCGCTATGAC	AAAACTTACG	TGCTCGAAGA	AACGATTGGT	GACGTTACTT
gTvQR1_n3A	ATGTTGACTT	CTGCTATGAC	AAAACTTACG	TGTTCGAAGA	AACGATTGGA	GACGTTACTT
gTvQR1_n3B	ATGTTGACTT	CTGCTATGAC	AAAACTTACG	TGTTCGAAGA	AACGATTGGA	GACGTTACTT

	1801					1860
gTvQR1_r1A	GTTGTGATCA	AGGGCGAGCA	TTTGAGTTAT	CTTGTCGAGT	TAATGAGAGA	GGGGAAACTT
gTvQR1_r1B	GTTGTGATCA	AGGGCGAGCA	TTTGAGTTAT	CTTGTCGAGT	TAATGAGAGA	GGGGAAACTT
gTvQR1_R3A	GTTGTGATCA	AGGGCGAGCA	TTTGAGTTAT	CTTGTCGAGT	TAATGAGAGA	GGGGAAACTT
gTvQR1_R3B	GTTGTGATCA	AGGGCGAGCA	TTTGAGTTAT	CTTGTCGAGT	TAATGAGAGA	GGGGAAACTT
gTvQR1_N1A	GTTGTGATCA	AGGGCGAGCA	TTTGAGTTAT	CTTGTCGGGT	TAATGAGAGA	AGGGAAACTT
gTvQR1_N1B	GTTGTGATCA	AGGGCGAGCA	TTTGAGTTAT	CTTGTCGGGT	TAATGAGAGA	AGGGAAACTT
gTvQR1_r4A	GTTGTGATCA	AGGATGAGCA	TTTGAGTTAT	CTGGTCGGGT	TAATGAGAGA	AGGGAAACTT
gTvQR1_r4B	GTTGTGATCA	AGGATGAGCA	TTTGAGTTAT	CTGGTCGGGT	TAATGAGAGA	AGGGAAACTT
gTvQR1_r7A	GTTGTGATCA	AGGGCGAGCA	TTTGAGTTAT	CTTGTCGGGT	TAATGAGAGA	AGGGAAACTT
gTvQR1_r7B	GTTGTGATCA	AGGGCGAGCA	TTTGAGTTAT	CTTGTCGGGT	TAATGAGAGA	AGGGAAACTT
gTvQR1_r3A	GTTGTGATCA	AGGGCGAGCA	TTTGAGTTAT	CTTGTCAAGT	TAATGAGAGA	AGGGAAACTT
gTvQR1_r3B	GTTGTGATCA	AGGGCGAGCA	TTTGAGTTAT	CTTGTCAAGT	TAATGAGAGA	AGGGAAACTT
gTvQR1_r6A	GTTGTGATCA	AGGGCGAGCA	TTTGAGTTAT	CTTGTCGAGT	TAATGAGAGA	AGGGAAACTT
gTvQR1_r6B	GTTGTGATCA	AGGGCGAGCA	TTTGAGTTAT	CTTGTCGAGT	TAATGAGAGA	AGGGAAACTT
gTvQR1_r2A	GTTGTGATCA	AGGGCGAGCA	TTTGAGTTAT	CTTGTCGAGT	TAATGAGAGA	AGGGAAACTT
gTvQR1_r2B	GTTGTGATCA	AGGGCGAGCA	TTTGAGTTAT	CTTGTCGAGT	TAATGAGAGA	AGGGAAACTT
gTvQR1_R1A	GTTGTGATCA	AGGGCGAGCA	TTTGAGTTAT	CTTGTCGAGT	TAATGAGAGA	AGGGAAACTT
gTvQR1_R1B	GTTGTGATCA	AGGGCGAGCA	TTTGAGTTAT	CTTGTCGAGT	TAATGAGAGA	AGGGAAACTT
gTvQR1_R4B	GTTGTGATCA	AGGGCGAGCA	TTTGAGTTAT	CTTGTCGAGT	TAATGAGAGA	AGGGAAACTT
gTvQR1_R4A	GTTGTGATCA	AGGGCGAGCA	TTTGAGTTAT	CTTGTCGAGT	TAATGAGAGA	AGGGAAACTT
gTvQR1_R6B	GTTGTGATCA	AGGGCGAGCA	TTTGAGTTAT	CTTGTCGAGT	TAATGAGAGA	AGGGAAACTT
gTvQR1_R6A	GTTGTGATCA	AGGGCGAGCA	TTTGAGTTAT	CTTGTCGAGT	TAATGAGAGA	AGGGAAACTT
gTvQR1_R5A	GTTGTGATCA	AGGGCGAGCA	TTTGAGTTAT	CTTGTCGAGT	TAATGAGAGA	AGGGAAACTT
gTvQR1_R5B	GTTGTGATCA	AGGGCGAGCA	TTTGAGTTAT	CTTGTCGAGT	TAATGAGAGA	AGGGAAACTT
gTvQR1_r8A	GTTGTGATCA	AGGGCGAGCA	TTTGAGTTAT	CTTGTCGGGT	TAATGAGAGA	AGGGAAACTT
gTvQR1_r8B	GTTGTGATCA	AGGGCGAGCA	TTTGAGTTAT	CTTGTCGGGT	TAATGAGAGA	AGGGAAACTT
gTvQR1_r5A	GTTGTGATCA	AGGGCGAGCA	TTTGAGTTAT	CTTGTCGAGT	TAATGAGAGA	AGGGAAACTT
gTvQR1_r5B	GTTGTGATCA	AGGGCGAGCA	TTTGAGTTAT	CTTGTCGAGT	TAATGAGAGA	AGGGAAACTT
gTvQR1_R2A	GTTGTGATCA	AGGGCGAGCA	TTTGAGTTAT	CTTGTCGAGT	TAATGAGAGA	GGGGAAACTT
gTvQR1_R2B	GTTGTGATCA	AGGGCGAGCA	TTTGAGTTAT	CTTGTCGAGT	TAATGAGAGA	GGGGAAACTT
gTvQR1_n1A	GTTGTGATCA	AGGGCGAGCA	TTTGAGTTAT	CTTGTCGAGT	TAATGAGAGA	GGGGAAACTT
gTvQR1_n1B	GTTGTGATCA	AGGGCGAGCA	TTTGAGTTAT	CTTGTCGAGT	TAATGAGAGA	GGGGAAACTT
gTvQR1_N2B	GTTGTGATCA	AGGGCGAGCA	TTTGAGTTAT	CTTGTCGAGT	TAATGAGAGA	GGGGAAACTT
gTvQR1_N2A	GTTGTGATCA	AGGGCGAGCA	TTTGAGTTAT	CTTGTCGAGT	TAATGAGAGA	GGGGAAACTT
gTvQR1_n2A	GTTGTGATCA	AGGGCGAGCA	TTTGAGTTAT	CTTGTCGAGT	TAATGAGAGA	GGGGAAACTT
gTvQR1_n2B	GTTGTGATCA	AGGGCGAGCA	TTTGAGTTAT	CTTGTCGAGT	TAATGAGAGA	GGGGAAACTT
gTvQR1_N3A	GTTGTGATCA	AGGGCGAGCA	TTTGAGTTAT	CTTGTCGAGT	TAATGAGAGA	GGGGAAACTT
gTvQR1_N3B	GTTGTGATCA	AGGGCGAGCA	TTTGAGTTAT	CTTGTCGAGT	TAATGAGAGA	GGGGAAACTT
gTvQR1_n3A	GTTGTGATCA	AGGGCGAGCA	TTTGAGTTAT	CTTGTCCAGT	TAATGAGAGA	AGGGAAACTT
gTvQR1_n3B	GTTGTGATCA	AGGGCGAGCA	TTTGAGTTAT	CTTGTCCAGT	TAATGAGAGA	AGGGAAACTT

```
            1861                                                        1920
gTvQR1_r1A  AAGACGGTCA TCGACTCTAA GTTTCCGTTG AGTAAGGCTG AGGAGGCTTG GGCTAAGAGC
gTvQR1_r1B  AAGACGGTCA TCGACTCTAA GTTTCCGTTG AGTAAGGCTG AGGAGGCTTG GGCTAAGAGC
gTvQR1_R3A  AAGACGGTCA TCGACTCTAA GTTTCCGTTG AGTAAGGCTG AGGAGGCTTG GGCTAAGAGC
gTvQR1_R3B  AAGACGGTCA TCGACTCTAA GTTTCCGTTG AGTAAGGCTG AGGAGGCTTG GGCTAAGAGC
gTvQR1_N1A  AAGACGGTTA TCGACTCTAA GTTTCTGTTG AGTAAAGCTG AGGAGGCTTG GGCTAAGAGC
gTvQR1_N1B  AAGACGGTTA TCGACTCTAA GTTTCTGTTG AGTAAAGCTG AGGAGGCTTG GGCTAAGAGC
gTvQR1_r4A  AGGACCGTTA TCGACTCTAA GTTTCCGTTA AGTAAGGCTG AGGAGGCTTG GGCAAAGAGC
gTvQR1_r4B  AGGACCGTTA TCGACTCTAA GTTTCCGTTA AGTAAGGCTG AGGAGGCTTG GGCAAAGAGC
gTvQR1_r7A  AAGACAGTTA TCGACTCTAA GTTTTCTTTG AGCAAGGCTG AGGAGGCTTG GGCAAAGAGC
gTvQR1_r7B  AAGACAGTTA TCGACTCTAA GTTTTCTTTG AGCAAGGCTG AGGAGGCTTG GGCAAAGAGC
gTvQR1_r3A  AAGACCGTTA TCGACTCTAA GTTTCCGTTG AGTAAGGCTG AGGAGGCTTG GGCTAAGAGC
gTvQR1_r3B  AAGACCGTTA TCGACTCTAA GTTTCCGTTG AGTAAGGCTG AGGAGGCTTG GGCTAAGAGC
gTvQR1_r6A  AAGACCGTTA TCGACTCTAA GTTTCCGTTA AGTAAGGCTG AGGAGGCTTG GGCAAAGAGC
gTvQR1_r6B  AAGACCGTTA TCGACTCTAA GTTTCCGTTA AGTAAGGCTG AGGAGGCTTG GGCAAAGAGC
gTvQR1_r2A  AAGACCGTTA TCGACTCTAA GTTTCCGTTG AGTAAGGCTG AGGAGGCTTG GGCAAAGAGC
gTvQR1_r2B  AAGACCGTTA TCGACTCTAA GTTTCCGTTG AGTAAGGCTG AGGAGGCTTG GGCAAAGAGC
gTvQR1_R1A  AAGACCGTTA TCGACTCTAA GTTTCCGTTG AGTAAGGCTG AGGAGGCTTG GGCAAAGAGC
gTvQR1_R1B  AAGACCGTTA TCGACTCTAA GTTTCCGTTG AGTAAGGCTG AGGAGGCTTG GGCAAAGAGC
gTvQR1_R4B  AAGACCGTTA TCGACTCTAA GTTTCCGTTG AGTAAGGCTG AGGAGGCTTG GGCAAAGAGC
gTvQR1_R4A  AAGACCGTTA TCGACTCTAA GTTTCCGTTG AGTAAGGCTG AGGAGGCTTG GGCAAAGAGC
gTvQR1_R6B  AAGACCGTTA TCGACTCTAA GTTTCCGTTG AGTAAGGCTG AGGAGGCTTG GGCAAAGAGC
gTvQR1_R6A  AAGACCGTTA TCGACTCTAA GTTTCCGTTG AGTAAGGCTG AGGAGGCTTG GGCAAAGAGC
gTvQR1_R5A  AAGACCGTTA TCGACTCTAA GTTTCCGTTG AGTAAGGCTG AGGAGGCTTG GGCAAAGAGC
gTvQR1_R5B  AAGACCGTTA TCGACTCTAA GTTTCCGTTG AGTAAGGCTG AGGAGGCTTG GGCAAAGAGC
gTvQR1_r8A  AAGACAGTTA TCGACTCTAA GTTTTCTTTG AGCAAGGCTG AGGAGGCTTG GGCTAAGAGC
gTvQR1_r8B  AAGACAGTTA TCGACTCTAA GTTTTCTTTG AGCAAGGCTG AGGAGGCTTG GGCTAAGAGC
gTvQR1_r5A  AAGACCGTTA TCGACTCTAA GTTTCCGTTG AGTAAGGCTG AGGAGGCTTG GGCAAAGAGC
gTvQR1_r5B  AAGACCGTTA TCGACTCTAA GTTTCCGTTG AGTAAGGCTG AGGAGGCTTG GGCAAAGAGC
gTvQR1_R2A  AAGACGGTCA TCGACTCTAA GTTTCCGTTG AGTAAGGCTG AGGAGGCTTG GGCTAAGAGC
gTvQR1_R2B  AAGACGGTCA TCGACTCTAA GTTTCCGTTG AGTAAGGCTG AGGAGGCTTG GGCTAAGAGC
gTvQR1_n1A  AAGACGGTCA TCGACTCTAA GTTTCCGTTG AGTAAGGCTG AGGAGGCTTG GGCTAAGAGC
gTvQR1_n1B  AAGACGGTCA TCGACTCTAA GTTTCCGTTG AGTAAGGCTG AGGAGGCTTG GGCTAAGAGC
gTvQR1_N2B  AAGACGGTCA TCGACTCTAA GTTTCCGTTG AGTAAGGCTG AGGAGGCTTG GGCTAAGAGC
gTvQR1_N2A  AAGACGGTCA TCGACTCTAA GTTTCCGTTG AGTAAGGCTG AGGAGGCTTG GGCTAAGAGC
gTvQR1_n2A  AAGACGGTCA TCGACTCTAA GTTTCCGTTG AGTAAGGCTG AGGAGGCTTG GGCTAAGAGC
gTvQR1_n2B  AAGACGGTCA TCGACTCTAA GTTTCCGTTG AGTAAGGCTG AGGAGGCTTG GGCTAAGAGC
gTvQR1_N3A  AAGACGGTCA TCGACTCTAA GTTTCCGTTG AGTAAGGCTG AGGAGGCTTG GGCTAAGAGC
gTvQR1_N3B  AAGACGGTCA TCGACTCTAA GTTTCCGTTG AGTAAGGCTG AGGAGGCTTG GGCTAAGAGC
gTvQR1_n3A  AAGACGGTTA TCGACTCTAA GTTTCCGTTA AGTAAGGCTG AGGAGGCTTG GGCTAAGAGC
gTvQR1_n3B  AAGACGGTTA TCGACTCTAA GTTTCCGTTA AGTAAGGCTG AGGAGGCTTG GGCTAAGAGC
```

```
              1921                                         1962
gTvQR1_r1A  ATCGACGGCC ATGCTACCGG GAAAATCGTT GTCGAGCCAT AA
gTvQR1_r1B  ATCGACGGCC ATGCTACCGG GAAAATCGTT GTCGAGCCAT AA
gTvQR1_R3A  ATCGACGGCC ATGCTACCGG GAAAATCGTT GTCGAGCCAT AA
gTvQR1_R3B  ATCGACGGCC ATGCTACCGG GAAAATCGTT GTCGAGCCAT AA
gTvQR1_N1A  ATCGACGGCC ATGCTACCGG GAAAATCGTT ATCGAGCCAT AA
gTvQR1_N1B  ATCGACGGCC ATGCTACCGG GAAAATCGTT ATCGAGCCAT AA
gTvQR1_r4A  ATCGACGGCC ATGCTACCGG GAAAATCGTT GTCGAGCCAT AA
gTvQR1_r4B  ATCGACGGCC ATGCTACCGG GAAAATCGTT GTCGAGCCAT AA
gTvQR1_r7A  ATCGACGGCC ATGCTACCGG GAAAATCGTT GTCGAGCCAT AA
gTvQR1_r7B  ATCGACGGCC ATGCTACCGG GAAAATCGTT GTCGAGCCAT AA
gTvQR1_r3A  ATCGACGGAC ATGCTACCGG GAAGATCGTT GTCGAGCCAT AA
gTvQR1_r3B  ATCGACGGAC ATGCTACCGG GAAGATCGTT GTCGAGCCAT AA
gTvQR1_r6A  ATCGACGGCC ATGCTACCGG GAAGATCGTT GTCGAGCCAT AA
gTvQR1_r6B  ATCGACGGCC ATGCTACCGG GAAGATCGTT GTCGAGCCAT AA
gTvQR1_r2A  ATCGACGGCC ATGCTACCGG GAAGATCGTT GTCGAGCCAT AA
gTvQR1_r2B  ATCGACGGCC ATGCTACCGG GAAGATCGTT GTCGAGCCAT AA
gTvQR1_R1A  ATCGACGGCC ATGCTACCGG GAAGATCGTT GTCGAGCCAT AA
gTvQR1_R1B  ATCGACGGCC ATGCTACCGG GAAGATCGTT GTCGAGCCAT AA
gTvQR1_R4B  ATCGACGGCC ATGCTACCGG GAAGATCGTT GTCGAGCCAT AA
gTvQR1_R4A  ATCGACGGCC ATGCTACCGG GAAGATCGTT GTCGAGCCAT AA
gTvQR1_R6B  ATCGACGGCC ATGCTACCGG GAAGATCGTT GTCGAGCCAT AA
gTvQR1_R6A  ATCGACGGCC ATGCTACCGG GAAGATCGTT GTCGAGCCAT AA
gTvQR1_R5A  ATCGACGGCC ATGCTACCGG GAAGATCGTT GTCGAGCCAT AA
gTvQR1_R5B  ATCGACGGCC ATGCTACCGG GAAGATCGTT GTCGAGCCAT AA
gTvQR1_r8A  ATCGACGGCC ATGCTACCGG GAAGATCGTT GTCGAGCCAT AA
gTvQR1_r8B  ATCGACGGCC ATGCTACCGG GAAGATCGTT GTCGAGCCAT AA
gTvQR1_r5A  ATCGACGGCC ATGCTACCGG GAAGATCGTT GTCGAGCCAT AA
gTvQR1_r5B  ATCGACGGCC ATGCTACCGG GAAGATCGTT GTCGAGCCAT AA
gTvQR1_R2A  ATCGACGGCC ATGCTACCGG GAAAATCGTT GTCGAGCCAT AA
gTvQR1_R2B  ATCGACGGCC ATGCTACCGG GAAAATCGTT GTCGAGCCAT AA
gTvQR1_n1A  ATCGACGGCC ATGCTACCGG GAAAATCGTT GTCGAGCCAT AA
gTvQR1_n1B  ATCGACGGCC ATGCTACCGG GAAAATCGTT GTCGAGCCAT AA
gTvQR1_N2B  ATCGACGGCC ATGCTACCGG GAAAATCGTT GTCGAGCCAT AA
gTvQR1_N2A  ATCGACGGCC ATGCTACCGG GAAAATCGTT GTCGAGCCAT AA
gTvQR1_n2A  ATCGACGGCC ATGCTACCGG GAAAATCGTT GTCGAGCCAT AA
gTvQR1_n2B  ATCGACGGCC ATGCTACCGG GAAAATCGTT GTCGAGCCAT AA
gTvQR1_N3A  ATCGACGGCC ATGCTACCGG GAAAATCGTT GTCGAGCCAT AA
gTvQR1_N3B  ATCGACGGCC ATGCTACCGG GAAAATCGTT GTCGAGCCAT AA
gTvQR1_n3A  ATCGACGGCC ATGCTACCGG GAAAATCGTT GTCGAGCCAT AA
gTvQR1_n3B  ATCGACGGCC ATGCTACCGG GAAAATCGTT GTCGAGCCAT AA
```
