# Supplementary material for: The differentially regulated genes TvQR1 and TvPirin of the parasitic plant Triphysaria exhibit distinctive natural allelic diversity
Source: BMC Plant Biol. 2013 Feb 18;13:28. doi: 10.1186/1471-2229-13-28 (PMC3599707; doi:10.1186/1471-2229-13-28)
Supplement: Additional file 6 — Multiple sequence alignment of all genomic TvPirin alleles. [file 1471-2229-13-28-S6.pdf]

Additional file 6. Multiple sequence alignment of all genomic *TvPirin* alleles

Two alleles of each plant are designated as “A” and “B”. Exon: upper case letters, intron: lower case letters. R: responsive to DMBQ, r: responsive to peonidin, N: non-responsive to DMBQ, n: non-responsive to peonidine.

```
            1                                                          60
gTvPir_r1A  ATGTCCAGTT TCCAACAGCC CAGACTAGTT GCCAAGAAGA TCTTGGCCAA GTCCCAGTCC
gTvPir_r2B  ATGTCCAGTT TCCAACAGCC CAGACTAGTT GCCAAGAAGA TCTTGGCCAA GTCCCAGTCC
gTvPir_r3B  ATGTCCAGTT TCCAACAGCC CAGACTAGTT GCCAAGAAGA TCTTGGCCAA GTCCCAGTCC
gTvPir_r5A  ATGTCCAGTT TCCAACAGCC CAGACTAGTT GCCAAGAAGA TCTTGGCCAA GTCCCAGTCC
gTvPir_r8A  ATGTCCAGTT TCCAACAGCC CAGACTAGTT GCCAAGAAGA TCTTGGCCAA GTCCCAGTCC
gTvPir_r8B  ATGTCCAGTT TCCAACAGCC CAGACTAGTT GCCAAGAAGA TCTTGGCCAA GTCCCAGTCC
gTvPir_R1A  ATGTCCAGTT TCCAACAGCC CAGACTAGTT GCCAAGAAGA TCTTGGCCAA GTCCCAGTCC
gTvPir_n1A  ATGTCCAGTT TCCAACAGCC CAGACTAGTT GCCAAGAAGA TCTTGGCCAA GTCCCAGTCC
gTvPir_n2A  ATGTCCAGTT TCCAACAGCC CAGACTAGTT GCCAAGAAGA TCTTGGCCAA GTCCCAGTCC
gTvPir_n1B  ATGTCCAGTT TCCAACAGCC CAGACTAGTT GCCAAGAAGA TCTTGGCCAA GTCCCAGTCC
gTvPir_r2A  ATGTCCAGTT TCCAACAGCC CAGACTAGTT GCCAAGAAGA TCTTGGCCAA GTCCCAGTCC
gTvPir_r3A  ATGTCCAGTT TCCAACAGCC CAGACTAGTT GCCAAGAAGA TCTTGGCCAA GTCCCAGTCC
gTvPir_r5B  ATGTCCAGTT TCCAACAGCC CAGACTAGTT GCCAAGAAGA TCTTGGCCAA GTCCCAGTCC
gTvPir_R1B  ATGTCCAGTT TCCAACAGCC CAGACTAGTT GCCAAGAAGA TCTTGGCCAA GTCCCAGTCC
gTvPir_r7A  ATGTCCAGTT TCCAACAGCC CAGACTAGTT GCCAAGAAGA TCTTGGCCAA GTCCCAGTCC
gTvPir_r1B  ATGTCCAGTT TCCAACAGCC CAGACTAGTT GCCAAGAAGA TCTTGGCCAA GTCCCAGTCC
gTvPir_R6B  ATGTCCAGTT TCCAACAGCC CAGACTAGTT GCCAAGAAGA TCTTGGCCAA GTCCCAGTCC
gTvPir_R4B  ATGTCCAGTT TCCAACAGCC CAGACTAGTT GCCAAGAAGA TCTTGGCCAA GTCCCAGTCC
gTvPir_R6A  ATGTCCAGTT TCCAACAGCC CAGACTAGTT GCCAAGAAGA TCTTGGCCAA GTCCCAGTCC
gTvPir_N2A  ATGTCCAGTT TCCAACAGCC CAGACTAGTT GCCAAGAAGA TCTTGGCCAA GTCCCAGTCC
gTvPir_N2B  ATGTCCAGTT TCCAACAGCC CAGACTAGTT GCCAAGAAGA TCTTGGCCAA GTCCCAGTCC
gTvPir_R2A  ATGTCCAGTT TCCAACAGCC CAGACTAGTT GCCAAGAAGA TCTTGGCCAA GTCCCAGTCC
gTvPir_N1B  ATGTCCAGTT TCCAACAGCC CAGACTAGTT GCCAAGAAGA TCTTGGCCAA GTCCCAGTCC
gTvPir_R4A  ATGTCCAGTT TCCAACAGCC CAGACTAGTT GCCAAGAAGA TCTTGGCCAA GTCCCAGTCC
gTvPir_n3A  ATGTCCAGTT TCCAACAGCC CAGACTAGTT GCCAAGAAGA TCTTGGCCAA GTCCCAGTCC
gTvPir_N1A  ATGTCCAGTT TCCAACAGCC CAGACTAGTT GCCAAGAAGA TCTTGGCCAA GTCCCAGTCC
gTvPir_n3B  ATGTCCAGTT TCCAACAGCC CAGACTAGTT GCCAAGAAGA TCTTGGCCAA GTCCCAGTCC
gTvPir_N3B  ATGTCCAGTT TCCAACAGCC CAGACTAGTT GCCAAGAAGA TCTTGGCCAA GTCCCAGTCC
gTvPir_r4B  ATGTCCAGTT TCCAACAGCC CAGACTAGTT GCCAAGAAGA TCTTGGCCAA GTCCCAGTCC
gTvPir_r6A  ATGTCCAGTT TCCAACAGCC CAGACTAGTT GCCAAGAAGA TCTTGGCCAA GTCCCAGTCC
gTvPir_R3A  ATGTCCAGTT TCCAACAGCC CAGACTAGTT GCCAAGAAGA TCTTGGCCAA GTCCCAGTCC
gTvPir_N3A  ATGTCCAGTT TCCAACAGCC CAGACTAGTT GCCAAGAAGA TCTTGGCCAA GTCCCAGTCC
gTvPir_r6B  ATGTCCAGTT TCCAACAGCC CAGACTAGTT GCCAAGAAGA TCTTGGCCAA GTCCCAGTCC
gTvPir_R5A  ATGTCCAGTT TCCAACAGCC CAGACTAGTT GCCAAGAAGA TCTTGGCCAA GTCCCAGTCC
gTvPir_r7B  ATGTCCAGTT TCCAACAGCC CAGACTAGTT GCCAAGAAGA TCTTGGCCAA GTCCCAGTCC
gTvPir_R2B  ATGTCCAGTT TCCAACAGCC CAGACTAGTT GCCAAGAAGA TCTTGGCCAA GTCCCAGTCC
gTvPir_R3B  ATGTCCAGTT TCCAACAGCC CAGACTAGTT GCCAAGAAGA TCTTGGCCAA GTCCCAGTCC
gTvPir_r4A  ATGTCCAGTT TCCAACAGCC CAGACTAGTT GCCAAGAAGA TCTTGGCCAA GTCCCAGTCC
gTvPir_R5B  ATGTCCAGTT TCCAACAGCC CAGACTAGTT GCCAAGAAGA TCTTGGCCAA GTCCCAGTCC
gTvPir_n2B  ATGTCCAGTT TCCAACAGCC CAGACTAGTT GCCAAGAAGA TCTTGGCCAA GTCCCAGTCC
 Consensus  ATGTCCAGTT TCCAACAGCC CAGACTAGTT GCCAAGAAGA TCTTGGCCAA GTCCCAGTCC
```

```
            61                                                        120
gTvPir_r1A  GAGGGCGATG GTGCACTTGT AAGAAGAAGC ATTGGCAGgt ttttcttttt cctttttttc
gTvPir_r2B  GAGGGCGATG GTGCACTTGT AAGAAGAAGC ATTGGCAGgt ttttcttttt cctttttttc
gTvPir_r3B  GAGGGCGATG GTGCACTTGT AAGAAGAAGC ATTGGCAGgt ttttcttttt cctttttttc
gTvPir_r5A  GAGGGCGATG GTGCACTTGT AAGAAGAAGC ATTGGCAGgt ttttcttttt cctttttttc
gTvPir_r8A  GAGGGCGATG GTGCACTTGT AAGAAGAAGC ATTGGCAGgt ttttcttttt cctttttttc
gTvPir_r8B  GAGGGCGATG GTGCACTTGT AAGAAGAAGC ATTGGCAGgt ttttcttttt cctttttttc
gTvPir_R1A  GAGGGCGATG GTGCACTTGT AAGAAGAAGC ATTGGCAGgt ttttcttttt cctttttttc
gTvPir_n1A  GAGGGCGATG GTGCACTTGT AAGAAGAAGC ATTGGCAGgt ttttcttttt cctttttttc
gTvPir_n2A  GAGGGCGATG GTGCACTTGT AAGAAGAAGC ATTGGCAGgt ttttcttttt cctttttttc
gTvPir_n1B  GAGGGCGATG GTGCACTTGT AAGAAGAAGC ATTGGCAGgt ttttcttttt cctttttttc
gTvPir_r2A  GAGGGCGATG GTGCACTTGT AAGAAGAAGC ATTGGCAGgt ttttcttttt cctttttttc
gTvPir_r3A  GAGGGCGATG GTGCACTTGT AAGAAGAAGC ATTGGCAGgt ttttcttttt cctttttttc
gTvPir_r5B  GAGGGCGATG GTGCACTTGT AAGAAGAAGC ATTGGCAGgt ttttcttttt cctttttttc
gTvPir_R1B  GAGGGCGATG GTGCACTTGT AAGAAGAAGC ATTGGCAGgt ttttcttttt cctttttttc
gTvPir_r7A  GAGGGCGATG GTGCACTTGT AAGAAGAAGC ATTGGCAGgt ttttcttttt cctttttttc
gTvPir_r1B  GAGGGCGATG GTGCACTTGT AAGAAGAAGC ATTGGCAGgt ttttcttttt cctttttttc
gTvPir_R6B  GAGGGCGATG GTGCACTTGT AAGAAGAAGC ATTGGCAGgt ttttcttttt cctttttttc
gTvPir_R4B  GAGGGCGATG GTGCACTTGT AAGAAGAAGC ATTGGCAGgt ttttcttttt cctttttttc
gTvPir_R6A  GAGGGCGATG GTGCACTTGT AAGAAGAAGC ATTGGCAGgt ttttcttttt cctttttttc
gTvPir_N2A  GAGGGCGATG GTGCACTTGT AAGAAGAAGC ATTGGCAGgt ttttcttttt cctttttttc
gTvPir_N2B  GAGGGCGATG GTGCACTTGT AAGAAGAAGC ATTGGCAGgt ttttcttttt cctttttttc
gTvPir_R2A  GAGGGCGATG GTGCACTTGT AAGAAGAAGC ATTGGCAGgt ttttcttttt cctttttttc
gTvPir_N1B  GAGGGCGATG GTGCACTTGT AAGAAGAAGC ATTGGCAGgt ttttcttttt cctttttttc
gTvPir_R4A  GAGGGCGATG GTGCACTTGT AAGAAGAAGC ATTGGCAGgt ttttcttttt cctttttttc
gTvPir_n3A  GAGGGCGATG GTGCACTTGT AAGAAGAAGC ATTGGCAGgt ttttcttttt cctttttttc
gTvPir_N1A  GAGGGCGATG GTGCACTTGT AAGAAGAAGC ATTGGCAGgt ttttcttttt cctttttttc
gTvPir_n3B  GAGGGCGATG GTGCACTTGT AAGAAGAAGC ATTGGCAGgt ttttcttttt cctttttttc
gTvPir_N3B  GAGGGCGATG GTGCACTTGT AAGAAGAAGC ATTGGCAGgt ttttcttttt cctttttttc
gTvPir_r4B  GAGGGCGATG GTGCACTTGT AAGAAGAAGC ATTGGCAGgt ttttcttttt cctttttttc
gTvPir_r6A  GAGGGCGATG GTGCACTTGT AAGAAGAAGC ATTGGCAGgt ttttcttttt cctttttttc
gTvPir_R3A  GAGGGCGATG GTGCACTTGT AAGAAGAAGC ATTGGCAGgt ttttcttttt cctttttttc
gTvPir_N3A  GAGGGCGATG GTGCACTTGT AAGAAGAAGC ATTGGCAGgt ttttcttttt cctttttttc
gTvPir_r6B  GAGGGCGATG GTGCACTTGT AAGAAGAAGC ATTGGCAGgt ttttcttttt cctttttttc
gTvPir_R5A  GAGGGCGATG GTGCACTTGT AAGAAGAAGC ATTGGCAGgt ttttcttttt cctttttttc
gTvPir_r7B  GAGGGCGATG GTGCACTTGT AAGAAGAAGC ATTGGCAGgt ttttcttttt cctttttttc
gTvPir_R2B  GAGGGCGATG GTGCACTTGT AAGAAGAAGC ATTGGCAGgt ttttcttttt cctttttttc
gTvPir_R3B  GAGGGCGATG GTGCACTTGT AAGAAGAAGC ATTGGCAGgt ttttcttttt cctttttttc
gTvPir_r4A  GAGGGCGATG GTGCACTTGT AAGAAGAAGC ATTGGCAGgt ttttcttttt cctttttttc
gTvPir_R5B  GAGGGCGATG GTGCACTTGT AAGAAGAAGC ATTGGCAGgt ttttcttttt cctttttttc
gTvPir_n2B  GAGGGCGATG GTGCACTTGT AAGAAGAAGC ATTGGCAGgt ttttcttttt cctttttttc
```

```
             121                                                     180
gTvPir_r1A  ttcatatttt tgtgtcttgt attttagatt tcttctccac ttaaattggg attttgaatt
gTvPir_r2B  ttcatatttt tgtgtcttgt attttagatt tcttctccac ttaaattggg attttgaatt
gTvPir_r3B  ttcatatttt tgtgtcttgt attttagatt tcttctccac ttaaattggg attttgaatt
gTvPir_r5A  ttcatatttt tgtgtcttgt attttagatt tcttctccac ttaaattggg attttgaatt
gTvPir_r8A  ttcatatttt tgtgtcttgt attttagatt tcttctccac ttaaattggg attttgaatt
gTvPir_r8B  ttcatatttt tgtgtcttgt attttagatt tcttctccac ttaaattggg attttgaatt
gTvPir_R1A  ttcatatttt tgtgtcttgt attttagatt tcttctccac ttaaattggg attttgaatt
gTvPir_n1A  ttcatatttt tgtgtcttgt attttagatt tcttctccac ttaaattggg attttgaatt
gTvPir_n2A  ttcatatttt tgtgtcttgt attttagatt tcttctccac ttaaattggg attttgaatt
gTvPir_n1B  ttcatatttt tgtgtcttgt attttagatt tcttctccac ttaaattggg attttgaatt
gTvPir_r2A  ttcatatttt tgtgtcttgt attttagatt tcttctccac ttaaattggg attttgaatt
gTvPir_r3A  ttcatatttt tgtgtcttgt attttagatt tcttctccac ttaaattggg attttgaatt
gTvPir_r5B  ttcatatttt tgtgtcttgt attttagatt tcttctccac ttaaattggg attttgaatt
gTvPir_R1B  ttcatatttt tgtgtcttgt attttagatt tcttctccac ttaaattggg attttgaatt
gTvPir_r7A  ttcatatttt tgtgtcttgt attttagatt tcttctccac ttaaattggg attttgaatt
gTvPir_r1B  ttcatatttt tgtgtcttgt attttagatt tcttctccac ttaaattggg attttgaatt
gTvPir_R6B  ttcatatttt tgtgtcttgt attttagatt tcttctccac ttaaattggg attttgaatt
gTvPir_R4B  ttcatatttt tgtgtcttgt attttagatt tcttctccac ttaaattggg attttgaatt
gTvPir_R6A  ttcatatttt tgtgtcttgt attttagatt tcttctccac ttaaattggg attttgaatt
gTvPir_N2A  ttcatatttt tgtgtcttgt attttagatt tcttctccac ttaaattggg attttgaatt
gTvPir_N2B  ttcatatttt tgtgtcttgt attttagatt tcttctccac ttaaattggg attttgaatt
gTvPir_R2A  ttcatatttt tgtgtcttgt attttagatt tcttctccac ttaaattggg attttgaatt
gTvPir_N1B  ttcatatttt tgtgtcttgt attttagatt tcttctccac ttaaattggg attttgaatt
gTvPir_R4A  ttcatatttt tgtgtcttgt attttagatt tcttctccac ttaaattggg attttgaatt
gTvPir_n3A  ttcatatttt tgtgtcttgt attttagatt tcttctccac ttaaattggg attttgaatt
gTvPir_N1A  ttcatatttt tgtgtcttgt attttagatt tcttctccac ttaaattggg attttgaatt
gTvPir_n3B  ttcatatttt tgtgtcttgt attttagatt tcttctccac ttaaattggg attttgaatt
gTvPir_N3B  ttcatatttt tgtgtcttgt attttagatt tcttctccac ttaaattggg attttgaatt
gTvPir_r4B  ttcatatttt tgtgtcttgt attttagatt tcttctccac ttaaattggg attttgaatt
gTvPir_r6A  ttcatatttt tgtgtcttgt attttagatt tcttctccac ttaaattggg attttgaatt
gTvPir_R3A  ttcatatttt tgtgtcttgt attttagatt tcttctccac ttaaattggg attttgaatt
gTvPir_N3A  ttcatatttt tgtgtcttgt attttagatt tcttctccac ttaaattggg attttgaatt
gTvPir_r6B  ttcatatttt tgtgtcttgt attttagatt tcttctccac ttaaattggg attttgaatt
gTvPir_R5A  ttcatatttt tgtgtcttgt attttagatt tcttctccac ttaaattggg attttgaatt
gTvPir_r7B  ttcatatttt tgtgtcttgt attttagatt tcttctccac ttaaattggg attttgaatt
gTvPir_R2B  ttcatatttt tgtgtcttgt attttagatt tcttctccac ttaaattggg attttgaatt
gtvpir_R3B  ttcatatttt tgtgtcttgt attttagatt tcttctccac ttaaattggg attttgaatt
gtvpir_r4A  ttcatatttt tgtgtcttgt attttagatt tcttctccac ttaaattggg attttgaatt
gtvpir_R5B  ttcatatttt tgtgtcttgt attttagatt tcttctccac ttaaattggg attttgaatt
gTvPir_n2B  ttcatatttt tgtgtcttgt attttagatt tcttctccac ttaaattggg attttgaatt
```

```
            181                                                        240
gTvPir_r1A  ggaagtcaat tagatttcta gttccaattt ggattggagt tagttttggt gttttggaga
gTvPir_r2B  ggaagtcaat tagatttcta gttccaattt ggattggagt tagttttggt gttttggaga
gTvPir_r3B  ggaagtcaat tagatttcta gttccaattt ggattggagt tagttttggt gttttggaga
gTvPir_r5A  ggaagtcaat tagatttcta gttccaattt ggattggagt tagttttggt gttttggaga
gTvPir_r8A  ggaagtcaat tagatttcta gttccaattt ggattggagt tagttttggt gttttggaga
gTvPir_r8B  ggaagtcaat tagatttcta gttccaattt ggattggagt tagttttggt gttttggaga
gTvPir_R1A  ggaagtcaat tagatttcta gttccaattt ggattggagt tagttttggt gttttggaga
gTvPir_n1A  ggaagtcaat tagatttcta gttccaattt ggattggagt tagttttggt gttttggaga
gTvPir_n2A  ggaagtcaat tagatttcta gttccaattt ggattggagt tagttttggt gttttggaga
gTvPir_n1B  ggaagtcaat tagatttcta gttccaattt ggattggagt tagttttggt gttttggaga
gTvPir_r2A  ggaagtcaat tagatttcta gttccaattt ggattggagt tagttttggt gttttggaga
gTvPir_r3A  ggaagtcaat tagatttcta gttccaattt ggattggagt tagttttggt gttttggaga
gTvPir_r5B  ggaagtcaat tagatttcta gttccaattt ggattggagt tagttttggt gttttggaga
gTvPir_R1B  ggaagtcaat tagatttcta gttccaattt ggattggagt tagttttggt gttttggaga
gTvPir_r7A  ggaagtcaat tagatttcta gttccaattt ggattggagt tagttttggt gttttggaga
gTvPir_r1B  ggaagtcaat tagatttcta gttccaattt ggattggagt tagttttggt gttttggaga
gTvPir_R6B  ggaagtcaat tagatttcta gttccaattt ggattggagt tagttttggt gttttggaga
gTvPir_R4B  ggaagtcaat tagatttcta gttccaattt ggattggagt tagttttggt gttttggaga
gTvPir_R6A  ggaagtcaat tagatttcta gttccaattt ggattggagt tagttttggt gttttggaga
gTvPir_N2A  ggaagtcaat tagatttcta gttccaattt ggattggagt tagttttggt gttttggaga
gTvPir_N2B  ggaagtcaat tagatttcta gttccaattt ggattggagt tagttttggt gttttggaga
gTvPir_R2A  ggaagtcaat tagatttcta gttccaattt ggattggagt tagttttggt gttttggaga
gTvPir_N1B  ggaagtcaat tagatttcta gttccaattt ggattggagt tagttttggt gttttggaga
gTvPir_R4A  ggaagtcaat tagatttcta gttccaattt ggattggagt tagttttggt gttttggaga
gTvPir_n3A  ggaagtcaat tagatttcta gttccaattt ggattggagt tagttttggt gttttggaga
gTvPir_N1A  ggaagtcaat tagatttcta gttccaattt ggattggagt tagttttggt gttttggaga
gTvPir_n3B  ggaagtcaat tagatttcta gttccaattt ggattggagt tagttttggt gttttggaga
gTvPir_N3B  ggaagtcaat tagatttcta gttccaattt ggattggagt tagttttggt gttttggaga
gTvPir_r4B  ggaagtcaat tagatttcta gttccaattt ggattggagt tagttttggt gttttggaga
gTvPir_r6A  ggaagtcaat tagatttcta gttccaattt ggattggagt tagttttggt gttttggaga
gTvPir_R3A  ggaagtcaat tagatttcta gttccaattt ggattggagt tagttttggt gttttggaga
gTvPir_N3A  ggaagtcaat tagatttcta gttccaattt ggattggagt tagttttggt gttttggaga
gTvPir_r6B  ggaagtcaat tagatttcta gttccaattt ggattggagt tagttttggt gttttggaga
gTvPir_R5A  ggaagtcaat tagatttcta gttccaattt ggattggagt tagttttggt gttttggaga
gTvPir_r7B  ggaagtcaat tagatttcta gttccaattt ggattggagt tagttttggt gttttggaga
gTvPir_R2B  ggaagtcaat tagatttcta gttccaattt ggattggagt tagttttggt gttttggaga
gTvPir_R3B  ggaagtcaat tagatttcta gttccaattt ggattggagt tagttttggt gttttggaga
gTvPir_r4A  ggaagtcaat tagatttcta gttccaattt ggattggagt tagttttggt gttttggaga
gTvPir_R5B  ggaagtcaat tagatttcta gttccaattt ggattggagt tagttttggt gttttggaga
gTvPir_n2B  ggaagtcaat tagatttcta gttccaattt ggattggagt tagttttggt gttttggaga
```

```
            241                                                     300
gTvPir_r1A  ataaatttgg ggcaatgaat tttgcagGCC TGAGTTGAAG TCCCTAGATC CATTTCTCTT
gTvPir_r2B  ataaatttgg ggcaatgaat tttgcagGCC TGAGTTGAAG TCCCTAGATC CATTTCTCTT
gTvPir_r3B  ataaatttgg ggcaatgaat tttgcagGCC TGAGTTGAAG TCCCTAGATC CATTTCTCTT
gTvPir_r5A  ataaatttgg ggcaatgaat tttgcagGCC TGAGTTGAAG TCCCTAGATC CATTTCTCTT
gTvPir_r8A  ataaatttgg ggcaatgaat tttgcagGCC TGAGTTGAAG TCCCTAGATC CATTTCTCTT
gTvPir_r8B  ataaatttgg ggcaatgaat tttgcagGCC TGAGTTGAAG TCCCTAGATC CATTTCTCTT
gTvPir_R1A  ataaatttgg ggcaatgaat tttgcagGCC TGAGTTGAAG TCCCTAGATC CATTTCTCTT
gTvPir_n1A  ataaatttgg ggcaatgaat tttgcagGCC TGAGTTGAAG TCCCTAGATC CATTTCTCTT
gTvPir_n2A  ataaatttgg ggcaatgaat tttgcagGCC TGAGTTGAAG TCCCTAGATC CATTTCTCTT
gTvPir_n1B  ataaatttgg ggcaatgaat tttgcagGCC TGAGTTGAAG TCCCTAGATC CATTTCTCTT
gTvPir_r2A  ataaatttgg ggcaatgaat tttgcagGCC TGAGTTGAAG TCCCTAGATC CATTTCTCTT
gTvPir_r3A  ataaatttgg ggcaatgaat tttgcagGCC TGAGTTGAAG TCCCTAGATC CATTTCTCTT
gTvPir_r5B  ataaatttgg ggcaatgaat tttgcagGCC TGAGTTGAAG TCCCTAGATC CATTTCTCTT
gTvPir_R1B  ataaatttgg ggcaatgaat tttgcagGCC TGAGTTGAAG TCCCTAGATC CATTTCTCTT
gTvPir_r7A  ataaatttgg ggcaatgaat tttgcagGCC TGAGTTGAAG TCCCTAGATC CATTTCTCTT
gTvPir_r1B  ataaatttgg ggcaatgaat tttgcagGCC TGAGTTGAAG TCCCTAGATC CATTTCTCTT
gTvPir_R6B  ataaatttgg ggcaatgaat tttgcagGCC TGAGTTGAAG TCCCTAGATC CATTTCTCTT
gTvPir_R4B  ataaatttgg ggcaatgaat tttgcagGCC TGAGTTGAAG TCCCTAGATC CATTTCTCTT
gTvPir_R6A  ataaatttgg ggcaatgaat tttgcagGCC TGAGTTGAAG TCCCTAGATC CATTTCTCTT
gTvPir_N2A  ataaatttgg ggcaatgaat tttgcagGCC TGAGTTGAAG TCCCTAGATC CATTTCTCTT
gTvPir_N2B  ataaatttgg ggcaatgaat tttgcagGCC TGAGTTGAAG TCCCTAGATC CATTTCTCTT
gTvPir_R2A  ataaatttgg ggcaatgaat tttgcagGCC TGAGTTGAAG TCCCTAGATC CATTTCTCTT
gTvPir_N1B  ataaatttgg ggcaatgaat tttgcagGCC TGAGTTGAAG TCCCTAGATC CATTTCTCTT
gTvPir_R4A  ataaatttgg ggcaatgaat tttgcagGCC TGAGTTGAAG TCCCTAGATC CATTTCTCTT
gTvPir_n3A  ataaatttgg ggcaatgaat tttgcagGCC TGAGTTGAAG TCCCTAGATC CATTTCTCTT
gTvPir_N1A  ataaatttgg ggcaatgaat tttgcagGCC TGAGTTGAAG TCCCTAGATC CATTTCTCTT
gTvPir_n3B  ataaatttgg ggcaatgaat tttgcagGCC TGAGTTGAAG TCCCTAGATC CATTTCTCTT
gTvPir_N3B  ataaatttgg ggcaatgaat tttgcagGCC TGAGTTGAAG TCCCTAGATC CATTTCTCTT
gTvPir_r4B  ataaatttgg ggcaatgaat tttgcagGCC TGAGTTGAAG TCCCTAGATC CATTTCTCTT
gTvPir_r6A  ataaatttgg ggcaatgaat tttgcagGCC TGAGTTGAAG TCCCTAGATC CATTTCTCTT
gTvPir_R3A  ataaatttgg ggcaatgaat tttgcagGCC TGAGTTGAAG TCCCTAGATC CATTTCTCTT
gTvPir_N3A  ataaatttgg ggcaatgaat tttgcagGCC TGAGTTGAAG TCCCTAGATC CATTTCTCTT
gTvPir_r6B  ataaatttgg ggcaatgaat tttgcagGCC TGAGTTGAAG TCCCTAGATC CATTTCTCTT
gTvPir_R5A  ataaatttgg ggcaatgaat tttgcagGCC TGAGTTGAAG TCCCTAGATC CATTTCTCTT
gTvPir_r7B  ataaatttgg ggcaatgaat tttgcagGCC TGAGTTGAAG TCCCTAGATC CATTTCTCTT
gTvPir_R2B  ataaatttgg ggcaatgaat tttgcagGCC TGAGTTGAAG TCCCTAGATC CATTTCTCTT
gTvPir_R3B  ataaatttgg ggcaatgaat tttgcagGCC TGAGTTGAAG TCCCTAGATC CATTTCTCTT
gTvPir_r4A  ataaatttgg ggcaatgaat tttgcagGCC TGAGTTGAAG TCCCTAGATC CATTTCTCTT
gTvPir_R5B  ataaatttgg ggcaatgaat tttgcagGCC TGAGTTGAAG TCCCTAGATC CATTTCTCTT
gTvPir_n2B  ataaatttgg ggcaatgaat tttgcagGCC TGAGTTGAAG TCCCTAGATC CATTTCTCTT
```

```
             301                                                        360
gTvPir_r1A  GCTAGATGAA TTTGCTGgta acacttataa ttatacctgt aaaaaagagt aatttgcaaa
gTvPir_r2B  GCTAGATGAA TTTGCTGgta acacttataa ttatacctgt aaaaaagagt aatttgcaaa
gTvPir_r3B  GCTAGATGAA TTTGCTGgta acacttataa ttatacctgt aaaaaagagt aatttgcaaa
gTvPir_r5A  GCTAGATGAA TTTGCTGgta acacttataa ttatacctgt aaaaaagagt aatttgcaaa
gTvPir_r8A  GCTAGATGAA TTTGCTGgta acacttataa ttatacctgt aaaaaagagt aatttgcaaa
gTvPir_r8B  GCTAGATGAA TTTGCTGgta acacttataa ttatacctgt aaaaaagagt aatttgcaaa
gTvPir_R1A  GCTAGATGAA TTTGCTGgta acacttataa ttatacctgt aaaaaagagt aatttgcaaa
gTvPir_n1A  GCTAGATGAA TTTGCTGgta acacttataa ttatacctgt aaaaaagagt aatttgcaaa
gTvPir_n2A  GCTAGATGAA TTTGCTGgta acacttataa ttatacctgt aaaaaagagt aatttgcaaa
gTvPir_n1B  GCTAGATGAA TTTGCTGgta acacttataa ttatacctgt aaaaaagagt aatttgcaaa
gTvPir_r2A  GCTAGATGAA TTTGCTGgta acacttataa ttatacctgt aaaaaagagt aatttgcaaa
gTvPir_r3A  GCTAGATGAA TTTGCTGgta acacttataa ttatacctgt aaaaaagagt aatttgcaaa
gTvPir_r5B  GCTAGATGAA TTTGCTGgta acacttataa ttatacctgt aaaaaagagt aatttgcaaa
gTvPir_R1B  GCTAGATGAA TTTGCTGgta acacttataa ttatacctgt aaaaaagagt aatttgcaaa
gTvPir_r7A  GCTAGATGAA TTTGCTGgta acacttataa ttatacctgt aaaaaagagt aatttgcaaa
gTvPir_r1B  GCTAGATGAA TTTGCTGgta acacttataa ttatacctgt aaaaaagagt aatttgcaaa
gTvPir_R6B  GCTAGATGAA TTTGCTGgta acacttataa ttatacctgt aaaaaagagt aatttgcaaa
gTvPir_R4B  GCTAGATGAA TTTGCTGgta acacttataa ttatacctgt aaaaaagagt aatttgcaaa
gTvPir_R6A  GCTAGATGAA TTTGCTGgta acacttataa ttatacctgt aaaaaagagt aatttgcaaa
gTvPir_N2A  GCTAGATGAA TTTGCTGgta acacttataa ttatacctgt aaaaaagagt aatttgcaaa
gTvPir_N2B  GCTAGATGAA TTTGCTGgta acacttataa ttatacctgt aaaaaagagt aatttgcaaa
gTvPir_R2A  GCTAGATGAA TTTGCTGgta acacttataa ttatacctgt aaaaaagagt aatttgcaaa
gTvPir_N1B  GCTAGATGAA TTTGCTGgta acacttataa ttatacctgt aaaaaagagt aatttgcaaa
gTvPir_R4A  GCTAGATGAA TTTGCTGgta acacttataa ttatacctgt aaaaaagagt aatttgcaaa
gTvPir_n3A  GCTAGATGAA TTTGCTGgta acacttataa ttatacctgt aaaaaagagt aatttgcaaa
gTvPir_N1A  GCTAGATGAA TTTGCTGgta acacttataa ttatacctgt aaaaaagagt aatttgcaaa
gTvPir_n3B  GCTAGATGAA TTTGCTGgta acacttataa ttatacctgt aaaaaagagt aatttgcaaa
gTvPir_N3B  GCTAGATGAA TTTGCTGgta acacttataa ttatacctgt aaaaaagagt aatttgcaaa
gTvPir_r4B  GCTAGATGAA TTTGCTGgta acacttataa ttatacctgt aaaaaagagt aatttgcaaa
gTvPir_r6A  GCTAGATGAA TTTGCTGgta acacttataa ttatacctgt aaaaaagagt aatttgcaaa
gTvPir_R3A  GCTAGATGAA TTTGCTGgta acacttataa ttatacctgt aaaaaagagt aatttgcaaa
gTvPir_N3A  GCTAGATGAA TTTGCTGgta acacttataa ttatacctgt aaaaaagagt aatttgcaaa
gTvPir_r6B  GCTAGATGAA TTTGCTGgta acacttataa ttatacctgt aaaaaagagt aatttgcaaa
gTvPir_R5A  GCTAGATGAA TTTGCTGgta acacttataa ttatacctgt aaaaaagagt aatttgcaaa
gTvPir_r7B  GCTAGATGAA TTTGCTGgta acacttataa ttatacctgt aaaaaagagt aatttgcaaa
gTvPir_R2B  GCTAGATGAA TTTGCTGgta acacttataa ttatacctgt aaaaaagagt aatttgcaaa
gTvPir_R3B  GCTAGATGAA TTTGCTGgta acacttataa ttatacctgt aaaaaagagt aatttgcaaa
gTvPir_r4A  GCTAGATGAA TTTGCTGgta acacttataa ttatacctgt aaaaaagagt aatttgcaaa
gTvPir_R5B  GCTAGATGAA TTTGCTGgta acacttataa ttatacctgt aaaaaagagt aatttgcaaa
gTvPir_n2B  GCTAGATGAA TTTGCTGgta acacttataa ttatacctgt aaaaaagagt aatttgcaaa
```

```
            361                                                   420
gTvPir_r1A  taacaccctg gtgtaatgtc aaactagcag aaagccaccg tgaaaaataa actagcaaat
gTvPir_r2B  taacaccctg gtgtaatgtc aaactagcag aaagccaccg tgaaaaataa actagcaaat
gTvPir_r3B  taacaccctg gtgtaatgtc aaactagcag aaagccaccg tgaaaaataa actagcaaat
gTvPir_r5A  taacaccctg gtgtaatgtc aaactagcag aaagccaccg tgaaaaataa actagcaaat
gTvPir_r8A  taacaccctg gtgtaatgtc aaactagcag aaagccaccg tgaaaaataa actagcaaat
gTvPir_r8B  taacaccctg gtgtaatgtc aaactagcag aaagccaccg tgaaaaataa actagcaaat
gTvPir_R1A  taacaccctg gtgtaatgtc aaactagcag aaagccaccg tgaaaaataa actagcaaat
gTvPir_n1A  taacaccctg gtgtaatgtc aaactagcag aaagccaccg tgaaaaataa actagcaaat
gTvPir_n2A  taacaccctg gtgtaatgtc aaactagcag aaagccaccg tgaaaaataa actagcaaat
gTvPir_n1B  taacaccctg gtgtaatgtc aaactagcag aaagccaccg tgaaaaataa actagcaaat
gTvPir_r2A  taacaccctg gtgtaatgtc aaactagcag aaagccaccg tgaaaaataa actagcaaat
gTvPir_r3A  taacaccctg gtgtaatgtc aaactagcag aaagccaccg tgaaaaataa actagcaaat
gTvPir_r5B  taacaccctg gtgtaatgtc aaactagcag aaagccaccg tgaaaaataa actagcaaat
gTvPir_R1B  taacaccctg gtgtaatgtc aaactagcag aaagccaccg tgaaaaataa actagcaaat
gTvPir_r7A  taacaccctg gtgtaatgtc aaactagcag aaagccaccg tgaaaaataa actagcaaat
gTvPir_r1B  taacaccctg gtgtaatgtc aaactagcag aaagccaccg tgaaaaataa actagcaaat
gTvPir_R6B  taacaccctg gtgtaatgtc aaactagcag aaagccaccg tgaaaaataa actagcaaat
gTvPir_R4B  taacaccctg gtgtaatgtc aaactagcag aaagccaccg tgaaaaataa actagcaaat
gTvPir_R6A  taacaccctg gtgtaatgtc aaactagcag aaagccaccg tgaaaaataa actagcaaat
gTvPir_N2A  taacaccctg gtgtaatgtc aaactagcag aaagccaccg tgaaaaataa actagcaaat
gTvPir_N2B  taacaccctg gtgtaatgtc aaactagcag aaagccaccg tgaaaaataa actagcaaat
gTvPir_R2A  taacaccctg gtgtaatgtc aaactagcag aaagccaccg tgaaaaataa actagcaaat
gTvPir_N1B  taacaccctg gtgtaatgtc aaactagcag aaagccaccg tgaaaaataa actagcaaat
gTvPir_R4A  taacaccctg gtgtaatgtc aaactagcag aaagccaccg tgaaaaataa actagcaaat
gTvPir_n3A  taacaccctg gtgtaatgtc aaactagcag aaagccaccg tgaaaaataa actagcaaat
gTvPir_N1A  taacaccctg gtgtaatgtc aaactagcag aaagccaccg tgaaaaataa actagcaaat
gTvPir_n3B  taacaccctg gtgtaatgtc aaactagcag aaagccaccg tgaaaaataa actagcaaat
gTvPir_N3B  taacaccctg gtgtaatgtc aaactagcag aaagccaccg tgaaaaataa actagcaaat
gTvPir_r4B  taacaccctg gtgtaatgtc aaactagcag aaagccaccg tgaaaaataa actagcaaat
gTvPir_r6A  taacaccctg gtgtaatgtc aaactagcag aaagccaccg tgaaaaataa actagcaaat
gTvPir_R3A  taacaccctg gtgtaatgtc aaactagcag aaagccaccg tgaaaaataa actagcaaat
gTvPir_N3A  taacaccctg gtgtaatgtc aaactagcag aaagccaccg tgaaaaataa actagcaaat
gTvPir_r6B  taacaccctg gtgtaatgtc aaactagcag aaagccaccg tgaaaaataa actagcaaat
gTvPir_R5A  taacaccctg gtgtaatgtc aaactagcag aaagccaccg tgaaaaataa actagcaaat
gTvPir_r7B  taacaccctg gtgtaatgtc aaactagcag aaagccaccg tgaaaaataa actagcaaat
gTvPir_R2B  taacaccctg gtgtaatgtc aaactagcag aaagccaccg tgaaaaataa actagcaaat
gTvPir_R3B  taacaccctg gtgtaatgtc aaactagcag aaagccaccg tgaaaaataa actagcaaat
gTvPir_r4A  taacaccctg gtgtaatgtc aaactagcag aaagccaccg tgaaaaataa actagcaaat
gTvPir_R5B  taacaccctg gtgtaatgtc aaactagcag aaagccaccg tgaaaaataa actagcaaat
gTvPir_n2B  taacaccctg gtgtaatgtc aaactagcag aaagccaccg tgaaaaataa actagcaaat
```

```
            421                                                          480
gTvPir_r1A  atccctgtgt aatttaaaat ttgcaaaact tctccaataa aaaataaatt agcctcctta
gTvPir_r2B  atccctgtgt aatttaaaat ttgcaaaact tctccaataa aaaataaatt agcctcctta
gTvPir_r3B  atccctgtgt aatttaaaat ttgcaaaact tctccaataa aaaataaatt agcctcctta
gTvPir_r5A  atccctgtgt aatttaaaat ttgcaaaact tctccaataa aaaataaatt agcctcctta
gTvPir_r8A  atccctgtgt aatttaaaat ttgcaaaact tctccaataa aaaataaatt agcctcctta
gTvPir_r8B  atccctgtgt aatttaaaat ttgcaaaact tctccaataa aaaataaatt agcctcctta
gTvPir_R1A  atccctgtgt aatttaaaat ttgcaaaact tctccaataa aaaataaatt agcctcctta
gTvPir_n1A  atccctgtgt aatttaaaat ttgcaaaact tctccaataa aaaataaatt agcctcctta
gTvPir_n2A  atccctgtgt aatttaaaat ttgcaaaact tctccaataa aaaataaatt agcctcctta
gTvPir_n1B  atccctgtgt aatttaaaat ttgcaaaact tctccaataa aaaataaatt agcctcctta
gTvPir_r2A  atccctgtgt aatttaaaat ttgcaaaact tctccaataa aaaataaatt agcctcctta
gTvPir_r3A  atccctgtgt aatttaaaat ttgcaaaact tctccaataa aaaataaatt agcctcctta
gTvPir_r5B  atccctgtgt aatttaaaat ttgcaaaact tctccaataa aaaataaatt agcctcctta
gTvPir_R1B  atccctgtgt aatttaaaat ttgcaaaact tctccaataa aaaataaatt agcctcctta
gTvPir_r7A  atccctgtgt aatttaaaat ttgcaaaact tctccaataa aaaataaatt agcctcctta
gTvPir_r1B  atccctgtgt aatttaaaat ttgcaaaact tctccaataa aaaataaatt agcctcctta
gTvPir_R6B  atccctgtgt aatttaaaat ttgcaaaact tctccaataa aaaataaatt agcctcctta
gTvPir_R4B  atccctgtgt aatttaaaat ttgcaaaact tctccaataa aaaataaatt agcctcctta
gTvPir_R6A  atccctgtgt aatttaaaat ttgcaaaact tctccaataa aaaataaatt agcctcctta
gTvPir_N2A  atccctgtgt aatttaaaat ttgcaaaact tctccaataa aaaataaatt agcctcctta
gTvPir_N2B  atccctgtgt aatttaaaat ttgcaaaact tctccaataa aaaataaatt agcctcctta
gTvPir_R2A  atccatgtgt aatttaaaat ttgcaaaact tctccaataa aaaataaatt agcctcctta
gTvPir_N1B  atccatgtgt aatttaaaat ttgcaaaact tctccaataa aaaataaatt agcctcctta
gTvPir_R4A  atccctgtgt aatttaaaat ttgcaaaact tctccaataa aaaataaatt agcctcctta
gTvPir_n3A  atccctgtgt aatttaaaat ttgcaaaact tctccaataa aaaataaatt agcctcctta
gTvPir_N1A  atccctgtgt aatttaaaat ttgcaaaact tctccaataa aaaataaatt agcctcctta
gTvPir_n3B  atccctgtgt aatttaaaat ttgcaaaact tctccaataa aaaataaatt agcctcctta
gTvPir_N3B  atccctgtgt aatttaaaat ttgcaaaact tctccaataa aaaataaatt agcctcctta
gTvPir_r4B  atccatgtgt aatttaaaat ttgcaaaact tctccaataa aaaataaatt agcctcctta
gTvPir_r6A  atccatgtgt aatttaaaat ttgcaaaact tctccaataa aaaataaatt agcctcctta
gTvPir_R3A  atccatgtgt aatttaaaat ttgcaaaact tctccaataa aaaataaatt agcctcctta
gTvPir_N3A  atccatgtgt aatttaaaat ttgcaaaact tctccaataa aaaataaatt agcctcctta
gTvPir_r6B  atccatgtgt aatttaaaat ttgcaaaact tctccaataa aaaataaatt agcctcctta
gTvPir_R5A  atccatgtgt aatttaaaat ttgcaaaact tctccaataa aaaataaatt agcctcctta
gTvPir_r7B  atccatgtgt aatttaaaat ttgcaaaact tctccaataa aaaataaatt agcctcctta
gTvPir_R2B  atccatgtgt aatttaaaat ttgcaaaact tctccaataa aaaataaatt agcctcctta
gTvPir_R3B  atccctgtgt aatttaaaat ttgcaaaact tctccaataa aaaataaatt agcctcctta
gTvPir_r4A  atccatgtgt aatttaaaat ttgcaaaact tctccaataa aaaataaatt agcctcctta
gTvPir_R5B  atccatgtgt aatttaaaat ttgcaaaact tctccaataa aaaataaatt agcctcctta
gTvPir_n2B  atccatgtgt aatttaaaat ttgcaaaact tctccaataa aaaataaatt agcctcctta
```

```
            481                                                      540
gTvPir_r1A  aaaattattt atccttataa ataggtgtgg gtctgtgcca ttaaataatt tttatagggg
gTvPir_r2B  aaaattattt atccttataa ataggtgtgg gtctgtgcca ttaaataatt tttatagggg
gTvPir_r3B  aaaattattt atccttataa ataggtgtgg gtctgtgcca ttaaataatt tttatagggg
gTvPir_r5A  aaaattattt atccttataa ataggtgtgg gtctgtgcca ttaaataatt tttatagggg
gTvPir_r8A  aaaattattt atccttataa ataggtgtgg gtctgtgcca ttaaataatt tttatagggg
gTvPir_r8B  aaaattattt atccttataa ataggtgtgg gtctgtgcca ttaaataatt tttatagggg
gTvPir_R1A  aaaattattt atccttataa ataggtgtgg gtctgtgcca ttaaataatt tttatagggg
gTvPir_n1A  aaaattattt atccttataa ataggtgtgg gtctgtgcca ttaaataatt tttatagggg
gTvPir_n2A  aaaattattt atccttataa ataggtgtgg gtctgtgcca ttaaataatt tttatagggg
gTvPir_n1B  aaaattattt atccttataa ataggtgtgg gtctgtgcca ttaaataatt tttatagggg
gTvPir_r2A  aaaattattt atccttataa ataggtgtgg gtctgtgcca ttaaataatt tttatagggg
gTvPir_r3A  aaaattattt atccttataa ataggtgtgg gtctgtgcca ttaaataatt tttatagggg
gTvPir_r5B  aaaattattt atccttataa ataggtgtgg gtctgtgcca ttaaataatt tttatagggg
gTvPir_R1B  aaaattattt atccttataa ataggtgtgg gtctgtgcca ttaaataatt tttatagggg
gTvPir_r7A  aaaattattt atccttataa ataggtgtgg gtctgtgcca ttaaataatt tttatagggg
gTvPir_r1B  aaaattattt atccttataa ataggtgtgg gtctgtgcca ttaaataatt tttatagggg
gTvPir_R6B  aaaattattt atccttataa ataggtgtgg gtctgtgcca ttaaataatt tttatagggg
gTvPir_R4B  aaaattattt atccttataa ataggtgtgg gtctgtgcca ttaaataatt tttatagggg
gTvPir_R6A  aaaattattt atccttataa ataggtgtgg gtctgtgcca ttaaataatt tttatagggg
gTvPir_N2A  aaaattattt atccttataa ataggtgtgg gtctgtgcca ttaaataatt tttatagggg
gTvPir_N2B  aaaattattt atccttataa ataggtgtgg gtctgtgcca ttaaataatt tttatagggg
gTvPir_R2A  aaaattattt atccttataa ataggtgtgg gtctgtgcca ttaaataatt tttatagggg
gTvPir_N1B  aaaattattt atccttataa ataggtgtgg gtctgtgcca ttaaataatt tttatagggg
gTvPir_R4A  aaaattattt atccttataa ataggtgtgg gtctgtgcca ttaaataatt tttatagggg
gTvPir_n3A  aaaattattt atccttataa ataggtgtgg gtctgtgcca ttaaataatt tttatagggg
gTvPir_N1A  aaaattattt atccttataa ataggtgtgg gtctgtgcca ttaaataatt tttatagggg
gTvPir_n3B  aaaattattt atccttataa ataggtgtgg gtctgtgcca ttaaataatt tttatagggg
gTvPir_N3B  aaaattattt atccttataa ataggtgtgg gtctgtgcca ttaaataatt tttatagggg
gTvPir_r4B  aaaattattt atccttataa ataggtgtgg gtctgtgcca ttaaataatt tttatagggg
gTvPir_r6A  aaaattattt atccttataa ataggtgtgg gtctgtgcca ttaaataatt tttatagggg
gTvPir_R3A  aaaattattt atccttataa ataggtgtgg gtctgtgcca ttaaataatt tttatagggg
gTvPir_N3A  aaaattattt atccttataa ataggtgtgg gtctgtgcca ttaaataatt tttatagggg
gTvPir_r6B  aaaattattt atccttataa ataggtgtgg gtctgtgcca ttaaataatt tttatagggg
gTvPir_R5A  aaaattattt atccttataa ataggtgtgg gtctgtgcca ttaaataatt tttatagggg
gTvPir_r7B  aaaattattt atccttataa ataggtgtgg gtctgtgcca ttaaataatt tttatagggg
gTvPir_R2B  aaaattattt atccttataa ataggtgtgg gtctgtgcca ttaaataatt tttatagggg
gTvPir_R3B  aaaattattt atccttataa ataggtgtgg gtctgtgcca ttaaataatt tttatagggg
gTvPir_r4A  aaaattattt atccttataa ataggtgtgg gtctgtgcca ttaaataatt tttatagggg
gTvPir_R5B  aaaattattt atccttataa ataggtgtgg gtctgtgcca ttaaataatt tttatagggg
gTvPir_n2B  aaaattattt atccttataa ataggtgtgg gtctgtgcca ttaaataatt tttatagggg
```

```
             541                                                        600
gTvPir_r1A  ttaaatgcta ttaactaaat ttcatagggg ttatgctagt tacaaagtta ccgggttatt
gTvPir_r2B  ttaaatgcta ttaactaaat ttcatagggg ttatgctagt tacaaagtta ccgggttatt
gTvPir_r3B  ttaaatgcta ttaactaaat ttcatagggg ttatgctagt tacaaagtta ccgggttatt
gTvPir_r5A  ttaaatgcta ttaactaaat ttcatagggg ttatgctagt tacaaagtta ccgggttatt
gTvPir_r8A  ttaaatgcta ttaactaaat ttcatagggg ttatgctagt tacaaagtta ccgggttatt
gTvPir_r8B  ttaaatgcta ttaactaaat ttcatagggg ttatgctagt tacaaagtta ccgggttatt
gTvPir_R1A  ttaaatgcta ttaactaaat ttcatagggg ttatgctagt tacaaagtta ccgggttatt
gTvPir_n1A  ttaaatgcta ttaactaaat ttcatagggg ttatgctagt tacaaagtta ccgggttatt
gTvPir_n2A  ttaaatgcta ttaactaaat ttcatagggg ttatgctagt tacaaagtta ccgggttatt
gTvPir_n1B  ttaaatgcta ttaactaaat ttcatagggg ttatgctagt tacaaagtta ccgggttatt
gTvPir_r2A  ttaaatgcta ttaactaaat ttcatagggg ttatgctagt tacaaagtta ccgggttatt
gTvPir_r3A  ttaaatgcta ttaactaaat ttcatagggg ttatgctagt tacaaagtta ccgggttatt
gTvPir_r5B  ttaaatgcta ttaactaaat ttcatagggg ttatgctagt tacaaagtta ccgggttatt
gTvPir_R1B  ttaaatgcta ttaactaaat ttcatagggg ttatgctagt tacaaagtta ccgggttatt
gTvPir_r7A  ttaaatgcta ttaactaaat ttcatagggg ttatgctagt tacaaagtta ccgggttatt
gTvPir_r1B  ttaaatgcta ttaactaaat ttcatagggg ttatgctagt tacaaagtta ccgggttatt
gTvPir_R6B  ttaaatgcta ttaactaaat ttcatagggg ttatgctagt tacaaagtta ccgggttatt
gTvPir_R4B  ttaaatgcta ttaactaaat ttcatagggg ttatgctagt tacaaagtta ccgggttatt
gTvPir_R6A  ttaaatgcta ttaactaaat ttcatagggg ttatgctagt tacaaagtta ccgggttatt
gTvPir_N2A  ttaaatgcta ttaactaaat ttcatagggg ttatgctagt tacaaagtta ccgggttatt
gTvPir_N2B  ttaaatgcta ttaactaaat ttcatagggg ttatgctagt tacaaagtta ccgggttatt
gTvPir_R2A  ttaaatgcta ttaactaaat ttcatagggg ttatgctagt tacaaagtta ccgggttatt
gTvPir_N1B  ttaaatgcta ttaactaaat ttcatagggg ttatgctagt tacaaagtta ccgggttatt
gTvPir_R4A  ttaaatgcta ttaactaaat ttcatagggg ttatgctagt tacaaagtta ccgggttatt
gTvPir_n3A  ttaaatgcta ttaactaaat ttcatagggg ttatgctagt tacaaagtta ccgggttatt
gTvPir_N1A  ttaaatgcta ttaactaaat ttcatagggg ttatgctagt tacaaagtta ccgggttatt
gTvPir_n3B  ttaaatgcta ttaactaaat ttcatagggg ttatgctagt tacaaagtta ccgggttatt
gTvPir_N3B  ttaaatgcta ttaactaaat ttcatagggg ttatgctagt tacaaagtta ccgggttatt
gTvPir_r4B  ttaaatgcta ttaactaaat ttcatagggg ttatgctagt tacaaagtta ccgggttatt
gTvPir_r6A  ttaaatgcta ttaactaaat ttcatagggg ttatgctagt tacaaagtta ccgggttatt
gTvPir_R3A  ttaaatgcta ttaactaaat ttcatagggg ttatgctagt tacaaagtta ccgggttatt
gTvPir_N3A  ttaaatgcta ttaactaaat ttcatagggg ttatgctagt tacaaagtta ccgggttatt
gTvPir_r6B  ttaaatgcta ttaactaaat ttcatagggg ttatgctagt tacaaagtta ccgggttatt
gTvPir_R5A  ttaaatgcta ttaactaaat ttcatagggg ttatgctagt tacaaagtta ccgggttatt
gTvPir_r7B  ttaaatgcta ttaactaaat ttcatagggg ttatgctagt tacaaagtta ccgggttatt
gTvPir_R2B  ttaaatgcta ttaactaaat ttcatagggg ttatgctagt tacaaagtta ccgggttatt
gTvPir_R3B  ttaaatgcta ttaactaaat ttcatagggg ttatgctagt tacaaagtta ccgggttatt
gTvPir_r4A  ttaaatgcta ttaactaaat ttcatagggg ttatgctagt tacaaagtta ccgggttatt
gTvPir_R5B  ttaaatgcta ttaactaaat ttcatagggg ttatgctagt tacaaagtta ccgggttatt
gTvPir_n2B  ttaaatgcta ttaactaaat ttcatagggg ttatgctagt tacaaagtta ccgggttatt
```

```
             601                                                     660
gTvPir_r1A  tgctagttta attttcacat ggtaatttgt gctaattttg aaaaaacaga ggggtgtata
gTvPir_r2B  tgctagttta attttcacat ggtaatttgt gctaattttg aaaaaacaga ggggtgtata
gTvPir_r3B  tgctagttta attttcacat ggtaatttgt gctaattttg aaaaaacaga ggggtgtata
gTvPir_r5A  tgctagttta attttcacat ggtaatttgt gctaattttg aaaaaacaga ggggtgtata
gTvPir_r8A  tgctagttta attttcacat ggtaatttgt gctaattttg aaaaaacaga ggggtgtata
gTvPir_r8B  tgctagttta attttcacat ggtaatttgt gctaattttg aaaaaacaga ggggtgtata
gTvPir_R1A  tgctagttta attttcacat ggtaatttgt gctaattttg aaaaaacaga ggggtgtata
gTvPir_n1A  tgctagttta attttcacat ggtaatttgt gctaattttg aaaaaacaga ggggtgtata
gTvPir_n2A  tgctagttta attttcacat ggtaatttgt gctaattttg aaaaaacaga ggggtgtata
gTvPir_n1B  tgctagttta attttcacat ggtaatttgt gctaattttg aaaaaacaga ggggtgtata
gTvPir_r2A  tgctagttta attttcacat ggtaatttgt gctaattttg aaaaaacgga ggggtgtata
gTvPir_r3A  tgctagttta attttcacat ggtaatttgt gctaattttg aaaaaacgga ggggtgtata
gTvPir_r5B  tgctagttta attttcacat ggtaatttgt gctaattttg aaaaaacgga ggggtgtata
gTvPir_R1B  tgctagttta attttcacat ggtaatttgt gctaattttg aaaaaacgga ggggtgtata
gTvPir_r7A  tgctagttta attttcacat ggtaatttgt gctaattttg aaaaaacaga ggggtgtata
gTvPir_r1B  tgctagttta attttcacat ggtaatttgt gctaattttg aaaaaacaga ggggtgtata
gTvPir_R6B  tgctagttta attttcacat ggtaatttgt gctaattttg aaaaaacaga ggggtgtata
gTvPir_R4B  tgctagttta attttcacat ggtaatttgt gctaattttg aaaaaacaga ggggtgtata
gTvPir_R6A  tgctagttta attttcacat ggtaatttgt gctaattttg aaaaaacaga ggggtgtata
gTvPir_N2A  tgctagttta attttcacat ggtaatttgt gctaattttg aaaaaacaga ggggtgtata
gTvPir_N2B  tgctagttta attttcacat ggtaatttgt gctaattttg aaaaaacaga ggggtgtata
gTvPir_R2A  tgctagttta attttcacat ggtaatttgt gctaattttg aaaaaacaga ggggtgtata
gTvPir_N1B  tgctagttta attttcacat ggtaatttgt gctaattttg aaaaaacaga ggggtgtata
gTvPir_R4A  tgctagttta attttcacat ggtaatttgt gctaattttg aaaaaacaga ggggtgtata
gTvPir_n3A  tgctagttta attttcacat ggtaatttgt gctaattttg aaaaaacaga ggggtgtata
gTvPir_N1A  tgctagttta attttcacat ggtaatttgt gctaattttg aaaaaacaga ggggtgtata
gTvPir_n3B  tgctagttta attttcacat ggtaatttgt gctaattttg aaaaaacaga ggggtgtata
gTvPir_N3B  tgctagttta attttcacat ggtaatttgt gctaattttg aaaaaacaga ggggtgtata
gTvPir_r4B  tgctagttta attttcacat ggtaatttgt gctaattttg aaaaaacaga ggggtgtata
gTvPir_r6A  tgctagttta attttcacat ggtaatttgt gctaattttg aaaaaacaga ggggtgtata
gTvPir_R3A  tgctagttta attttcacat ggtaatttgt gctaattttg aaaaaacaga ggggtgtata
gTvPir_N3A  tgctagttta attttcacat ggtaatttgt gctaattttg aaaaaacaga ggggtgtata
gTvPir_r6B  tgctagttta attttcacat ggtaatttgt gctaattttg aaaaaacaga ggggtgtata
gTvPir_R5A  tgctagttta attttcacat ggtaatttgt gctaattttg aaaaaacaga ggggtgtata
gTvPir_r7B  tgctagttta attttcacat ggtaatttgt gctaattttg aaaaaacaga ggggtgtata
gTvPir_R2B  tgctagttta attttcacat ggtaatttgt gctaattttg aaaaaacaga ggggtgtata
gTvPir_R3B  tgctagttta attttcacat ggtaatttgt gctaattttg aaaaaaccga ggggtgtata
gTvPir_r4A  tgctagttta attttcacat ggtaatttgt gctaattttg aaaaaacaga ggggtgtata
gTvPir_R5B  tgctagttta attttcacat ggtaatttgt gctaattttg aaaaaacaga ggggtgtata
gTvPir_n2B  tgctagttta attttcacat ggtaatttgt gctaattttg aaaaaacaga ggggtgtata
```

```
            661                                                        720
gTvPir_r1A  tgcaactagc tcagtaaaaa attaaaaaat tgggttttat ctaaaattca gatgataatt
gTvPir_r2B  tgcaactagc tcagtaaaaa attaaaaaat tgggttttat ctaaaattca gatgataatt
gTvPir_r3B  tgcaactagc tcagtaaaaa attaaaaaat tgggttttat ctaaaattca gatgataatt
gTvPir_r5A  tgcaactagc tcagtaaaaa attaaaaaat tgggttttat ctaaaattca gatgataatt
gTvPir_r8A  tgcaactagc tcagtaaaaa attaaaaaat tgggttttat ctaaaattca gatgataatt
gTvPir_r8B  tgcaactagc tcagtaaaaa attaaaaaat tgggttttat ctaaaattca gatgataatt
gTvPir_R1A  tgcaactagc tcagtaaaaa attaaaaaat tgggttttat ctaaaattca gatgataatt
gTvPir_n1A  tgcaactagc tcagtaaaaa attaaaaaat tgggttttat ctaaaattca gatgataatt
gTvPir_n2A  tgcaactagc tcagtaaaaa attaaaaaat tgggttttat ctaaaattca gatgataatt
gTvPir_n1B  tgcaactagc tcagtaaaaa attaaaaaat tgggttttat ctaaaattca gatgataatt
gTvPir_r2A  tgcaactagc tcagtaaaaa attaaaaaat tgggttttat ctaaaattca gatgataatt
gTvPir_r3A  tgcaactagc tcagtaaaaa attaaaaaat tgggttttat ctaaaattca gatgataatt
gTvPir_r5B  tgcaactagc tcagtaaaaa attaaaaaat tgggttttat ctaaaattca gatgataatt
gTvPir_R1B  tgcaactagc tcagtaaaaa attaaaaaat tgggttttat ctaaaattca gatgataatt
gTvPir_r7A  tgcaactagc tcagtaaaaa attaaaaaat tgggttttat ctaaaattca gatgataatt
gTvPir_r1B  tgcaactagc tcagtaaaaa attaaaaaat tgggttttat ctaaaattca gatgataatt
gTvPir_R6B  tgcaactagc tcagtaaaaa attaaaaaat tgggttttat ctaaaattca gatgataatt
gTvPir_R4B  tgcaactagc tcagtaaaaa attaaaaaat tgggttttat ctaaaattca gatgataatt
gTvPir_R6A  tgcaactagc tcagtaaaaa attaaaaaat tgggttttat ctaaaattca gatgataatt
gTvPir_N2A  tgcaactagc tcagtaaaaa attaaaaaat tgggttttat ctaaaattca gatgataatt
gTvPir_N2B  tgcaactagc tcagtaaaaa attaaaaaat tgggttttat ctaaaattca gatgataatt
gTvPir_R2A  tgcaactagc tcagtaaaaa attaaaaaat tgggttttat ctaaaattca gatgataatt
gTvPir_N1B  tgcaactagc tcagtaaaaa attaaaaaat tgggttttat ctaaaattca gatgataatt
gTvPir_R4A  tgcaactagc tcagtaaaaa attaaaaaat tgggttttat ctaaaattca gatgataatt
gTvPir_n3A  tgcaactagc tcagtaaaaa attaaaaaat tgggttttat ctaaaattca gatgataatt
gTvPir_N1A  tgcaactagc tcagtaaaaa attaaaaaat tgggttttat ctaaaattca gatgataatt
gTvPir_n3B  tgcaactagc tcagtaaaaa attaaaaaat tgggttttat ctaaaattca gatgataatt
gTvPir_N3B  tgcaactagc tcagtaaaaa attaaaaaat tgggttttat ctaaaattca gatgataatt
gTvPir_r4B  tgcaactagc tcagtaaaaa attaaaaaat tgggttttat ctaaaattca gatgataatt
gTvPir_r6A  tgcaactagc tcagtaaaaa attaaaaaat tgggttttat ctaaaattca gatgataatt
gTvPir_R3A  tgcaactagc tcagtaaaaa attaaaaaat tgggttttat ctaaaattca gatgataatt
gTvPir_N3A  tgcaactagc tcagtaaaaa attaaaaaat tgggttttat ctaaaattca gatgataatt
gTvPir_r6B  tgcaactagc tcagtaaaaa attaaaaaat tgggttttat ctaaaattca gatgataatt
gTvPir_R5A  tgcaactagc tcagtaaaaa attaaaaaat tgggttttat ctaaaattca gatgataatt
gTvPir_r7B  tgcaactagc tcagtaaaaa attaaaaaat tgggttttat ctaaaattca gatgataatt
gTvPir_R2B  tgcaactagc tcagtaaaaa attaaaaaat tgggttttat ctaaaattca gatgataatt
gTvPir_R3B  tgcaactagc tcagtaaaaa attaaaaaat tgggttttat ctaaaattca gatgataatt
gTvPir_r4A  tgcaactagc tcagtaaaaa attaaaaaat tgggttttat ctaaaattca gatgataatt
gTvPir_R5B  tgcaactagc tcagtaaaaa attaaaaaat tgggttttat ctaaaattca gatgataatt
gTvPir_n2B  tgcaactagc tcagtaaaaa attaaaaaat tgggttttat ctaaaattca gatgataatt
```

```
            721                                                        780
gTvPir_r1A  tatttgtatt tttaccaatt cctatattac agTTACACCT CCTGCTGGAT TTCCTGACCA
gTvPir_r2B  tatttgtatt tttaccaatt cctatattac agTTACACCT CCTGCTGGAT TTCCTGACCA
gTvPir_r3B  tatttgtatt tttaccaatt cctatattac agTTACACCT CCTGCTGGAT TTCCTGACCA
gTvPir_r5A  tatttgtatt tttaccaatt cctatattac agTTACACCT CCTGCTGGAT TTCCTGACCA
gTvPir_r8A  tatttgtatt tttaccaatt cctatattac agTTACACCT CCTGCTGGAT TTCCTGACCA
gTvPir_r8B  tatttgtatt tttaccaatt cctatattac agTTACACCT CCTGCTGGAT TTCCTGACCA
gTvPir_R1A  tatttgtatt tttaccaatt cctatattac agTTACACCT CCTGCTGGAT TTCCTGACCA
gTvPir_n1A  tatttgtatt tttaccaatt cctatattac agTTACACCT CCTGCTGGAT TTCCTGACCA
gTvPir_n2A  tatttgtatt tttaccaatt cctatattac agTTACACCT CCTGCTGGAT TTCCTGACCA
gTvPir_n1B  tatttgtatt tttaccaatt cctatattac agTTACACCT CCTGCTGGAT TTCCTGACCA
gTvPir_r2A  tatttgtatt tttaccaatt cctatattac agTTACACCT CCTGCTGGAT TTCCTGACCA
gTvPir_r3A  tatttgtatt tttaccaatt cctatattac agTTACACCT CCTGCTGGAT TTCCTGACCA
gTvPir_r5B  tatttgtatt tttaccaatt cctatattac agTTACACCT CCTGCTGGAT TTCCTGACCA
gTvPir_R1B  tatttgtatt tttaccaatt cctatattac agTTACACCT CCTGCTGGAT TTCCTGACCA
gTvPir_r7A  tatttgtatt tttaccaatt cctatattac agTTACACCT CCTGCTGGAT TTCCTGACCA
gTvPir_r1B  tatttgtatt tttaccaatt cctatattac agTTACACCT CCTGCTGGAT TTCCTGACCA
gTvPir_R6B  tatttgtatt tttaccaatt cctatattac agTTACACCT CCTGCTGGAT TTCCTGACCA
gTvPir_R4B  tatttgtatt tttaccaatt cctatattac agTTACACCT CCTGCTGGAT TTCCTGACCA
gTvPir_R6A  tatttgtatt tttaccaatt cctatattac agTTACACCT CCTGCTGGAT TTCCTGACCA
gTvPir_N2A  tatttgtatt tttaccaatt cctatattac agTTACACCT CCTGCTGGAT TTCCTGACCA
gTvPir_N2B  tatttgtatt tttaccaatt cctatattac agTTACACCT CCTGCTGGAT TTCCTGACCA
gTvPir_R2A  tatttgtatt tttaccaatt cctatattac agTTACACCT CCTGCTGGAT TTCCTGACCA
gTvPir_N1B  tatttgtatt tttaccaatt cctatattac agTTACACCT CCTGCTGGAT TTCCTGACCA
gTvPir_R4A  tatttgtatt tttaccaatt cctatattac agTTACACCT CCTGCTGGAT TTCCTGACCA
gTvPir_n3A  tatttgtatt tttaccaatt cctatattac agTTACACCT CCTGCTGGAT TTCCTGACCA
gTvPir_N1A  tatttgtatt tttaccaatt cctatattac agTTACACCT CCTGCTGGAT TTCCTGACCA
gTvPir_n3B  tatttgtatt tttaccaatt cctatattac agTTACACCT CCTGCTGGAT TTCCTGACCA
gTvPir_N3B  tatttgtatt tttaccaatt cctatattac agTTACACCT CCTGCTGGAT TTCCTGACCA
gTvPir_r4B  tatttgtatt tttaccaatt cctatattac agTTACACCT CCTGCTGGAT TTCCTGACCA
gTvPir_r6A  tatttgtatt tttaccaatt cctatattac agTTACACCT CCTGCTGGAT TTCCTGACCA
gTvPir_R3A  tatttgtatt tttaccaatt cctatattac agTTACACCT CCTGCTGGAT TTCCTGACCA
gTvPir_N3A  tatttgtatt tttaccaatt cctatattac agTTACACCT CCTGCTGGAT TTCCTGACCA
gTvPir_r6B  tatttgtatt tttaccaatt cctatattac agTTACACCT CCTGCTGGAT TTCCTGACCA
gTvPir_R5A  tatttgtatt tttaccaatt cctatattac agTTACACCT CCTGCTGGAT TTCCTGACCA
gTvPir_r7B  tatttgtatt tttaccaatt cctatattac agTTACACCT CCTGCTGGAT TTCCTGACCA
gTvPir_R2B  tatttgtatt tttaccaatt cctatattac agTTACACCT CCTGCTGGAT TTCCTGACCA
gTvPir_R3B  tatttgtatt tttaccaatt cctatattac agTTACACCT CCTGCTGGAT TTCCTGACCA
gTvPir_r4A  tatttgtatt tttaccaatt cctatattac agTTACACCT CCTGCTGGAT TTCCTGACCA
gTvPir_R5B  tatttgtatt tttaccaatt cctatattac agTTACACCT CCTGCTGGAT TTCCTGACCA
gTvPir_n2B  tatttgtatt tttaccaatt cctatattac agTTACACCT CCTGCTGGAT TTCCTGACCA
```

```
             781                                                         840
gTvPir_r1A  TCCTCACCGA Ggtgctttgt cctctttgtt tgatattcat ttttaagttg cttaacgttt
gTvPir_r2B  TCCTCACCGA Ggtgctttgt cctctttgtt tgatattcat ttttaagttg cttaacgttt
gTvPir_r3B  TCCTCACCGA Ggtgctttgt cctctttgtt tgatattcat ttttaagttg cttaacgttt
gTvPir_r5A  TCCTCACCGA Ggtgctttgt cctctttgtt tgatattcat ttttaagttg cttaacgttt
gTvPir_r8A  TCCTCACCGA Ggtgctttgt cctctttgtt tgatattcat ttttaagttg cttaacgttt
gTvPir_r8B  TCCTCACCGA Ggtgctttgt cctctttgtt tgatattcat ttttaagttg cttaacgttt
gTvPir_R1A  TCCTCACCGA Ggtgctttgt cctctttgtt tgatattcat ttttaagttg cttaacgttt
gTvPir_n1A  TCCTCACCGA Ggtgctttgt cctctttgtt tgatattcat ttttaagttg cttaacgttt
gTvPir_n2A  TCCTCACCGA Ggtgctttgt cctctttgtt tgatattcat ttttaagttg cttaacgttt
gTvPir_n1B  TCCTCACCGA Ggtgctttgt cctctttgtt tgatattcat ttttaagttg cttaacgttt
gTvPir_r2A  TCCTCACCGA Ggtgctttgt cctctttgtt tgatattcat ttttaagttg cttaacgttt
gTvPir_r3A  TCCTCACCGA Ggtgctttgt cctctttgtt tgatattcat ttttaagttg cttaacgttt
gTvPir_r5B  TCCTCACCGA Ggtgctttgt cctctttgtt tgatattcat ttttaagttg cttaacgttt
gTvPir_R1B  TCCTCACCGA Ggtgctttgt cctctttgtt tgatattcat ttttaagttg cttaacgttt
gTvPir_r7A  TCCTCACCGA Ggtgctttgt cctctttgtt tgatattcat ttttaagttg cttaacgttt
gTvPir_r1B  TCCTCACCGA Ggtgctttgt cctctttgtt tgatattcat ttttaagttg cttaacgttt
gTvPir_R6B  TCCTCACCGA Ggtgctttgt cctctttgtt tgatattcat ttttaagttg cttaacgttt
gTvPir_R4B  TCCTCACCGA Ggtgctttgt cctctttgtt tgatattcat ttttaagttg cttaacgttt
gTvPir_R6A  TCCTCACCGA Ggtgctttgt cctctttgtt tgatattcat ttttaagttg cttaacgttt
gTvPir_N2A  TCCTCACCGA Ggtgctttgt cctctttgtt tgatattcat ttttaagttg cttaacgttt
gTvPir_N2B  TCCTCACCGA Ggtgctttgt cctctttgtt tgatattcat ttttaagttg cttaacgttt
gTvPir_R2A  TCCTCACCGA Ggtgctttgt cctctttgtt tgatattcat ttttaagttg cttaacgttt
gTvPir_N1B  TCCTCACCGA Ggtgctttgt cctctttgtt tgatattcat ttttaagttg cttaacgttt
gTvPir_R4A  TCCTCACCGA Ggtgctttgt cctctttgtt tgatattcat ttttaagttg cttaacgttt
gTvPir_n3A  TCCTCACCGA Ggtgctttgt cctctttgtt tgatattcat ttttaagttg cttaacgttt
gTvPir_N1A  TCCTCACCGA Ggtgctttgt cctctttgtt tgatattcat ttttaagttg cttaacgttt
gTvPir_n3B  TCCTCACCGA Ggtgctttgt cctctttgtt tgatattcat ttttaagttg cttaacgttt
gTvPir_N3B  TCCTCACCGA Ggtgctttgt cctctttgtt tgatattcat ttttaagttg cttaacgttt
gTvPir_r4B  TCCTCACCGA Ggtgctttgg cctctttgtt tgatattcat ttttaagttg cttaacgttt
gTvPir_r6A  TCCTCACCGA Ggtgctttgt cctctttgtt tgatattcat ttttaagttg cttaacgttt
gTvPir_R3A  TCCTCACCGA Ggtgctttgt cctctttgtt tgatattcat ttttaagttg cttaacgttt
gTvPir_N3A  TCCTCACCGA Ggtgctttgt cctctttgtt tgatattcat ttttaagttg cttaacgttt
gTvPir_r6B  TCCTCACCGA Ggtgctttgt cctctttgtt tgatattcat ttttaagttg cttaacgttt
gTvPir_R5A  TCCTCACCGA Ggtgctttgt cctctttgtt tgatattcat ttttaagttg cttaacgttt
gTvPir_r7B  TCCTCACCGA Ggtgctttgt cctctttgtt tgatattcat ttttaagttg cttaacgttt
gTvPir_R2B  TCCTCACCGA Ggtgctttgt cctctttgtt tgatattcat ttttaagttg cttaacgttt
gTvPir_R3B  TCCTCACCGA Ggtgctttgt cctctttgtt tgatattcat ttttaagttg cttaacgttt
gTvPir_r4A  TCCTCACCGA Ggtgctttgg cctctttgtt tgatattcat ttttaagttg cttaacgttt
gTvPir_R5B  TCCTCACCGA Ggtgctttgt cctctttgtt tgatattcat ttttaagttg cttaacgttt
gTvPir_n2B  TCCTCACCGA Ggtgctttgt cctctttgtt tgatattcat ttttaagttg cttaacgttt
```

```
             841                                                          900
gTvPir_r1A  atgcaaacag aatattaatg aagtgcttgt tctacagGCT TTGAGACTGT TACATATATG
gTvPir_r2B  atgcaaacag aatattaatg aagtgcttgt tctacagGCT TTGAGACTGT TACATATATG
gTvPir_r3B  atgcaaacag aatattaatg aagtgcttgt tctacagGCT TTGAGACTGT TACATATATG
gTvPir_r5A  atgcaaacag aatattaatg aagtgcttgt tctacagGCT TTGAGACTGT TACATATATG
gTvPir_r8A  atgcaaacag aatattaatg aagtgcttgt tctacagGCT TTGAGACTGT TACATATATG
gTvPir_r8B  atgcaaacag aatattaatg aagtgcttgt tctacagGCT TTGAGACTGT TACATATATG
gTvPir_R1A  atgcaaacag aatattaatg aagtgcttgt tctacagGCT TTGAGACTGT TACATATATG
gTvPir_n1A  atgcaaacag aatattaatg aagtgcttgt tctacagGCT TTGAGACTGT TACATATATG
gTvPir_n2A  atgcaaacag aatattaatg aagtgcttgt tctacagGCT TTGAGACTGT TACATATATG
gTvPir_n1B  atgcaaacag aatattaatg aagtgcttgt tctacagGCT TTGAGACTGT TACATATATG
gTvPir_r2A  atgcaaacag aatattaatg aagtgcttgt tctacagGCT TTGAGACTGT TACATATATG
gTvPir_r3A  atgcaaacag aatattaatg aagtgcttgt tctacagGCT TTGAGACTGT TACATATATG
gTvPir_r5B  atgcaaacag aatattaatg aagtgcttgt tctacagGCT TTGAGACTGT TACATATATG
gTvPir_R1B  atgcaaacag aatattaatg aagtgcttgt tctacagGCT TTGAGACTGT TACATATATG
gTvPir_r7A  atgcaaacag aatattaatg aagtgcttgt tctacagGCT TTGAGACTGT TACATATATG
gTvPir_r1B  atgcaaacag aatattaatg aagtgcttgt tctacagGCT TTGAGACTGT TACATATATG
gTvPir_R6B  atgcaaacag aatattaatg aagtgcttgt tctacagGCT TTGAGACTGT TACATATATG
gTvPir_R4B  atgcaaacag aatattaatg aagtgcttgt tctacagGCT TTGAGACTGT TACATATATG
gTvPir_R6A  atgcaaacag aatattaatg aagtgcttgt tctacagGCT TTGAGACTGT TACATATATG
gTvPir_N2A  atgcaaacag aatattaatg aagtgcttgt tctacagGCT TTGAGACTGT TACATATATG
gTvPir_N2B  atgcaaacag aatattaatg aagtgcttgt tctacagGCT TTGAGACTGT TACATATATG
gTvPir_R2A  atgcaaacag aatattaatg aagtgcttgt tctacagGCT TTGAGACTGT TACATATATG
gTvPir_N1B  atgcaaacag aatattaatg aagtgcttgt tctacagGCT TTGAGACTGT TACATATATG
gTvPir_R4A  atgcaaacag aatattaatg aagtgcttgt tctacagGCT TTGAGACTGT TACATATATG
gTvPir_n3A  atgcaaacag aatattaatg aagtgcttgt tctacagGCT TTGAGACTGT TACATATATG
gTvPir_N1A  atgcaaacag aatattaatg aagtgcttgt tctacagGCT TTGAGACTGT TACATATATG
gTvPir_n3B  atgcaaacag aatattaatg aagtgcttgt tctacagGCT TTGAGACTGT TACATATATG
gTvPir_N3B  atgcaaacag aatattaatg aagtgcttgt tctacagGCT TTGAGACTGT TACATATATG
gTvPir_r4B  atgcaaacag aatattaatg aagtgcttgt tctacagGCT TTGAGACTGT TACATATATG
gTvPir_r6A  atgcaaacag aatattaatg aagtgcttgt tctacagGCT TTGAGACTGT TACATATATG
gTvPir_R3A  atgcaaacag aatattaatg aagtgcttgt tctacagGCT TTGAGACTGT TACATATATG
gTvPir_N3A  atgcaaacag aatattaatg aagtgcttgt tctacagGCT TTGAGACTGT TACATATATG
gTvPir_r6B  atgcaaacag aatattaatg aagtgcttgt tctacagGCT TTGAGACTGT TACATATATG
gTvPir_R5A  atgcaaacag aatattaatg aagtgcttgt tctacagGCT TTGAGACTGT TACATATATG
gTvPir_r7B  atgcaaacag aatattaatg aagtgcttgt tctacagGCT TTGAGACTGT TACATATATG
gTvPir_R2B  atgcaaacag aatattaatg aagtgcttgt tctacagGCT TTGAGACTGT TACATATATG
gTvPir_R3B  atgcaaacag aatattaatg aagtgcttgt tctacagGCT TTGAGACTGT TACATATATG
gTvPir_r4A  atgcaaacag aatattaatg aagtgcttgt tctacagGCT TTGAGACTGT TACATATATG
gTvPir_R5B  atgcaaacag aatattaatg aagtgcttgt tctacagGCT TTGAGACTGT TACATATATG
gTvPir_n2B  atgcaaacag aatattaatg aagtgcttgt tctacagGCT TTGAGACTGT TACATATATG
```

```
             901                                                        960
gTvPir_r1A  TTGCAGgtaa aaacataaat aaatttggag atcttttcgt tagaatccta agactaataa
gTvPir_r2B  TTGCAGgtaa aaacataaat aaatttggag atcttttcgt tagaatccta agactaataa
gTvPir_r3B  TTGCAGgtaa aaacataaat aaatttggag atcttttcgt tagaatccta agactaataa
gTvPir_r5A  TTGCAGgtaa aaacataaat aaatttggag atcttttcgt tagaatccta agactaataa
gTvPir_r8A  TTGCAGgtaa aaacataaat aaatttggag atcttttcgt tagaatccta agactaataa
gTvPir_r8B  TTGCAGgtaa aaacataaat aaatttggag atcttttcgt tagaatccta agactaataa
gTvPir_R1A  TTGCAGgtaa aaacataaat aaatttggag atcttttcgt tagaatccta agactaataa
gTvPir_n1A  TTGCAGgtaa aaacataaat aaatttggag atcttttcgt tagaatccta agactaataa
gTvPir_n2A  TTGCAGgtaa aaacataaat aaatttggag atcttttcgt tagaatccta agactaataa
gTvPir_n1B  TTGCAGgtaa aaacataaat aaatttggag atcttttcgt tagaatccta agactaataa
gTvPir_r2A  TTGCAGgtaa aaacataaat aaatttggag atcttttcgt tagaatccta agactaataa
gTvPir_r3A  TTGCAGgtaa aaacataaat aaatttggag atcttttcgt tagaatccta agactaataa
gTvPir_r5B  TTGCAGgtaa aaacataaat aaatttggag atcttttcgt tagaatccta agactaataa
gTvPir_R1B  TTGCAGgtaa aaacataaat aaatttggag atcttttcgt tagaatccta agactaataa
gTvPir_r7A  TTGCAGgtaa aaacataaat aaatttggag atcttttcgt tagaatccta agactaataa
gTvPir_r1B  TTGCAGgtaa aaacataaat aaatttggag atcttttcgt tagaatccta agactaataa
gTvPir_R6B  TTGCAGgtaa aaacataaat aaatttggag atcttttcgt tagaatccta agactaataa
gTvPir_R4B  TTGCAGgtaa aaacataaat aaatttggag atcttttcgt tagaatccta agactaataa
gTvPir_R6A  TTGCAGgtaa aaacataaat aaatttggag atcttttcgt tagaatccta agactaataa
gTvPir_N2A  TTGCAGgtaa aaacataaat aaatttggag atcttttcgt tagaatccta agactaataa
gTvPir_N2B  TTGCAGgtaa aaacataaat aaatttggag atcttttcgt tagaatccta agactaataa
gTvPir_R2A  TTGCAGgtaa aaacataaat aaatttggag atcttttcgt tagaatccta agactaataa
gTvPir_N1B  TTGCAGgtaa aaacataaat aaatttggag atcttttcgt tagaatccta agactaataa
gTvPir_R4A  TTGCAGgtaa aaacataaat aaatttggag atattttcgt tagaatccta agactaataa
gTvPir_n3A  TTGCAGgtaa aaacataaat aaatttggag atattttcgt tagaatccta agactaataa
gTvPir_N1A  TTGCAGgtaa aaacataaat aaatttggag atattttcgt tagaatccta agactaataa
gTvPir_n3B  TTGCAGgtaa aaacataaat aaatttggag atattttcgt tagaatccta agactaataa
gTvPir_N3B  TTGCAGgtaa aaacataaat aaatttggag atattttcgt tagaatccta agactaataa
gTvPir_r4B  TTGCAGgtaa aaacataaat aaatttggag atcttttcgt tagaatccta agactaataa
gTvPir_r6A  TTGCAGgtaa aaacataaat aaatttggag atcttttcgt tagaatccta agactaataa
gTvPir_R3A  TTGCAGgtaa aaacataaat aaatttggag atcttttcgt tagaatccta agactaataa
gTvPir_N3A  TTGCAGgtaa aaacataaat aaatttggag atcttttcgt tagaatccta agactaataa
gTvPir_r6B  TTGCAGgtaa aaacataaat aaatttggag atcttttcgt tagaatccta agactaataa
gTvPir_R5A  TTGCAGgtaa aaacataaat aaatttggag atcttttcgt tagaatccta agactaataa
gTvPir_r7B  TTGCAGgtaa aaacataaat aaatttggag atcttttcgt tagaatccta agactaataa
gTvPir_R2B  TTGCAGgtaa aaacataaat aaatttggag atcttttcgt tagaatccta agactaataa
gTvPir_R3B  TTGCAGgtaa aaacataaat aaatttggag atcttttcgt tagaatccta agactaataa
gTvPir_r4A  TTGCAGgtaa aaacataaat aaatttggag atattttcgt tagaatccta agactaataa
gTvPir_R5B  TTGCAGgtaa aaacataaat aaatttggag atattttcgt tagaatccta agactaataa
gTvPir_n2B  TTGCAGgtaa aaacataaat aaatttggag atattttcgt tagaatccta agactaataa
```

```
             961                                                         1020
gTvPir_r1A   gaactcaaaa taaattttct tttatttagG GAGGCGTCAC ACATCAAGAT TTTGCCGGTC
gTvPir_r2B   gaactcaaaa taaattttct tttatttagG GAGGCGTCAC ACATCAAGAT TTTGCCGGTC
gTvPir_r3B   gaactcaaaa taaattttct tttatttagG GAGGCGTCAC ACATCAAGAT TTTGCCGGTC
gTvPir_r5A   gaactcaaaa taaattttct tttatttagG GAGGCGTCAC ACATCAAGAT TTTGCCGGTC
gTvPir_r8A   gaactcaaaa taaattttct tttatttagG GAGGCGTCAC ACATCAAGAT TTTGCCGGTC
gTvPir_r8B   gaactcaaaa taaattttct tttatttagG GAGGCGTCAC ACATCAAGAT TTTGCCGGTC
gTvPir_R1A   gaactcaaaa taaattttct tttatttagG GAGGCGTCAC ACATCAAGAT TTTGCCGGTC
gTvPir_n1A   gaactcaaaa taaattttct tttatttagG GAGGCGTCAC ACATCAAGAT TTTGCCGGTC
gTvPir_n2A   gaactcaaaa taaattttct tttatttagG GAGGCGTCAC ACATCAAGAT TTTGCCGGTC
gTvPir_n1B   gaactcaaaa taaattttct tttatttagG GAGGCGTCAC ACATCAAGAT TTTGCCGGTC
gTvPir_r2A   gaactcaaaa taaattttct tttatttagG GAGGCGTCAC ACATCAAGAT TTTGCCGGTC
gTvPir_r3A   gaactcaaaa taaattttct tttatttagG GAGGCGTCAC ACATCAAGAT TTTGCCGGTC
gTvPir_r5B   gaactcaaaa taaattttct tttatttagG GAGGCGTCAC ACATCAAGAT TTTGCCGGTC
gTvPir_R1B   gaactcaaaa taaattttct tttatttagG GAGGCGTCAC ACATCAAGAT TTTGCCGGTC
gTvPir_r7A   gaactcaaaa taaattttct tttatttagG GAGGCGTCAC ACATCAAGAT TTTGCCGGTC
gTvPir_r1B   gaactcaaaa taaattttct tttatttagG GAGGCGTCAC ACATCAAGAT TTTGCCGGTC
gTvPir_R6B   gaactcaaaa taaattttct tttatttagG GAGGCGTCAC ACATCAAGAT TTTGCCGGTC
gTvPir_R4B   gaactcaaaa taaattttct tttatttagG GAGGCGTCAC ACATCAAGAT TTTGCCGGTC
gTvPir_R6A   gaactcaaaa taaattttct tttatttagG GAGGCGTCAC ACATCAAGAT TTTGCCGGTC
gTvPir_N2A   gaactcaaaa taaattttct tttatttagG GAGGCGTCAC ACATCAAGAT TTTGCCGGTC
gTvPir_N2B   gaactcaaaa taaattttct tttatttagG GAGGCGTCAC ACATCAAGAT TTTGCCGGTC
gTvPir_R2A   gaactcaaaa taaattttct tttatttagG GAGGCGTCAC ACATCAAGAT TTTGCCGGTC
gTvPir_N1B   gaactcaaaa taaattttct tttatttagG GAGGCGTCAC ACATCAAGAT TTTGCCGGTC
gTvPir_R4A   gaactcaaaa taaattttct tttatttagG GAGGCGTCAC ACATCAAGAT TTTGCCGGTC
gTvPir_n3A   gaactcaaaa taaattttct tttatttagG GAGGCGTCAC ACATCAAGAT TTTGCCGGTC
gTvPir_N1A   gaactcaaaa taaattttct tttatttagG GAGGCGTCAC ACATCAAGAT TTTGCCGGTC
gTvPir_n3B   gaactcaaaa taaattttct tttatttagG GAGGCGTCAC ACATCAAGAT TTTGCCGGTC
gTvPir_N3B   gaactcaaaa taaattttct tttatttagG GAGGCGTCAC ACATCAAGAT TTTGCCGGTC
gTvPir_r4B   gaactcaaaa taaattttct tttatttagG GAGGCGTCAC ACATCAAGAT TTTGCCGGTC
gTvPir_r6A   gaactcaaaa taaattttct tttatttagG GAGGCGTCAC ACATCAAGAT TTTGCCGGTC
gTvPir_R3A   gaactcaaaa taaattttct tttatttagG GAGGCGTCAC ACATCAAGAT TTTGCCGGTC
gTvPir_N3A   gaactcaaaa taaattttct tttatttagG GAGGCGTCAC ACATCAAGAT TTTGCCGGTC
gTvPir_r6B   gaactcaaaa taaattttct tttatttagG GAGGCGTCAC ACATCAAGAT TTTGCCGGTC
gTvPir_R5A   gaactcaaaa taaattttct tttatttagG GAGGCGTCAC ACATCAAGAT TTTGCCGGTC
gTvPir_r7B   gaactcaaaa taaattttct tttatttagG GAGGCGTCAC ACATCAAGAT TTTGCCGGTC
gTvPir_R2B   gaactcaaaa taaattttct tttatttagG GAGGCGTCAC ACATCAAGAT TTTGCCGGTC
gTvPir_R3B   gaactcaaaa taaattttct tttatttagG GAGGCGTCAC ACATCAAGAT TTTGCCGGTC
gTvPir_r4A   gaactcaaaa taaattttct tttatttagG GAGGCGTCAC ACATCAAGAT TTTGCCGGTC
gTvPir_R5B   gaactcaaaa taaattttct tttatttagG GAGGCGTCAC ACATCAAGAT TTTGCCGGTC
gTvPir_n2B   gaactcaaaa taaattttct tttatttagG GAGGCGTCAC ACATCAAGAT TTTGCCGGTC
```

```
             1021                                                        1080
gTvPir_r1A  ATAAGGGCAC CATAGGTGCT GGTGACGTGC AGgtacagaa taaggatcgt ggcctcgttt
gTvPir_r2B  ATAAGGGCAC CATAGGTGCT GGTGACGTGC AGgtacagaa taaggatcgt ggcctcgttt
gTvPir_r3B  ATAAGGGCAC CATAGGTGCT GGTGACGTGC AGgtacagaa taaggatcgt ggcctcgttt
gTvPir_r5A  ATAAGGGCAC CATAGGTGCT GGTGACGTGC AGgtacagaa taaggatcgt ggcctcgttt
gTvPir_r8A  ATAAGGGCAC CATAGGTGCT GGTGACGTGC AGgtacagaa taaggatcgt ggcctcgttt
gTvPir_r8B  ATAAGGGCAC CATAGGTGCT GGTGACGTGC AGgtacagaa taaggatcgt ggcctcgttt
gTvPir_R1A  ATAAGGGCAC CATAGGTGCT GGTGACGTGC AGgtacagaa taaggatcgt ggcctcgttt
gTvPir_n1A  ATAAGGGCAC CATAGGTGCT GGTGACGTGC AGgtacagaa taaggatcgt ggcctcgttt
gTvPir_n2A  ATAAGGGCAC CATAGGTGCT GGTGACGTGC AGgtacagaa taaggatcgt ggcctcgttt
gTvPir_n1B  ATAAGGGCAC CATAGGTGCT GGTGACGTGC AGgtacagaa taaggatcgt ggcctcgttt
gTvPir_r2A  ATAAGGGCAC CATAGGTGCT GGTGACGTGC AGgtacagaa taaggatcgt ggcctcgttt
gTvPir_r3A  ATAAGGGCAC CATAGGTGCT GGTGACGTGC AGgtacagaa taaggatcgt ggcctcgttt
gTvPir_r5B  ATAAGGGCAC CATAGGTGCT GGTGACGTGC AGgtacagaa taaggatcgt ggcctcgttt
gTvPir_R1B  ATAAGGGCAC CATAGGTGCT GGTGACGTGC AGgtacagaa taaggatcgt ggcctcgttt
gTvPir_r7A  ATAAGGGCAC CATAGGTGCT GGTGACGTGC AGgtacagaa taaggatcgt ggcctcgttt
gTvPir_r1B  ATAAGGGCAC CATAGGTGCT GGTGACGTGC AGgtacagaa taaggatcgt ggcctcgttt
gTvPir_R6B  ATAAGGGCAC CATAGGTGCT GGTGACGTGC AGgtacagaa taaggatcgt ggcctcgttt
gTvPir_R4B  ATAAGGGCAC CATAGGTGCT GGTGACGTGC AGgtacagaa taaggatcgt ggcctcgttt
gTvPir_R6A  ATAAGGGCAC CATAGGTGCT GGTGACGTGC AGgtacagaa taaggatcgt ggcctcgttt
gTvPir_N2A  ATAAGGGCAC CATAGGTGCT GGTGACGTGC AGgtacagaa taaggatcgt ggcctcgttt
gTvPir_N2B  ATAAGGGCAC CATAGGTGCT GGTGACGTGC AGgtacagaa taaggatcgt ggcctcgttt
gTvPir_R2A  ATAAGGGCAC CATAGGTGCT GGTGACGTGC AGgtacagaa taaggatcgt ggcctcgttt
gTvPir_N1B  ATAAGGGCAC CATAGGTGCT GGTGACGTGC AGgtacagaa taaggatcgt ggcctcgttt
gTvPir_R4A  ATAAGGGCAC CATAGGTGCT GGTGACGTGC AGgtacagaa taaggatcgt ggcctcgttt
gTvPir_n3A  ATAAGGGCAC CATAGGTGCT GGTGACGTGC AGgtacagaa taaggatcgt ggcctcgttt
gTvPir_N1A  ATAAGGGCAC CATAGGTGCT GGTGACGTGC AGgtacagaa taaggatcgt ggcctcgttt
gTvPir_n3B  ATAAGGGCAC CATAGGTGCT GGTGACGTGC AGgtacagaa taaggatcgt ggcctcgttt
gTvPir_N3B  ATAAGGGCAC CATAGGTGCT GGTGACGTGC AGgtacagaa taaggatcgt ggcctcgttt
gTvPir_r4B  ATAAGGGCAC CATAGGTGCT GGTGACGTGC AGgtacagaa taaggatcgt ggcctcgttt
gTvPir_r6A  ATAAGGGCAC CATAGGTGCT GGTGACGTGC AGgtacagaa taaggatcgt ggcctcgttt
gTvPir_R3A  ATAAGGGCAC CATAGGTGCT GGTGACGTGC AGgtacagaa taaggatcgt ggcctcgttt
gTvPir_N3A  ATAAGGGCAC CATAGGTGCT GGTGACGTGC AGgtacagaa taaggatcgt ggcctcgttt
gTvPir_r6B  ATAAGGGCAC CATAGGTGCT GGTGACGTGC AGgtacagaa taaggatcgt ggcctcgttt
gTvPir_R5A  ATAAGGGCAC CATAGGTGCT GGTGACGTGC AGgtacagaa taaggatcgt ggcctcgttt
gTvPir_r7B  ATAAGGGCAC CATAGGTGCT GGTGACGTGC AGgtacagaa taaggatcgt ggcctcgttt
gTvPir_R2B  ATAAGGGCAC CATAGGTGCT GGTGACGTGC AGgtacagaa taaggatcgt ggcctcgttt
gTvPir_R3B  ATAAGGGCAC CATAGGTGCT GGTGACGTGC AGgtacagaa taaggatcgt ggcctcgttt
gTvPir_r4A  ATAAGGGCAC CATAGGTGCT GGTGACGTGC AGgtacagaa taaggatcgt ggcctcgttt
gTvPir_R5B  ATAAGGGCAC CATAGGTGCT GGTGACGTGC AGgtacagaa taaggatcgt ggcctcgttt
gTvPir_n2B  ATAAGGGCAC CATAGGTGCT GGTGACGTGC AGgtacagaa taaggatcgt ggcctcgttt
```

```
             1081                                                        1140
gTvPir_r1A  tgattgaaaa atcgatttct atttgaataa taatttgcaa aaatattaag tttaagtctc
gTvPir_r2B  tgattgaaaa atcgatttct atttgaataa taatttgcaa aaatattaag tttaagtctc
gTvPir_r3B  tgattgaaaa atcgatttct atttgaataa taatttgcaa aaatattaag tttaagtctc
gTvPir_r5A  tgattgaaaa atcgatttct atttgaataa taatttgcaa aaatattaag tttaagtctc
gTvPir_r8A  tgattgaaaa atcgatttct atttgaataa taatttgcaa aaatattaag tttaagtctc
gTvPir_r8B  tgattgaaaa atcgatttct atttgaataa taatttgcaa aaatattaag tttaagtctc
gTvPir_R1A  tgattgaaaa atcgatttct atttgaataa taatttgcaa aaatattaag tttaagtctc
gTvPir_n1A  tgattgaaaa atcgatttct atttgaataa taatttgcaa aaatattaag tttaagtctc
gTvPir_n2A  tgattgaaaa atcgatttct atttgaataa taatttgcaa aaatattaag tttaagtctc
gTvPir_n1B  tgattgaaaa atcgatttct atttgaataa taatttgcaa aaatattaag tttaagtctc
gTvPir_r2A  tgattgaaaa atcgatttct atttgaataa taatttgcaa aaatattaag tttaagtctc
gTvPir_r3A  tgattgaaaa atcgatttct atttgaataa taatttgcaa aaatattaag tttaagtctc
gTvPir_r5B  tgattgaaaa atcgatttct atttgaataa taatttgcaa aaatattaag tttaagtctc
gTvPir_R1B  tgattgaaaa atcgatttct atttgaataa taatttgcaa aaatattaag tttaagtctc
gTvPir_r7A  tgattgaaaa atcgatttct atttgaataa taatttgcaa aaatattaag tttaagtctc
gTvPir_r1B  tgattgaaaa atcgatttct atttgaataa taatttgcaa aaatattaag tttaagtctc
gTvPir_R6B  tgattgaaaa atcgatttct atttgaataa taatttgcaa aaatattaag tttaagtctc
gTvPir_R4B  tgattgaaaa atcgatttct atttgaataa taatttgcaa aaatattaag tttaagtctc
gTvPir_R6A  tgattgaaaa atcgatttct atttgaataa taatttgcaa aaatattaag tttaagtctc
gTvPir_N2A  tgattgaaaa atcgatttct atttgaataa taatttgcaa aaatattaag tttaagtctc
gTvPir_N2B  tgattgaaaa atcgatttct atttgaataa taatttgcaa aaatattaag tttaagtctc
gTvPir_R2A  tgattgaaaa atcgatttct atttgaataa taatttgcaa aaatattaag tttaagtctc
gTvPir_N1B  tgattgaaaa atcgatttct atttgaataa taatttgcaa aaatattaag tttaagtctc
gTvPir_R4A  tgattgaaaa atcgatttct atttgaataa taatttgcaa aaatattaag tttaagtctc
gTvPir_n3A  tgattgaaaa atcgatttct atttgaataa taatttgcaa aaatattaag tttaagtctc
gTvPir_N1A  tgattgaaaa atcgatttct atttgaataa taatttgcaa aaatattaag tttaagtctc
gTvPir_n3B  tgattgaaaa atcgatttct atttgaataa taatttgcaa aaatattaag tttaagtctc
gTvPir_N3B  tgattgaaaa atcgatttct atttgaataa taatttgcaa aaatattaag tttaagtctc
gTvPir_r4B  tgattgaaaa atcgatttct atttgaataa taatttgcaa aaatattaag tttaagtctc
gTvPir_r6A  tgattgaaaa atcgatttct atttgaataa taatttgcaa aaatattaag tttaagtctc
gTvPir_R3A  tgattgaaaa atcgatttct atttgaataa taatttgcaa aaatattaag tttaagtctc
gTvPir_N3A  tgattgaaaa atcgatttct atttgaataa taatttgcaa aaatattaag tttaagtctc
gTvPir_r6B  tgattgaaaa atcgatttct atttgaataa taatttgcaa aaatattaag tttaagtctc
gTvPir_R5A  tgattgaaaa atcgatttct atttgaataa taatttgcaa aaatattaag tttaagtctc
gTvPir_r7B  tgattgaaaa atcgatttct atttgaataa taatttgcaa aaatattaag tttaagtctc
gTvPir_R2B  tgattgaaaa atcgatttct atttgaataa taatttgcaa aaatattaag tttaagtctc
gTvPir_R3B  tgattgaaaa atcgatttct atttgaataa taatttgcaa aaatattaag tttaagtctc
gTvPir_r4A  tgattgaaaa atcgatttct atttgaataa taatttgcaa aaatattaag tttaagtctc
gTvPir_R5B  tgattgaaaa atcgatttct atttgaataa taatttgcaa aaatattaag tttaagtctc
gTvPir_n2B  tgattgaaaa atcgatttct atttgaataa taatttgcaa aaatattaag tttaagtctc
```

```
             1141                                                       1200
gTvPir_r1A  tgcttttgag aataagagta tcacaatagg agagctttct tttgtcctag tcaggtcaac
gTvPir_r2B  tgcttttgag aataagagta tcacaatagg agagctttct tttgtcctag tcaggtcaac
gTvPir_r3B  tgcttttgag aataagagta tcacaatagg agagctttct tttgtcctag tcaggtcaac
gTvPir_r5A  tgcttttgag aataagagta tcacaatagg agagctttct tttgtcctag tcaggtcaac
gTvPir_r8A  tgcttttgag aataagagta tcacaatagg agagctttct tttgtcctag tcaggtcaac
gTvPir_r8B  tgcttttgag aataagagta tcacaatagg agagctttct tttgtcctag tcaggtcaac
gTvPir_R1A  tgcttttgag aataagagta tcacaatagg agagctttct tttgtcctag tcaggtcaac
gTvPir_n1A  tgcttttgag aataagagta tcacaatagg agagctttct tttgtcctag tcaggtcaac
gTvPir_n2A  tgcttttgag aataagagta tcacaatagg agagctttct tttgtcctag tcaggtcaac
gTvPir_n1B  tgcttttgag aataagagta tcacaatagg agagctttct tttgtcctag tcaggtcaac
gTvPir_r2A  tgcttttgag aataagagta tcacaatagg agagctttct tttgtcctag tcaggtcaac
gTvPir_r3A  tgcttttgag aataagagta tcacaatagg agagctttct tttgtcctag tcaggtcaac
gTvPir_r5B  tgcttttgag aataagagta tcacaatagg agagctttct tttgtcctag tcaggtcaac
gTvPir_R1B  tgcttttgag aataagagta tcacaatagg agagctttct tttgtcctag tcaggtcaac
gTvPir_r7A  tgcttttgag aataagagta tcacaatagg agagctttct tttgtcctag tcaggtcaac
gTvPir_r1B  tgcttttgag aataagagta tcacaatagg agagctttct tttgtcctag tcaggtcaac
gTvPir_R6B  tgcttttgag aataagagta tcacaatagg agagctttct tttgtcctag tcaggtcaac
gTvPir_R4B  tgcttttgag aataagagta tcacaatagg agagctttct tttgtcctag tcaggtcaac
gTvPir_R6A  tgcttttgag aataagagta tcacaatagg agagctttct tttgtcctag tcaggtcaac
gTvPir_N2A  tgcttttgag aataagagta tcacaatagg agagctttct tttgtcctag tcaggtcaac
gTvPir_N2B  tgcttttgag aataagagta tcacaatagg agagctttct tttgtcctag tcaggtcaac
gTvPir_R2A  tgcttttgag aataagagta tcacaatagg agagctttct tttgtcctag tcaggtcaac
gTvPir_N1B  tgcttttgag aataagagta tcacaatagg agagctttct tttgtcctag tcaggtcaac
gTvPir_R4A  tgcttttgag aataagagta tcacaatagg agagctttct tttgtcctag tcaggtcaac
gTvPir_n3A  tgcttttgag aataagagta tcacaatagg agagctttct tttgtcctag tcaggtcaac
gTvPir_N1A  tgcttttgag aataagagta tcacaatagg agagctttct tttgtcctag tcaggtcaac
gTvPir_n3B  tgcttttgag aataagagta tcacaatagg agagctttct tttgtcctag tcaggtcaac
gTvPir_N3B  tgcttttgag aataagagta tcacaatagg agagctttct tttgtcctag tcaggtcaac
gTvPir_r4B  tgcttttgag aataagagta tcacaatagg agagctttct tttgtcctag tcaggtcaac
gTvPir_r6A  tgcttttgag aataagagta tcacaatagg agagctttct tttgtcctag tcaggtcaac
gTvPir_R3A  tgcttttgag aataagagta tcacaatagg agagctttct tttgtcctag tcaggtcaac
gTvPir_N3A  tgcttttgag aataagagta tcacaatagg agagctttct tttgtcctag tcaggtcaac
gTvPir_r6B  tgcttttgag aataagagta tcacaatagg agagctttct tttgtcctag tcaggtcaac
gTvPir_R5A  tgcttttgag aataagagta tcacaatagg agagctttct tttgtcctag tcaggtcaac
gTvPir_r7B  tgcttttgag aataagagta tcacaatagg agagctttct tttgtcctag tcaggtcaac
gTvPir_R2B  tgcttttgag aataagagta tcacaatagg agagctttct tttgtcctag tcaggtcaac
gTvPir_R3B  tgcttttgag aataagagta tcacaatagg agagctttct tttgtcctag tcaggtcaac
gTvPir_r4A  tgcttttgag aataagagta tcacaatagg agagctttct tttgtcctag tcaggtcaac
gTvPir_R5B  tgcttttgag aataagagta tcacaatagg agagctttct tttgtcctag tcaggtcaac
gTvPir_n2B  tgcttttgag aataagagta tcacaatagg agagctttct tttgtcctag tcaggtcaac
```

```
            1201                                                          1260
gTvPir_r1A  gaaggcatgg aatatttcga tttttctaat actgaaacca agttatagta catgatttat
gTvPir_r2B  gaaggcatgg aatatttcga tttttctaat actgaaacca agttatagta catgatttat
gTvPir_r3B  gaaggcatgg aatatttcga tttttctaat actgaaacca agttatagta catgatttat
gTvPir_r5A  gaaggcatgg aatatttcga tttttctaat actgaaacca agttatagta catgatttat
gTvPir_r8A  gaaggcatgg aatatttcga tttttctaat actgaaacca agttatagta catgatttat
gTvPir_r8B  gaaggcatgg aatatttcga tttttctaat actgaaacca agttatagta catgatttat
gTvPir_R1A  gaaggcatgg aatatttcga tttttctaat actgaaacca agttatagta catgatttat
gTvPir_n1A  gaaggcatgg aatatttcga tttttctaat actgaaacca agttatagta catgatttat
gTvPir_n2A  gaaggcatgg aatatttcga tttttctaat actgaaacca agttatagta catgatttat
gTvPir_n1B  gaaggcatgg aatatttcga tttttctaat actgaaacca agttatagta catgatttat
gTvPir_r2A  gaaggcatgg aatatttcga tttttctaat actgaaacca agttatagta catgatttat
gTvPir_r3A  gaaggcatgg aatatttcga tttttctaat actgaaacca agttatagta catgatttat
gTvPir_r5B  gaaggcatgg aatatttcga tttttctaat actgaaacca agttatagta catgatttat
gTvPir_R1B  gaaggcatgg aatatttcga tttttctaat actgaaacca agttatagta catgatttat
gTvPir_r7A  gaaggcatgg aatatttcga tttttctaat actgaaacca agttatagta catgatttat
gTvPir_r1B  gaaggcatgg aatatttcga tttttctaat actgaaacca agttatagta catgatttat
gTvPir_R6B  gaaggcatgg aatatttcga tttttctaat actgaaacca agttatagta catgatttat
gTvPir_R4B  gaaggcatgg aatatttcga tttttctaat actgaaacca agttatagta catgatttat
gTvPir_R6A  gaaggcatgg aatatttcga tttttctaat actgaaacca agttatagta catgatttat
gTvPir_N2A  gaaggcatgg aatatttcga tttttctaat actgaaacca agttatagta catgatttat
gTvPir_N2B  gaaggcatgg aatatttcga tttttctaat actgaaacca agttatagta catgatttat
gTvPir_R2A  gaaggcatgg aatatttcga tttttctaat actgaaacca agttatagta catgatttat
gTvPir_N1B  gaaggcatgg aatatttcga tttttctaat actgaaacca agttatagta catgatttat
gTvPir_R4A  gaaggcatgg aatatttcga tttttctaat actgaaacca agttatagta catgatttat
gTvPir_n3A  gaaggcatgg aatatttcga tttttctaat actgaaacca agttatagta catgatttat
gTvPir_N1A  gaaggcatgg aatatttcga tttttctaat actgaaacca agttatagta catgatttat
gTvPir_n3B  gaaggcatgg aatatttcga tttttctaat actgaaacca agttatagta catgatttat
gTvPir_N3B  gaaggcatgg aatatttcga tttttctaat actgaaacca agttatagta catgatttat
gTvPir_r4B  gaaggcatgg aatatttcga tttttctaat actgaaacca agttatagta catgatttat
gTvPir_r6A  gaaggcatgg aatatttcga tttttctaat actgaaacca agttatagta catgatttat
gTvPir_R3A  gaaggcatgg aatatttcga tttttctaat actgaaacca agttatagta catgatttat
gTvPir_N3A  gaaggcatgg aatatttcga tttttctaat actgaaacca agttatagta catgatttat
gTvPir_r6B  gaaggcatgg aatatttcga tttttctaat actgaaacca agttatagta catgatttat
gTvPir_R5A  gaaggcatgg aatatttcga tttttctaat actgaaacca agttatagta catgatttat
gTvPir_r7B  gaaggcatgg aatatttcga tttttctaat actgaaacca agttatagta catgatttat
gTvPir_R2B  gcaggcatgg aatatttcga tttttctaat actgaaacca agttatagta catgatttat
gTvPir_R3B  gaaggcatgg aatatttcga tttttctaat actgaaacca agttatagta catgatttat
gTvPir_r4A  gaaggcatgg aatatttcga tttttctaat actgaaacca agttatagta catgatttat
gTvPir_R5B  gtaggcatgg aatatttcga tttttctaat actgaaacca agttatagta catgatttat
gTvPir_n2B  gaaggcatgg aatatttcga tttttctaat actgaaacca agttatagta catgatttat
```

	1261					1320
gTvPir_r1A	ctcaacaggc	gaataattaa	caatactaat	ttgtgtacaa	ctttcttgtt	ttggaaaatc
gTvPir_r2B	ctcaacaggc	gaataattaa	caatactaat	ttgtgtacaa	ctttcttgtt	ttggaaaatc
gTvPir_r3B	ctcaacaggc	gaataattaa	caatactaat	ttgtgtacaa	ctttcttgtt	ttggaaaatc
gTvPir_r5A	ctcaacaggc	gaataattaa	caatactaat	ttgtgtacaa	ctttcttgtt	ttggaaaatc
gTvPir_r8A	ctcaacaggc	gaataattaa	caatactaat	ttgtgtacaa	ctttcttgtt	ttggaaaatc
gTvPir_r8B	ctcaacaggc	gaataattaa	caatactaat	ttgtgtacaa	ctttcttgtt	ttggaaaatc
gTvPir_R1A	ctcaacaggc	gaataattaa	caatactaat	ttgtgtacaa	ctttcttgtt	ttggaaaatc
gTvPir_n1A	ctcaacaggc	gaataattaa	caatactaat	ttgtgtacaa	ctttcttgtt	ttggaaaatc
gTvPir_n2A	ctcaacaggc	gaataattaa	caatactaat	ttgtgtacaa	ctttcttgtt	ttggaaaatc
gTvPir_n1B	ctcaacaggc	gaataattaa	caatactaat	ttgtgtacaa	ctttcttgtt	ttggaaaatc
gTvPir_r2A	ctcaacaggc	gaataattaa	caatactaat	ttgtgtacaa	ctttcttgtt	ttggaaaatc
gTvPir_r3A	ctcaacaggc	gaataattaa	caatactaat	ttgtgtacaa	ctttcttgtt	ttggaaaatc
gTvPir_r5B	ctcaacaggc	gaataattaa	caatactaat	ttgtgtacaa	ctttcttgtt	ttggaaaatc
gTvPir_R1B	ctcaacaggc	gaataattaa	caatactaat	ttgtgtacaa	ctttcttgtt	ttggaaaatc
gTvPir_r7A	ctcaacaggc	gaataattaa	caatactaat	ttgtgtacaa	ctttcttgtt	ttggaaaatc
gTvPir_r1B	ctcaacaggc	gaataattaa	caatactaat	ttgtgtacaa	ctttcttgtt	ttggaaaatc
gTvPir_R6B	ctcaacaggc	gaataattaa	caatactaat	ttgtgtacaa	ctttcttgtt	ttggaaaatc
gTvPir_R4B	ctcaacaggc	gaataattaa	caatactaat	ttgtgtacaa	ctttcttgtt	ttggaaaatc
gTvPir_R6A	ctcaacaggc	gaataattaa	caatactaat	ttgtgtacaa	ctttcttgtt	ttggaaaatc
gTvPir_N2A	ctcaacaggc	gaataattaa	caatactaat	ttgtgtacaa	ctttcttgtt	ttggaaaatc
gTvPir_N2B	ctcaacaggc	gaataattaa	caatactaat	ttgtgtacaa	ctttcttgtt	ttggaaaatc
gTvPir_R2A	ctcaacaggc	gaataattaa	caatactaat	ttgtgtacaa	ctttcttgtt	ttggaaaatc
gTvPir_N1B	ctcaacaggc	gaataattaa	caatactaat	ttgtgtacaa	ctttcttgtt	ttggaaaatc
gTvPir_R4A	ctcaacaggc	gaataattaa	caatactaat	ttgtgtacaa	ctttcttgtt	ttggaaaatc
gTvPir_n3A	ctcaacaggc	gaataattaa	caatactaat	ttgtgtacaa	ctttcttgtt	ttggaaaatc
gTvPir_N1A	ctcaacaggc	gaataattaa	caatactaat	ttgtgtacaa	ctttcttgtt	ttggaaaatc
gTvPir_n3B	ctcaacaggc	gaataattaa	caatactaat	ttgtgtacaa	ctttcttgtt	ttggaaaatc
gTvPir_N3B	ctcaacaggc	gaataattaa	caatactaat	ttgtgtacaa	ctttcttgtt	ttggaaaatc
gTvPir_r4B	ctcaacaggc	gaataattaa	caatactaat	ttgtgtacaa	ctttcttgtt	ttggaaaatc
gTvPir_r6A	ctcaacaggc	gaataattaa	caatactaat	ttgtgtacaa	ctttcttgtt	ttggaaaatc
gTvPir_R3A	ctcaacaggc	gaataattaa	caatactaat	ttgtgtacaa	ctttcttgtt	ttggaaaatc
gTvPir_N3A	ctcaacaggc	gaataattaa	caatactaat	ttgtgtacaa	ctttcttgtt	ttggaaaatc
gTvPir_r6B	ctcaacaggc	gaataattaa	caatactaat	ttgtgtacaa	ctttcttgtt	ttggaaaatc
gTvPir_R5A	ctcaacaggc	gaataattaa	caatactaat	ttgtgtacaa	ctttcttgtt	ttggaaaatc
gTvPir_r7B	ctcaacaggc	gaataattaa	caatactaat	ttgtgtacaa	ctttcttgtt	ttggaaaatc
gTvPir_R2B	ctcaacaggc	gaataattaa	caatactaat	ttgtgtacaa	ctttcttgtt	ttggaaaatc
gTvPir_R3B	ctcaacaggc	gaataattaa	caatactaat	ttgtgtacaa	ctttcttgtt	ttggaaaatc
gTvPir_r4A	ctcaacaggc	gaataattaa	caatactaat	ttgtgtacaa	ctttcttgtt	ttggaaaatc
gTvPir_R5B	ctcaacaggc	gaataattaa	caatactaat	ttgtgtacaa	ctttcttgtt	ttggaaaatc
gTvPir_n2B	ctcaacaggc	gaataattaa	caatactaat	ttgtgtacaa	ctttcttgtt	ttggaaaatc

```
             1321                                                   1380
gTvPir_r1A   acagTGGATG ACTGCCGGCA GAGGTATTGT TCACTCAGAA ATGCCCGCCG GAGAAGGCAC
gTvPir_r2B   acagTGGATG ACTGCCGGCA GAGGTATTGT TCACTCAGAA ATGCCCGCCG GAGAAGGCAC
gTvPir_r3B   acagTGGATG ACTGCCGGCA GAGGTATTGT TCACTCAGAA ATGCCCGCCG GAGAAGGCAC
gTvPir_r5A   acagTGGATG ACTGCCGGCA GAGGTATTGT TCACTCAGAA ATGCCCGCCG GAGAAGGCAC
gTvPir_r8A   acagTGGATG ACTGCCGGCA GAGGTATTGT TCACTCAGAA ATGCCCGCCG GAGAAGGCAC
gTvPir_r8B   acagTGGATG ACTGCCGGCA GAGGTATTGT TCACTCAGAA ATGCCCGCCG GAGAAGGCAC
gTvPir_R1A   acagTGGATG ACTGCCGGCA GAGGTATTGT TCACTCAGAA ATGCCCGCCG GAGAAGGCAC
gTvPir_n1A   acagTGGATG ACTGCCGGCA GAGGTATTGT TCACTCAGAA ATGCCCGCCG GAGAAGGCAC
gTvPir_n2A   acagTGGATG ACTGCCGGCA GAGGTATTGT TCACTCAGAA ATGCCCGCCG GAGAAGGCAC
gTvPir_n1B   acagTGGATG ACTGCCGGCA GAGGTATTGT TCACTCAGAA ATGCCCGCCG GAGAAGGCAC
gTvPir_r2A   acagTGGATG ACTGCCGGCA GAGGTATTGT TCACTCAGAA ATGCCCGCCG GAGAAGGCAC
gTvPir_r3A   acagTGGATG ACTGCCGGCA GAGGTATTGT TCACTCAGAA ATGCCCGCCG GAGAAGGCAC
gTvPir_r5B   acagTGGATG ACTGCCGGCA GAGGTATTGT TCACTCAGAA ATGCCCGCCG GAGAAGGCAC
gTvPir_R1B   acagTGGATG ACTGCCGGCA GAGGTATTGT TCACTCAGAA ATGCCCGCCG GAGAAGGCAC
gTvPir_r7A   acagTGGATG ACTGCCGGCA GAGGTATTGT TCACTCAGAA ATGCCCGCCG GAGAAGGCAC
gTvPir_r1B   acagTGGATG ACTGCCGGCA GAGGTATTGT TCACTCAGAA ATGCCCGCCG GAGAAGGCAC
gTvPir_R6B   acagTGGATG ACTGCCGGCA GAGGTATTGT TCACTCAGAA ATGCCCGCCG GAGAAGGCAC
gTvPir_R4B   acagTGGATG ACTGCCGGCA GAGGTATTGT TCACTCAGAA ATGCCCGCCG GAGAAGGCAC
gTvPir_R6A   acagTGGATG ACTGCCGGCA GAGGTATTGT TCACTCAGAA ATGCCCGCCG GAGAAGGCAC
gTvPir_N2A   acagTGGATG ACTGCCGGCA GAGGTATTGT TCACTCAGAA ATGCCCGCCG GAGAAGGCAC
gTvPir_N2B   acagTGGATG ACTGCCGGCA GAGGTATTGT TCACTCAGAA ATGCCCGCCG GAGAAGGCAC
gTvPir_R2A   acagTGGATG ACTGCCGGCA GAGGTATTGT TCACTCAGAA ATGCCCGCCG GAGAAGGCAC
gTvPir_N1B   acagTGGATG ACTGCCGGCA GAGGTATTGT TCACTCAGAA ATGCCCGCCG GAGAAGGCAC
gTvPir_R4A   acagTGGATG ACTGCCGGCA GAGGTATTGT TCACTCAGAA ATGCCCGCCG GAGAAGGCAC
gTvPir_n3A   acagTGGATG ACTGCCGGCA GAGGTATTGT TCACTCAGAA ATGCCCGCCG GAGAAGGCAC
gTvPir_N1A   acagTGGATG ACTGCCGGCA GAGGTATTGT TCACTCAGAA ATGCCCGCCG GAGAAGGCAC
gTvPir_n3B   acagTGGATG ACTGCCGGCA GAGGTATTGT TCACTCAGAA ATGCCCGCCG GAGAAGGCAC
gTvPir_N3B   acagTGGATG ACTGCCGGCA GAGGTATTGT TCACTCAGAA ATGCCCGCCG GAGAAGGCAC
gTvPir_r4B   acagTGGATG ACTGCCGGCA GAGGTATTGT TCACTCAGAA ATGCCCGCCG GAGAAGGCAC
gTvPir_r6A   acagTGGATG ACTGCCGGCA GAGGTATTGT TCACTCAGAA ATGCCCGCCG GAGAAGGCAC
gTvPir_R3A   acagTGGATG ACTGCCGGCA GAGGTATTGT TCACTCAGAA ATGCCCGCCG GAGAAGGCAC
gTvPir_N3A   acagTGGATG ACTGCCGGCA GAGGTATTGT TCACTCAGAA ATGCCCGCCG GAGAAGGCAC
gTvPir_r6B   acagTGGATG ACTGCCGGCA GAGGTATTGT TCACTCAGAA ATGCCCGCCG GAGAAGGCAC
gTvPir_R5A   acagTGGATG ACTGCCGGCA GAGGTATTGT TCACTCAGAA ATGCCCGCCG GAGAAGGCAC
gTvPir_r7B   acagTGGATG ACTGCCGGCA GAGGTATTGT TCACTCAGAA ATGCCCGCCG GAGAAGGCAC
gTvPir_R2B   acagTGGATG ACTGCCGGCA GAGGTATTGT TCACTCAGAA ATGCCCGCCG GAGAAGGCAC
gTvPir_R3B   acagTGGATG ACTGCCGGCA GAGGTATTGT TCACTCAGAA ATGCCCGCCG GAGAAGGCAC
gTvPir_r4A   acagTGGATG ACTGCCGGCA GAGGTATTGT TCACTCAGAA ATGCCCGCCG GAGAAGGCAC
gTvPir_R5B   acagTGGATG ACTGCCGGCA GAGGTATTGT TCACTCAGAA ATGCCCGCCG GAGAAGGCAC
gTvPir_n2B   acagTGGATG ACTGCCGGCA GAGGTATTGT TCACTCAGAA ATGCCCGCCG GAGAAGGCAC
```

```
            1381                                                         1440
gTvPir_r1A  TCACACTGGT CTCCAATTGT GGATCAATCT CTCTTCCAAG GACAAAATgt gagccttatt
gTvPir_r2B  TCACACTGGT CTCCAATTGT GGATCAATCT CTCTTCCAAG GACAAAATgt gagccttatt
gTvPir_r3B  TCACACTGGT CTCCAATTGT GGATCAATCT CTCTTCCAAG GACAAAATgt gagccttatt
gTvPir_r5A  TCACACTGGT CTCCAATTGT GGATCAATCT CTCTTCCAAG GACAAAATgt gagccttatt
gTvPir_r8A  TCACACTGGT CTCCAATTGT GGATCAATCT CTCTTCCAAG GACAAAATgt gagccttatt
gTvPir_r8B  TCACACTGGT CTCCAATTGT GGATCAATCT CTCTTCCAAG GACAAAATgt gagccttatt
gTvPir_R1A  TCACACTGGT CTCCAATTGT GGATCAATCT CTCTTCCAAG GACAAAATgt gagccttatt
gTvPir_n1A  TCACACTGGT CTCCAATTGT GGATCAATCT CTCTTCCAAG GACAAAATgt gagccttatt
gTvPir_n2A  TCACACTGGT CTCCAATTGT GGATCAATCT CTCTTCCAAG GACAAAATgt gagccttatt
gTvPir_n1B  TCACACTGGT CTCCAATTGT GGATCAATCT CTCTTCCAAG GACAAAATgt gagccttatt
gTvPir_r2A  TCACACTGGT CTCCAATTGT GGATCAATCT CTCTTCCAAG GACAAAATgt gagccttatt
gTvPir_r3A  TCACACTGGT CTCCAATTGT GGATCAATCT CTCTTCCAAG GACAAAATgt gagccttatt
gTvPir_r5B  TCACACTGGT CTCCAATTGT GGATCAATCT CTCTTCCAAG GACAAAATgt gagccttatt
gTvPir_R1B  TCACACTGGT CTCCAATTGT GGATCAATCT CTCTTCCAAG GACAAAATgt gagccttatt
gTvPir_r7A  TCACACTGGT CTCCAATTGT GGATCAATCT CTCTTCCAAG GACAAAATgt gagccttatt
gTvPir_r1B  TCACACTGGT CTCCAATTGT GGATCAATCT CTCTTCCAAG GACAAAATgt gagccttatt
gTvPir_R6B  TCACACTGGT CTCCAATTGT GGATCAATCT CTCTTCCAAG GACAAAATgt gagccttatt
gTvPir_R4B  TCACACTGGT CTCCAATTGT GGATCAATCT CTCTTCCAAG GACAAAATgt gagccttatt
gTvPir_R6A  TCACACTGGT CTCCAATTGT GGATCAATCT CTCTTCCAAG GACAAAATgt gagccttatt
gTvPir_N2A  TCACACTGGT CTCCAATTGT GGATCAATCT CTCTTCCAAG GACAAAATgt gagccttatt
gTvPir_N2B  TCACACTGGT CTCCAATTGT GGATCAATCT CTCTTCCAAG GACAAAATgt gagccttatt
gTvPir_R2A  TCACACTGGT CTCCAATTGT GGATCAATCT CTCTTCCAAG GACAAAATgt gagccttatt
gTvPir_N1B  TCACACTGGT CTCCAATTGT GGATCAATCT CTCTTCCAAG GACAAAATgt gagccttatt
gTvPir_R4A  TCACACTGGT CTCCAATTGT GGATCAATCT CTCTTCCAAG GACAAAATgt gagccttatt
gTvPir_n3A  TCACACTGGT CTCCAATTGT GGATCAATCT CTCTTCCAAG GACAAAATgt gagccttatt
gTvPir_N1A  TCACACTGGT CTCCAATTGT GGATCAATCT CTCTTCCAAG GACAAAATgt gagccttatt
gTvPir_n3B  TCACACTGGT CTCCAATTGT GGATCAATCT CTCTTCCAAG GACAAAATgt gagccttatt
gTvPir_N3B  TCACACTGGT CTCCAATTGT GGATCAATCT CTCTTCCAAG GACAAAATgt gagccttatt
gTvPir_r4B  TCACACTGGT CTCCAATTGT GGATCAATCT CTCTTCCAAG GACAAAATgt gagccttatt
gTvPir_r6A  TCACACTGGT CTCCAATTGT GGATCAATCT CTCTTCCAAG GACAAAATgt gagccttatt
gTvPir_R3A  TCACACTGGT CTCCAATTGT GGATCAATCT CTCTTCCAAG GACAAAATgt gagccttatt
gTvPir_N3A  TCACACTGGT CTCCAATTGT GGATCAATCT CTCTTCCAAG GACAAAATgt gagccttatt
gTvPir_r6B  TCACACTGGT CTCCAATTGT GGATCAATCT CTCTTCCAAG GACAAAATgt gagccttatt
gTvPir_R5A  TCACACTGGT CTCCAATTGT GGATCAATCT CTCTTCCAAG GACAAAATgt gagccttatt
gTvPir_r7B  TCACACTGGT CTCCAATTGT GGATCAATCT CTCTTCCAAG GACAAAATgt gagccttatt
gTvPir_R2B  TCACACTGGT CTCCAATTGT GGATCAATCT CTCTTCCAAG GACAAAATgt gagccttatt
gTvPir_R3B  TCACACTGGT CTCCAATTGT GGATCAATCT CTCTTCCAAG GACAAAATgt gagccttatt
gTvPir_r4A  TCACACTGGT CTCCAATTGT GGATCAATCT CTCTTCCAAG GACAAAATgt gagccttatt
gTvPir_R5B  TCACACTGGT CTCCAATTGT GGATCAATCT CTCTTCCAAG GACAAAATgt gagccttatt
gTvPir_n2B  TCACACTGGT CTCCAATTGT GGATCAATCT CTCTTCCAAG GACAAAATgt gagccttatt
```

```
            1441                                                       1500
gTvPir_r1A  atgttcttaa tttatcgggc ataacaaatg tagtgatatg tctatatttg tgggaaatgc
gTvPir_r2B  atgttcttaa tttatcgggc ataacaaatg tagtgatatg tctatatttg tgggaaatgc
gTvPir_r3B  atgttcttaa tttatcgggc ataacaaatg tagtgatatg tctatatttg tgggaaatgc
gTvPir_r5A  atgttcttaa tttatcgggc ataacaaatg tagtgatatg tctatatttg tgggaaatgc
gTvPir_r8A  atgttcttaa tttatcgggc ataacaaatg tagtgatatg tctatatttg tgggaaatgc
gTvPir_r8B  atgttcttaa tttatcgggc ataacaaatg tagtgatatg tctatatttg tgggaaatgc
gTvPir_R1A  atgttcttaa tttatcgggc ataacaaatg tagtgatatg tctatatttg tgggaaatgc
gTvPir_n1A  atgttcttaa tttatcgggc ataacaaatg tagtgatatg tctatatttg tgggaaatgc
gTvPir_n2A  atgttcttaa tttatcgggc ataacaaatg tagtgatatg tctatatttg tgggaaatgc
gTvPir_n1B  atgttcttaa tttatcgggc ataacaaatg tagtgatatg tctatatttg tgggaaatgc
gTvPir_r2A  atgttcttaa tttatcgggc ataacaaatg tagtgatatg tctatatttg tgggaaatgc
gTvPir_r3A  atgttcttaa tttatcgggc ataacaaatg tagtgatatg tctatatttg tgggaaatgc
gTvPir_r5B  atgttcttaa tttatcgggc ataacaaatg tagtgatatg tctatatttg tgggaaatgc
gTvPir_R1B  atgttcttaa tttatcgggc ataacaaatg tagtgatatg tctatatttg tgggaaatgc
gTvPir_r7A  atgttcttaa tttatcgggc ataacaaatg tagtgatatg tctatatttg tgggaaatgc
gTvPir_r1B  atgttcttaa tttatcgggc ataacaaatg tagtgatatg tctatatttg tgggaaatgc
gTvPir_R6B  atgttcttaa tttatcgggc ataacaaatg tagtgatatg tctatatttg tgggaaatgc
gTvPir_R4B  atgttcttaa tttatcgggc ataacaaatg tagtgatatg tctatatttg tgggaaatgc
gTvPir_R6A  atgttcttaa tttatcgggc ataacaaatg tagtgatatg tctatatttg tgggaaatgc
gTvPir_N2A  atgttcttaa tttatcgggc ataacaaatg tagtgatatg tctatatttg tgggaaatgc
gTvPir_N2B  atgttcttaa tttatcgggc ataacaaatg tagtgatatg tctatatttg tgggaaatgc
gTvPir_R2A  atgttcttaa tttatcgggc ataacaaatg tagtgatatg tctatatttg tgggaaatgc
gTvPir_N1B  atgttcttaa tttatcgggc ataacaaatg tagtgatatg tctatatttg tgggaaatgc
gTvPir_R4A  atgttcttaa tttatcgggc ataacaaatg tagtgatatg tctatatttg tgggaaatgc
gTvPir_n3A  atgttcttaa tttatcgggc ataacaaatg tagtgatatg tctatatttg tgggaaatgc
gTvPir_N1A  atgttcttaa tttatcgggc ataacaaatg tagtgatatg tctatatttg tgggaaatgc
gTvPir_n3B  atgttcttaa tttatcgggc ataacaaatg tagtgatatg tctatatttg tgggaaatgc
gTvPir_N3B  atgttcttaa tttatcgggc ataacaaatg tagtgatatg tctatatttg tgggaaatgc
gTvPir_r4B  atgttcttaa tttatcgggc ataacaaatg tagtgatatg tctatatttg tgggaaatgc
gTvPir_r6A  atgttcttaa tttatcgggc ataacaaatg tagtgatatg tctatatttg tgggaaatgc
gTvPir_R3A  atgttcttaa tttatcgggc ataacaaatg tagtgatatg tctatatttg tgggaaatgc
gTvPir_N3A  atgttcttaa tttatcgggc ataacaaatg tagtgatatg tctatatttg tgggaaatgc
gTvPir_r6B  atgttcttaa tttatcgggc ataacaaatg tagtgatatg tctatatttg tgggaaatgc
gTvPir_R5A  atgttcttaa tttatcgggc ataacaaatg tagtgatatg tctatatttg tgggaaatgc
gTvPir_r7B  atgttcttaa tttatcgggc ataacaaatg tagtgatatg tctatatttg tgggaaatgc
gTvPir_R2B  atgttcttaa tttatcgggc ataacaaatg tagtgatatg tctatatttg tgggaaatgc
gTvPir_R3B  atgttcttaa tttatcgggc ataacaaatg tagtgatatg tctatatttg tgggaaatgc
gTvPir_r4A  atgttcttaa tttatcgggc ataacaaatg tagtgatatg tctatatttg tgggaaatgc
gTvPir_R5B  atgttcttaa tttatcgggc ataacaaatg tagtgatatg tctatatttg tgggaaatgc
gTvPir_n2B  atgttcttaa tttatcgggc ataacaaatg tagtgatatg tctatatttg tgggaaatgc
```

```
            1501                                                   1560
gTvPir_r1A  gaaatagtaa cacaaccagt cctgcagGAT TGAACCTAGG TATCAAGAAT TGCTGAGTAA
gTvPir_r2B  gaaatagtaa cacaaccagt cctgcagGAT TGAACCTAGG TATCAAGAAT TGCTGAGTAA
gTvPir_r3B  gaaatagtaa cacaaccagt cctgcagGAT TGAACCTAGG TATCAAGAAT TGCTGAGTAA
gTvPir_r5A  gaaatagtaa cacaaccagt cctgcagGAT TGAACCTAGG TATCAAGAAT TGCTGAGTAA
gTvPir_r8A  gaaatagtaa cacaaccagt cctgcagGAT TGAACCTAGG TATCAAGAAT TGCTGAGTAA
gTvPir_r8B  gaaatagtaa cacaaccagt cctgcagGAT TGAACCTAGG TATCAAGAAT TGCTGAGTAA
gTvPir_R1A  gaaatagtaa cacaaccagt cctgcagGAT TGAACCTAGG TATCAAGAAT TGCTGAGTAA
gTvPir_n1A  gaaatagtaa cacaaccagt cctgcagGAT TGAACCTAGG TATCAAGAAT TGCTGAGTAA
gTvPir_n2A  gaaatagtaa cacaaccagt cctgcagGAT TGAACCTAGG TATCAAGAAT TGCTGAGTAA
gTvPir_n1B  gaaatagtaa cacaaccagt cctgcagGAT TGAACCTAGG TATCAAGAAT TGCTGAGTAA
gTvPir_r2A  gaaatagtaa cacaaccagt cctgcagGAT TGAACCTAGG TATCAAGAAT TGCTGAGTAA
gTvPir_r3A  gaaatagtaa cacaaccagt cctgcagGAT TGAACCTAGG TATCAAGAAT TGCTGAGTAA
gTvPir_r5B  gaaatagtaa cacaaccagt cctgcagGAT TGAACCTAGG TATCAAGAAT TGCTGAGTAA
gTvPir_R1B  gaaatagtaa cacaaccagt cctgcagGAT TGAACCTAGG TATCAAGAAT TGCTGAGTAA
gTvPir_r7A  gaaatagtaa cacaaccagt cctgcagGAT TGAACCTAGG TATCAAGAAT TGCTGAGTAA
gTvPir_r1B  gaaatagtaa cacaaccagt cctgcagGAT TGAACCTAGG TATCAAGAAT TGCTGAGTAA
gTvPir_R6B  gaaatagtaa cacaaccagt cctgcagGAT TGAACCTAGG TATCAAGAAT TGCTGAGTAA
gTvPir_R4B  gaaatagtaa cacaaccagt cctgcagGAT TGAACCTAGG TATCAAGAAT TGCTGAGTAA
gTvPir_R6A  gaaatagtaa cacaaccagt cctgcagGAT TGAACCTAGG TATCAAGAAT TGCTGAGTAA
gTvPir_N2A  gaaatagtaa cacaaccagt cctgcagGAT TGAACCTAGG TATCAAGAAT TGCTGAGTAA
gTvPir_N2B  gaaatagtaa cacaaccagt cctgcagGAT TGAACCTAGG TATCAAGAAT TGCTGAGTAA
gTvPir_R2A  gaaatagtaa cacaaccagt cctgcagGAT TGAACCTAGG TATCAAGAAT TGCTGAGTAA
gTvPir_N1B  gaaatagtaa cacaaccagt cctgcagGAT TGAACCTAGG TATCAAGAAT TGCTGAGTAA
gTvPir_R4A  gaaatagtaa cacaaccagt cctgcagGAT TGAACCTAGG TATCAAGAAT TGCTGAGTAA
gTvPir_n3A  gaaatagtaa cacaaccagt cctgcagGAT TGAACCTAGG TATCAAGAAT TGCTGAGTAA
gTvPir_N1A  gaaatagtaa cacaaccagt cctgcagGAT TGAACCTAGG TATCAAGAAT TGCTGAGTAA
gTvPir_n3B  gaaatagtaa cacaaccagt cctgcagGAT TGAACCTAGG TATCAAGAAT TGCTGAGTAA
gTvPir_N3B  gaaatagtaa cacaaccagt cctgcagGAT TGAACCTAGG TATCAAGAAT TGCTGAGTAA
gTvPir_r4B  gaaatagtaa cacaaccagt cctgcagGAT TGAACCTAGG TATCAAGAAT TGCTGAGTAA
gTvPir_r6A  gaaatagtaa cacaaccagt cctgcagGAT TGAACCTAGG TATCAAGAAT TGCTGAGTAA
gTvPir_R3A  gaaatagtaa cacaaccagt cctgcagGAT TGAACCTAGG TATCAAGAAT TGCTGAGTAA
gTvPir_N3A  gaaatagtaa cacaaccagt cctgcagGAT TGAACCTAGG TATCAAGAAT TGCTGAGTAA
gTvPir_r6B  gaaatagtaa cacaaccagt cctgcagGAT TGAACCTAGG TATCAAGAAT TGCTGAGTAA
gTvPir_R5A  gaaatagtaa cacaaccagt cctgcagGAT TGAACCTAGG TATCAAGAAT TGCTGAGTAA
gTvPir_r7B  gaaatagtaa cacaaccagt cctgcagGAT TGAACCTAGG TATCAAGAAT TGCTGAGTAA
gTvPir_R2B  gaaatagtaa cacaaccagt cctgcagGAT TGAACCTAGG TATCAAGAAT TGCTGAGTAA
gTvPir_R3B  gaaatagtaa cacaaccagt cctgcagGAT TGAACCTAGG TATCAAGAAT TGCTGAGTAA
gTvPir_r4A  gaaatagtaa cacaaccagt cctgcagGAT TGAACCTAGG TATCAAGAAT TGCTGAGTAA
gTvPir_R5B  gaaatagtaa cacaaccagt cctgcagGAT TGAACCTAGG TATCAAGAAT TGCTGAGTAA
gTvPir_n2B  gaaatagtaa cacaaccagt cctgcagGAT TGAACCTAGG TATCAAGAAT TGCTGAGTAA
```

```
            1561                                                       1620
gTvPir_r1A  TGACATCCCC AAAGCACAAA AAGATGGGGT TGAAGTAAAA GTGATCGCAG GAGAAGCAAT
gTvPir_r2B  TGACATCCCC AAAGCACAAA AAGATGGGGT TGAAGTAAAA GTGATCGCAG GAGAAGCAAT
gTvPir_r3B  TGACATCCCC AAAGCACAAA AAGATGGGGT TGAAGTAAAA GTGATCGCAG GAGAAGCAAT
gTvPir_r5A  TGACATCCCC AAAGCACAAA AAGATGGGGT TGAAGTAAAA GTGATCGCAG GAGAAGCAAT
gTvPir_r8A  TGACATCCCC AAAGCACAAA AAGATGGGGT TGAAGTAAAA GTGATCGCAG GAGAAGCAAT
gTvPir_r8B  TGACATCCCC AAAGCACAAA AAGATGGGGT TGAAGTAAAA GTGATCGCAG GAGAAGCAAT
gTvPir_R1A  TGACATCCCC AAAGCACAAA AAGATGGGGT TGAAGTAAAA GTGATCGCAG GAGAAGCAAT
gTvPir_n1A  TGACATCCCC AAAGCACAAA AAGATGGGGT TGAAGTAAAA GTGATCGCAG GAGAAGCAAT
gTvPir_n2A  TGACATCCCC AAAGCACAAA AAGATGGGGT TGAAGTAAAA GTGATCGCAG GAGAAGCAAT
gTvPir_n1B  TGACATCCCC AAAGCACAAA AAGATGGGGT TGAAGTAAAA GTGATCGCAG GAGAAGCAAT
gTvPir_r2A  TGACATCCCC AAAGCACAAA AAGATGGGGT TGAAGTAAAA GTGATCGCAG GAGAAGCAAT
gTvPir_r3A  TGACATCCCC AAAGCACAAA AAGATGGGGT TGAAGTAAAA GTGATCGCAG GAGAAGCAAT
gTvPir_r5B  TGACATCCCC AAAGCACAAA AAGATGGGGT TGAAGTAAAA GTGATCGCAG GAGAAGCAAT
gTvPir_R1B  TGACATCCCC AAAGCACAAA AAGATGGGGT TGAAGTAAAA GTGATCGCAG GAGAAGCAAT
gTvPir_r7A  TGACATCCCC AAAGCACAAA AAGATGGGGT TGAAGTAAAA GTGATCGCAG GAGAAGCAAT
gTvPir_r1B  TGACATCCCC AAAGCACAAA AAGATGGGGT TGAAGTAAAA GTGATCGCAG GAGAAGCAAT
gTvPir_R6B  TGACATCCCC AAAGCACAAA AAGATGGGGT TGAAGTAAAA GTGATCGCAG GAGAAGCAAT
gTvPir_R4B  TGACATCCCC AAAGCACAAA AAGATGGGGT TGAAGTAAAA GTGATCGCAG GAGAAGCAAT
gTvPir_R6A  TGACATCCCC AAAGCACAAA AAGATGGGGT TGAAGTAAAA GTGATCGCAG GAGAAGCAAT
gTvPir_N2A  TGACATCCCC AAAGCACAAA AAGATGGGGT TGAAGTAAAA GTGATCGCAG GAGAAGCAAT
gTvPir_N2B  TGACATCCCC AAAGCACAAA AAGATGGGGT TGAAGTAAAA GTGATCGCAG GAGAAGCAAT
gTvPir_R2A  TGACATCCCC AAAGCACAAA AAGATGGGGT TGAAGTAAAA GTGATCGCAG GAGAAGCAAT
gTvPir_N1B  TGACATCCCC AAAGCACAAA AAGATGGGGT TGAAGTAAAA GTGATCGCAG GAGAAGCAAT
gTvPir_R4A  TGACATCCCC AAAGCACAAA AAGATGGGGT TGAAGTAAAA GTGATCGCAG GAGAAGCAAT
gTvPir_n3A  TGACATCCCC AAAGCACAAA AAGATGGGGT TGAAGTAAAA GTGATCGCAG GAGAAGCAAT
gTvPir_N1A  TGACATCCCC AAAGCACAAA AAGATGGGGT TGAAGTAAAA GTGATCGCAG GAGAAGCAAT
gTvPir_n3B  TGACATCCCC AAAGCACAAA AAGATGGGGT TGAAGTAAAA GTGATCGCAG GAGAAGCAAT
gTvPir_N3B  TGACATCCCC AAAGCACAAA AAGATGGGGT TGAAGTAAAA GTGATCGCAG GAGAAGCAAT
gTvPir_r4B  TGACATCCCC AAAGCACAAA AAGATGGGGT TGAAGTAAAA GTGATCGCAG GAGAAGCAAT
gTvPir_r6A  TGACATCCCC AAAGCACAAA AAGATGGGGT TGAAGTAAAA GTGATCGCAG GAGAAGCAAT
gTvPir_R3A  TGACATCCCC AAAGCACAAA AAGATGGGGT TGAAGTAAAA GTGATCGCAG GAGAAGCAAT
gTvPir_N3A  TGACATCCCC AAAGCACAAA AAGATGGGGT TGAAGTAAAA GTGATCGCAG GAGAAGCAAT
gTvPir_r6B  TGACATCCCC AAAGCACAAA AAGATGGGGT TGAAGTAAAA GTGATCGCAG GAGAAGCAAT
gTvPir_R5A  TGACATCCCC AAAGCACAAA AAGATGGGGT TGAAGTAAAA GTGATCGCAG GAGAAGCAAT
gTvPir_r7B  TGACATCCCC AAAGCACAAA AAGATGGGGT TGAAGTAAAA GTGATCGCAG GAGAAGCAAT
gTvPir_R2B  TGACATCCCC AAAGCACAAA AAGATGGGGT TGAAGTAAAA GTGATCGCAG GAGAAGCAAT
gTvPir_R3B  TGACATCCCC AAAGCACAAA AAGATGGGGT TGAAGTAAAA GTGATCGCAG GAGAAGCAAT
gTvPir_r4A  TGACATCCCC AAAGCACAAA AAGATGGGGT TGAAGTAAAA GTGATCGCAG GAGAAGCAAT
gTvPir_R5B  TGACATCCCC AAAGCACAAA AAGATGGGGT TGAAGTAAAA GTGATCGCAG GAGAAGCAAT
gTvPir_n2B  TGACATCCCC AAAGCACAAA AAGATGGGGT TGAAGTAAAA GTGATCGCAG GAGAAGCAAT
```

```
           1621                                                          1680
gTvPir_r1A  GGGTGTTCAC TCACAAGTTT TCACACGAAC CCCAACAATG TACGTCGATT TCACTCTAAA
gTvPir_r2B  GGGTGTTCAC TCACAAGTTT TCACACGAAC CCCAACAATG TACGTCGATT TCACTCTAAA
gTvPir_r3B  GGGTGTTCAC TCACAAGTTT TCACACGAAC CCCAACAATG TACGTCGATT TCACTCTAAA
gTvPir_r5A  GGGTGTTCAC TCACAAGTTT TCACACGAAC CCCAACAATG TACGTCGATT TCACTCTAAA
gTvPir_r8A  GGGTGTTCAC TCACAAGTTT TCACACGAAC CCCAACAATG TACGTCGATT TCACTCTAAA
gTvPir_r8B  GGGTGTTCAC TCACAAGTTT TCACACGAAC CCCAACAATG TACGTCGATT TCACTCTAAA
gTvPir_R1A  GGGTGTTCAC TCACAAGTTT TCACACGAAC CCCAACAATG TACGTCGATT TCACTCTAAA
gTvPir_n1A  GGGTGTTCAC TCACAAGTTT TCACACGAAC CCCAACAATG TACGTCGATT TCACTCTAAA
gTvPir_n2A  GGGTGTTCAC TCACAAGTTT TCACACGAAC CCCAACAATG TACGTCGATT TCACTCTAAA
gTvPir_n1B  GGGTGTTCAC TCACAAGTTT TCACACGAAC CCCAACAATG TACGTCGATT TCACTCTAAA
gTvPir_r2A  GGGTGTTCAC TCACAAGTTT TCACACGAAC CCCAACAATG TACGTCGATT TCACTCTAAA
gTvPir_r3A  GGGTGTTCAC TCACAAGTTT TCACACGAAC CCCAACAATG TACGTCGATT TCACTCTAAA
gTvPir_r5B  GGGTGTTCAC TCACAAGTTT TCACACGAAC CCCAACAATG TACGTCGATT TCACTCTAAA
gTvPir_R1B  GGGTGTTCAC TCACAAGTTT TCACACGAAC CCCAACAATG TACGTCGATT TCACTCTAAA
gTvPir_r7A  GGGTGTTCAC TCACAAGTTT ACACACGAAC CCCAACAATG TACGTCGATT TCACTCTAAA
gTvPir_r1B  GGGTGTTCAC TCACAAGTTT TCACACGAAC CCCAACAATG TACGTCGATT TCACTCTAAA
gTvPir_R6B  GGGTGTTCAC TCACAAGTTT TCACACGAAC CCCAACAATG TACGTCGATT TCACTCTAAA
gTvPir_R4B  GTGTGTTCAC TCACAAGTTT TCACACGAAC CCCAACAATG TACGTCGATT TCACTCTAAA
gTvPir_R6A  GTGTGTTCAC TCACAAGTTT TCACACGAAC CCCAACAATG TACGTCGATT TCACTCTAAA
gTvPir_N2A  GTGTGTTCAC TCACAAGTTT TCACACGAAC CCCAACAATG TACGTCGATT TCACTCTAAA
gTvPir_N2B  GTGTGTTCAC TCACAAGTTT TCACACGAAC CCCAACAATG TACGTCGATT TCACTCTAAA
gTvPir_R2A  GGGTGTTCAC TCACAAGTTT TCACACGAAC CCCAACAATG TACGTCGATT TCACTCTAAA
gTvPir_N1B  GGGTGTTCAC TCACAAGTTT TCACACGAAC CCCAACAATG TACGTCGATT TCACTCTAAA
gTvPir_R4A  GGGTGTTCAC TCACAAGTTT TCACACGAAC CCCAACAATG TACGTCGATT TCACTCTAAA
gTvPir_n3A  GGGTGTTCAC TCACAAGTTT TCACACGAAC CCCAACAATG TACGTCGATT TCACTCTAAA
gTvPir_N1A  GGGTGTTCAC TCACAAGTTT TCACACGAAC CCCAACAATG TACGTCGATT TCACTCTAAA
gTvPir_n3B  GGGTGTTCAC TCACAAGTTT TCACACGAAC CCCAACAATG TACGTCGATT TCACTCTAAA
gTvPir_N3B  GGGTGTTCAC TCACAAGTTT TCACACGAAC CCCAACAATG TACGTCGATT TCACTCTAAA
gTvPir_r4B  GGGTGTTCAC TCACAAGTTT TCACACGAAC CCCAACAATG TACGTCGATT TCACTCTAAA
gTvPir_r6A  GGGTGTTCAC TCACAAGTTT TCACACGAAC CCCAACAATG TACGTCGATT TCACTCTAAA
gTvPir_R3A  GGGTGTTCAC TCACAAGTTT TCACACGAAC CCCAACAATG TACGTCGATT TCACTCTAAA
gTvPir_N3A  GGGTGTTCAC TCACAAGTTT TCACACGAAC CCCAACAATG TACGTCGATT TCACTCTAAA
gTvPir_r6B  GGGTGTTCAC TCACAAGTTT TCACACGAAC CCCAACAATG TACGTCGATT TCACTCTAAA
gTvPir_R5A  GGGTGTTCAC TCACAAGTTT TCACACGAAC CCCAACAATG TACGTCGATT TCACTCTAAA
gTvPir_r7B  GGGTGTTCAC TCACAAGTTT TCACACGAAC CCCAACAATG TACGTCGATT TCACTCTAAA
gTvPir_R2B  GGGTGTTCAC TCACAAGTTT TCACACGAAC CCCAACAATG TACGTCGATT TCACTCTAAA
gTvPir_R3B  GGGTGTTCAC TCACAAGTTT TCACACGAAC CCCAACAATG TACGTCGATT TCACTCTAAA
gTvPir_r4A  GGGTGTTCAC TCACAAGTTT TCACACGAAC CCCAACAATG TACGTCGATT TCACTCTAAA
gTvPir_R5B  GGGTGTTCAC TCACAAGTTT TCACACGAAC CCCAACAATG TACGTCGATT TCACTCTAAA
gTvPir_n2B  GGGTGTTCAC TCACAAGTTT TCACACGAAC CCCAACAATG TACGTCGATT TCACTCTAAA
```

```
            1681                                                        1740
gTvPir_r1A  ACCAAATTCC GAGTATCATC AAGGCATTCC GGATTCTTGG AATGCGTTTG TGTACATTAT
gTvPir_r2B  ACCAAATTCC GAGTATCATC AAGGCATTCC GGATTCTTGG AATGCGTTTG TGTACATTAT
gTvPir_r3B  ACCAAATTCC GAGTATCATC AAGGCATTCC GGATTCTTGG AATGCGTTTG TGTACATTAT
gTvPir_r5A  ACCAAATTCC GAGTATCATC AAGGCATTCC GGATTCTTGG AATGCGTTTG TGTACATTAT
gTvPir_r8A  ACCAAATTCC GAGTATCATC AAGGCATTCC GGATTCTTGG AATGCGTTTG TGTACATTAT
gTvPir_r8B  ACCAAATTCC GAGTATCATC AAGGCATTCC GGATTCTTGG AATGCGTTTG TGTACATTAT
gTvPir_R1A  ACCAAATTCC GAGTATCATC AAGGCATTCC GGATTCTTGG AATGCGTTTG TGTACATTAT
gTvPir_n1A  ACCAAATTCC GAGTATCATC AAGGCATTCC GGATTCTTGG AATGCGTTTG TGTACATTAT
gTvPir_n2A  ACCAAATTCC GAGTATCATC AAGGCATTCC GGATTCTTGG AATGCGTTTG TGTACATTAT
gTvPir_n1B  ACCAAATTCC GAGTATCATC AAGGCATTCC GGATTCTTGG AATGCGTTTG TGTACATTAT
gTvPir_r2A  ACCAAATTCC GAGTATCATC AAGGCATTCC GGATTCTTGG AATGCGTTTG TGTACATTAT
gTvPir_r3A  ACCAAATTCC GAGTATCATC AAGGCATTCC GGATTCTTGG AATGCGTTTG TGTACATTAT
gTvPir_r5B  ACCAAATTCC GAGTATCATC AAGGCATTCC GGATTCTTGG AATGCGTTTG TGTACATTAT
gTvPir_R1B  ACCAAATTCC GAGTATCATC AAGGCATTCC GGATTCTTGG AATGCGTTTG TGTACATTAT
gTvPir_r7A  ACCAAATTCC GAGTATCATC AAGGCATTCC GGATTCTTGG AATGCGTTTG TGTACATTAT
gTvPir_r1B  ACCAAATTCC GAGTATCATC AAGGCATTCC GGATTCTTGG AATGCGTTTG TGTACATTAT
gTvPir_R6B  ACCAAATTCC GAGTATCATC AAGGCATTCC GGATTCTTGG AATGCGTTTG TGTACATTAT
gTvPir_R4B  ACCAAATTCC GAGTATCATC AAGGCATTCC GGATTCTTGG AATGCGTTTG TGTACATTAT
gTvPir_R6A  ACCAAATTCC GAGTATCATC AAGGCATTCC GGATTCTTGG AATGCGTTTG TGTACATTAT
gTvPir_N2A  ACCAAATTCC GAGTATCATC AAGGCATTCC GGATTCTTGG AATGCGTTTG TGTACATTAT
gTvPir_N2B  ACCAAATTCC GAGTATCATC AAGGCATTCC GGATTCTTGG AATGCGTTTG TGTACATTAT
gTvPir_R2A  ACCAAATTCC GAGTATCATC AAGGCATTCC GGATTCTTGG AATGCGTTTG TGTACATTAT
gTvPir_N1B  ACCAAATTCC GAGTATCATC AAGGCATTCC GGATTCTTGG AATGCGTTTG TGTACATTAT
gTvPir_R4A  ACCAAATTCC GAGTATCATC AAGGCATTCC GGATTCTTGG AATGCGTTTG TGTACATTAT
gTvPir_n3A  ACCAAATTCC GAGTATCATC AAGGCATTCC GGATTCTTGG AATGCGTTTG TGTACATTAT
gTvPir_N1A  ACCAAATTCC GAGTATCATC AAGGCATTCC GGATTCTTGG AATGCGTTTG TGTACATTAT
gTvPir_n3B  ACCAAATTCC GAGTATCATC AAGGCATTCC GGATTCTTGG AATGCGTTTG TGTACATTAT
gTvPir_N3B  ACCAAATTCC GAGTATCATC AAGGCATTCC GGATTCTTGG AATGCGTTTG TGTACATTAT
gTvPir_r4B  ACCAAATTCC GAGTATCATC AAGGCATTCC GGATTCTTGG AATGCGTTTG TGTACATTAT
gTvPir_r6A  ACCAAATTCC GAGTATCATC AAGGCATTCC GGATTCTTGG AATGCGTTTG TGTACATTAT
gTvPir_R3A  ACCAAATTCC GAGTATCATC AAGGCATTCC GGATTCTTGG AATGCGTTTG TGTACATTAT
gTvPir_N3A  ACCAAATTCC GAGTATCATC AAGGCATTCC GGATTCTTGG AATGCGTTTG TGTACATTAT
gTvPir_r6B  ACCAAATTCC GAGTATCATC AAGGCATTCC GGATTCTTGG AATGCGTTTG TGTACATTAT
gTvPir_R5A  ACCAAATTCC GAGTATCATC AAGGCATTCC GGATTCTTGG AATGCGTTTG TGTACATTAT
gTvPir_r7B  ACCAAATTCC GAGTATCATC AAGGCATTCC GGATTCTTGG AATGCGTTTG TGTACATTAT
gTvPir_R2B  ACCAAATTCC GAGTATCATC AAGGCATTCC GGATTCTTGG AATGCGTTTG TGTACATTAT
gTvPir_R3B  ACCAAATTCC GAGTATCATC AAGGCATTCC GGATTCTTGG AATGCGTTTG TGTACATTAT
gTvPir_r4A  ACCAAATTCC GAGTATCATC AAGGCATTCC GGATTCTTGG AATGCGTTTG TGTACATTAT
gTvPir_R5B  ACCAAATTCC GAGTATCATC AAGGCATTCC GGATTCTTGG AATGCGTTTG TGTACATTAT
gTvPir_n2B  ACCAAATTCC GAGTATCATC AAGGCATTCC GGATTCTTGG AATGCGTTTG TGTACATTAT
```

```
            1741                                                        1800
gTvPir_r1A  TGAAGGTGAA GGTGTATTTG GCATCCCGAA TTCGAAGCCT GCAGAAGCTC ATCATATCTT
gTvPir_r2B  TGAAGGTGAA GGTGTATTTG GCATCCCGAA TTCGAAGCCT GCAGAAGCTC ATCATATCTT
gTvPir_r3B  TGAAGGTGAA GGTGTATTTG GCATCCCGAA TTCGAAGCCT GCAGAAGCTC ATCATATCTT
gTvPir_r5A  TGAAGGTGAA GGTGTATTTG GCATCCCGAA TTCGAAGCCT GCAGAAGCTC ATCATATCTT
gTvPir_r8A  TGAAGGTGAA GGTGTATTTG GCATCCCGAA TTCGAAGCCT GCAGAAGCTC ATCATATCTT
gTvPir_r8B  TGAAGGTGAA GGTGTATTTG GCATCCCGAA TTCGAAGCCT GCAGAAGCTC ATCATATCTT
gTvPir_R1A  TGAAGGTGAA GGTGTATTTG GCATCCCGAA TTCGAAGCCT GCAGAAGCTC ATCATATCTT
gTvPir_n1A  TGAAGGTGAA GGTGTATTTG GCATCCCGAA TTCGAAGCCT GCAGAAGCTC ATCATATCTT
gTvPir_n2A  TGAAGGTGAA GGTGTATTTG GCATCCCGAA TTCGAAGCCT GCAGAAGCTC ATCATATCTT
gTvPir_n1B  TGAAGGTGAA GGTGTATTTG GCATCCCGAA TTCGAAGCCT GCAGAAGCTC ATCATATCTT
gTvPir_r2A  TGAAGGTGAA GGTGTATTTG GCATCCCGAA TTCGAAGCCT GCAGAAGCTC ATCATATCTT
gTvPir_r3A  TGAAGGTGAA GGTGTATTTG GCATCCCGAA TTCGAAGCCT GCAGAAGCTC ATCATATCTT
gTvPir_r5B  TGAAGGTGAA GGTGTATTTG GCATCCCGAA TTCGAAGCCT GCAGAAGCTC ATCATATCTT
gTvPir_R1B  TGAAGGTGAA GGTGTATTTG GCATCCCGAA TTCGAAGCCT GCAGAAGCTC ATCATATCTT
gTvPir_r7A  TGAAGGTGAA GGTGTATTTG GCATCCCGAA TTCGAAGCCT GCAGAAGCTC ATCATATCTT
gTvPir_r1B  TGAAGGTGAA GGTGTATTTG GCATCCCGAA TTCGAAGCCT GCAGAAGCTC ATCATATCTT
gTvPir_R6B  TGAAGGTGAA GGTGTATTTG GCATCCCGAA TTCGAAGCCT GCAGAAGCTC ATCATATCTT
gTvPir_R4B  TGAAGGTGAA GGTGTATTTG GCATCCCGAA TTCGAAGCCT GCAGAAGCTC ATCATATCTT
gTvPir_R6A  TGAAGGTGAA GGTGTATTTG GCATCCCGAA TTCGAAGCCT GCAGAAGCTC ATCATATCTT
gTvPir_N2A  TGAAGGTGAA GGTGTATTTG GCATCCCGAA TTCGAAGCCT GCAGAAGCTC ATCATATCTT
gTvPir_N2B  TGAAGGTGAA GGTGTATTTG GCATCCCGAA TTCGAAGCCT GCAGAAGCTC ATCATATCTT
gTvPir_R2A  TGAAGGTGAA GGTGTATTTG GCATCCCGAA TTCGAAGCCT GCAGAAGCTC ATCATATCTT
gTvPir_N1B  TGAAGGTGAA GGTGTATTTG GCATCCCGAA TTCGAAGCCT GCAGAAGCTC ATCATATCTT
gTvPir_R4A  TGAAGGTGAA GGTGTATTTG GCATCCCGAA TTCGAAGCCT GCAGAAGCTC ATCATATCTT
gTvPir_n3A  TGAAGGTGAA GGTGTATTTG GCATCCCGAA TTCGAAGCCT GCAGAAGCTC ATCATATCTT
gTvPir_N1A  TGAAGGTGAA GGTGTATTTG GCATCCCGAA TTCGAAGCCT GCAGAAGCTC ATCATATCTT
gTvPir_n3B  TGAAGGTGAA GGTGTATTTG GCATCCCGAA TTCGAAGCCT GCAGAAGCTC ATCATATCTT
gTvPir_N3B  TGAAGGTGAA GGTGTATTTG GCATCCCGAA TTCGAAGCCT GCAGAAGCTC ATCATATCTT
gTvPir_r4B  TGAAGGTGAA GGTGTATTTG GCATCCCGAA TTCGAAGCCT GCAGAAGCTC ATCATATCTT
gTvPir_r6A  TGAAGGTGAA GGTGTATTTG GCATCCCGAA TTCGAAGCCT GCAGAAGCTC ATCATATCTT
gTvPir_R3A  TGAAGGTGAA GGTGTATTTG GCATCCCGAA TTCGAAGCCT GCAGAAGCTC ATCATATCTT
gTvPir_N3A  TGAAGGTGAA GGTGTATTTG GCATCCCGAA TTCGAAGCCT GCAGAAGCTC ATCATATCTT
gTvPir_r6B  TGAAGGTGAA GGTGTATTTG GCATCCCGAA TTCGAAGCCT GCAGAAGCTC ATCATATCTT
gTvPir_R5A  TGAAGGTGAA GGTGTATTTG GCATCCCGAA TTCGAAGCCT GCAGAAGCTC ATCATATCTT
gTvPir_r7B  TGAAGGTGAA GGTGTATTTG GCATCCCGAA TTCGAAGCCT GCAGAAGCTC ATCATATCTT
gTvPir_R2B  TGAAGGTGAA GGTGTATTTG GCATCCCGAA TTCGAAGCCT GCAGAAGCTC ATCATATCTT
gTvPir_R3B  TGAAGGTGAA GGTGTATTTG GCATCCCGAA TTCGAAGCCT GCAGAAGCTC ATCATATCTT
gTvPir_r4A  TGAAGGTGAA GGTGTATTTG GCATCCCGAA TTCGAAGCCT GCAGAAGCTC ATCATATCTT
gTvPir_R5B  TGAAGGTGAA GGTGTATTTG GCATCCCGAA TTCGAAGCCT GCAGAAGCTC ATCATATCTT
gTvPir_n2B  TGAAGGTGAA GGTGTATTTG GCATCCCGAA TTCGAAGCCT GCAGAAGCTC ATCATATCTT
```

```
            1801                                                       1860
gTvPir_r1A  GGTTTTGGGA CCTGGAGAGG GGCTAAGTGT GTGGAACAAG TTCACAGAAC CATTGAGGTT
gTvPir_r2B  GGTTTTGGGA CCTGGAGAGG GGCTAAGTGT GTGGAACAAG TTCACAGAAC CATTGAGGTT
gTvPir_r3B  GGTTTTGGGA CCTGGAGAGG GGCTAAGTGT GTGGAACAAG TTCACAGAAC CATTGAGGTT
gTvPir_r5A  GGTTTTGGGA CCTGGAGAGG GGCTAAGTGT GTGGAACAAG TTCACAGAAC CATTGAGGTT
gTvPir_r8A  GGTTTTGGGA CCTGGAGAGG GGCTAAGTGT GTGGAACAAG TTCACAGAAC CATTGAGGTT
gTvPir_r8B  GGTTTTGGGA CCTGGAGAGG GGCTAAGTGT GTGGAACAAG TTCACAGAAC CATTGAGGTT
gTvPir_R1A  GGTTTTGGGA CCTGGAGAGG GGCTAAGTGT GTGGAACAAG TTCACAGAAC CATTGAGGTT
gTvPir_n1A  GGTTTTGGGA CCTGGAGAGG GGCTAAGTGT GTGGAACAAG TTCACAGAAC CATTGAGGTT
gTvPir_n2A  GGTTTTGGGA CCTGGAGAGG GGCTAAGTGT GTGGAACAAG TTCACAGAAC CATTGAGGTT
gTvPir_n1B  GGTTTTGGGA CCTGGAGAGG GGCTAAGTGT GTGGAACAAG TTCACAGAAC CATTGAGGTT
gTvPir_r2A  GGTTTTGGGA CCTGGAGAGG GGCTAAGTGT GTGGAACAAG TTCACAGAAC CATTGAGGTT
gTvPir_r3A  GGTTTTGGGA CCTGGAGAGG GGCTAAGTGT GTGGAACAAG TTCACAGAAC CATTGAGGTT
gTvPir_r5B  GGTTTTGGGA CCTGGAGAGG GGCTAAGTGT GTGGAACAAG TTCACAGAAC CATTGAGGTT
gTvPir_R1B  GGTTTTGGGA CCTGGAGAGG GGCTAAGTGT GTGGAACAAG TTCACAGAAC CATTGAGGTT
gTvPir_r7A  GGTTTTGGGA CCTGGAGAGG GGCTAAGTGT GTGGAACAAG TTCACAGAAC CATTGAGGTT
gTvPir_r1B  GGTTTTGGGA CCTGGAGAGG GGCTAAGTGT GTGGAACAAG TTCACAGAAC CATTGAGGTT
gTvPir_R6B  GGTTTTGGGA CCTGGAGAGG GGCTAAGTGT GTGGAACAAG TTCACAGAAC CATTGAGGTT
gTvPir_R4B  GGTTTTGGGA CCTGGAGAGG GGCTAAGTGT GTGGAACAAG TTCACAGAAC CATTGAGGTT
gTvPir_R6A  GGTTTTGGGA CCTGGAGAGG GGCTAAGTGT GTGGAACAAG TTCACAGAAC CATTGAGGTT
gTvPir_N2A  GGTTTTGGGA CCTGGAGAGG GGCTAAGTGT GTGGAACAAG TTCACAGAAC CATTGAGGTT
gTvPir_N2B  GGTTTTGGGA CCTGGAGAGG GGCTAAGTGT GTGGAACAAG TTCACAGAAC CATTGAGGTT
gTvPir_R2A  GGTTTTGGGA CCTGGAGAGG GGCTAAGTGT GTGGAACAAG TTCACAGAAC CATTGAGGTT
gTvPir_N1B  GGTTTTGGGA CCTGGAGAGG GGCTAAGTGT GTGGAACAAG TTCACAGAAC CATTGAGGTT
gTvPir_R4A  GGTTTTGGGA CCTGGAGAGG GGCTAAGTGT GTGGAACAAG TTCACAGAAC CATTGAGGTT
gTvPir_n3A  GGTTTTGGGA CCTGGAGAGG GGCTAAGTGT GTGGAACAAG TTCACAGAAC CATTGAGGTT
gTvPir_N1A  GGTTTTGGGA CCTGGAGAGG GGCTAAGTGT GTGGAACAAG TTCACAGAAC CATTGAGGTT
gTvPir_n3B  GGTTTTGGGA CCTGGAGAGG GGCTAAGTGT GTGGAACAAG TTCACAGAAC CATTGAGGTT
gTvPir_N3B  GGTTTTGGGA CCTGGAGAGG GGCTAAGTGT GTGGAACAAG TTCACAGAAC CATTGAGGTT
gTvPir_r4B  GGTTTTGGGA CCTGGAGAGG GGCTAAGTGT GTGGAACAAG TTCACAGAAC CATTGAGGTT
gTvPir_r6A  GGTTTTGGGA CCTGGAGAGG GGCTAAGTGT GTGGAACAAG TTCACAGAAC CATTGAGGTT
gTvPir_R3A  GGTTTTGGGA CCTGGAGAGG GGCTAAGTGT GTGGAACAAG TTCACAGAAC CATTGAGGTT
gTvPir_N3A  GGTTTTGGGA CCTGGAGAGG GGCTAAGTGT GTGGAACAAG TTCACAGAAC CATTGAGGTT
gTvPir_r6B  GGTTTTGGGA CCTGGAGAGG GGCTAAGTGT GTGGAACAAG TTCACAGAAC CATTGAGGTT
gTvPir_R5A  GGTTTTGGGA CCTGGAGAGG GGCTAAGTGT GTGGAACAAG TTCACAGAAC CATTGAGGTT
gTvPir_r7B  GGTTTTGGGA CCTGGAGAGG GGCTAAGTGT GTGGAACAAG TTCACAGAAC CATTGAGGTT
gTvPir_R2B  GGTTTTGGGA CCTGGAGAGG GGCTAAGTGT GTGGAACAAG TTCACAGAAC CATTGAGGTT
gTvPir_R3B  GGTTTTGGGA CCTGGAGAGG GGCTAAGTGT GTGGAACAAG TTCACAGAAC CATTGAGGTT
gTvPir_r4A  GGTTTTGGGA CCTGGAGAGG GGCTAAGTGT GTGGAACAAG TTCACAGAAC CATTGAGGTT
gTvPir_R5B  GGTTTTGGGA CCTGGAGAGG GGCTAAGTGT GTGGAACAAG TTCACAGAAC CATTGAGGTT
gTvPir_n2B  GGTTTTGGGA CCTGGAGAGG GGCTAAGTGT GTGGAACAAG TTCACAGAAC CATTGAGGTT
```

```
             1861                                                             1920
gTvPir_r1A   TATTTTGGTA GGCGGGCAGC CACTGAATGA GCCTGTGGTT CAGCACGGTC CGTTTGTGAT
gTvPir_r2B   TATTTTGGTA GGCGGGCAGC CACTGAATGA GCCTGTGGTT CAGCACGGTC CGTTTGTGAT
gTvPir_r3B   TATTTTGGTA GGCGGGCAGC CACTGAATGA GCCTGTGGTT CAGCACGGTC CGTTTGTGAT
gTvPir_r5A   TATTTTGGTA GGCGGGCAGC CACTGAATGA GCCTGTGGTT CAGCACGGTC CGTTTGTGAT
gTvPir_r8A   TATTTTGGTA GGCGGGCAGC CACTGAATGA GCCTGTGGTT CAGCACGGTC CGTTTGTGAT
gTvPir_r8B   TATTTTGGTA GGCGGGCAGC CACTGAATGA GCCTGTGGTT CAGCACGGTC CGTTTGTGAT
gTvPir_R1A   TATTTTGGTA GGCGGGCAGC CACTGAATGA GCCTGTGGTT CAGCACGGTC CGTTTGTGAT
gTvPir_n1A   TATTTTGGTA GGCGGGCAGC CACTGAATGA GCCTGTGGTT CAGCACGGTC CGTTTGTGAT
gTvPir_n2A   TATTTTGGTA GGCGGGCAGC CACTGAATGA GCCTGTGGTT CAGCACGGTC CGTTTGTGAT
gTvPir_n1B   TATTTTGGTA GGCGGGCAGC CACTGAATGA GCCTGTGGTT CAGCACGGTC CGTTTGTGAT
gTvPir_r2A   TATTTTGGTA GGCGGGCAGC CACTGAATGA GCCTGTGGTT CAGCACGGTC CGTTTGTGAT
gTvPir_r3A   TATTTTGGTA GGCGGGCAGC CACTGAATGA GCCTGTGGTT CAGCACGGTC CGTTTGTGAT
gTvPir_r5B   TATTTTGGTA GGCGGGCAGC CACTGAATGA GCCTGTGGTT CAGCACGGTC CGTTTGTGAT
gTvPir_R1B   TATTTTGGTA GGCGGGCAGC CACTGAATGA GCCTGTGGTT CAGCACGGTC CGTTTGTGAT
gTvPir_r7A   TATTTTGGTA GGCGGGCAGC CACTGAATGA GCCTGTGGTT CAGCACGGTC CGTTTGTGAT
gTvPir_r1B   TATTTTGGTA GGCGGGCAGC CACTTAATGA GCCTGTGGTT CAGCACGGTC CGTTTGTGAT
gTvPir_R6B   TATTTTGGTA GGCGGGCAGC CACTTAATGA GCCTGTGGTT CAGCACGGTC CGTTTGTGAT
gTvPir_R4B   TATTTTGGTA GGCGGGCAGC CACTGAATGA GCCTGTGGTT CAGCACGGTC CGTTTGTGAT
gTvPir_R6A   TATTTTGGTA GGCGGGCAGC CACTGAATGA GCCTGTGGTT CAGCACGGTC CGTTTGTGAT
gTvPir_N2A   TATTTTGGTA GGCGGGCAGC CACTGAATGA GCCTGTGGTT CAGCACGGTC CGTTTGTGAT
gTvPir_N2B   TATTTTGGTA GGCGGGCAGC CACTGAATGA GCCTGTGGTT CAGCACGGTC CGTTTGTGAT
gTvPir_R2A   TATTTTGGTA GGCGGGCAGC CACTGAATGA GCCTGTGGTT CAGCACGGTC CGTTTGTGAT
gTvPir_N1B   TATTTTGGTA GGCGGGCAGC CACTGAATGA GCCTGTGGTT CAGCACGGTC CGTTTGTGAT
gTvPir_R4A   TATTTTGGTA GGCGGGCAGC CACTGAATGA GCCTGTGGTT CAGCACGGTC CGTTTGTGAT
gTvPir_n3A   TATTTTGGTA GGCGGGCAGC CACTGAATGA GCCTGTGGTT CAGCACGGTC CGTTTGTGAT
gTvPir_N1A   TATTTTGGTA GGCGGGCAGC CACTGAATGA GCCTGTGGTT CAGCACGGTC CGTTTGTGAT
gTvPir_n3B   TATTTTGGTA GGCGGGCAGC CACTTAATGA GCCTGTGGTT CAGCACGGTC CGTTTGTGAT
gTvPir_N3B   TATTTTGGTA GGCGGGCAGC CACTTAATGA GCCTGTGGTT CAGCACGGTC CGTTTGTGAT
gTvPir_r4B   TATTTTGGTA GGCGGGCAGC CACTTAATGA GCCTGTGGTT CAGCACGGTC CGTTTGTGAT
gTvPir_r6A   TATTTTGGTA GGCGGGCAGC CACTTAATGA GCCTGTGGTT CAGCACGGTC CGTTTGTGAT
gTvPir_R3A   TATTTTGGTA GGCGGGCAGC CACTTAATGA GCCTGTGGTT CAGCACGGTC CGTTTGTGAT
gTvPir_N3A   TATTTTGGTA GGCGGGCAGC CACTTAATGA GCCTGTGGTT CAGCACGGTC CGTTTGTGAT
gTvPir_r6B   TATTTTGGTA GGCGGGCAGC CACTTAATGA GCCTGTGGTT CAGCACGGTC CGTTTGTGAT
gTvPir_R5A   TATTTTGGTA GGCGGGCAGC CACTTAATGA GCCTGTGGTT CAGCACGGTC CGTTTGTGAT
gTvPir_r7B   TATTTTGGTA GGCGGGCAGC CACTTAATGA GCCTGTGGTT CAGCACGGTC CGTTTGTGAT
gTvPir_R2B   TATTTTGGTA GGCGGGCAGC CACTTAATGA GCCTGTGGTT CAGCACGGTC CGTTTGTGAT
gTvPir_R3B   TATTTTGGTA GGCGGGCAGC CACGTAATGA GCCTGTGGTT CAGCACGGTC CGTTTGTGAT
gTvPir_r4A   TATTTTGGTA GGCGGGCAGC CACTGAATGA GCCTGTGGTT CAGCACGGTC CGTTTGTGAT
gTvPir_R5B   TATTTTGGTA GGCGGGCAGC CACTGAATGA GCCTGTGGTT CAGCACGGTC CGTTTGTGAT
gTvPir_n2B   TATTTTGGTA GGCGGGCAGC CACTGAATGA GCCTGTGGTT CAGCACGGTC CGTTTGTGAT
```

```
             1921                                                        1980
gTvPir_r1A  GAATACGCAC GCTGAGATTG ACAAAACAAT GGAAGACTAT TACTATGGCA AGAATGGTTT
gTvPir_r2B  GAATACGCAC GCTGAGATTG ACAAAACAAT GGAAGACTAT TACTATGGCA AGAATGGTTT
gTvPir_r3B  GAATACGCAC GCTGAGATTG ACAAAACAAT GGAAGACTAT TACTATGGCA AGAATGGTTT
gTvPir_r5A  GAATACGCAC GCTGAGATTG ACAAAACAAT GGAAGACTAT TACTATGGCA AGAATGGTTT
gTvPir_r8A  GAATACGCAC GCTGAGATTG ACAAAACAAT GGAAGACTAT TACTATGGCA AGAATGGTTT
gTvPir_r8B  GAATACGCAC GCTGAGATTG ACAAAACAAT GGAAGACTAT TACTATGGCA AGAATGGTTT
gTvPir_R1A  GAATACGCAC GCTGAGATTG ACAAAACAAT GGAAGACTAT TACTATGGCA AGAATGGTTT
gTvPir_n1A  GAATACGCAC GCTGAGATTG ACAAAACAAT GGAAGACTAT TACTATGGCA AGAATGGTTT
gTvPir_n2A  GAATACGCAC GCTGAGATTG ACAAAACAAT GGAAGACTAT TACTATGGCA AGAATGGTTT
gTvPir_n1B  GAATACGCAC GCTGAGATTG ACAAAACAAT GGAAGACTAT TACTATGGCA AGAATGGTTT
gTvPir_r2A  GAATACGCAC GCTGAGATTG ACAAAACAAT GGAAGACTAT TACTATGGCA AGAATGGTTT
gTvPir_r3A  GAATACGCAC GCTGAGATTG ACAAAACAAT GGAAGACTAT TACTATGGCA AGAATGGTTT
gTvPir_r5B  GAATACGCAC GCTGAGATTG ACAAAACAAT GGAAGACTAT TACTATGGCA AGAATGGTTT
gTvPir_R1B  GAATACGCAC GCTGAGATTG ACAAAACAAT GGAAGACTAT TACTATGGCA AGAATGGTTT
gTvPir_r7A  GAATACGCAC GCTGAGATTG ACAAAACAAT GGAAGACTAT TACTATGGCA AGAATGGTTT
gTvPir_r1B  GAATACGCAC GCTGAGATTG ACAAAACAAT GGAAGACTAT TACTATGGCA AGAATGGTTT
gTvPir_R6B  GAATACGCAC GCTGAGATTG ACAAAACAAT GGAAGACTAT TACTATGGCA AGAATGGTTT
gTvPir_R4B  GAATACGCAC GCTGAGATTG ACAAAACAAT GGAAGACTAT TACTATGGCA AGAATGGTTT
gTvPir_R6A  GAATACGCAC GCTGAGATTG ACAAAACAAT GGAAGACTAT TACTATGGCA AGAATGGTTT
gTvPir_N2A  GAATACGCAC GCTGAGATTG ACAAAACAAT GGAAGACTAT TACTATGGCA AGAATGGTTT
gTvPir_N2B  GAATACGCAC GCTGAGATTG ACAAAACAAT GGAAGACTAT TACTATGGCA AGAATGGTTT
gTvPir_R2A  GAATACGCAC GCTGAGATTG ACAAAACAAT GGAAGACTAT TACTATGGCA AGAATGGTTT
gTvPir_N1B  GAATACGCAC GCTGAGATTG ACAAAACAAT GGAAGACTAT TACTATGGCA AGAATGGTTT
gTvPir_R4A  GAATACGCAC GCTGAGATTG ACAAAACAAT GGAAGACTAT TACTATGGCA AGAATGGTTT
gTvPir_n3A  GAATACGCAC GCTGAGATTG ACAAAACAAT GGAAGACTAT TACTATGGCA AGAATGGTTT
gTvPir_N1A  GAATACGCAC GCTGAGATTG ACAAAACAAT GGAAGACTAT TACTATGGCA AGAATGGTTT
gTvPir_n3B  GAATACGCAC GCTGAGATTG ACAAAACAAT GGAAGACTAT TACTATGGCA AGAATGGTTT
gTvPir_N3B  GAATACGCAC GCTGAGATTG ACAAAACAAT GGAAGACTAT TACTATGGCA AGAATGGTTT
gTvPir_r4B  GAATACGCAC GCTGAGATTG ACAAAACAAT GGAAGACTAT TACTATGGCA AGAATGGTTT
gTvPir_r6A  GAATACGCAC GCTGAGATTG ACAAAACAAT GGAAGACTAT TACTATGGCA AGAATGGTTT
gTvPir_R3A  GAATACGCAC GCTGAGATTG ACAAAACAAT GGAAGACTAT TACTATGGCA AGAATGGTTT
gTvPir_N3A  GAATACGCAC GCTGAGATTG ACAAAACAAT GGAAGACTAT TACTATGGCA AGAATGGTTT
gTvPir_r6B  GAATACGCAC GCTGAGATTG ACAAAACAAT GGAAGACTAT TACTATGGCA AGAATGGTTT
gTvPir_R5A  GAATACGCAC GCTGAGATTG ACAAAACAAT GGAAGACTAT TACTATGGCA AGAATGGTTT
gTvPir_r7B  GAATACGCAC GCTGAGATTG ACAAAACAAT GGAAGACTAT TACTATGGCA AGAATGGTTT
gTvPir_R2B  GAATACGCAC GCTGAGATTG ACAAAACAAT GGAAGACTAT TACTATGGCA AGAATGGTTT
gTvPir_R3B  GAATACGCAC GCTGAGATTG ACAAAACAAT GGAAGACTAT TACTATGGCA AGAATGGTTT
gTvPir_r4A  GAATACGCAC GCTGAGATTG ACAAAACAAT GGAAGACTAT TACTATGGCA AGAATGGTTT
gTvPir_R5B  GAATACGCAC GCTGAGATTG ACAAAACAAT GGAAGACTAT TACTATGGCA AGAATGGTTT
gTvPir_n2B  GAATACGCAC GCTGAGATTG ACAAAACAAT GGAAGACTAT TACTATGGCA AGAATGGTTT
```

```
             1981                           2011
gTvPir_r1A  CGAGCTGGCA AAAAAATGGA AATCTAAATG A
gTvPir_r2B  CGAGCTGGCA AAAAAATGGA AATCTAAATG A
gTvPir_r3B  CGAGCTGGCA AAAAAATGGA AATCTAAATG A
gTvPir_r5A  CGAGCTGGCA AAAAAATGGA AATCTAAATG A
gTvPir_r8A  CGAGCTGGCA AAAAAATGGA AATCTAAATG A
gTvPir_r8B  CGAGCTGGCA AAAAAATGGA AATCTAAATG A
gTvPir_R1A  CGAGCTGGCA AAAAAATGGA AATCTAAATG A
gTvPir_n1A  CGAGCTGGCA AAAAAATGGA AATCTAAATG A
gTvPir_n2A  CGAGCTGGCA AAAAAATGGA AATCTAAATG A
gTvPir_n1B  CGAGCTGGCA AAAAAATGGA AATCTAAATG A
gTvPir_r2A  CGAGCTGGCA AAAAAATGGA AATCTAAATG A
gTvPir_r3A  CGAGCTGGCA AAAAAATGGA AATCTAAATG A
gTvPir_r5B  CGAGCTGGCA AAAAAATGGA AATCTAAATG A
gTvPir_R1B  CGAGCTGGCA AAAAAATGGA AATCTAAATG A
gTvPir_r7A  CGAGCTGGCA AAAAAATGGA AATCTAAATG A
gTvPir_r1B  CGAGCTGGCA AAAAAATGGA AATCTAAATG A
gTvPir_R6B  CGAGCTGGCA AAAAAATGGA AATCTAAATG A
gTvPir_R4B  CGAGCTGGCA AAAAAATGGA AATCTAAATG A
gTvPir_R6A  CGAGCTGGCA AAAAAATGGA AATCTAAATG A
gTvPir_N2A  CGAGCTGGCA AAAAAATGGA AATCTAAATG A
gTvPir_N2B  CGAGCTGGCA AAAAAATGGA AATCTAAATG A
gTvPir_R2A  CGAGCTGGCA AAAAAATGGA AATCTAAATG A
gTvPir_N1B  CGAGCTGGCA AAAAAATGGA AATCTAAATG A
gTvPir_R4A  CGAGCTGGCA AAAAAATGGA AATCTAAATG A
gTvPir_n3A  CGAGCTGGCA AAAAAATGGA AATCTAAATG A
gTvPir_N1A  CGAGCTGGCA AAAAAATGGA AATCTAAATG A
gTvPir_n3B  CGAGCTGGCA AAAAAATGGA AATCTAAATG A
gTvPir_N3B  CGAGCTGGCA AAAAAATGGA AATCTAAATG A
gTvPir_r4B  CGAGCTGGCA AAAAAATGGA AATCTAAATG A
gTvPir_r6A  CGAGCTGGCA AAAAAATGGA AATCTAAATG A
gTvPir_R3A  CGAGCTGGCA AAAAAATGGA AATCTAAATG A
gTvPir_N3A  CGAGCTGGCA AAAAAATGGA AATCTAAATG A
gTvPir_r6B  CGAGCTGGCA AAAAAATGGA AATCTAAATG A
gTvPir_R5A  CGAGCTGGCA AAAAAATGGA AATCTAAATG A
gTvPir_r7B  CGAGCTGGCA AAAAAATGGA AATCTAAATG A
gTvPir_R2B  CGAGCTGGCA AAAAAATGGA AATCTAAATG A
gTvPir_R3B  CGAGCTGGCA AAAAAATGGA AATCTAAATG A
gTvPir_r4A  CGAGCTGGCA AAAAAATGGA AATCTAAATG A
gTvPir_R5B  CGAGCTGGCA AAAAAATGGA AATCTAAATG A
gTvPir_n2B  CGAGCTGGCA AAAAAATGGA AATCTAAATG A
```
