# Supplementary material for: The differentially regulated genes TvQR1 and TvPirin of the parasitic plant Triphysaria exhibit distinctive natural allelic diversity
Source: BMC Plant Biol. 2013 Feb 18;13:28. doi: 10.1186/1471-2229-13-28 (PMC3599707; doi:10.1186/1471-2229-13-28)
Supplement: Additional file 7 — TvQR1 protein domains and amino acid diversity. [file 1471-2229-13-28-S7.doc]

**Additional file 7. TvQR1 protein domains and amino acid diversity**

A. PFAM domains in the TvQR1 protein


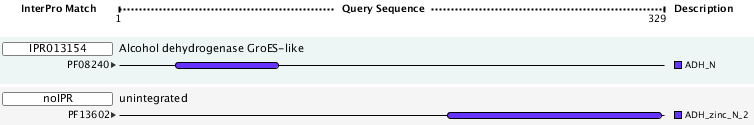


B. Multiple sequence alignment of deduced TvQR1 proteins from all full-length cDNA and genomic *TvQR1* clones

The ORFs of 9 cDNA alleles (cTvQR1_#) and 20 genomic alleles (gTvQR1_#) are aligned. Homozygotes or heterozygotes with identical aa sequences for both alleles are presented as single protein alleles. Dots represent identical residues. The ADH GrosES-like and ADH_N_2 Rossmann domains are underlined at the N and C termini, respectively.

1 60

cTvQR1_1 MAGKLMRAVQ YDGYGGGAAG LKHVEVPIPS PGKGEVLIKL EAISLNQLDW KLQNGMVRPF

cTvQR1_9 .......... .......... .......... .......... .......... ..........

cTvQR1_3 .......... .......... .......... .......... .......... ..........

cTvQR1_5 .......... .......... .......... .......... .......... ..........

cTvQR1_4 .......... .......... .......... .......... .......... ..........

gTvQR1_r2 .......... .......... .......... .......... .......... ..........

gTvQR1_R4 .......... .......... .......... .......... .......... ..........

gTvQR1_R6 .......... .......... .......... .......... .......... ..........

gTvQR1_R5 .......... .......... .......... .......... .......... ..........

gTvQR1_R1 .......... .......... .......... .......... .......... ..........

gTvQR1_r5 .......... .......... ...D...... .......... .......... ..........

gTvQR1_R2 .......... .......... ...D...... .......... .......... ..........

gTvQR1_N3 .......... .......... ...D...... .......... .......... ..........

gTvQR1_N2 .......... .......... ...D...... .......... .......... ..........

gTvQR1_n1 .......... .......... ...D...... .......... .......... ..........

gTvQR1_n2 .......... .......... ...D...... .......... .......... ..........

gTvQR1_n3 .......... .......... ...D...... .......... .......... ..........

cTvQR1_7 .......... .......... .......... .......... .......... ..........

cTvQR1_8 .......... .......... .......... .......... .......... ..........

gTvQR1_r8 .......... .......... .......... .......... .......... ..........

cTvQR1_2 .......... ....S..... .......V.. .......... .......... .I.......I

gTvQR1_r3 .......... ....S..... .....I.V.. .......... .......... .I.......I

gTvQR1_r6 .......... ....S..... .....I.V.. .......... .......... .I.......I

cTvQR1_6 .......... ....S..... .......V.. .......... .......... .I.......I

gTvQR1_r1 .......... ....S..... .......V.. .......... .......... .I.......I

gTvQR1_R3 .......... ....S..... .......V.. .......... .......... .I.......I

gTvQR1_N1 .......... ....S..... .......V.. .......... .......... .I.......I

gTvQR1_r4 .......... ....S..... .......V.. .......... ..V....... .I.......I

gTvQR1_r7 .......... ....S..... .......V.. .......... .......... .I.......I

61 120

cTvQR1_1 LPRKFPFIPA TDVAGEVVRI GPDVKNFKPG DKVVAMLGSF GGGGLAEYGV ASAKLTVHRP

cTvQR1_9 .......... .......... .......... .......... .......... ..........

cTvQR1_3 .......... .......... .......... .......... .......... ..E.......

cTvQR1_5 .......... .......... .......... .......... .......... ..E.......

cTvQR1_4 .......... .......... .......... .......... .......... ..E.......

gTvQR1_r2 .......... .......... .......E.. .......... .........I ..........

gTvQR1_R4 .......... .......... .......E.. .......... .........I ..........

gTvQR1_R6 .......... .......... .......E.. .......... .........I ..........

gTvQR1_R5 .......... .......... .......E.. .......... .........I ..........

gTvQR1_R1 .......... .......... .......E.. .......... .........I ..........

gTvQR1_r5 .......... .......... .Q........ .......... .......... ..E.......

gTvQR1_R2 .......... .......... .Q........ .......... .......... ..E.......

gTvQR1_N3 .......... .......... .Q........ .......... .......... ..E.......

gTvQR1_N2 .......... .......... .Q........ .......... .......... ..E.......

gTvQR1_n1 .......... .......... .Q........ .......... .......... ..E.......

gTvQR1_n2 .......... .......... .Q........ .......... .......... ..E.......

gTvQR1_n3 .......... .......... .Q........ .......... .......... ..E.......

cTvQR1_7 .L........ .......... .......... .......... .......... ..E.......

cTvQR1_8 .......... .......... .......... .......... .......... ..E.......

gTvQR1_r8 .......... .......... .......... .......... .......... ..E.......

cTvQR1_2 .......... .......... ..G.E..... ....V...H. R......... ..E.......

gTvQR1_r3 .......... .......... ..G.E..... ........H. R......... ..E.......

gTvQR1_r6 .......... .......... ..G.E..... ........H. R......... ..E.......

cTvQR1_6 .......... .......... ..G.E..... ........H. R......... ..E.......

gTvQR1_r1 ..Q....... .......... ..G....... ........H. R......... ..E.......

gTvQR1_R3 ..Q....... .......... ..G....... ........H. R......... ..E.......

gTvQR1_N1 .......... .......... ..G.E..... ........H. R......... ..E.......

gTvQR1_r4 .......... .......... ..G....... ........H. R......... ..E.......

gTvQR1_r7 .......... ........QV .......... ........H. R......... ..E.......

121 180

cTvQR1_1 PEVSAAESSG LPIAGLTAHM ALTQHIGLNL DKSGPHKNIL ITAASGGVGQ YAVQLAKLGN

cTvQR1_9 .......... .......... .......... .......... .......... ..........

cTvQR1_3 .......... .......... .......... .......... .......... ..........

cTvQR1_5 .......... .......... .......... .......... .......... ..........

cTvQR1_4 .......... .......... .......... .......... .......... ..........

gTvQR1_r2 .......... .......... .......... .......... .......... ..........

gTvQR1_R4 .......... .......... .......... .......... .......... ..........

gTvQR1_R6 .......... .......... .......... .......... .......... ..........

gTvQR1_R5 .......... .......... .......... .......... .......... ..........

gTvQR1_R1 .......... .......... .......... .......... .......... ..........

gTvQR1_r5 .......... .......... .......... .......... .......... ..........

gTvQR1_R2 .......... .......... .......... .......... .......... ..........

gTvQR1_N3 .......... .......... .......... .......... .......... ..........

gTvQR1_N2 .......... .......... .......... .......... .......... ..........

gTvQR1_n1 .......... .......... .......... .......... .......... ..........

gTvQR1_n2 .......... .......... .......... .......... .......... ..........

gTvQR1_n3 .......... .......... .......... .......... .......... ..........

cTvQR1_7 .......... .......... .......... .......... .P........ ..........

cTvQR1_8 .......... .......... .......... .......... .......... ..I.......

gTvQR1_r8 .......... .......... .......... .......... .......... ..I.......

cTvQR1_2 .......... .......... .......... .......... .......... ..........

gTvQR1_r3 .......... .......... .......... .......... .......... ..........

gTvQR1_r6 .......... .......... .......... .......... .......... ..........

cTvQR1_6 .......... .......... .......... ....S..... .......... ..........

gTvQR1_r1 .......... .......... .......... .......... .......... ..........

gTvQR1_R3 .......... .......... .......... .......... .......... ..........

gTvQR1_N1 .......... .......... .......... .......... .......... ..........

gTvQR1_r4 .......... .......... .......... .......... .......... ..........

gTvQR1_r7 .......... .......... ........S. .......... .......... ..........

181 240

cTvQR1_1 THVTATCGSR NFDLVKSLGA DEVIDYKTPE GAALKSPSGK KYDAVIHCAS PLPWSVFKPN

cTvQR1_9 .......... .......... .......... .......... .......... ..........

cTvQR1_3 .......... .......... .......... .......... .......... ..........

cTvQR1_5 .......... .......... .......... .......... .......... ..........

cTvQR1_4 .......... .......... N......... .......... .......... ..........

gTvQR1_r2 .......... .......... .......... .......... .......... ..........

gTvQR1_R4 .......... .......... .......... .......... .......... ..........

gTvQR1_R6 .......... .......... .......... .......... .......... ..........

gTvQR1_R5 .......... .......... .......... .......... .......... ..........

gTvQR1_R1 .......... .......... .......... .......... .......... ..........

gTvQR1_r5 .......... .......... .......... .......... .......... ..........

gTvQR1_R2 .......... .......... .......... .......... .......... ..........

gTvQR1_N3 .......... .......... .......... .......... .......... ..........

gTvQR1_N2 .......... .......... .......... .......... .......... ..........

gTvQR1_n1 .......... .......... .......... .......... .......... ..........

gTvQR1_n2 .......... .......... .......... .......... .......... ..........

gTvQR1_n3 .......... .......... .......... .......... .......... ..........

cTvQR1_7 .......... .......... .......... .......... .......... ..........

cTvQR1_8 .......... .......... .......... ....T..... .......... ..........

gTvQR1_r8 .......... .......... .......... ....T..... .......... ..........

cTvQR1_2 .......... .......... .......... .......... .......... ..........

gTvQR1_r3 .......... .......... .......... .......... .......... ..........

gTvQR1_r6 .......... .......... .......... .......... .......... ..........

cTvQR1_6 .......... .......... .......... .......... .......... ..........

gTvQR1_r1 .......... .......... .......... .......... .......... ..........

gTvQR1_R3 .......... .......... .......... .......... .......... ..........

gTvQR1_N1 .......... .......... .......... .......... .......... ..........

gTvQR1_r4 .......... .......... .......... .......... .......... ..........

gTvQR1_r7 .......... .......... .......... .......... .......... ..........

241 300

cTvQR1_1 LSKHGKVIDI TPGPRVMLTS AMTKLTCSKK RLVTLLVVIK GEHLSYLVEL MREGKLKTVI

cTvQR1_9 .......... .......... .......... .......... .......... ..........

cTvQR1_3 .......... .......... .......... .......... .......... ..........

cTvQR1_5 .......... .......... .......... .......... .......... ..........

cTvQR1_4 .......... .......... .......... .......... .......... ..........

gTvQR1_r2 ...R...... .......... .......... .......... .......... ..........

gTvQR1_R4 ...R...... .......... .......... .......... .......... ..........

gTvQR1_R6 ...R...... .......... .......... .......... .......... ..........

gTvQR1_R5 ...R...... .......... .......... .......... .......... ..........

gTvQR1_R1 ...R...... .......... .......... .......... .......... ..........

gTvQR1_r5 ...R...... .......... .......... .......... .......... ..........

gTvQR1_R2 .......... .......... .......... .......... .......... ..........

gTvQR1_N3 .......... .......... .......... .......... .......... ..........

gTvQR1_N2 .......... .......... .......... .......... .......... ..........

gTvQR1_n1 .......... .......... .......... .......... .......... ..........

gTvQR1_n2 .......... .......... .......... .......... .......... ..........

gTvQR1_n3 .......... .......... .......... ..E....... ........Q. ..........

cTvQR1_7 .......... .......... .......... .......... .......... ..........

cTvQR1_8 .......... .......... .......... .......... ........G. ..........

gTvQR1_r8 .......... .......... .......... .......... ........G. ..........

cTvQR1_2 .......... .......... .......... .......... .......... ..........

gTvQR1_r3 .......... .......... .......... .......... ........K. ..........

gTvQR1_r6 .......... .......... .......... .......... .......... ..........

cTvQR1_6 .......... .......... .......... .......... .......... ..........

gTvQR1_r1 .......... .......... .......... .......... .......... ..........

gTvQR1_R3 .......... .......... .......... .......... .......... ..........

gTvQR1_N1 .......... .......... .......... .......... ........G. ..........

gTvQR1_r4 .......... .......... .......... .......... D.......G. ......R...

gTvQR1_r7 .......... .......... .......... .......... ........G. ..........

301 329

cTvQR1_1 DSKFSLSKAE EAWAKSIDGH ATGKIVVEP

cTvQR1_9 .......... .......... .........

cTvQR1_3 ....P..... .......... .........

cTvQR1_5 ....P..... .......... .........

cTvQR1_4 ....P..... .......... .........

gTvQR1_r2 ....P..... .......... .........

gTvQR1_R4 ....P..... .......... .........

gTvQR1_R6 ....P..... .......... .........

gTvQR1_R5 ....P..... .......... .........

gTvQR1_R1 ....P..... .......... .........

gTvQR1_r5 ....P..... .......... .........

gTvQR1_R2 ....P..... .......... .........

gTvQR1_N3 ....P..... .......... .........

gTvQR1_N2 ....P..... .......... .........

gTvQR1_n1 ....P..... .......... .........

gTvQR1_n2 ....P..... .......... .........

gTvQR1_n3 ....P..... .......... .........

cTvQR1_7 ....P..... .......... .........

cTvQR1_8 .......... .......... .........

gTvQR1_r8 .......... .......... .........

cTvQR1_2 ....P..... .......... .........

gTvQR1_r3 ....P..... .......... .........

gTvQR1_r6 ....P..... .......... .........

cTvQR1_6 ....P..... .......... .........

gTvQR1_r1 ....P..... .......... .........

gTvQR1_R3 ....P..... .......... .........

gTvQR1_N1 ....L..... .......... ......I..

gTvQR1_r4 ....P..... .......... .........

gTvQR1_r7 .......... .......... .........
