# Supplementary material for: The differentially regulated genes TvQR1 and TvPirin of the parasitic plant Triphysaria exhibit distinctive natural allelic diversity
Source: BMC Plant Biol. 2013 Feb 18;13:28. doi: 10.1186/1471-2229-13-28 (PMC3599707; doi:10.1186/1471-2229-13-28)
Supplement: Additional file 8 — TvPirin protein domains and amino acid diversity. [file 1471-2229-13-28-S8.pdf]

Additional file 8. TvPirin protein domains and amino acid diversity

A. PFAM domains in the TvPirin protein

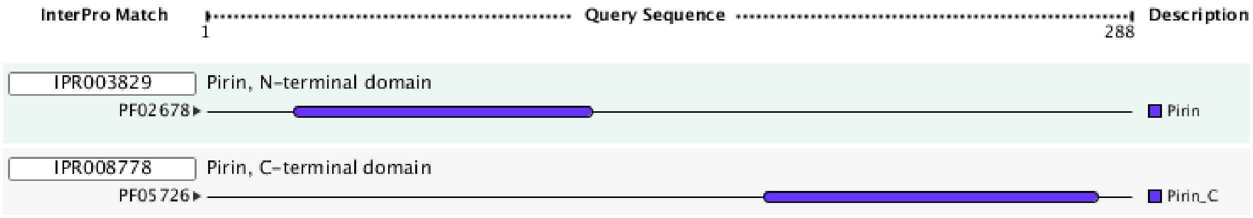

B. Multiple sequence alignment of deduced TvPirin proteins from all full-length cDNA and genomic *TvPirin* clones

The ORFs of 4 cDNA alleles (cTvPir_#) and 29 genomic alleles (gTvPir_#) are aligned. Homozygotes or heterozygotes with identical aa sequences for both alleles are presented as single protein alleles. Heterozygotes with different aa sequences for both alleles are designated with “A” and “B” at the end of sequence names. Dots represent identical residues.

```
             1                                                          60
  cTvPir_1   MSSFQQPRLV AKKISAKSQS EGDGALVRRS IGRPELKSLD PFLLLDEFAV TPPAGFPDHP
gTvPir_r1A   .......... ....L..... .......... .......... .......... ..........
gTvPir_r1B   .......... ....L..... .......... .......... .......... ..........
 gTvPir_N1   .......... ....L..... .......... .......... .......... ..........
gTvPir_n3B   .......... ....L..... .......... .......... .......... ..........
gTvPir_n3A   .......... ....L..... .......... .......... .......... ..........
 gTvPir_n2   .......... ....L..... .......... .......... .......... ..........
 gTvPir_n1   .......... ....L..... .......... .......... .......... ..........
gTvPir_R6B   .......... ....L..... .......... .......... .......... ..........
 gTvPir_N3   .......... ....L..... .......... .......... .......... ..........
gTvPir_R5B   .......... ....L..... .......... .......... .......... ..........
gTvPir_R5A   .......... ....L..... .......... .......... .......... ..........
 gTvPir_r2   .......... ....L..... .......... .......... .......... ..........
gTvPir_R4A   .......... ....L..... .......... .......... .......... ..........
 gTvPir_r3   .......... ....L..... .......... .......... .......... ..........
gTvPir_R3A   .......... ....L..... .......... .......... .......... ..........
gTvPir_R2B   .......... ....L..... .......... .......... .......... ..........
gTvPir_R2A   .......... ....L..... .......... .......... .......... ..........
 gTvPir_R1   .......... ....L..... .......... .......... .......... ..........
 gTvPir_r8   .......... ....L..... .......... .......... .......... ..........
gTvPir_r7B   .......... ....L..... .......... .......... .......... ..........
gTvPir_r4A   .......... ....L..... .......... .......... .......... ..........
 gTvPir_r6   .......... ....L..... .......... .......... .......... ..........
 gTvPir_r5   .......... ....L..... .......... .......... .......... ..........
gTvPir_r4B   .......... ....L..... .......... .......... .......... ..........
gTvPir_r7A   .......... ....L..... .......... .......... .......... ..........
gTvPir_R3B   .......... ....L..... .......... .......... .......... ..........
gTvPir_R4B   .......... ....L..... .......... .......... .......... ..........
gTvPir_R6A   .......... ....L..... .......... .......... .......... ..........
 gTvPir_N2   .......... ....L..... .......... .......... .......... ..........
  cTvPir_2   .......... ....L..... .......... .......... .......... ..........
  cTvPir_3   .......... ....L..... .......... .......... .......... ..........
  cTvPir_4   .......... ....L..... .......... .......... .......... ..........
```

```
             61                                                         120
  cTvPir_1   HRGFETVTYM LQGGVTHQDF AGHKGTIGAG DVQWMTAGRG IVHSEMPAGE GTHTGLQLWI
gTvPir_r1A   .......... .......... .......... .......... .......... ..........
gTvPir_r1B   .......... .......... .......... .......... .......... ..........
 gTvPir_N1   .......... .......... .......... .......... .......... ..........
gTvPir_n3B   .......... .......... .......... .......... .......... ..........
gTvPir_n3A   .......... .......... .......... .......... .......... ..........
 gTvPir_n2   .......... .......... .......... .......... .......... ..........
 gTvPir_n1   .......... .......... .......... .......... .......... ..........
gTvPir_R6B   .......... .......... .......... .......... .......... ..........
 gTvPir_N3   .......... .......... .......... .......... .......... ..........
gTvPir_R5B   .......... .......... .......... .......... .......... ..........
gTvPir_R5A   .......... .......... .......... .......... .......... ..........
 gTvPir_r2   .......... .......... .......... .......... .......... ..........
gTvPir_R4A   .......... .......... .......... .......... .......... ..........
 gTvPir_r3   .......... .......... .......... .......... .......... ..........
gTvPir_R3A   .......... .......... .......... .......... .......... ..........
gTvPir_R2B   .......... .......... .......... .......... .......... ..........
gTvPir_R2A   .......... .......... .......... .......... .......... ..........
 gTvPir_R1   .......... .......... .......... .......... .......... ..........
 gTvPir_r8   .......... .......... .......... .......... .......... ..........
gTvPir_r7B   .......... .......... .......... .......... .......... ..........
gTvPir_r4A   .......... .......... .......... .......... .......... ..........
 gTvPir_r6   .......... .......... .......... .......... .......... ..........
 gTvPir_r5   .......... .......... .......... .......... .......... ..........
gTvPir_r4B   .......... .......... .......... .......... .......... ..........
gTvPir_r7A   .......... .......... .......... .......... .......... ..........
gTvPir_R3B   .......... .......... .......... .......... .......... ..........
gTvPir_R4B   .......... .......... .......... .......... .......... ..........
gTvPir_R6A   .......... .......... .......... .......... .......... ..........
 gTvPir_N2   .......... .......... .......... .......... .......... ..........
  cTvPir_2   .......... .......... .......... .....A.... ...L...... ..........
  cTvPir_3   .......... .......... .......... .......... .......... ..........
  cTvPir_4   .......... .......... .......... .......... .......... ..........
```

```
            121                                                        180
  cTvPir_1  NLSSKDKMIE PRYQELLSND IPKSQKDGVE VKVIAGEAMG VHSQVFTRTP TMYVDFTLKP
gTvPir_r1A  .......... .......... ...A...... .......... .......... ..........
gTvPir_r1B  .......... .......... ...A...... .......... .......... ..........
 gTvPir_N1  .......... .......... ...A...... .......... .......... ..........
gTvPir_n3B  .......... .......... ...A...... .......... .......... ..........
gTvPir_n3A  .......... .......... ...A...... .......... .......... ..........
 gTvPir_n2  .......... .......... ...A...... .......... .......... ..........
 gTvPir_n1  .......... .......... ...A...... .......... .......... ..........
gTvPir_R6B  .......... .......... ...A...... .......... .......... ..........
 gTvPir_N3  .......... .......... ...A...... .......... .......... ..........
gTvPir_R5B  .......... .......... ...A...... .......... .......... ..........
gTvPir_R5A  .......... .......... ...A...... .......... .......... ..........
 gTvPir_r2  .......... .......... ...A...... .......... .......... ..........
gTvPir_R4A  .......... .......... ...A...... .......... .......... ..........
 gTvPir_r3  .......... .......... ...A...... .......... .......... ..........
gTvPir_R3A  .......... .......... ...A...... .......... .......... ..........
gTvPir_R2B  .......... .......... ...A...... .......... .......... ..........
gTvPir_R2A  .......... .......... ...A...... .......... .......... ..........
 gTvPir_R1  .......... .......... ...A...... .......... .......... ..........
 gTvPir_r8  .......... .......... ...A...... .......... .......... ..........
gTvPir_r7B  .......... .......... ...A...... .......... .......... ..........
gTvPir_r4A  .......... .......... ...A...... .......... .......... ..........
 gTvPir_r6  .......... .......... ...A...... .......... .......... ..........
 gTvPir_r5  .......... .......... ...A...... .......... .......... ..........
gTvPir_r4B  .......... .......... ...A...... .......... .......... ..........
gTvPir_r7A  .......... .......... ...A...... .......... .....Y.... ..........
gTvPir_R3B  .......... .......... ...A...... .......... .......... ..........
gTvPir_R4B  .......... .......... ...A...... .........C .......... ..........
gTvPir_R6A  .......... .......... ...A...... .........C .......... ..........
 gTvPir_N2  .......... .......... ...A...... .........C .......... ..........
  cTvPir_2  .......... .......... ...A...... .......... .......... ..........
  cTvPir_3  .......... ..H....... ...A...... .......... ..P....... ..........
  cTvPir_4  .......... .......... ...A...... ...V...... ......P... ..........
```

```
             181                                                         240
   cTvPir_1  NSEYHQGIPD SWNAFVYIIE GEGVFGIPNS KPAEAHHILV LGPGEGLSVW NKFTEPLRFI
 gTvPir_r1A  .......... .......... .......... .......... .......... ..........
 gTvPir_r1B  .......... .......... .......... .......... .......... ..........
  gTvPir_N1  .......... .......... .......... .......... .......... ..........
 gTvPir_n3B  .......... .......... .......... .......... .......... ..........
 gTvPir_n3A  .......... .......... .......... .......... .......... ..........
  gTvPir_n2  .......... .......... .......... .......... .......... ..........
  gTvPir_n1  .......... .......... .......... .......... .......... ..........
 gTvPir_R6B  .......... .......... .......... .......... .......... ..........
  gTvPir_N3  .......... .......... .......... .......... .......... ..........
 gTvPir_R5B  .......... .......... .......... .......... .......... ..........
 gTvPir_R5A  .......... .......... .......... .......... .......... ..........
  gTvPir_r2  .......... .......... .......... .......... .......... ..........
 gTvPir_R4A  .......... .......... .......... .......... .......... ..........
  gTvPir_r3  .......... .......... .......... .......... .......... ..........
 gTvPir_R3A  .......... .......... .......... .......... .......... ..........
 gTvPir_R2B  .......... .......... .......... .......... .......... ..........
 gTvPir_R2A  .......... .......... .......... .......... .......... ..........
  gTvPir_R1  .......... .......... .......... .......... .......... ..........
  gTvPir_r8  .......... .......... .......... .......... .......... ..........
 gTvPir_r7B  .......... .......... .......... .......... .......... ..........
 gTvPir_r4A  .......... .......... .......... .......... .......... ..........
  gTvPir_r6  .......... .......... .......... .......... .......... ..........
  gTvPir_r5  .......... .......... .......... .......... .......... ..........
 gTvPir_r4B  .......... .......... .......... .......... .......... ..........
 gTvPir_r7A  .......... .......... .......... .......... .......... ..........
 gTvPir_R3B  .......... .......... .......... .......... .......... ..........
 gTvPir_R4B  .......... .......... .......... .......... .......... ..........
 gTvPir_R6A  .......... .......... .......... .......... .......... ..........
  gTvPir_N2  .......... .......... .......... .......... .......... ..........
   cTvPir_2  .......... .......... .......... .......... .......... ..........
   cTvPir_3  D......... .......... .......... .......... .......... ..........
   cTvPir_4  .......... .......... .......... .......... .......... ..........
```

```
              241                                                288
   cTvPir_1  LVGGQPLNEP VVQHGPFVMN THAEIDKTME DYYYGKNGFE LAKKWKSK
 gTvPir_r1A  .......... .......... .......... .......... ........
 gTvPir_r1B  .......... .......... .......... .......... ........
  gTvPir_N1  .......... .......... .......... .......... ........
 gTvPir_n3B  .......... .......... .......... .......... ........
 gTvPir_n3A  .......... .......... .......... .......... ........
  gTvPir_n2  .......... .......... .......... .......... ........
  gTvPir_n1  .......... .......... .......... .......... ........
 gTvPir_R6B  .......... .......... .......... .......... ........
  gTvPir_N3  .......... .......... .......... .......... ........
 gTvPir_R5B  .......... .......... .......... .......... ........
 gTvPir_R5A  .......... .......... .......... .......... ........
  gTvPir_r2  .......... .......... .......... .......... ........
 gTvPir_R4A  .......... .......... .......... .......... ........
  gTvPir_r3  .......... .......... .......... .......... ........
 gTvPir_R3A  .......... .......... .......... .......... ........
 gTvPir_R2B  .......... .......... .......... .......... ........
 gTvPir_R2A  .......... .......... .......... .......... ........
  gTvPir_R1  .......... .......... .......... .......... ........
  gTvPir_r8  .......... .......... .......... .......... ........
 gTvPir_r7B  .......... .......... .......... .......... ........
 gTvPir_r4A  .......... .......... .......... .......... ........
  gTvPir_r6  .......... .......... .......... .......... ........
  gTvPir_r5  .......... .......... .......... .......... ........
 gTvPir_r4B  .......... .......... .......... .......... ........
 gTvPir_r7A  .......... .......... .......... .......... ........
 gTvPir_R3B  ......R... .......... .......... .......... ........
 gTvPir_R4B  .......... .......... .......... .......... ........
 gTvPir_R6A  .......... .......... .......... .......... ........
  gTvPir_N2  .......... .......... .......... .......... ........
   cTvPir_2  .......... .......... .......... .......... ........
   cTvPir_3  .......... .......... .......... .......... ........
   cTvPir_4  ..C....... .......... .......... .......... ........
```
